# A generalized scaling law reveals synchrony-driven reorganization of brain dynamics across human lifespan

**DOI:** 10.1371/journal.pcbi.1014821

**Published:** 2026-10-05

**Authors:** Suman Saha, Chittaranjan Hens, Priyanka Chakraborty, Tomasz Kapitaniak, Gustavo Deco, Arpan Banerjee, Syamal Kumar Dana, Dipanjan Roy

**Affiliations:** 1 School of Electronics Engineering, Vellore Institute of Technology, Chennai, Tamil Nadu, India; 2 Center for Computational Natural Sciences and Bioinformatics, International Institute of Information Technology, Hyderabad, Telangana, India; 3 Biomedical Research Center, International Institute of Information Technology, Hyderabad, Telangana, India; 4 Department of Mathematics, Rampurhat College, Rampurhat, West Bengal, India; 5 Division of Dynamics, Technical University of Lodz, Lodz, Poland; 6 Centre for Brain and Cognition, Computational Neuroscience Group, Universitat Pompeu Fabra, Barcelona, Spain; 7 Department of Information and Communication Technologies, Universitat Pompeu Fabra, Barcelona, Spain; 8 Cognitive Brain Dynamics Lab, National Brain Research Centre, Manesar, Haryana, India; 9 Centre for Mathematical Biology and Ecology, Department of Mathematics, Jadavpur University, Kolkata, West Bengal, India; 10 Department of Bioscience and Bioengineering, Indian Institute of Technology, Jodhpur, Rajasthan, India; Monash University, AUSTRALIA

## Abstract

Ecological systems obey universal scaling laws such as Taylor’s law, which links population variance to the sample mean. Whether other complex systems with integrated and segregated network topology, the human brain, in particular, follow analogous scaling laws, and how these laws behave under synchronized neural activity, remains unknown. To address this question, our initial attempt to implement the Taylor’s law, has been hindered because neural signals at all measurement scales contain both positive and negative values, whereas Taylor’s law was restricted to non-negative ecological data. To circumvent this limitation, we extend spatial Taylor’s law by replacing the sample mean with the root-mean-square (RMS) of the measurements, and apply this generalized law to detrended multivariate neural time series. The detrending operation has been performed as a necessary step to remove the trends and temporal mean from the data. Within this framework, we address our next question how synchronous or coordinated activity of the brain network influences the scaling law. We quantify synchrony using a dedicated metric of functional coordination and show analytically, and via numerical simulations using multivariate Poisson, negative binomial, uniform and gamma distributions, that the scaling exponent is inversely related to synchrony. Applying this approach to human fMRI data from three large cohorts (*N* = 840, ages 18–88 years) reveals distinct age-related trajectories of the scaling exponent, with healthy aging characterized by a substantial synchrony-induced reduction in this exponent. The synchrony–scaling relationship remains stable during resting-state activity but progressively shifts during naturalistic tasks across the lifespan, and is pronounced in limbic, subcortical, and cerebellar subnetworks, indicating preservation at subnetwork levels. Finally, individuals with ADHD exhibit altered synchrony–scaling coupling, highlighting the potential clinical and translational utility of this generalized scaling metric.

## Author summary

The human brain constantly reorganizes the way its regions coordinate with one another, yet a general, quantitative way to link this coordination to the brain’s variability has been missing. Classical scaling laws from ecology, such as Taylor’s law, connect the variance of a population to its mean, but they only work for non-negative data distributions and therefore could not be applied directly to brain signals, which fluctuate above and below zero once slow trends are removed. Here we generalize this scaling law by replacing the mean with the root-mean-square of detrended brain activity, which lets us apply it to the blood-oxygen-level-dependent activity (BOLD). Using analytical, simulations, and functional Magnetic Resonance Imaging (fMRI) data from 840 participants aged 18–88 years, we show that the strength of coordinated activity between brain regions, captured by average synchrony, systematically changes how the variance of brain activity scales in space. The average synchrony-scaling law association is stable in the resting state but changes during naturalistic tasks, such as movie watching or sensorimotor tasks, becomes weaker with healthy brain aging, and is disrupted in people with Attention-Deficit/Hyperactivity Disorder (ADHD). Our findings introduce a compact, interpretable, and broadly applicable metric that links long-term coordination to the variability of neural activity, with potential use as a marker of healthy aging and neurological or psychiatric disorders.

## Introduction

Complex systems often exhibit scale-invariant fluctuations [1–5], as also reported in the human brain [6–9], yet a unifying law is lacking that connects such variability to the coordinated dynamics of the human brain, particularly across different mental states, healthy aging, and neurological disorders.

On the other hand, a universal scaling relation such as Taylor’s law [1] is available, in the literature, that links mean and variance in non-negative ecological counts, however, this cannot be directly applicable to signed, detrended neural signals that spread over negative and positive values. This hinders our initial attempt to explain how large-scale coordinated dynamics shapes brain-wide variability in neuronal activity and limits our ability to interpret ubiquitous power-law signatures in neuroimaging [8,10–13] within a common theoretical framework and to relate them to healthy lifespan aging and brain disorders.

Taylor’s scaling law (TL), also known as fluctuation scaling, has been verified with non-negative data in diverse complex systems, including ecology [1,14–18], physics [3,19–22], climatology [23,24], economics [25], real-world networks [5,26–30], human population [4,31], life science [32–34], dynamical systems [35] and neuroscience [10]. TL is described by a scalar relation between variance (*v*) and mean (μ) of non-negative data distributions, $v=a\mu^{b}$, where *a* is a constant and *b* is the exponent, predominantly existing in the range [1/2, 1] [36]. Upon a logarithmic transformation, the equation becomes log(v)=blog(μ)+log(a). While obeying the power law, since there is no characteristic scale (size) for the phenomena under investigation, it is referred to as *scale-free* [37,38], Among the two variants of TL, *temporal* and *spatial*, we focus on the spatial TL that takes mean and variance of measurements over locations at each time point. This concept of spatial scaling inspired us to investigate the effect of coordinated brain activity on the scale-invariant property of BOLD signals, if any and to establish a theoretically grounded biomarker of healthy aging and neural disorders with necessary modifications.

Firstly, we replace the sample mean with the spatial root-mean-square (RMS) of measurement data and thereby extend the application of the spatial Taylor’s scaling law to multivariate signals with positive and negative fluctuations. This simple reformulation yields a general scaling law now applicable to multivariate data across various disciplines and removes the hinderance of our search for a scaling law in neural activity. We are able to derive a closed-form link between a spatial scaling exponent and average spatial synchrony, allowing a coordinated dynamics to be read out from scale-free variability in a principled way. We make a combined approach of analytical derivations, simulations and testing with large cohort spanning the adult lifespan. A detrending operation is applied to multivariate data to remove any non-stationarity present in empirical data. Our results reveal that synchrony-driven changes in the scaling exponent track age-dependent reconfiguration of brain-wide coordination, that remain stable at rest but reorganize during naturalistic tasks, and are selectively disrupted in neuropsychiatric conditions. By connecting coordination dynamics, fluctuation scaling and brain health within a single framework, our results position synchrony-induced scaling laws as a compact and general marker for complex brain dynamics.

While studying brain-wide coordinated dynamics of the BOLD activity, the multivariate data analysis significantly enhances the sensitivity of the human neuroimaging data [39–41] compared to conventional single-location-based approaches, e.g., temporal scaling law [10] or temporal autocorrelation [42]. Nevertheless, coordinated neural activity or the collective dynamical behavior is often characterized by the global metric, average spatial synchrony [43–49]. It quantifies the tendency of neural population to be correlated through time across the distributed cortical regions [47–49]. More generally, a coherent collective dynamics emerges in a network of dynamical units, as widely studied across disciplines, including physics [46], ecology [50–53], and engineered systems [54].

Available evidence from ecology suggests that emergent coordinated dynamics among spatially separated populations, measured by the average synchrony– as exemplified by the Moran effect, can significantly alter the spatial TL exponent [2,55]. While theoretical and empirical links between average synchrony and the scaling law (SL) have been realized [2,36,53,56,57], prior works overlooked the bias introduced by the temporal mean and trends that are common in real-time or empirical data obtained from natural and experimental systems [58,59]. We revisit this fundamental problem in data analysis that may have obscured the contributions coming from internal or external stimuli in neuroscience, or Moran effect in ecology, rather than any random processes. We specifically ask if the bias in measurement data is removed, how the presence and strength of the brain-wide coordinated activity influence the slope *b* of TL, and if so, how? The detrending operation [58] alleviates the bias, ensuring stationarity and preserving spectral and autocorrelation properties, while transforming data to span from negative to positive values; a methodological aspect overlooked in earlier works on fluctuation scaling. This development prompts an important question: Can the spatial scaling law be re-formulated to accommodate distributions containing both negative and positive values? This question also becomes crucial when examining how synchrony governs universal scaling laws in complex systems, particularly, in the context of applying Taylor’s law in brain dynamics; this relationship remains largely unexplored. Extending Taylor’s law from its traditional domain of non-negative data distributions is therefore a crucial step forward to encompass the full range of values after detrending, while effectively isolating the effects of trends and temporal means in brain signals. Indeed, such an extension of the spatial Taylor’s scaling law makes it available for fluctuation analysis in other disciplines, including many physical and experimental systems. To implement this generic application of Taylor’s law, as mentioned above, we simply replace the sample mean by spatial root-mean-square (RMS), and derive a revised scaling law between the spatial RMS and sample variance. Although detrended fluctuation analysis has been widely employed in climatology, extreme-event studies [59,60], the scaling behavior of spatial aggregation in detrended data with suppressed temporal mean and trends remains unexplored. More importantly, the effect of synchrony on spatial scaling was not widely explored earlier, except in the context of population segregation in ecology. Only recently, studies with coupled oscillators showed [35] a clear connection between synchrony and Taylor’s scaling law.

The brain-wide coordinated hemodynamics, in general, has been formalized as a temporal alignment or co-ordination among distributed cortical regions during specific functional states [47–49]. The extent of coordinated hemodynamics or blood-oxygen-level-dependent (BOLD) activity, obtained from functional magnetic resonance imaging (fMRI), or the degree of correlation is quantified by a global metric, namely, average spatial synchrony by averaging functional connectivity (FC). In this work, the average synchrony is measured by the variance of spatial mean of the BOLD signals. The FC is obtained from pairwise Pearson correlations between the BOLD signals (typically spanning 10 minutes) across all cortical regions [61–69]. The average synchrony is elicited in response to internal or external stimuli, and holds significant clinical relevance [70–73]. Earlier studies investigated temporal correlations in brain oscillations as physiological correlates of behavior, highlighting scale-free $1/f^{\beta }$ noise as a hallmark of complexity [74], and reporting long-range correlations using the measure of synchronization errors during human coordination tasks [75]. Research utilizing EEG and MEG data has demonstrated self-organized criticality in brain oscillations through detrended fluctuation analysis and frequency–power scaling, suggesting that critical-state dynamics enable neural network reorganization [76]. Furthermore, analyses of healthy human fMRI data revealed that temporal TL exponents change with age and vary across brain regions [10]. Yet, a substantial gap remains concerning the generic role of synchrony-induced spatial scaling across diverse brain states, particularly, in relation to healthy aging and neural disorders. Building upon the revised scaling law, we investigate how coordinated neural dynamics. indexed by the average spatial synchrony, shape the fluctuation scaling exponent, and what this reveals about the empirical and theoretical links between the synchrony and spatial scaling in the human brain. By quantifying coherent BOLD activity across multiple brain regions and subnetworks, we assess how aging affects synchrony-induced scaling across different task conditions throughout the lifespan, comprising both normative aging and neural disorders.

## Results

Our approach is illustrated in Fig 1 using exemplary BOLD signals from a single participant. The multivariate time series data of size T×n (where *T* is total time length; *n* is brain regions or locations) is first detrended. Time series from four locations (*n* = 4) are presented in green, red, blue and yellow. The detrending operation removes additional trends and sets the temporal mean to zero from each time series. The detrended data $Y_{i}(t)$ at each instant *t* in all locations follows a near-normal distribution. Next, amplitudes of the detrended BOLD signal of all *t*he brain regions are squared individually and summed at a time instant *t* to obtain spatial RMS (*m*) at time *t*. Similarly, we store *t*he spatial variance (*v*) at each time instant. The spa*t*ial SL exponent (*b*) is derived from the linear relationship between $\log_{10}(m)$ and $\log_{10}(v)$ taken at different time instants. Long-term correlation of measurements between a pair of locations is derived by Pearson correlation coefficient, giving a correlation matrix. Average spatial synchrony or average synchrony Ω is the var(μ(t)) (where μ is the sample mean) derived from the pair-wise correlation matrix, see S1 Appendix for detailed derivation. The association slope, β is finally obtained by a linear regression fit that describes the influence of synchrony Ω on the spatial SL exponent *b*. We rounded up to 1e-6 decimal values in all our simulations. For detailed derivations and descriptions, see Methodology section and supporting information (S1 Appendix).

**Fig 1:**
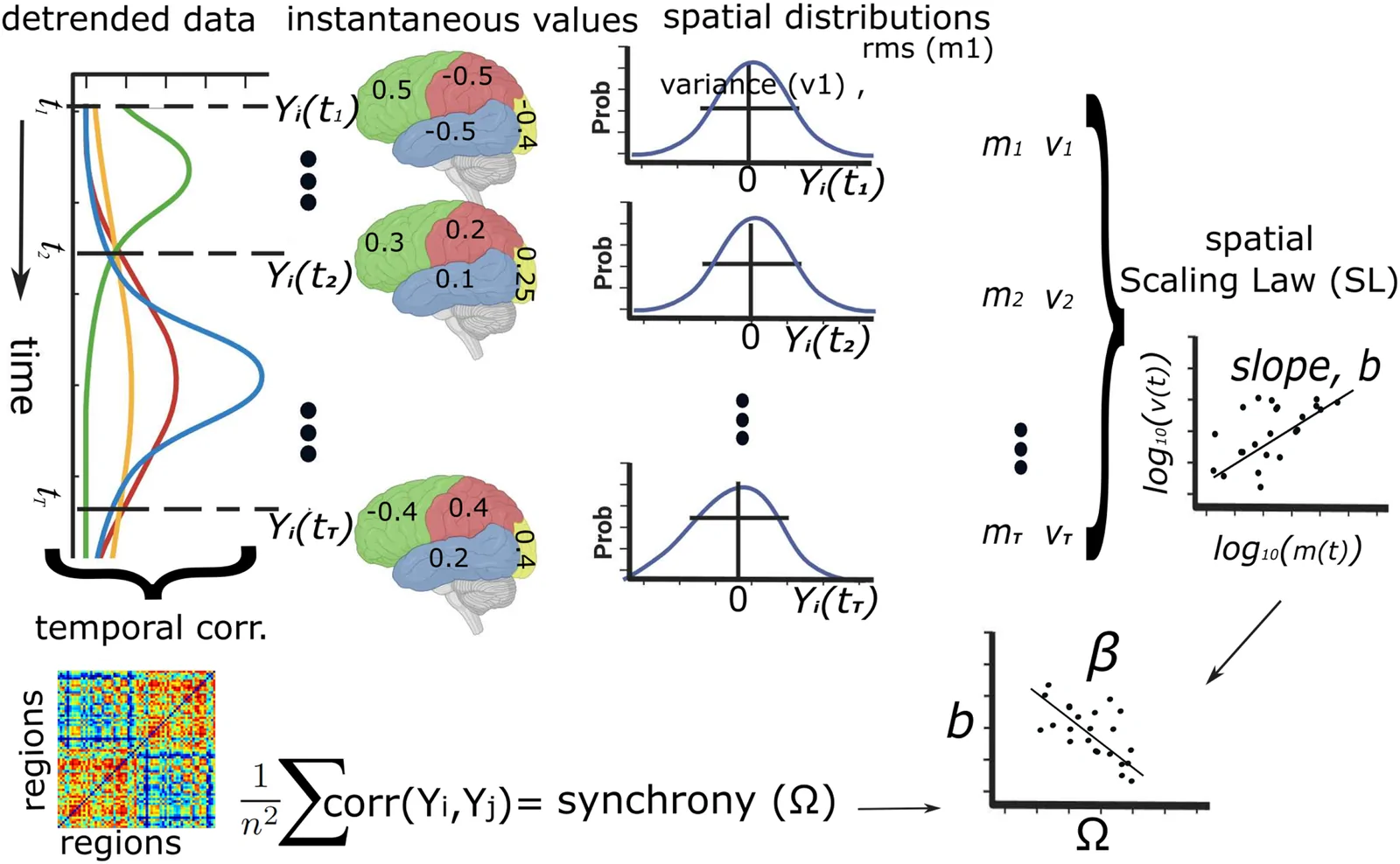
Schematic of the synchrony-scaling analysis pipeline applied to detrended spatio-temporal BOLD data. Multivariate BOLD time series from *n* brain regions are detrended to remove temporal means and trends, yielding zero-mean signals $Y_{i}(t)$ with near-normal spatial distributions at each time instant ***t*.** The spatial RMS (*m*) and variance (*v*) are derived from spatial distributions over the entire space at each time point, e.g., *m*_1_ and *v*_1_ (where the subscript denotes the time index) are the spatial RMS and variance, respectively, derived at time point *t* = *t*_1_. The log-log linear relationship between *t*he spa*t*ial RMS and variance defines the spatial scaling law (SL) exponent ***b*.** Average spatial synchrony Ω is derived as the average of the pairwise inter-regional correlation matrix. The association slope β, obtained by regressing *b* against Ω, quantifies the influence of synchrony on the spatial scaling exponent.

### Numerical simulations: Effect of synchrony on the scaling law

For modeling correlated local dynamics, we generate multivariate time series data from a zero-mean Gaussian distribution with a specified covariance matrix. The time series dimension is *T* = 261 time points and *n* = 116 regions, matching empirical data as discussed below.

Fig 2a and 2b show the log-normal distributions of spatial RMS log(m) and variance log(v), in logarithmic scale, respectively, as exemplary normal distributions 𝒩(0,1). The spatial fluctuation scaling between *m* and *v* is shown in a $\log_{10}$-vs-$\log_{10}$ scatterplot in Fig 2c in the case of i.i.d. data, when long-term correlations between any two locations, ρ=corr$(Y_{i},Y_{j})≃0$ for i≠j. The exponent is estimated from semi-analytical and linear fitting as *b* = 1.988 and 1.991, respectively, shown in Fig 2c. The correlation between the spatial SL (*b*) and average synchrony (Ω) is illustrated in Fig 2d. The spatial SL exponent remains near 2 under near-zero average synchrony, confirming that the underlying dynamics originate from a purely stochastic process in the absence of inter-regional coordination. For identically distributed but not necessarily independent data, the synchrony substantially influences the spatial SL exponent; see Fig 2e–2g for ρ=0.001, 0.01, and 0.1. The spatio-temporal time series data is constructed depending on the correlation value ρ using the *common element method* [77]; see SI for details and other distributions. Fig 2h shows that an increasing degree of synchrony induces a monotonic decrease of the SL slope as obtained from analytical results using Eq. (6). The inset shows the fitting errors quantified by root mean squared errors (RMSE) between the actual log(v) values and blog(m) values from the linear regression of log(m)-vs-log(v). The spatial SL exponent, based on spatial RMS and variance, thus decreases monotonically with the degree of synchrony. This is independent of distributions of data as numerically confirmed with other distributions, namely, Poisson, negative binomial, uniform, and gamma distributions (see Fig B-Fig M in S1 Appendix for detailed results). Note that irrespective of the original distribution of measurements, after the detrending operation, the data follow a normal distribution.

**Fig 2:**
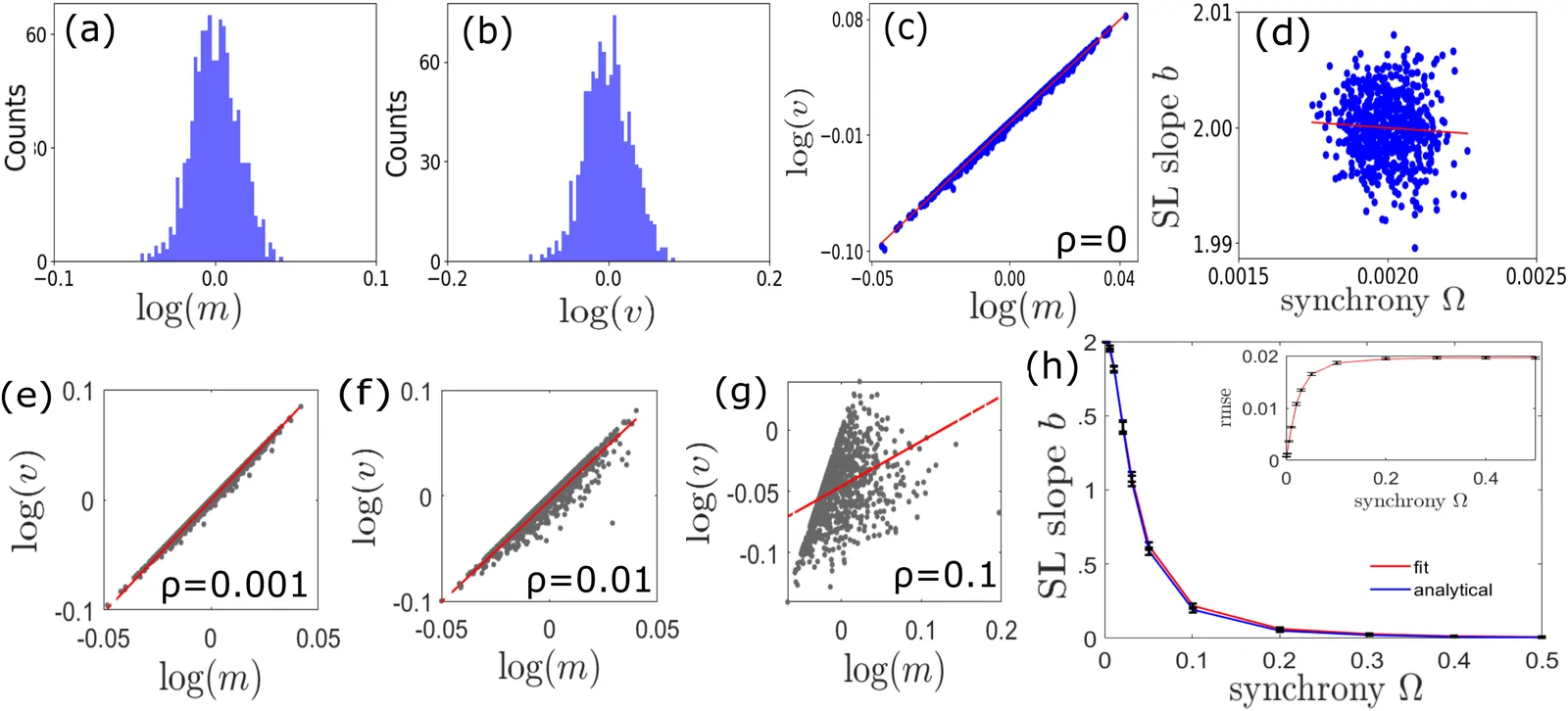
Numerical example: Analysis of spatio-temporal time series. Time series data at each location, generated by randomly drawn values from normal distribution. **(a, b)** Log-normal distributions of spatial RMS and variance. **(c)** Linear fit of spatial log(m) vs. log(v), the spatial SL slope b≃2, when long-term correlation between any pair of locations is almost zero (ρ≃0). **(d)** The SL slope plotted against average synchrony for several independent realizations (independently and randomly drawn data). **(e-g)** For identically distributed but not necessarily independent data, the effect of synchrony (Ω) on the SL for a model in different sampling locations in the multivariate time series drawn from normal distribution using the common element method [77] given that ρ=0.001, 0.01, 0.1. The scaling exponent decreases with increasing correlation ρ. **(h)** The SL slope *b* against Ω is plotted using Eq. (6) that confirms the numerical trend in the scaling exponent. Inset shows the root mean squared error (RMSE) derived from the linear regression of log(v)-vs-log(m). Error bars depict standard deviation for 50 independent realizations.

### Empirical data distribution: Effect of detrending

Fig 3a shows the empirical BOLD signals from 116 brain regions of one young adult during resting state, where non-zero temporal means and low-frequency trends are prominently visible in the raw data, as illustrated for one exemplary brain region (left cerebellum-10) Fig 3b. Fig 3c and 3d present a histogram and normal probability plot of the raw data. The data fit neither the normal line (black dashed line) nor the location-scale curve (red).

**Fig 3:**
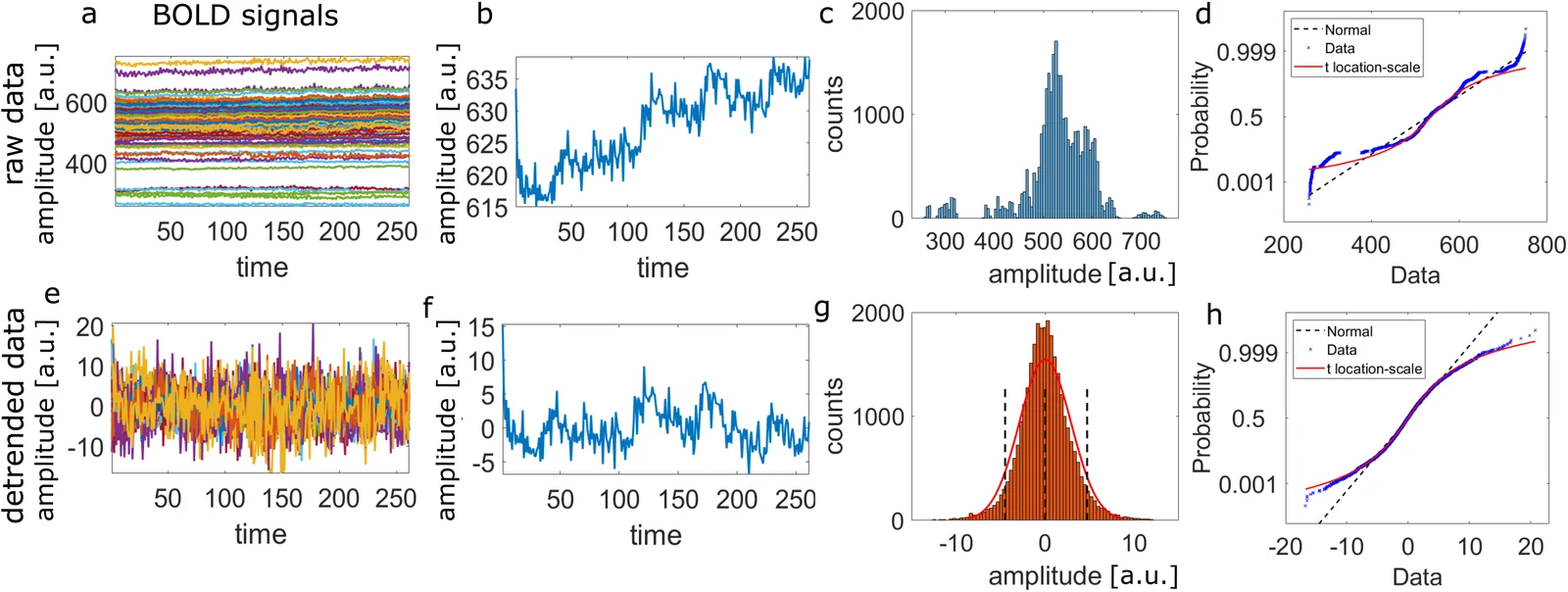
Empirical data distributions: Before and after detrending operation. **(a)** Empirical BOLD series in the resting state of one participant. Data size is 261×116; brain regions = 116 (AAL atlas), time points = 261. Amplitude is in arbitrary units [a.u.], while time is given in units of TR (repetition time in seconds). **(b)** BOLD signal of left cerebellum-10. **(c)** Histogram plot of the raw data. **(d)** The normal probability plot shows that neither the normal line (black dashed line, the reference line corresponding to a perfect normal distribution fit) nor the location-scale curve (red) fits very well. **(e)** After detrending, the BOLD series of all the brain regions is shown to spread across zero. **(f)** Suppressed trend of the BOLD signal of left cerebellum-10, compare **(b)**. **(g)** Data distribution with normal fit of the blue curve in **(f)**. The vertical black dashed lines indicate 95% CI. **(h)** Normal probability plot of the detrended data shows that it fits well to the normal distribution for the data range within 95% CI.

Fig 3e illustrates that following a detrending operation, BOLD signals across all brain regions oscillate around a zero temporal mean with both positive and negative fluctuations. The detrending removes trends and temporal mean, as illustrated in Fig 3f for one brain region. The detrended spatiotemporal BOLD data tend to follow a normal distribution, as confirmed by the plots in Fig 3g and 3h for one exemplary brain region. The histogram and normal probability plots for the detrended data further confirm the normal distribution fit within a 95% confidence interval (CI). We perform the detrending operation on 116 brain regions of all the participants (*N* = 840) using MATLAB and Python built-in functions. Results for the same subject during two naturalistic tasks are shown in Fig O and Fig P in S1 Appendix. Also, subject-wise spatial SL exponents are computed and plotted for three brain states. See Fig Q and Fig R in S1 Appendix for log-normal plots of spatial RMS and spatial variance, quantile-quantile plots, and spatial RMS-versus-variance scatter plots.

### Task-driven coordinated dynamics and spatial scaling law

We examine how degree of brain-wide coordinated dynamics, quantified by average synchrony Ω as the temporal variability of the spatial mean activity of 116 brain regions, modulate the spatial scaling law, during both resting state and naturalistic tasks using fMRI data of *N* = 645 participants spanning 18–88 years from the CamCAN dataset [78,79]. The BOLD time series has size of 261 time points × 116 brain regions [78,79].

The SL slope is plotted against Ω for resting state (rest), sensorimotor task (smt), and movie watching (mov) task in Fig 4, respectively, arranged in order from top to third row. We have considered two methods to derive the slopes, *b*, using Eq. (6), and linear regression fitting (shown in Fig S in S1 Appendix).

**Fig 4:**
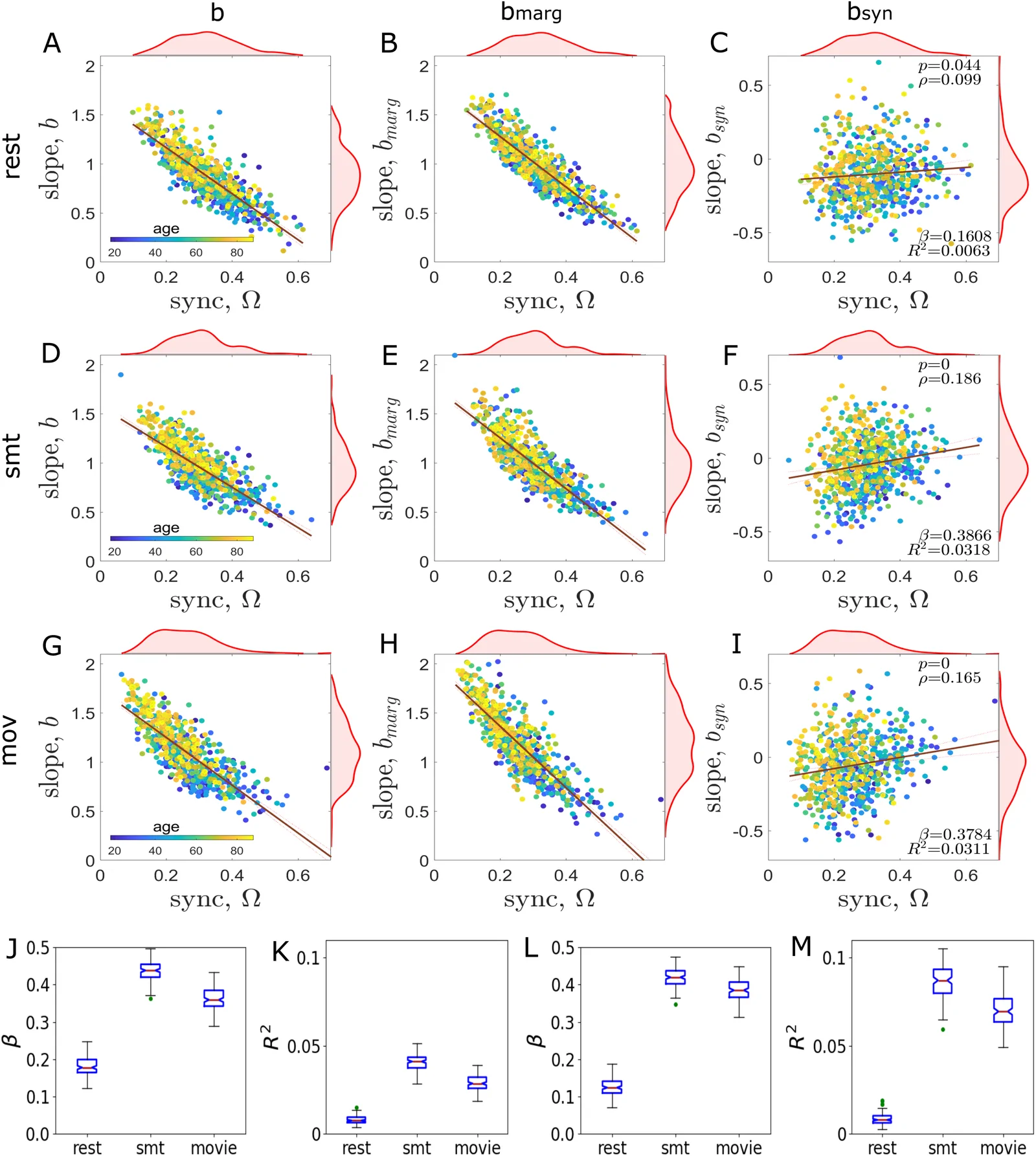
Spatial SL exponent against synchrony for different brain states. (A-C) resting state, (D-F) sensorimotor task, and (G-I) movie watch. (A,D,G) Scatter plots (colored circles) and linear regression fit (red lines) between *b* and Ω in three brain states. (B,E,H) Contributions of marginal distributions, slope $b_{marg}$ against Ω. (C,F,I) Synchrony-driven SL slope ($b_{syn}$) after removing marginal effects. Statistical significance is considered when *p* < 0.005. **(J,L)** The association between $b_{syn}$ and Ω is captured by β, together with goodness of fit *R*^2^ in (K,M) for 100 realizations. Results are shown for semi-analytical (J,K) and fitting-based analysis **(L,M)**.

To assess the overall association between the scaling exponent and average synchrony prior to removing marginal contributions, we first examine the raw correlation between *b* and Ω across participants in all three brain states, shown in Fig 4A, 4D, and 4G. Note that this raw correlation reflects combined contributions from both brain-wide synchrony and local marginal signal statistics, necessitating the decomposition described below. The relation between the scaling exponent and average synchrony is significantly negative in all three brain states. Higher synchrony Ω is associated with lower slope *b* across all three brain states in the raw correlation, where contributions from marginal signal distributions have not yet been removed. A higher association between *b* and Ω may be attributed to contributions from marginal distribution.

To account for the actual influence of synchrony, we remove the effect of marginal distributions that may have influenced the slope in conjunction with the changes due to synchrony. We decompose the scaling exponents, $b=b_{marg}+b_{syn}$, where $b_{marg}$ accounts for the contributions from time series marginals and $b_{syn}$ due to synchrony. The scaling exponent $b_{marg}$ is approximated from independently randomizing phases [80] of the empirical BOLD signals (refer to Section *Surrogate time series* in S1 Appendix), and recomputing the log(v) vs. log(m) slope, which ensures that marginals do not contribute to the slope, and thereby to extract the influence of synchrony. Fig 4B, 4E, and 4H show correlation between the spatial SL ($b_{marg}$) vs. average synchrony (Ω) in three brain states. Next, we derive $b_{syn}$=$b-b_{marg}$ to estimate the synchrony-induced SL exponent ($b_{syn}$), which is only observed after masking the effects from the time series marginals and shown in Fig 4C, 4F, and 4I. Changes in three different brain states delineate distinct patterns of association between the scaling exponent ($b_{syn}$) and Ω. At rest, we find no significant correlation between the two metrics (Fig 4C; *p* > 0.005). Whereas in sensorimotor (Fig 4F; *p* < 0.005) and movie-watching tasks (Fig 4I; *p* < 0.005), the ordinary least-squares regression of $b_{syn}$ on Ω shows a significant positive slope, i.e., the SL exponent $b_{syn}$ increases with increasing strength of synchrony. The Spearman correlation coefficient (ρ), and p-value (*p*), association slope (β), and adjusted *R*^2^ values are denoted in Fig 4C, 4F, and 4I. The slope β is estimated by ordinary least-squares linear regression of $b_{syn}$ on Ω (i.e., with $b_{syn}$ as the dependent variable and Ω as the independent variable). The parameter β in three brain states is verified with 100 independent realizations. The task-dependent modulation of β, lowest at rest and significantly higher during sensorimotor and movie-watching tasks, is consistently reproduced by both semi-analytical and fitting-based approaches (see Fig 4J and 4L), with comparable goodness of fit *R*^2^, validating the robustness of the synchrony-scaling relationship across estimation methods; see Fig 4K and 4M.

### Reduced synchrony-scaling association in aging brain

We have separated the CamCAN cohort into three age groups: (i) young adults (YA: 18–36 years, *n* = 138), (ii) middle-aged adults (MA: 46–60 years, *n* = 155), and (iii) older adults (OA: 72–88 years, *n* = 146). The three age groups were defined to maintain a year gap of around 10 years between any two groups while trying to keep almost similar number of subjects across the three groups, consistent with prior lifespan neuroimaging studies [71,72]. The correlations between synchrony and the spatial scaling exponent are presented for three age groups in three columns, shown in Fig 5A, 5D, and 5G for YA, Fig 5B, 5E, and 5H for MA, and Fig 5C, 5F, and 5I for OA. Across all three brain states, the synchrony-scaling association slope β progressively attenuates from young to older adults, indicating age-related deterioration in coordination-variability coupling. The association between the exponent $b_{syn}$ and average synchrony Ω remains significantly positive in all three mental states in YA; see Fig 5A, 5D, and 5G. In MA group, β values significantly decrease in advanced age compared to YA, as seen in Fig 5B, 5E, and 5H, which further decreases in OA group; see Fig 5C, 5F, and 5I. The Spearman correlation (ρ) and p-value (*p*), association slopes β, and adjusted-$R^{2}$ derived from a linear fit between average synchrony Ω and $b_{syn}$ are mentioned in the figures. Strength of the correlation between Ω and $b_{syn}$, measured by the associated slope β, has a tendency to rotate clockwise with increasing age in all three states.

**Fig 5:**
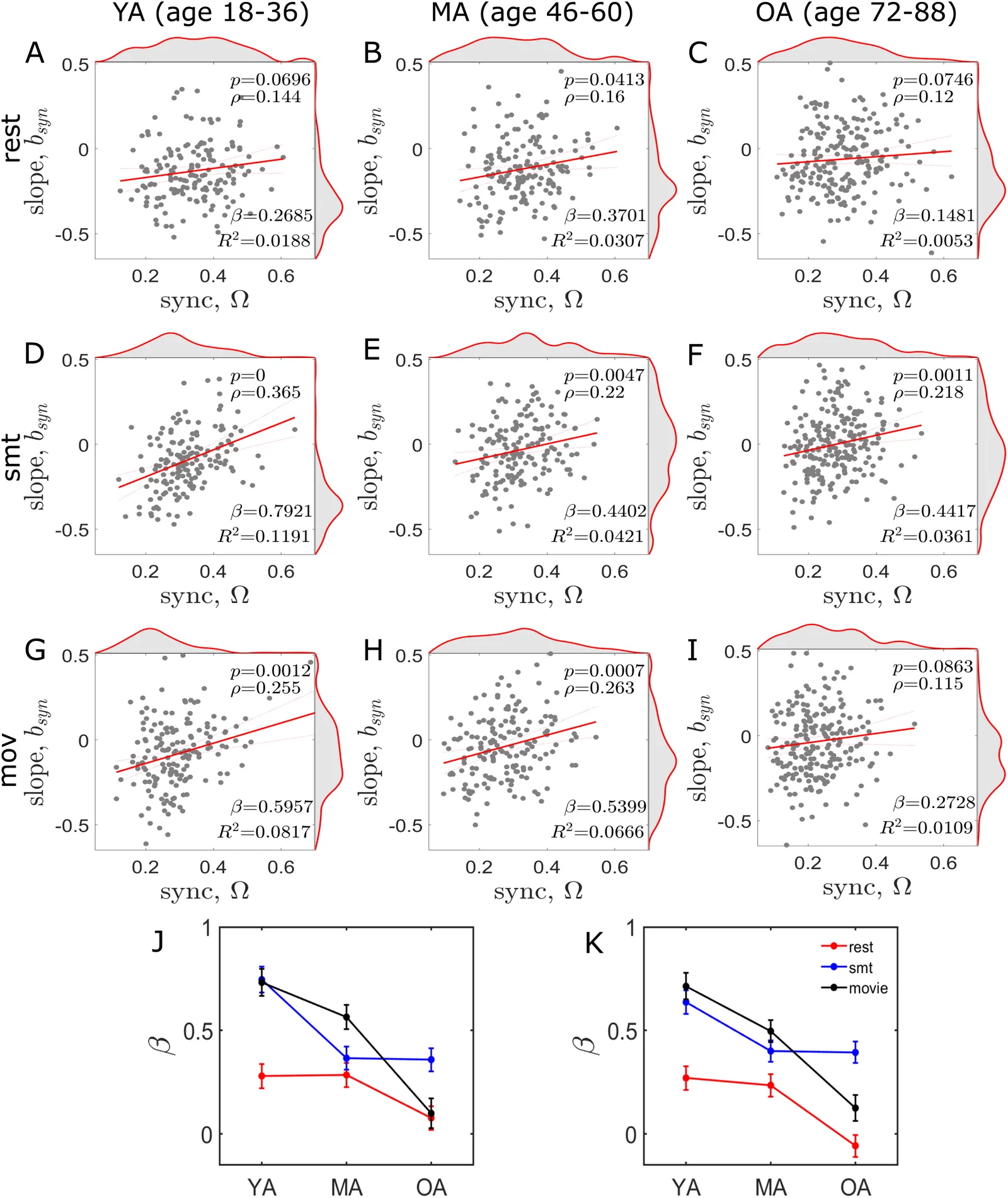
Group-wise age-related alterations in the spatial exponent. Associations between the spatial SL ($b_{syn}$) and average synchrony (Ω) are shown for three age groups in rest **(A-C)**, smt (D-F) and mov (G-I) states. (A,D,G) In young adults (YA), the slope (β) remains positive and significant in all three conditions. In middle-aged adults (shown in (B,E,H)), β rotates in clock-wise direction compared to young adults, and (C,F,I) rotates further in clock-wise direction in older adults. The slope β shows a tendency to rotate clockwise with increasing age in all three brain states. **(J,K)** Association between Ω and $b_{syn}$ is monitored by the slope β for three age groups in three brain states, where left and right plots are obtained from the semi-analytically derived and fitting-based SL slopes, respectively. Consistency in β is checked by 1000 independent realizations, i.e., randomization of phases of the BOLD signals. The error bars indicate variations over the trials. p<0.005 implies statistical significance.

Furthermore, we present the results for 1000 independent realizations by randomizing BOLD phases of individual subjects to check consistency in the association (measured by the slope β) between average synchrony (Ω) and SL slope ($b_{syn}$) for the three age groups in Fig 5J and 5K, where left (J) and right (K) figures are plotted using semi-analytical approach in Eq. (6) and a numerical fitting-based SL exponent, respectively. The synchrony-scaling association β shows distinct age-related trajectories across brain states: during movie-watching, β declines progressively across all three age groups, during resting state β remains comparable between young and middle-aged adults but declines in older adults, while the sensorimotor task shows an early attenuation of β from young to middle-aged adults that plateaus in older adults. For fitting-based results, see Fig U in S1 Appendix. We have conducted Breusch-Pagan test to examine error variance of the least regression linear fittings, showing fitted value-versus-residuals plots across the three mental states and age groups. The lower values of the test statistics suggest that there is no statistically significant evidence of heteroscedasticity.

### Synchrony-scaling association in functional subnetworks

While brain-wide synchrony-scaling associations capture global coordination dynamics, the human brain is organized into functionally specialized subnetworks with distinct connectivity architectures and task-dependent activation profiles [67,68]. The whole-brain synchrony metric necessarily aggregates contributions from all inter-regional pairs, potentially obscuring heterogeneity in coordination regimes operating simultaneously across hierarchical functional systems. For example, default mode network (DMN) mediates internally directed cognition predominantly engaged during resting state [81], fronto-parietal network (FPN) underlies executive control with sustained baseline activity across brain states [82], sensorimotor network (SMN) governs sensorimotor integration selectively recruited during the sensorimotor task [83], visual network (VN) is driven by external visual stimulation particularly during naturalistic movie watching [84], while limbic and subcortical regions serve as integrative hubs for effective and mnemonic functions across all brain states [85,86], and the cerebellum contributes to motor and cognitive coordination [87]. Critically, age-related neurodegeneration affects these networks unevenly, with subcortical and limbic structures among the earliest to exhibit white matter degradation and metabolic decline [43,88]. Decomposing the synchrony-scaling relationship at the subnetwork level across all three brain states therefore serves a dual purpose: validating whether the global age-related attenuation of β reflects selective disruptions within specific functional circuits, and identifying which subnetworks and brain states contribute most strongly to brain-wide coordination-variability coupling across the lifespan.

To delineate the contributions of the subnetworks in age associated changes on spatial SL against synchrony in empirical data, we separate seven functional subnetworks [89] for the AAL atlas [90], such as: DMN, FPN, SMN, VN, limbic, subcortical, and cerebellum regions. The DMN includes 12 regions of interest (ROIs): middle orbitofrontal (ORBmid L/R), inferior orbitofrontal (ORBinf L/R), anterior cingulate (ACG L/R), posterior cingulate (PCG L/R), angular gyrus (ANG L/R), precuneus (PCUN L/R) [89,91]. FPN includes middle frontal (MFG L/R), frontal inferior triangularis (IFGtriang L/R), superior parietal (SPG L/R), inferior parietal (IPL L/R), supramarginal (SPG L/R), and angular gyrus (ANG L/R) [89,92]. SMN includes precentral (PreCG L/R), rolandic operculum (ROL L/R), supplementary motor area (SMA L/R), superior temporal (STG L/R), and postcentral gyrus (PoCG L/R) [89,93]. VN includes calcarine (CAL L/R), cuneus (CUN L/R), lingual (LING L/R), middle occipital (MOG L/R), inferior occipital (IOG L/R), superior occipital (SOG L/R), fusiform (FFG L/R) [94]. Limbic regions include anterior cingulum (ACG L/R), posterior cingulum (PCG L/R), dorosal cingulum (DCG L/R), hippocampus (HIP L/R), parahippocampal (PHG L/R), superior temporal pole(TPsup), and mid temporal pole (TPmid L/R) [90]. Subcortical regions include amygdala (AMYG L/R), caudate (CAU L/R), putamen (PUT L/R), pallidum (PAL L/R), thalamus (THA L/R)) [90]. The subnetworks are shown in the first and second rows in Fig 6.

**Fig 6:**
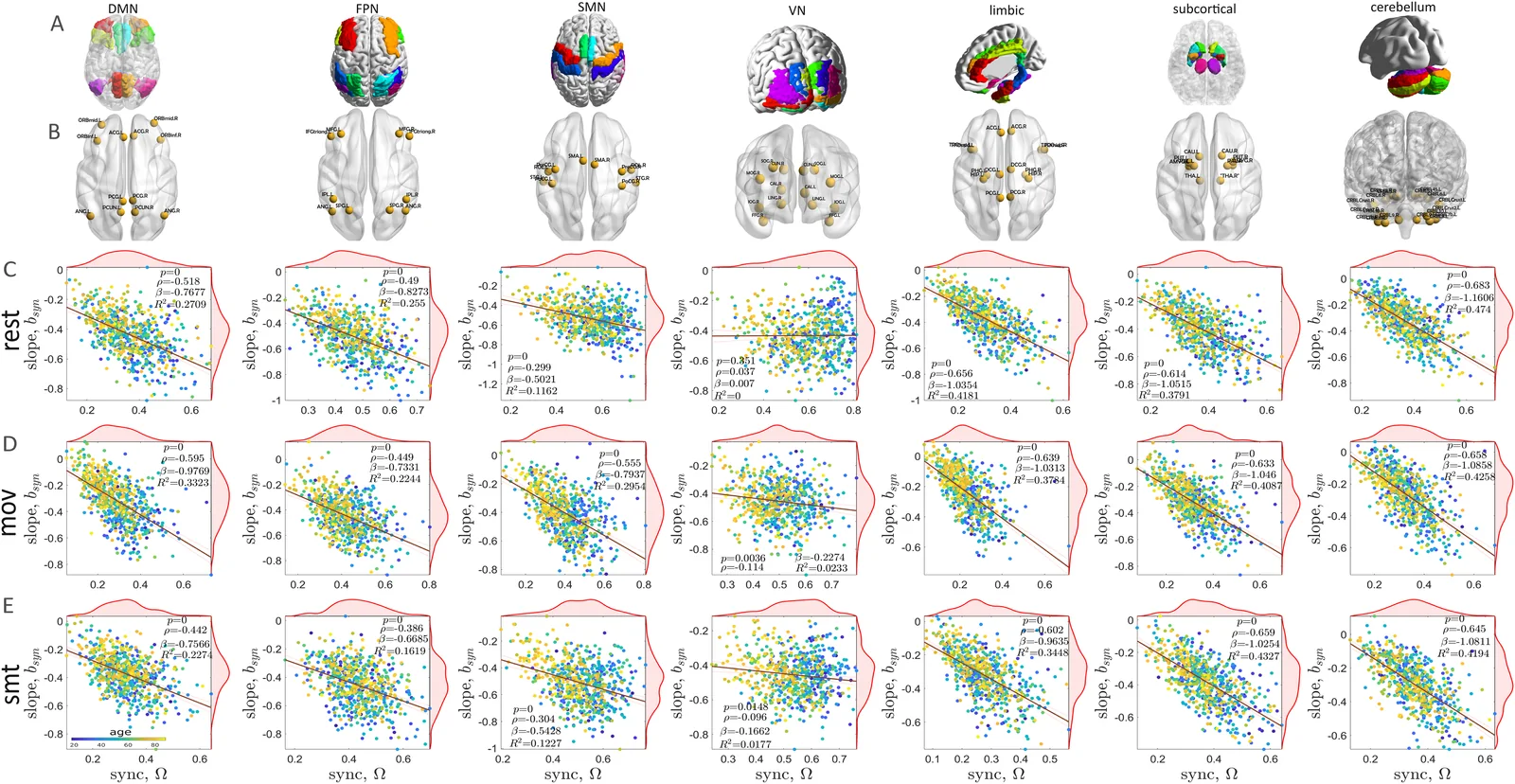
Spatial SL slope against synchrony is shown for seven subnetworks for the three brain states. **(A, B)** Visualization of the seven subnetworks in the brain, including cortical and subcortical regions involved in neurodevelopment and aging. Subnetworks are defined based on the AAL atlas. **(C-E)** The scatterplot (colors represent age) of spatial SL slope, $b_{syn}$, against synchrony, Ω, during resting state, movie watching, and sensorimotor task are shown.

The synchrony-scaling association is most prominent in limbic, subcortical, and cerebellar regions in the three brain states, compared to the remaining subnetworks. The visual network shows a significant synchrony-scaling correlation specifically during movie watching, consistent with its established role in stimulus-driven visual processing [84]. DMN and SMN show stronger synchrony-scaling associations during movie watching compared to resting state and sensorimotor task, whereas FPN exhibits comparatively stronger associations at rest. Overall, limbic, subcortical, and cerebellar regions consistently exhibit the strongest synchrony-scaling coupling across all the mental states.

### Cross-validation, clinical relevance and comparison with other metrics

We test generalization across independent datasets and clinical populations using two independent cohorts: NKI/Rockland and UCLA datasets. Cross-validation in the NKI/Rockland dataset (*N* = 89) replicates the negative relationship between synchrony and spatial scaling exponents with aging (Fig V in S1 Appendix). Comparing young adults (ages 18–30) versus older adults (ages 59–83), the association slope (β) between synchrony and scaling exponents shows a significant clockwise rotation with age, consistent with CamCAN findings. Clinical validation in the UCLA-CNP dataset (*N* = 106) reveals that individuals with ADHD (*N* = 40) compared to healthy controls (*N* = 66) shows a non-significant synchrony-induced spatial scaling relationship (p>0.05; Fig W in S1 Appendix). For detailed data descriptions see Table B in S1 Appendix. The association slope β is profoundly reduced in ADHD, confirmed across 1000 independent realizations, suggesting that neural disorders disrupt the coupling between brain-wide coordinated activity and fluctuation scaling patterns observed in healthy adults. These validations demonstrate that the synchrony-scaling relationship is robust across independent healthy aging cohorts and sensitive to neuropsychiatric conditions, supporting its utility as a biomarker of brain dynamics.

Finally, a comparison with the traditional metrics for brain-wide dynamics shows consistent patterns. Metastability [95,96], entropy of functional connectivity dynamics [97,98], and phase synchrony using Kuramoto order parameter [99] all show minimal age-related changes in resting state but significant alterations during movie-watching and sensorimotor tasks (Fig X in S1 Appendix). These findings agree with the synchrony-induced spatial SL results (see Fig T in S1 Appendix), confirming that aging effects are stronger in task-evoked than resting-state activity across multiple measures of brain dynamics.

## Discussion

The relationship between synchrony and TL has demonstrated a significant applied importance in ecological populations, where climate-driven changes in spatial synchrony systematically altered the TL exponent, linking large-scale environmental forcing to population-level fluctuation structure [51, 53, 100]. If synchrony governs the scaling of variance in spatially distributed biological populations, an analogous mechanism was expected to operate in any complex systems whose components are coupled across space. The present study demonstrated precisely this in human brain function. Spatial synchrony inversely modulated the generalized scaling exponent *b* in fMRI BOLD signals across the adult lifespan. A shared scaling exponent *b* across brain regions reflects a common inter-regional coordination mechanism, while divergence in *b* across subnetworks indicates structurally distinct coordination regimes operating simultaneously within the distributed cortical network. The significance of the proposed framework is multi-fold.

*Theoretical extension:* Classical TL is developed exclusively for non-negative data that is a fundamental barrier for neuroscience applications since neural signals span both positive and negative values after the detrending operation. We circumvent the main barrier by replacing the sample mean with the spatial root-mean-square (RMS), in the definition of the scaling law, thereby obtain a generalized spatial TL now applicable beyond non-negative measurements across various disciplines. The detrending operation was necessary to remove any temporal trends and means in any measurement data, especially in neural signals, while preserving the spectral and correlation structure of the data having positive and negative values. This reformulation helps establishing a principled analytical link between the spatial scaling exponent and synchrony. In this framework, *b* operates near 2 when the fluctuations is solely driven by random processes in absence of any biophysical mechanisms [69,101–104], irrespective of the original distribution of measurements (Poisson, negative binomial, uniform, gamma); after the detrending operation the data follow a normal distribution as available for fluctuation analysis across all marginal distributions. The exponent *b* is progressively suppressed as synchrony increases, marking the transition from a spatially uncorrelated state toward collective coordination in the brain. This is directly analogous to Taylor’s original interpretation of *b* as an index of aggregation [1], now extended to signed neural signals; a lower *b* signals stronger brain-wide coordination, while *b* near 2 reflects near-independent regional dynamics.

*Disentangling random processes and coordinated dynamics:* The theoretical extension allowed us to disentangling coordinated dynamics and random processes in empirical data analysis. The framework operates at two layers of disentanglement. First, the detrending operation explicitly removes temporal means and trends, isolating intrinsic fluctuation structure from stimulus-evoked components while preserving spectral and correlation properties. Secondly, in line with the reasoning of [2], the modified synchrony-scaling association offers a straightforward approach to decompose *b* into components attributable to random processes and stimulus-driven coordinated dynamics. Decomposing *b* into a marginal component ($b_{marg}$) and a synchrony-driven component ($b_{syn}$) added a further layer of biological clarity. Particularly, the scaling exponent is decomposed as $b=b_{marg}+b_{syn}$ using phase randomized surrogate time series, explicitly separating the contributions due to time series marginal distributions ($b_{marg}$) from genuine synchrony-driven component ($b_{syn}$). Synchrony profoundly influenced the revised scaling law essentially in all our analytical, numerical, empirical and randomization-based examples. Specifically, under near-zero synchrony (Ω→0), the spatial scaling exponent approaches b≃2, confirmed by our analytical via Eq. 6 and numerical examples with 645 independent realizations, establishing this as the null baseline of purely stochastic behavior. Synchrony-induced deviations from this baseline therefore unambiguously reflect genuine coordinated dynamics rather than artifacts of the underlying signal distribution or external drive.

*Neurobiological interpretation of synchrony-driven scaling law:* The marginal component $b_{marg}$ that captures region-wise signal statistics is largely driven by spatially uncorrelated, non-neuronal fMRI noise sources such as scanner thermal noise, cardiac and respiratory fluctuations, vasomotion, scanner drifts, and changes in blood pressure and cerebral autoregulation [105–107], independent of any brain-wide coordinated activity. Quantitatively, thermal noise alone accounts for ~35% of signal fluctuations in gray matter voxels, followed by low-frequency scanner drifts (~35%) and spontaneous BOLD activity (~18%) [106]. Together, these region-specific contributions shape each brain region’s marginal signal distribution independently of any inter-regional coordination. By contrast, $b_{syn}$, isolated via phase randomisation surrogates that destroy inter-regional correlations while preserving local signal statistics, captures exclusively the collective coordination fingerprint of the brain.

*Characterizing resting-state and task-evoked activity:* The contrast between rest and task points to distinct organizing principles. The observation that $b_{syn}$ shows no significant correlation with synchrony at rest (*p* > 0.005) but significant positive correlations during sensorimotor (β = 0.3866, *R*^2^ = 0.0318, *p* < 0.005) and movie-watching tasks (β=0.3784, *R*^2^ = 0.0311, *p* < 0.005) directly confirms that the framework successfully separates intrinsic from task-evoked coordination [cf. Fig 4C, 4F, and 4I]. Resting-state dynamics primarily reflect intrinsic functional network architectures that are largely invariant to coordination strength, whereas task-evoked dynamics recruit flexible cortical coordination patterns that substantially reshape scaling relationships. Age-related effects predominantly impact task-evoked dynamics, suggesting that healthy aging preserves intrinsic brain-wide functional organization in spontaneous activity at rest, while reorganizing the adaptive coordination capacities required to meet external cognitive demands in task. This task-dependent modulation in synchrony-scaling association indicates that externally driven or goal-directed brain states recruit coordinated activity patterns that fundamentally alter fluctuation scaling properties, a signature absent during unconstrained spontaneous activity.

*Lifespan sensitivity with a unified compact metric:* Applied to the CamCAN cohort (*N* = 645, ages 18–88 years), the synchrony-scaling association slope β reveals a progressive clockwise rotation across three age groups — young adults, middle-aged adults, and older adults — in all the three brain states. This age-related attenuation is strongest during sensorimotor tasks (β declining from 0.7921 in YA to 0.4417 in OA), moderate during movie watching (β from 0.5957 in YA to 0.2728 in OA), and weakest at rest, a pattern replicated independently in the NKI/Rockland cohort (*N* = 89). This sensitivity to both task condition and age is not achievable by conventional single-region temporal scaling or autocorrelation approaches, which lack the multivariate spatial sensitivity of the present framework. The pattern suggests that aging compromises the brain’s capacity to modulate spatial scaling through coordinated activity during cognitively demanding or stimulus-driven states, while preserving the intrinsic scaling properties of spontaneous dynamics, which declines with advance age.

*Subnetwork specificity:* The synchrony–scaling association is not uniformly distributed across the brain. Limbic, subcortical, and cerebellar regions exhibit the enhanced synchrony–scaling associations across all three brain states, while the visual network shows robust associations specifically during movie watching, and frontoparietal networks show comparatively stronger effects at rest. This network selectivity demonstrates that the framework captures biophysically meaningful spatial heterogeneity in coordinated dynamics, rather than a global artifact.

Clinical relevance: In the UCLA-CNP dataset (*N* = 106), individuals with ADHD (*N* = 40) show a profoundly reduced synchrony-scaling association slope β compared to healthy controls (*N* = 66), confirmed across 1000 independent realizations. This disruption in synchrony–scaling coupling suggests that the metric is sensitive to neuropsychiatric conditions in a way that goes beyond conventional functional connectivity measures, which do not capture how coordination strength shapes scale-free variability patterns. This demonstrates that the framework can be extended to disorders characterized by atypical large-scale synchrony, including autism spectrum disorder, epilepsy, schizophrenia, and neurodegenerative diseases [73,108,109], enabling quantification of how coordination deficits reshape fluctuation scaling properties of brain-wide neural activity.

### Connection to existing neuroscience literature

During active and passive tasks, the age-related reduction in the synchrony–scaling association slope β reflects a weakening of the brain’s capacity to regulate its spatial scaling properties through coordinated activity. This attenuation is evident across both task conditions; in the sensorimotor task, β decreases from 0.7921 in young adults to 0.4417 in older adults, and in movie-watching, from 0.5957 to 0.2728. This suggests that in older adults, increases in brain-wide synchrony no longer produce the same degree of reorganization in spatial fluctuation patterns as observed in younger adults, pointing to a fundamental deterioration in the coupling between large-scale network coordination and the brain’s scale-free dynamics. This is consistent with Hinault et al. (2020) [88], who showed that disrupted neural synchrony mediates the relationship between white matter integrity and cognitive performance in older adults. Structurally, the degradation of long-range white matter connections in advanced age would directly reduce the capacity for spatially distributed regions to achieve temporally aligned coordination, which our framework captures as a reduction in β. Complementarily, Weistuch et al. (2021) [43] demonstrated that metabolic changes modulate network synchrony in the aging brain, offering a further mechanistic account: as metabolic efficiency declines with age, the energetic resources needed to sustain coordinated large-scale brain activity become limited, manifesting in our framework as a diminished synchrony-driven scaling signature.

In contrast, during resting-state, the absence of a significant correlation between $b_{syn}$ and average synchrony suggests that the intrinsic network architecture governing spontaneous hemodynamic fluctuations remains relatively intact across the adult lifespan. What deteriorates with age is specifically the brain’s adaptive coordination capacity, reflected in its ability to flexibly recruit and sustain large-scale synchronized activity in response to cognitive or sensory demands. This dissociation between preserved resting-state scaling and degraded task-evoked scaling aligns with prior works [72,110]. D’Esposito et al. (2003) [110] reported that aging effects on BOLD signals are stronger under cognitive demand than during spontaneous activity. Complementarily, Pathak et al. (2022) [72] demonstrated compensatory preservation of neural synchrony across the adult lifespan. Our framework extends these findings by revealing that such compensation operates primarily at the level of intrinsic resting dynamics, while task-evoked coordination mechanisms undergo selective deterioration with age.

The heightened synchrony-scaling associations in limbic, subcortical, and cerebellar regions reflect their roles as integrative hubs whose coordinated activity shapes brain-wide fluctuation patterns [85–87]. The moderate associations observed in the DMN, particularly during movie watching, align with its established role in internally directed cognition and mental simulation [81]. The frontoparietal network’s comparatively higher resting-state association is consistent with its established role in domain-general executive control, maintaining a degree of coordinated baseline activity even during unconstrained cognition [82]. The visual network shows a comparatively higher association during movie watching than during the other two brain states, consistent with its established role in stimulus-driven visual processing during naturalistic stimuli [84]. Comparatively weaker associations in sensorimotor network are consistent with its more spatially constrained and functionally localized architecture [83].

In ADHD, the coupling between large-scale coordinated brain activity and spatial fluctuation scaling is absent, in contrast to healthy adults, with profoundly reduced β. It reveals a fundamental decoupling between network-level coordination and the scale-free organization of brain-wide neural dynamics, aligns with Mathalon and Sohal (2015) [108] on neural oscillations and synchrony in brain dysfunction, and King et al. (2018) [73] on temporal synchrony differences in individuals with autism. This suggests that the disrupted synchrony–scaling coupling may be a transdiagnostic signature of conditions characterized by atypical large-scale coordination, including autism spectrum disorder, epilepsy, and schizophrenia.

The proposed framework differs from existing approaches in three important ways. First, Tomasi et al. [10] applied a temporal Taylor’s law to the amplitude of low-frequency fluctuations (ALFF) and local functional connectivity density, showing that scaling exponents decrease with age, but this approach operates on temporal mean-variance relationships within individual regions and does not capture how brain-wide spatial coordination drives fluctuation scaling simultaneously across regions. Second, He et al. [9] and He [111] characterized scale-free properties of BOLD signals, showing that the power-law exponent differentiates brain networks and is modulated between rest and task, but these approaches capture temporal scaling within individual regions and cannot isolate inter-regional coordination from local signal statistics, a separation achieved by the present framework through decomposition of the scaling exponent into marginal and synchrony-driven components. Third, Ciuciu et al. [112] characterized multifractal BOLD dynamics using power-law spectral exponents within individual regions, whereas the present framework provides a compact metric derived by explicitly linking average spatial synchrony to the spatial scaling exponent instantaneously across all brain regions. Furthermore, unlike the Kuramoto order parameter [99], derived from phase coherence, and metastability [95], which captures fluctuations around a mean synchrony state, the synchrony-scaling association slope provides a direct mechanistic link between brain-wide coordination and scale-free spatial variability using detrended BOLD amplitudes, offering a more parsimonious characterization of age-related reorganization than entropy of dynamic functional connectivity [97,98].

### Robustness checks

The fMRI data acquisition parameters (TR, TE, voxel size) do not influence the synchrony-scaling association. The detrending operation explicitly removes low-frequency scanner drifts, temporal means, and slow trends, which are the primary channels through which TR-dependent sampling artifacts and TE-dependent differences could otherwise influence the signal [58]. The scaling exponent *b* derived as a log-log slope between spatial RMS and variance each normalized by *n* locations, is a dimensionless quantity independent of total recording duration, sampling rate, time window size, and number of spatial locations [36], since multiplicative TE-induced amplitude shifts cancel as additive constants in log space and do not affect the slope [111,112]. Similarly, Ω, computed as a normalized average of pairwise correlations normalized by *n*^2^, is robust to network size differences. Voxel count does not affect our analysis, as the BOLD signal for each brain region is spatially averaged during preprocessing into a single representative time series, contributing exactly one time series per region to the T×n data matrix regardless of the number of voxels it contains [90].

We made multiple layers of robustness checks of our findings. First, the theoretical extension of the framework to multivariate data spanning negative and positive values is validated numerically across Poisson, negative binomial, uniform, and gamma distributions following detrending (Fig B–Fig M in S1 Appendix), demonstrating that the revised scaling law is a property of the coordination structure itself, independent of the underlying signal distribution. Crucially, this establishes b≈2 under near-zero synchrony as the null baseline of purely stochastic behavior, against which synchrony-driven deviations in empirical analyses reflect genuine coordinated dynamics rather than artifacts of marginal signal statistics. Second, the synchrony–scaling relationship is validated using two independent methods, an analytical approach based on Eq. 6 and a fitting-based linear regression approach, producing consistent results across all brain states and age groups. Third, cross-cohort validation is demonstrated across two independent healthy aging cohorts. The age-related decline in β observed in CamCAN is independently replicated in the NKI/Rockland dataset [113], confirming that the progressive deterioration of synchrony-driven scaling with age is a generalizable biological phenomenon rather than a dataset-specific artifact. This is further supported by [114], who demonstrated that lifespan BOLD variability trajectories replicated across two datasets acquired with substantially different TRs, confirming that TR differences do not drive age-related effects in BOLD signal measures. Fourth, biological consistency is verified by randomizing the phases of empirical BOLD signals across independent realizations for both lifespan analyses and the clinical ADHD comparison, with error bars reflecting the stability of the synchrony-scaling association across repeated randomization. Fifth, cross-metric consistency with established neurobiological measures such as metastability, entropy of dynamic functional connectivity [98], and the Kuramoto order parameter [99], reveals consistent patterns of minimal age-related changes at rest but significant alterations during movie-watching and sensorimotor tasks (Fig X in S1 Appendix), confirming that the synchrony-induced spatial scaling results reflect genuine age-related reorganization of brain dynamics.

Beyond neuroscience, the proposed framework naturally can be extended to any complex system having negative and positive spans of values to examine scale-invariant spatial organization. The generalized RMS–variance formulation overcomes prior limitations by mitigating the confounding effects of temporal means and trends, thereby revealing coordination-driven scaling laws that would otherwise remain obscured. By stripping away marginal structure, it reveals fundamental scaling principles governed by collective dynamics, providing a unified analytical approach for measurements across ecological, financial, climate, and engineered systems.

## Methods

### Spatial scaling law

Our general methodology is elaborated here using a realistic example of spatiotemporal data from the brain. Consider spatio-temporal time series data $y_{i}(t)$, where *i* = 1,2,...,*n*; *n* is the number of nodes or locations or brain regions, that represent the BOLD signals of four exemplary colored cortical regions (green, red, blue, and yellow) in Fig 1. The multivariate data may follow any distribution (Poisson, negative binomial, gamma, uniform, and others). After a detrending operation, the additional trends and temporal mean are removed from such multivariate spatio-temporal time series. The spatial distribution of the detrended data $Y_{i}(t)$ across the entire location then follows a normal-like distribution, having negative to positive data spreading.

Exemplary values of the variable $Y_{i}(t_{1})$ at time *t*_1_, $Y_{i}(t_{2})$ at time *t*_2_ and so on, $Y_{i}(t_{T})$ a*t* time $t_{T}$ of the detrended data shown in Fig 1 are taken from the four colored brain regions. The spa*t*ial variance *v*(*t*) and spatial root-mean-square (RMS) *m*(*t*) values are derived from spatial distributions over the entire space at each time point, e.g., *m*_1_ and *v*_1_ (where the subscript denotes the *t*ime index) are the spatial RMS and variance, respec*t*ively, derived at time point *t* = *t*_1_, as illustrated in Fig 1. Next, the exponent *b* is obtained by fitting the least-squares linear regression to the log(*m*(*t*)) sca*tt*erplo*t* against log(*v*(*t*)), which here is referred to as the spatial scaling law (SL). Please refer to Fig 1 for visual interpretation.

We assume that the detrended multivariate stochastic process represented by $Y_{i}(t)$ = (*Y*_1_(*t*), ..., $Y_{n}(t)$) is assumed to be stationary and ergodic [2,115]. The spa*t*ial SL is then formulated as a linear relation between the spatial RMS (*m*(*t*)) and spatial variance (*v*(*t*)) after logarithmic transformation at *t*ime point *t*. The spatial RMS is defined as the square root of the quadratic mean over the entire loca*t*ions (all brain regions) at time instant *t* as,


$$\begin{array}{c} m(t)=\sqrt{\frac{1}{n}\sum_{i=1}^{n}Y_{i}^{2}(t)}. \end{array}$$ (1)

Thus, the spatial RMS considers squared values of all the data points directly, indicating the effective magnitude of the signal, averaged over the space at each time point. The sample mean over the whole location at time *t* is calculated by:


$$\begin{array}{c} \mu (t)=\frac{1}{n}\sum_{i=1}^{n}Y_{i}(t), \end{array}$$ (2)

which measures the central tendency of the data at time *t*. The spatial variance at time *t* is expressed as:


$$\begin{array}{c} v(t)=\sum_{i=1}^{n}Y_{i}^{2}(t)/(n-1)-n\mu^{2}(t)/(n-1), \end{array}$$ (3)

which measures the dispersion of data points around the sample mean. Here, the spatial variance (*v*) is expressed as a function of spatial RMS (*m*) as *v*(*t*)=$n\left(m^{2}(t)-\mu^{2}(t)\right)/(n-1)$. The scaler relationship between RMS and variance is defined by, $v(t)=am^{b}(t)$, or after a logarithmic transformation, we have,


$$\begin{array}{c} log_{10}(v)=b\;log_{10}(m)+log_{10}(a). \end{array}$$ (4)

### Coordinated hemodynamics quantified by average synchrony (Ω)

The long-time correlation between measurements in any pair of locations is derived using pairwise correlation coefficients, yielding a region-vs-region correlation matrix, as shown in Fig 1. The terms *average spatial synchrony* and *average synchrony* are used interchangeably throughout the text to refer to Ω, which quantifies brain-wide coordinated hemodynamics. Defining $Z_{i}=Y_{i}/\sqrt{var(Y_{i})}$, the standard measure of average synchrony [2,52] is given by,


$$\begin{array}{c} \Omega =(1/n^{2})\sum_{i,j=1}^{n}cor(Y_{i}(t),Y_{j}(t))=(1/n^{2})\sum_{i,j=1}^{n}cov(Z_{i}(t),Z_{j}(t))=var(\mu (t)). \end{array}$$ (5)

Detailed derivations are presented in S1 Appendix. The average synchrony Ω can be 0≤Ω≤1, which is a global metric capturing overall coordinated neural responses underlying internal and external stimuli driven brain activity from the pair-wise correlation matrix. Ω is 1 when all the regions are highly correlated, could be in- or, anti-phase correlation, and Ω=0 represents complete desynchrony across all regions. The pair-wise correlation matrix is also referred to as functional connectivity (FC) in neuroscience [62–69], which varies from -1–1, where ${FC}_{ij}=1$ implies complete coherence between $i^{th}$ and $j^{th}$ brain regions; ${FC}_{ij}=-1$ implies antisynchrony, and ${FC}_{ij}=0$ depicts complete desynchrony between two brain regions. While FC can be quantified by correlation, covariance, or mutual information [62,63,116], it primarily serves as an empirical characterization of interregional spatial relationships without revealing the underlying mechanisms driving the covariations [62,64].

### Analytical formulation: Synchrony-induced spatial scaling law

The traditional way to test the scaling law is a linear fit between log(m(t)) and log(v(t)) for a finite realization of the processes. The linear regression exponent is b=cov(log(m),log(v))/var(log(m)) [2]. Applying the delta method [117], we derive,


$$\begin{array}{c} b\approx \frac{\left(n-1)E(m\right)}{n}\frac{cov(m,v)}{\left[E(m{)}^{2}+var(m)-\Omega \right]var(m)}. \end{array}$$ (6)

On the right-hand side, the first factor and the expected value E(*m*) depend on marginal distributions, $Y_{i}$, but not on the correlations cor$\left(Y_{i},Y_{j}\right)$. Please refer to the S1 Appendix for the detailed derivation of Eq. 6. This provides an idea behind our subsequent analyses: if the brain-wide coordinated activity measured by the average synchrony (Ω or var(μ)) changes, but the marginals $Y_{i}$ remain fixed, we expect the slope *b* will change. We assume that cov(*m*,*v*) and var(*m*) have minimal effect on the slope. As the average synchrony appears in the denominator of Eq. (6), the slope *b* is inversely proportional to the variations in Ω, thereby encapsulating the long-term correlations across the spatial domain. In our empirical analysis, we restrict our examination to the linear association between Ω and spatial SL exponent (*b*) captured by the association slope β, described in the section below.

In our empirical examples, we extracted the slope $b_{syn}$ attributable to coordinated dynamics by removing the contribution of marginal distribution ($b_{marg}$, driven by random processes) from the spatial SL exponent (*b*) using


$$\begin{array}{c} b_{syn}=b-b_{marg}. \end{array}$$ (7)

The slope $b_{marg}$ is derived from the surrogate time series obtained after phase randomizing the empirical BOLD signal at each location; see SI text “Section 6.3. Surrogate time series: phase-randomization.

### Synchrony-scaling association (β)

Finally, we assessed the impact of synchrony on spatial scaling through the synchrony-scaling association slope (β), obtained by regressing $b_{syn}$ on Ω, while accounting for the marginal contributions of random processes. We derive an approximate analytical relation between spatial SL and synchrony, then validate the framework using empirical fMRI data from healthy aging and developmental cohorts to demonstrate generalizability. The slope β is utilized to capture alterations in resting-state and task-evoked BOLD activity across brain regions and subnetworks along the lifespan trajectory. We have obtained the degree of impact of synchrony on the spatial SL using least-square linear regression fit between *b* and Ω. We obtained β using two methods: (i) Analytical approach: We analytically derived *b* from Eq. 6, calculated $b_{syn}$ using Eq. 7, and then obtained β [Figs 4J, 4K, and 5J]. (ii) Fitting-based approach: We estimated *b* via least-squares linear regression of $\log_{10}(m)$-versus-$\log_{10}(v)$, calculated $b_{syn}$, and then obtained β [Figs 4L, 4M, and 5K].

### Empirical datasets

We used three independent cohorts from publicly available datasets. The Cambridge Centre for Aging and Neuroscience (CamCAN) *N* = 645 healthy participants, http://www.mrc-cbu.cam.ac.uk/datasets/camcan [78, 79]. See Table A in S1 Appendix for details of fMRI acquisition parameters for CamCAN. Total *N* = 89 healthy participants from the Nathan Kline Institute (NKI)/Rockland dataset, available in the UCLA Multimodal Connectivity Database (UMCD) [113]. We have randomly selected *N* = 66 healthy controls (HC) and *N* = 40 Attention-Deficit Hyperactivity Disorder (ADHD) participants from the Consortium for Neuropsychiatric Phenomics, https://f1000research.com/articles/6-1262/v2 [118]. See Table B in S1 Appendix for details of the participants in three datasets.

### Mathematical derivation of the spatial scaling exponent

Lets assume, the amplitude of a spatio-temporal time series data at time *t* in $i^{th}$ location is modeled by a real valued random variable $Y_{i}(t)$ for i=1,...,n, where $Y(t)=(Y_{1},...,Y_{n})$ is stationary and ergodic. We are interested in the log(v)-log(m) scatter plot for a finite duration realization of these processes (ignoring the times *t*, when *m*(*t*) or *v*(*t*) was zero), and particularly, the slope and intercept obtained by regressing the y-axis quantity on the x-axis quantity of this scatter plot,


$$\begin{array}{cc} b & =\frac{\text{cov}\left(\log(m(t)),\log(v(t))\right)}{\text{var}(\log(m(t)))}, \end{array}$$ (8)

$$\begin{array}{cc} \log(a) & =\text{E}(\log(v(t)))-b\text{ E}(\log(m(t))). \end{array}$$ (9)

These are the two basic quantities in the spatial scaling law (SL). The covariance, variance, and RMS are sample quantities computed over time for the finite-duration realization. Given the ergodicity of *Y* and consistency of the spatial RMS, mean, variance and covariance, we consider the previous equations as a general form in the sequel for large samples, valid for both temporal and spatial cases. We compute the covariance, variance, RMS, and expected values for the marginal distributions of the processes *m* and *v* when these quantities are positive, and likewise we get the same results from the positive- and negative-valued data distributions. Furthermore, we assume that both the scaling parameters (*m* and *v*) are positive and finite.

Suppose the long-term temporal correlations $\rho_{ij}=cor(Y_{i},Y_{j})$ exist for all i,j=1,⋯,n. These temporal correlations between a pair of sites represent spatial synchrony between the regions *i* and *j* (again making use of ergodicity, and consistency of the sample covariance and variance). The average synchrony, $\Omega =\frac{1}{n^{2}}\sum_{i}\sum_{j}\rho_{ij}$ is an average correlation across all pairs of sampling locations (including comparisons of a location with itself, which give $\rho_{ij}=1$, and is commonly used in empirical studies [2]. We will analyze the influence of Ω on *b*. Ω is naturally interpretable as a measure of average coherence in anti- and in-phase state for the following additional reasons. First, letting, $Z_{i}=Y_{i}/\sqrt{var(Y_{i})}$, we have


$$\begin{array}{cc} \Omega & =\frac{1}{n^{2}}\sum_{i=1}^{n}\sum_{j=1}^{n}\text{cor}(Y_{i}(t),Y_{j}(t)) \end{array}$$ (10)

$$\begin{array}{cc} & =\frac{1}{n^{2}}\sum_{i=1}^{n}\sum_{j=1}^{n}\text{cor}(Z_{i}(t),Z_{j}(t)) \end{array}$$ (11)

$$\begin{array}{cc} & =\frac{1}{n^{2}}\sum_{i=1}^{n}\sum_{j=1}^{n}\text{cov}(Z_{i}(t),Z_{j}(t)) \end{array}$$ (12)

$$\begin{array}{cc} & =\frac{1}{n^{2}}\text{ cov}\left(\sum_{i=1}^{n}Z_{i}(t),\sum_{j=1}^{n}Z_{j}(t)\right) \end{array}$$ (13)

$$\begin{array}{cc} & =\text{var}\left(\frac{1}{n}\sum_{i=1}^{n}Z_{i}(t)\right) \end{array}$$ (14)

$$\begin{array}{cc} & =\frac{\text{var}\left(\frac{1}{n}\sum_{i=1}^{n}Z_{i}(t)\right)}{\frac{1}{n}\sum_{i=1}^{n}\text{var}(Z_{i}(t))} \end{array}$$ (15)

$$\begin{array}{cc} & =\text{var}\left(\mu (t)\right)\; \end{array}$$ (16)

where, equation (16) is the variance of the spatial mean μ, and is readily interpretable as the extent to which oscillations in local time series reinforce each other or cancel in the average time series. In all our examples for the detrended data, var$(Z_{i}(t))=1$.

Second, Eq. (16) and the definition of Ω show that 0≤Ω≤1. The value 0 occurs when the variability in the $Z_{i}$ exactly cancels, i.e., var($\frac{1}{n}\sum_{i}Z_{i}$)=0 and perfect asynchrony. The value 1 occurs for perfect synchrony, when $Z_{i}=Z_{j}$ for all *i*,*j*. An alternative measure for average synchrony is $\left|\frac{1}{n(n-1)}\sum_{i\text{≠}j}\rho_{ij}\right|$. The correlation coefficient ρ can take negative values. In case of perfect anti-synchrony and synchrony, the Ω can be 1, except, for perfect asynchrony.

To understand the relationship between the average synchrony and the spatial scaling law for a large sample under the assumptions of stationarity and ergodicity, it is sufficient to work with the marginal distribution *Y*, which follow a normal distribution in our case, instead of with the process *Y*(*t*). We have considered families of distribution (e.g., Poisson, negative binomial, uniform and gamma distributions) of the form $Y=(Y_{1},\cdots ,Y_{n})$ such that for each *i*, all members of the family have the same marginals $Y_{i}$. We also consider how Ω and *b* covary through the family before and after the detrending operation. After detrending operation, the underlying distribution of the marginal started following Gaussian normal distribution. In the theoretical development, marginals are kept constant to understand the influence of synchrony Ω in isolation from other potential influences on *b*. To describe the intuition of the mathematical analyses that will be described in details in the next section, we need a lemma, and taken from [2,3,117,119].

**Lemma 1.** Let *x* be a real valued random variable with finite mean E(*x*) and variance var(*x*). If a real-valued function *f* of real *x* is twice differentiable at E(*x*), then the *delta method* [117] gives


$$\begin{array}{cc} & f(x)\approx f(\text{E}(x))+(X-\text{E}(x))f^{\prime }(\text{E}(x)) \end{array}$$ (17)

$$\begin{array}{cc} & \text{E}(f(x))\approx f(\text{E}(x))+\frac{1}{2}f^{\prime \prime }(\text{E}(x))\text{ var}(x) \end{array}$$ (18)

$$\begin{array}{cc} & \text{var}(f(x))\approx [f^{\prime }(\text{E}(x)){]}^{2}\text{ var}(x) \end{array}$$ (19)

We use the Lemma 1 with the real-valued function f→log, and for two random variables, x→m,v (RMS and variance), which are ergodic stationary stochastic processes.


$$\begin{array}{cc} & \log(m)\approx \log(E(m))+(m-E(m))/E(m) \end{array}$$ (20)

$$\begin{array}{cc} & ln(v)\approx ln(E(v))+(v-E(v))/E(v) \end{array}$$ (21)

$$\begin{array}{cc} & var(ln(m))\approx \frac{var(m)}{E(m{)}^{2}} \end{array}$$ (22)

where the approximation is good as long as *m* and *v* do not vary too much around E(*m*) and E(*v*), relative to the range of *x* for which first- and second-order Taylor expansions of the log(x) at E(*m*) and E(*v*) are good approximation. Therefore,


$$\begin{array}{c} \frac{\text{cov}(\log(m),\log(v))}{\text{var}(\log(m))}\approx \frac{\text{E}(m)}{\text{E}(v)}\frac{\text{cov}(m,v)}{\text{var}(m)}. \end{array}$$ (23)

Now, using Eqs. (20) and (21), we can write


$$\begin{array}{cc} cov(\ln(m),\ln(v)) & =\;E[XY]-E[X]E[Y]\\ & \approx \;E\left[\left(ln(E(m))+\frac{m-E(m)}{E(m)}\right)\left(ln(E(v))+\frac{v-E(v)}{E(v)}\right)\right]\\ & -ln(E(m))ln(E(v)) \end{array}$$ (24)

$$\begin{array}{cc} = & \;ln(E(m))ln(E(v))+\frac{E(m-E(m))}{E(m)}ln(E(v))+ln(E(m))\frac{E(v-E(v))}{E(v)}\\ & +\frac{E[(m-E(m))(v-E(v))]}{E(m)E(v)}-ln(E(m))ln(E(v)) \end{array}$$ (25)

$$\begin{array}{cc} \approx & \;\frac{E[(m-E(m))(v-E(v))]}{E(m)E(v)} \end{array}$$ (26)

$$\begin{array}{cc} \approx & \;\frac{cov(m,v)}{E(m)E(v)} \end{array}$$ (27)

Using equation Eqs. (22) and (27) we have,


$$\begin{array}{c} b=\frac{cov(ln(m),ln(v))}{var(ln(m))}\approx \frac{cov(m,v)E(m{)}^{2}}{E(m)E(v)var(m)}\approx \frac{E(m)cov(m,v)}{E(v)var(m)} \end{array}$$ (28)

Now, $var(m)=E(m^{2})-E(m{)}^{2}$, or, $E(m^{2})=var(m)+E(m{)}^{2}$, and $var(\mu )=E(\mu^{2})-E(\mu {)}^{2}$ or, $E(\mu^{2})=var(\mu )+E(\mu {)}^{2}$.

The RMS is defined as, $m=\sqrt{\frac{1}{n}\sum_{i=1}^{n}Y_{i}^{2}}$ or, $m^{2}=\frac{1}{n}\sum_{i=1}^{n}Y_{i}^{2}$. The variance is defined as, $v=\frac{1}{n-1}\sum_{i}Y_{i}^{2}-\frac{n}{n-1}\mu^{2}$, or, $v=\frac{n}{n-1}m^{2}-\frac{n}{n-1}\mu^{2}$. Taking expectation to the both side, $E(v)=\frac{n}{n-1}(E(m^{2})-E(\mu^{2}))$. We can rewrite as,


$$\begin{array}{c} E(v)=\frac{n}{n-1}(var(m)+E(m{)}^{2}-var(\mu )-E(\mu {)}^{2}). \end{array}$$ (29)

Next, putting the results from Eq. (29) into Eq. (28), we get,


$$\begin{array}{cc} b & \approx \frac{E(m)cov(m,v)}{E(v)var(m)} \end{array}$$ (30)

$$\begin{array}{cc} & =\frac{\left(n-1)E(m)cov(m,v\right)}{n(E(m{)}^{2}-E(\mu {)}^{2}+var(m)-var(\mu ))var(m)} \end{array}$$ (31)

$$\begin{array}{cc} & \approx \frac{\left(n-1)E(m\right)}{n}\frac{cov(m,v)}{\left(E(m{)}^{2}+var(m)-\Omega \right)var(m)} \end{array}$$ (32)

The first factor on the right-hand side of Eq. (32) solely depends on the number of location or brain areas, *n*, and the marginals $Y_{i}$, but not on their correlations. The quantities E(*m*) and var(*m*) also depend only on marginal distribution structure. The quantity var(μ), equals to $\frac{1}{n^{2}}\sum_{i,j}\text{cov}(Y_{i},Y_{j})$, is directly related and similar in form to the measure of coherence, i.e., the average spatial synchrony Ω. Therefore, Eq. (32) provides the intuition behind our analysis: only if synchrony (Ω or var(μ)) changes but the marginal $Y_{i}$ remain fixed, the slope *b* will be affected. In other words, if the long-term correlation among the spatio-temporal time series data varies, but the complexity in the time series remain fixed, the slope *b* will be altered. From a logical assumption, the time series complexity [120] can be represented by a specific dynamical nature, such as periodic, two periodic, quasiperiodic, chaotic and stochastic.

Since, the synchrony Ω is in the denominator, the slope *b* is inversely proportional to the changes in the synchrony, which captures long-term correlations across the entire space.

### Surrogate time series: Phase-randomization

We randomized phases of the empirical BOLD series to produce surrogated data utilized to examine marginal’s contributions to measurement. Given a time series *Y*(*t*), the complex valued Fourier transform can be written as,


$$\begin{array}{c} Y(f)=A(f)e^{i\phi (f)}. \end{array}$$

*A*(*f*) and ϕ(f) are amplitude and phase, respectively. We considered a univariate phase randomization using fast Fourier-transform surrogate data [80]. A ‘phase-randomized’ Fourier transform $\tilde{Y}(f)$ is made by randomly rotating the phase ϕ at each frequency *f* by an independent random variable φ which is chosen from uniform distribution in the range [0,π]. The φ’s are not completely independent, but instead are constrained to satisfy the symmetry property φ(f)=−φ(−f) to insure that the inverse Fourier transform is a time series of real values. That is,


$$\begin{array}{c} \tilde{Y}(f)=A(f)e^{i\varphi (f)}. \end{array}$$

From this surrogate time series is given by the inverse Fourier transform by,


$$\begin{array}{c} \tilde{Y}(t)={ℱ}^{-1}\{\tilde{Y}(f)\}={ℱ}^{-1}\{A(f)e^{i\varphi (f)}\}. \end{array}$$

By construction, $\tilde{Y}(t)$ will have the same power spectrum as the original data set *Y*(*t*), and by the Wiener-Khintchine theorem *t*he same autocorrelation function, i.e., the circular autocorrelation is preserved [80], when *Y*(*t*) has a zero temporal mean.

Repeated independent random surrogate realizations are used to build a statistical distribution of the quantity of interest under a well-defined null condition [80,121–123]. The phase randomization preserves the power spectrum, the autocorrelations of each of the *n* variables in the original data set, and also the cross-correlations between all the variables as well [2,80]. It has been widely applied in neuroscience for testing nonlinearity and coordination structure in EEG, MEG, fMRI data, and hypothesis testing of other physical systems [2,122,124,125]. Independence across realizations is guaranteed because, at each realization, Fourier phases are drawn freshly and independently from a uniform random distribution over [0, 2π) for each brain region and each participant, with no dependence on phases drawn in any previous realization– a property that directly follows from the independence of uniform random sampling [2,80].

## Supporting information

S1 Appendix Supplementary methods, figures and tables. Contains definitions of all metrics; the detailed analytical derivation of the synchrony-scaling relationship (Eq (6)) and the linear-fitting procedure for the scaling exponent; algorithms used to construct spatio-temporal time series; details of the empirical datasets and preprocessing; the phase-randomization procedure applied to the empirical BOLD signals; and the definitions of metastability, phase synchrony, and the entropy of functional connectivity dynamics. Fig A: visualization of the synchrony-induced scaling law. Fig B–Fig H: effect of shifting the temporal mean on the spatial SL slope for Poisson, Binomial, negative Binomial, Uniform, and Normal distributions (independent and identically distributed data). Fig I: spatial SL–average synchrony association for i.i.d. normal data (645 realizations). Fig J–Fig M: effect of average synchrony on the spatial SL slope for Poisson, negative Binomial, Gamma, and Normal distributions (identically distributed but not necessarily independent data). Fig N: empirical data and detrending operation in resting state. Fig O: empirical data and detrending operation in movie watching task. Fig P: empirical data and detrending operation in sensorimotor task. Fig Q: spatial RMS, variance, and spatial SL of three participants in three brain states. Fig R: spatial RMS, variance, and spatial SL of participants from young, middle-aged, and older groups in three brain states. Fig S: spatial SL exponent and average synchrony for the resting state, sensorimotor and movie watching task in the CamCAN aging cohort. Fig T: effects of age on the synchrony-driven spatial SL for three brain states. Fig U: group-wise analysis, age-related alterations in the synchrony-induced SL exponent in three brain states. Fig V: cross-validation in NKI dataset of healthy aging cohorts. Fig W: synchrony-induced scaling law in neurodevelopmental disorder. Fig X: age-related changes captured by other metrics (metastability, global phase synchrony, and entropy of functional connectivity dynamics). Table A: functional MRI scan acquisition parameters. Table B: details of the empirical data from three independent cohorts.
(PDF)
