# Supplementary material for: A generalized scaling law reveals synchrony-driven reorganization of brain dynamics across human lifespan
Source: PLoS Comput Biol. 2026 Oct 5;22(10):e1014821. doi: 10.1371/journal.pcbi.1014821 (PMC13652345; doi:10.1371/journal.pcbi.1014821)
Supplement: Supplementary file 1 [file pcbi.1014821.s001.pdf]

Supporting Information

for

A generalized scaling law reveals synchrony-driven reorganization of brain dynamics across the human lifespan

Suman Saha[1*], Chittaranjan Hens[2,3], Priyanka Chakraborty[4], Tomasz Kapitaniak[5], Gustavo Deco[6,7], Arpan Banerjee[8], Syamal Kumar Dana[5,9], and Dipanjan Roy[8,10*]

[1]School of Electronics Engineering, Vellore Institute of Technology, Chennai, Tamil Nadu 600127, India

[2]Center for Computational Natural Sciences and Bioinformatics, International Institute of Information Technology, Gachibowli, Hyderabad-500032, India

[3]Biomedical Research Center, International Institute of Information Technology, Gachibowli, Hyderabad-500032, India

[4]Department of Mathematics, Rampurhat College, West Bengal, India

[5]Division of Dynamics, Technical University of Lodz, 90-924 Lodz, Poland

[6]Centre for Brain and Cognition, Computational Neuroscience Group, Universitat Pompeu Fabra, Barcelona 08018, Spain

[7]Department of Information and Communication Technologies, Universitat Pompeu Fabra, Barcelona 08018, Spain

[8]Cognitive Brain Dynamics Lab, National Brain Research Centre, Manesar, Haryana 122052, India

[9]Centre for Mathematical Biology and Ecology, Department of Mathematics, Jadavpur University, Kolkata 700032, India

[10]Department of Bioscience and Bioengineering, Indian Institute of Technology, Jodhpur, Rajasthan, India.

*suman.saha@vit.ac.in (S.S.), *droy@iitj.ac.in (D.R.)

September 25, 2026

Section 1 shows the general framework for the main mathematical analysis. Definitions of the metrics are given in Section 2. Section 3 carries out the main analysis, which covers the case that the Y_i (see main text for a definition of Y_i) are identically distributed. Section 4 presents numerically simulated results for various case studies of distributions $Y = (Y_1, \cdots, Y_n)$, constructed by transforming multivariate random variables with identically distributed marginals Y_i, for both independent and not-necessarily-independent cases. Section 5 shows algorithms to construct multivariate time series for different case studies by randomly drawing values from various distributions. Section 6 shows details of the empirical data and results.

Contents

1 Visualization of the synchrony-induced scaling law **3**

2 Definitions **4**

2.1 Temporal mean (μ_t) 4

2.2 Temporal variance (v_t) 4

2.3 Spatial mean (μ) 4

2.4 Spatial variance (v) . . . 5
2.5 Spatial RMS (m) . . . 5
2.6 Average Synchrony (Ω) . . . 5

3 Analytical results **6**
3.1 Mathematical derivation of the spatial scaling exponent . . . 6
3.2 Linear Fitting . . . 8

4 Simulation-based results **10**
4.1 Effect of non-zero temporal mean on spatial scaling exponent: Independent and identically distributed . . . 10
4.1.1 Independent and identically distributed multivariate data: Poisson distribution . . . 10
4.1.2 Independent and identically distributed data: negative Binomial distributions . . . 13
4.1.3 Independent and identically distributed data: Uniform distribution . . . 17
4.1.4 Independent and identically distributed multivariate data: Normal distribution . . . 19
4.2 Summary: Independent and identically distributed data . . . 20
4.3 Identically distributed, but not necessarily independent . . . 21
4.3.1 Poisson distribution . . . 21
4.3.2 Negative Binomial distribution . . . 23
4.3.3 Gamma Distribution . . . 24
4.3.4 Normal Distribution . . . 25

5 Algorithm to construct multivariate time series **26**
5.1 Construct spatio-temporal time series: identically independent distributions . . . 26
5.1.1 Poisson distribution . . . 26
5.1.2 Binomial distribution . . . 26
5.1.3 Uniform distribution . . . 26
5.1.4 Normal distribution . . . 27
5.1.5 Construct spatio-temporal time series: identically distributed, but not necessarily independent . . . 27

6 Empirical data analysis using CamCAN dataset **28**
6.1 Data sources and participants . . . 28
6.2 Data preprocessing . . . 28
6.3 Surrogate time series: phase-randomization . . . 28
6.4 Empirical Results . . . 28
6.5 Empirical data visualization . . . 31
6.6 Task driven coordinated dynamics and spatial scaling law . . . 34
6.7 Aging effects on synchrony and spatial SL in three brain states . . . 35
6.8 Group-wise analysis: Age-related alterations in the synchrony-induced SL exponent under three brain states . . . 36

7 Cross validation in NKI dataset: Healthy aging cohort **37**

8 Synchrony-induced scaling in neural disorder in UCLA dataset **39**

9 Details of the Datasets **40**

10 Comparison with other metrics : Metastability, phase synchrony, entropy of functional connectivity dynamics **41**
10.1 Metastability and Phase Synchrony . . . 41
10.2 Entropy of functional connectivity dynamics . . . 42
10.3 Results . . . 42

1 Visualization of the synchrony-induced scaling law

Lets consider an arbitrary spatio-temporal time series data, $y_i(t)$, where, $i = 1, 2, ..., N$; N is the total number of nodes/sites/locations/brain areas. A Fig shows the schematic of the pipeline. The four colours represent resting state BOLD activity from four cortical regions. First, we apply detrending operation on the multivariate spatio-temporal time series. Thereafter, in the detrended data $Y_i(t)$, the additional trends and bias are removed, and temporal mean is set to zero. The bias can be described by taking an example from signal processing. Suppose, a DC voltage or a ramp like signal is added to a sinusoidal signal. Now, the signal starts oscillating around a positive voltage above the zero temporal mean. The detrending process removes the trend and DC bias. Thereafter, the signal starts oscillating around the zero mean voltage or zero temporal mean. We rounded up to 1e-6 decimal for zero value in numerical simulation. In second column in A Fig, the instantaneous values ($Y_i(t_1)$ at time t_1, or $Y_i(t_2)$ at time t_2

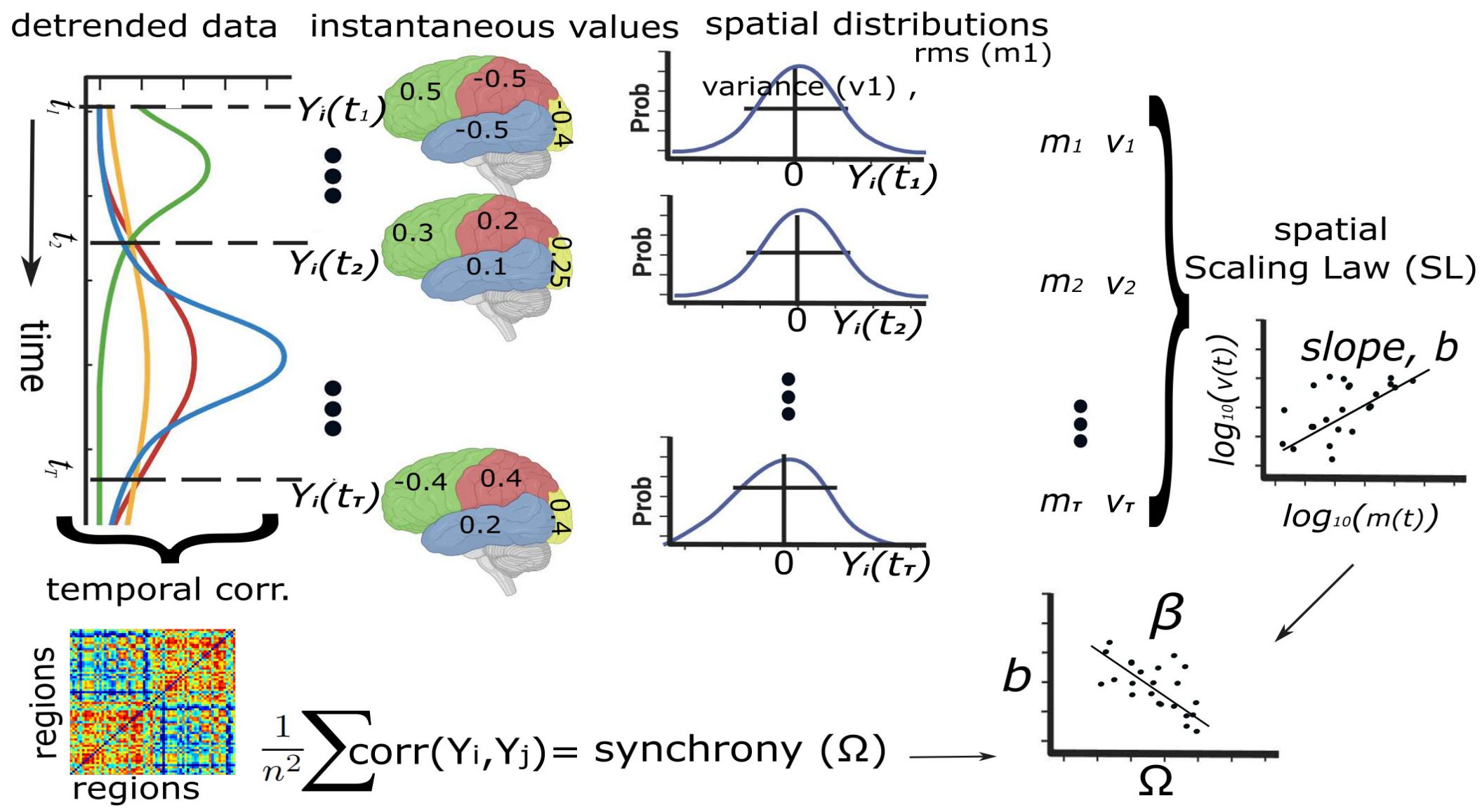

Fig A. Schematic diagram of the proposed pipeline has been illustrated using a BOLD series from a participant. First step of our method involves, detrending process, where we remove the trends and the temporal mean is set to zero. Next, the amplitude of the detrended BOLD signal of all the brain regions are squared and summed at each instant to obtain spatial RMS at time t, Similarly, we derive spatial variance at each time point. For example, $m_1(t_1)$ and $v_1(t_1)$, where the subscript denotes the time index, are the spatial RMS and variance, respectively, derived at first time point $t = t_1$. Likewise, we derived the values of $m(t)$ and $v(t)$ for every instances. The exponent (b) of the least-square linear fit between the spatial RMS ($\log_{10}(v)$) and variance ($\log_{10}(v)$) is obtained, besides the average spatial synchrony (Ω) is also calculated for individual participants. Finally, we make a scatter plot to examine the relationship between synchrony (Ω) and SL exponent (b) using a linear regression model. The slope β and p-value describe the influence of the synchrony on the emergence of the spatial SL in both the empirical and numerical examples.

or $Y_i(t_n)$ at time t_n) of the detrended data from all the four brain regions are shown on the brain plot. The spatial variance (v_1) and spatial root-mean-square (RMS, m_1) value are then derived from the spatial distribution over the entire space for each time instant. For example, the instantaneous values of spatial RMS $m_1(t_1)$ and spatial variance $v_1(t_1)$, where the subscript denotes the time index, are derived at first time point $t = t_1$. Likewise, we derived the values of $m(t)$ and $v(t)$ for every times points. Next, the exponent b is obtained from the log-log relationship between the RMS and variance, which is referred to as spatial scaling law (spatial SL). We derive the long-time correlation using Pearson correlation coefficient between any pair of regions, which gives a region-vs-region (space-vs-space) square matrix. Averaging over the absolute values of the entire matrix the spatial synchrony (Ω) is obtained. Finally, we examine the impact of synchrony on the spatial SL exponent from a linear fitting using common least square regression, considering Ω and b as independent and dependent variables, respectively. From the regression model, we estimate the slope (β), that implies the degree of influence of synchrony on the SL. In addition, we

analytically derive the approximate formula for the spatial SL as a function of synchrony. We have tested the method on empirical examples using fMRI data from CamCAN healthy aging cohorts; see Empirical results section. The metric β is used to examine the changes in rest and task evoked BOLD activity across the adulthood aging trajectory.

2 Definitions

Suppose the amplitude (e.g., population density, voltage, amplitude of blood-oxygen level dependent activity) of the spatio-temporal time series data in location i at time t is modeled by the random variable $Y_i(t)$, $i = 1, ..., n$ and n = total number of sites (e.g., nodes in a network, number of patches in ecological network, number of brain areas or region of interests). We assume that the multivariate stochastic process $Y(t)$ is stationary and ergodic [1, 2].

A wide range of stochastic processes have the property that their average statistical properties are independent of where they are formed along the time [3]. For a stationary signal, the basic signal properties of mean and variance do not change over time [4]. Roughly speaking, a "stationary" process is one whose joint probability distribution is not affected by shifts in time, so that mechanisms of the dynamics are not shifting in time [2]. For such a signal, the measurement of the mean or variance over only one segment is sufficient to estimate the signal's true mean [3, 4]. An "ergodic" process is one for which statistical characteristics can be deduced from a single, sufficiently long realization of the process, so that, for instant, long-term statistical outcomes are independent of initial conditions [2]. Ergodicity is a more restrictive concept compared with that of wide-sense stationarity, or even of stationarity in the strict sense [5]. More precisely, for an ergodic process, time averages coincide with ensemble averages [5]. In neurophysiological experiments, the validity of the ergodicity assumption, i.e., replacing ensemble means by time means, is often implicitly assumed [3]. Precise definitions of stationary and ergodic processes can be found in Ref. [1]. The definition of the spatial RMS, sample mean, spatial variance and synchrony of a given multivariate time series data are given below.

2.1 Temporal mean (μ_t)

Temporal mean [6] for a given raw data, i.e., spatio-temporal time series, $Y_i(t)$ is computed for individual site i, as

$$\mathrm{E}[Y_i(t)]_t = \mu_{it} = \langle Y_i(t) \rangle_t \,, \tag{S1}$$

where, i represents index of the space. $\langle . \rangle$ denotes long-time average, or average over one cycle when $Y_i(t)$ is periodic. After detrend operation, the temporal mean μ_t becomes 0.

2.2 Temporal variance (v_t)

Temporal variance [2] of location i over the entire time length is expressed as,

$$\mathrm{V}[Y_i(t)]_t = v_{it} = \left\langle (Y_i(t) - \mathrm{E}[Y_i(t)]_t)^2 \right\rangle = \left\langle (Y_i(t))^2 - \mu_t^2 \right\rangle . \tag{S2}$$

This spatial quantity measures dispersion or spread of the data points around the temporal mean (μ_t).

2.3 Spatial mean (μ)

The arithmetic mean or sample mean over all regions at time t is calculated by

$$\mu(t) = \frac{1}{n} \sum_{i=1}^{n} Y_i(t), \tag{S3}$$

which measures the central tendency of the sample data at time t. The spatial mean contains negative and positive values $\mu \in [-1, 1]$, where var(μ)>0, and E(μ)= 0.

2.4 Spatial variance (v)

Spatial variance [2] over the space at a time instant t is expressed by,

$$v(t) = \sum_{i=1}^{n} \frac{Y_i^2(t)}{n-1} - \frac{n\,\mu^2(t)}{n-1}. \tag{S4}$$

This spatial quantity measures dispersion or spread of the data points around the sample/ensemble/spatial mean (μ).

2.5 Spatial RMS (m)

The spatial root-mean-square (RMS) is defined as square root of the quadratic mean, i.e., RMS over the entire space (e.g., over all brain regions) at time t as,

$$m(t) = \sqrt{\frac{1}{n}\sum_{i=1}^{n} Y_i^2(t)}. \tag{S5}$$

Thus, the spatial RMS of the signals takes into account the squared values of all the data points directly, indicating effective magnitude of the signal, averaged across space at each time instant. The values of m are always positive, i.e., $m > 0$.

2.6 Average Synchrony (Ω)

The term 'synchrony', used in this study, is referred to as a global metric that quantifies the strength of coordinated cortical activity or brain-wide coactivation pattern. To define the metric synchrony, we have first selected a very fundamental measure, functional connectivity (FC), frequently used in neuroscience to study resting state fMRI data. The FC matrix is computed using Pearson correlation coefficient that captures the long-term correlation or degree of coherence between two BOLD signals from each pair of brain regions. We get a symmetric square matrix of size=regions X regions, where the off-diagonal entries can vary between [-1,1]. Finally, we averaged the entire matrix which is referred to as the spatial synchrony (Ω), that measures the strength of coherence in BOLD activity of individual adults. The standard measure of average spatial synchrony is also used earlier in ecological population [2, 7]. Expression for the global metric, average synchrony is,

$$\Omega = \frac{1}{n^2}\sum_{j=1}^{n}\sum_{i=1}^{n} \mathrm{cor}_t(Y_i(t), Y_j(t)) = (1/n^2)\sum_{i,j=1}^{n} FC_{i,j}. \tag{S6}$$

where, $FC_{i,j} = \mathrm{cor}(Y_i, Y_j)$. FC is the static functional connectivity matrix with a size ($n \times n$), measured by Pearson correlation coefficient between the time series of a pair of locations. n is the number of locations, sites, space, ensembles, nodes in a network, or, brain regions. For example, n is the total number of brain regions for a given brain atlas, such as, automated anatomical labelling (AAL) atlas [8], where $n = 116$.

3 Analytical results

3.1 Mathematical derivation of the spatial scaling exponent

Lets assume, the amplitude of a spatio-temporal time series data at time t in i^{th} location is modeled by a real valued random variable $Y_i(t)$ for $i = 1, \ldots, n$, where $Y(t) = (Y_1, \ldots, Y_n)$ is stationary and ergodic. We are interested in the $\log(v)$-$\log(m)$ scatter plot for a finite duration realization of these processes (ignoring the times t, when $m(t)$ or $v(t)$ was zero), and particularly in the slope and intercept of an ordinary linear regression through such a plot,

$$b = \frac{\text{cov}\left(\log(m(t)), \log(v(t))\right)}{\text{var}(\log(m(t)))}, \tag{S7}$$

$$\log(a) = \text{E}(\log(v(t))) - b\text{E}(\log(m(t))). \tag{S8}$$

These are the two basic quantities in the spatial scaling law (SL). The covariance, variance, and RMS are sample quantities computed over time for the finite-duration realization. Given the ergodicity of Y and consistency of the spatial RMS, mean, variance and covariance, we consider the previous equations as a general form in the sequel for large samples, valid for both temporal and spatial cases. We compute the covariance, variance, RMS, and expected values for the marginal distributions of the processes m and v when these quantities are positive, and likewise we get the same results from the positive- and negative-valued data distributions. Furthermore, we assume that both the scaling parameters (m and v) are positive and finite.

Suppose the long-term temporal correlations $\rho_{ij} = \text{cor}(Y_i, Y_j)$ exist for all $i, j = 1, \cdots, n$. These temporal correlations between a pair of sites represent spatial synchrony between the regions i and j (again making use of ergodicity, and consistency of the sample covariance and variance). The synchrony, $\Omega = \frac{1}{n^2}\sum_i \sum_j \rho_{ij}$ is an average correlation across all pairs of sampling locations (including comparisons of a location with itself, which give $\rho_{ij} = 1$, and is commonly used in empirical studies. We will analyze the influence of Ω on b. Ω is naturally interpretable as a measure of average coherence in anti- and in-phase state for the following additional reasons. First, letting, $Z_i = Y_i/\sqrt{\text{var}(Y_i)}$, we have

$$\Omega = \frac{1}{n^2}\sum_{i=1}^{n}\sum_{j=1}^{n} \text{cor}(Y_i(t), Y_j(t)) \tag{S9}$$

$$= \frac{1}{n^2}\sum_{i=1}^{n}\sum_{j=1}^{n} \text{cor}(Z_i(t), Z_j(t)) \tag{S10}$$

$$= \frac{1}{n^2}\sum_{i=1}^{n}\sum_{j=1}^{n} \text{cov}(Z_i(t), Z_j(t)) \tag{S11}$$

$$= \frac{1}{n^2}\,\text{cov}\left(\sum_{i=1}^{n} Z_i(t), \sum_{j=1}^{n} Z_j(t)\right) \tag{S12}$$

$$= \text{var}\left(\frac{1}{n}\sum_{i=1}^{n} Z_i(t)\right) \tag{S13}$$

$$= \frac{\text{var}\left(\frac{1}{n}\sum_{i=1}^{n} Z_i(t)\right)}{\frac{1}{n}\sum_{i=1}^{n}\text{var}(Z_i(t))} \tag{S14}$$

$$= \text{var}\left(\mu(t)\right) \tag{S15}$$

where, Eq. (S15) is the variance of the spatial mean μ, and is readily interpretable as the extent to which oscillations in local time series reinforce each other or cancel in the average time series. In all our examples for the detrended data, $\text{var}(Z_i(t)) = 1$.

Second, Eq. (S15) and the definition of Ω show that $0 \leq \Omega \leq 1$. The value 0 occurs when the variability in the Z_i exactly cancels, i.e., $\text{var}(\frac{1}{n}\sum_i Z_i)$=0 and perfect asynchrony. The value 1 occurs for perfect synchrony, when $Z_i = Z_j$ for all i, j. An alternative measure for average synchrony is $\left|\frac{1}{n(n-1)}\sum_{i\neq j}\rho_{ij}\right|$.

The correlation coefficient ρ can take negative values. In case of perfect anti-synchrony and synchrony, the Ω can be 1, except, for perfect asynchrony.

To understand the relationship between synchrony and scaling law for large samples under assumptions of stationarity and ergodicity, it is sufficient to work with the marginal distribution Y, which follow a normal distribution in our case, instead of with the process $Y(t)$. We therefore consider families of distributions of the form $Y = (Y_1, \cdots, Y_n)$ such that for each i, all members of the family have the same marginals Y_i, and we consider how Ω and b covary through the family before and after the detrending operation. After detrending operation, the underlying distribution of the marginal started following Gaussian normal distribution. In the theoretical development, marginals are kept constant to understand the influence of synchrony Ω in isolation from other potential influences on b. To describe the intuition of the mathematical analyses that will be described in details in the next section, we need a lemma, which is taken directly from [2, 9–11].

Lemma 1. Let x be a real valued random variable with finite mean $\mathrm{E}(x)$ and variance $\mathrm{var}(x)$. If a real-valued function f of real x is twice differentiable at $\mathrm{E}(x)$, then the *delta method* [10] gives

$$f(x) \approx f(\mathrm{E}(x)) + (X - \mathrm{E}(x))f'(\mathrm{E}(x)) \tag{S16}$$

$$\mathrm{E}(f(x)) \approx f(\mathrm{E}(x)) + \frac{1}{2}f''(\mathrm{E}(x))\ \mathrm{var}(x) \tag{S17}$$

$$\mathrm{var}(f(x)) \approx [f'(\mathrm{E}(x))]^2\ \mathrm{var}(x) \tag{S18}$$

We use the Lemma 1 with the real-valued function $f \to \log$, and for two random variables, $x \to m, v$ (RMS and variance), which are ergodic stationary stochastic processes.

$$\log(m) \approx \log(\mathrm{E}(m)) + (m - \mathrm{E}(m))/\mathrm{E}(m) \tag{S19}$$

$$\ln(v) \approx \ln(\mathrm{E}(v)) + (v - \mathrm{E}(v))/\mathrm{E}(v) \tag{S20}$$

$$\mathrm{var}(\ln(m)) \approx \frac{\mathrm{var}(m)}{\mathrm{E}(m)^2} \tag{S21}$$

where the approximation is good as long as m and v do not vary too much around $\mathrm{E}(m)$ and $\mathrm{E}(v)$, relative to the range of x for which first- and second-order Taylor expansions of the $\log(x)$ at $\mathrm{E}(m)$ and $\mathrm{E}(v)$ are good approximation. Therefore,

$$\frac{\mathrm{cov}(\log(m), \log(v))}{\mathrm{var}(\log(m))} \approx \frac{\mathrm{E}(m)}{\mathrm{E}(v)} \frac{\mathrm{cov}(m, v)}{\mathrm{var}(m)}. \tag{S22}$$

Now, using Eqs. (S19) and (S20), we can write

$$\begin{aligned}
\mathrm{cov}(\ln(m), \ln(v)) &= \mathrm{E}[XY] - \mathrm{E}[X]\mathrm{E}[Y] \\
&\approx \mathrm{E}\left[\left(\ln(\mathrm{E}(m)) + \frac{m - \mathrm{E}(m)}{\mathrm{E}(m)}\right)\left(\ln(\mathrm{E}(v)) + \frac{v - \mathrm{E}(v)}{\mathrm{E}(v)}\right)\right] \\
&\quad - \ln(\mathrm{E}(m))\ln(\mathrm{E}(v)) && \text{(S23)} \\
&= \ln(\mathrm{E}(m))\ln(\mathrm{E}(v)) + \frac{\mathrm{E}(m - \mathrm{E}(m))}{\mathrm{E}(m)}\ln(\mathrm{E}(v)) + \ln(\mathrm{E}(m))\frac{\mathrm{E}(v - \mathrm{E}(v))}{\mathrm{E}(v)} \\
&\quad + \frac{\mathrm{E}[(m - \mathrm{E}(m))(v - \mathrm{E}(v))]}{\mathrm{E}(m)\mathrm{E}(v)} - \ln(\mathrm{E}(m))\ln(\mathrm{E}(v)) && \text{(S24)} \\
&\approx \frac{\mathrm{E}[(m - \mathrm{E}(m))(v - \mathrm{E}(v))]}{\mathrm{E}(m)\mathrm{E}(v)} && \text{(S25)} \\
&\approx \frac{\mathrm{cov}(m, v)}{\mathrm{E}(m)\mathrm{E}(v)} && \text{(S26)}
\end{aligned}$$

Using equation Eqs. (S21) and (S26) we have,

$$b = \frac{\mathrm{cov}(\ln(m), \ln(v))}{\mathrm{var}(\ln(m))} \approx \frac{\mathrm{cov}(m, v)\mathrm{E}(m)^2}{\mathrm{E}(m)\mathrm{E}(v)\mathrm{var}(m)} \approx \frac{\mathrm{E}(m)\mathrm{cov}(m, v)}{\mathrm{E}(v)\mathrm{var}(m)} \tag{S27}$$

Now, $\mathrm{var}(m) = \mathrm{E}(m^2) - \mathrm{E}(m)^2$, or, $\mathrm{E}(m^2) = \mathrm{var}(m) + \mathrm{E}(m)^2$, and $\mathrm{var}(\mu) = \mathrm{E}(\mu^2) - \mathrm{E}(\mu)^2$ or, $\mathrm{E}(\mu^2) = \mathrm{var}(\mu) + \mathrm{E}(\mu)^2$.

The RMS is defined as, $m = \sqrt{\frac{1}{n}\sum_{i=1}^{n} Y_i^2}$ or, $m^2 = \frac{1}{n}\sum_{i=1}^{n} Y_i^2$. The variance is defined as, $v = \frac{1}{n-1}\sum_i Y_i^2 - \frac{n}{n-1}\mu^2$, or, $v = \frac{n}{n-1}m^2 - \frac{n}{n-1}\mu^2$. Taking expectation to the both side, $\mathrm{E}(v) = \frac{n}{n-1}(\mathrm{E}(m^2) - \mathrm{E}(\mu^2))$. We can rewrite as,

$$\mathrm{E}(v) = \frac{n}{n-1}(\mathrm{var}(m) + \mathrm{E}(m)^2 - \mathrm{var}(\mu) - \mathrm{E}(\mu)^2). \tag{S28}$$

Next, putting the results from Eq. (S28) into Eq. (S27), we get,

$$b \approx \frac{\mathrm{E}(m)\mathrm{cov}(m,v)}{\mathrm{E}(v)\mathrm{var}(m)} \tag{S29}$$

$$= \frac{(n-1)\mathrm{E}(m)\mathrm{cov}(m,v)}{n(\mathrm{E}(m)^2 - \mathrm{E}(\mu)^2 + \mathrm{var}(m) - \mathrm{var}(\mu))\mathrm{var}(m)} \tag{S30}$$

$$\approx \frac{(n-1)\mathrm{E}(m)}{n}\frac{\mathrm{cov}(m,v)}{(\mathrm{E}(m)^2 + \mathrm{var}(m) - \Omega)\,\mathrm{var}(m)} \tag{S31}$$

The first factor on the right-hand side of Eq. (S31) solely depends on the number of location or brain areas, n, and the marginals Y_i, but not on their correlations. The quantities $\mathrm{E}(m)$ and $\mathrm{var}(m)$ also depend only on marginal distribution structure. The quantity $\mathrm{var}(\mu)$, equals to $\frac{1}{n^2}\sum_{i,j}\mathrm{cov}(Y_i, Y_j)$, is directly related and similar in form to the measure of coherence, i.e., the average spatial synchrony Ω. Therefore, Eq. (S31) provides the intuition behind our analysis: only if synchrony (Ω or $\mathrm{var}(\mu)$) changes but the marginal Y_i remain fixed, the slope b will be affected. In other words, if the long-term correlation among the spatio-temporal time series data varies, but the complexity in the time series remain fixed, the slope b will be altered. From a logical assumption, the time series complexity [12] can be represented by a specific dynamical nature, such as periodic, two periodic, quasiperiodic, chaotic and stochastic.

Since, the synchrony Ω is in the denominator, the slope b is inversely proportional to the changes in the synchrony, which captures long-term correlations across the entire space.

3.2 Linear Fitting

Dropping the time dimension, the spatial RMS at time t is expressed as,

$$m = \sqrt{1/n\sum_{i=1}^{n} Y_i^2}, \quad m^2 = 1/n\sum_{i=1}^{n} Y_i^2. \tag{S32}$$

Sample mean at time t is $\mu = 1/n\sum_{i=1}^{n} Y_i$.
Spatial variance at time t is,

$$\begin{aligned} v &= \frac{1}{n-1}\sum_{i=1}^{n} Y_i^2 - \frac{n}{n-1}\left(\frac{1}{n}\sum_{i=1}^{n} Y_i\right)^2 \\ &= \frac{n}{n-1}(1/n)\sum_{i=1}^{n} Y_i^2 - \frac{n}{n-1}\mu^2 \\ &= \frac{n}{n-1}\left(m^2 - \mu^2\right) \end{aligned} \tag{S33}$$

Taking ‘log’ to both sides of Eq. (S33), we get,

$$\begin{aligned} \log(v) &= \log\left(m^2 - \mu^2\right) + \log\left(\frac{n}{n-1}\right) \\ &= \log\left(m^2\left(1 - \frac{\mu^2}{m^2}\right)\right) + \log\left(\frac{n}{n-1}\right) \\ &= \log(m^2) + \log\left(1 - \frac{\mu^2}{m^2}\right) + \log\left(\frac{n}{n-1}\right) \end{aligned} \tag{S34}$$

Detrending sets the temporal mean to zero, $\mu_t = 0$; though, this can take any value in natural or physical processes prior to detrending. Thus, the data tends to follow Gaussian distribution, and the second term in Eq. (S34) will be

$$\log\left(1 - \frac{\mu^2}{m^2}\right) \to 0, \text{ and } |m| \gg |\mu|. \tag{S35}$$

Eq. (S34) can be written as,

$$\log(v) \simeq \log(m^2) + \log\left(\frac{n}{n-1}\right) = 2\log(m) + \log\left(\frac{n}{n-1}\right). \tag{S36}$$

From the log-log fitting between the two quantities m and v, in a normally distributed data, the SL exponent is found as, $b = 2$. Thereby, we considered normal distribution as a baseline distribution corresponding to the contribution coming from data marginals, which is further utilized to remove marginal effect on the measurement in empirical data analysis.

The SL slope b was computed through ordinary linear regression of $\log(v)$ against $\log(m)$ using most common least-square fit. In this study, we used the least-square regression in all our linear fittings using MATLAB function 'fitlm', and Python function 'stats.linregress'. For each block, residuals of the $\log(v)$-versus-$\log(m)$ regression were computed and used to generate a root mean squared error (RMSE). The mean (across block) RMSE was plotted against average synchrony Ω.

$$V_{fit} = \text{intercept} + b\,\log(m) \tag{S37}$$

$$RMSE = \sqrt{\left\langle [V_{fit} - \log(v)]^2 \right\rangle} \tag{S38}$$

4 Simulation-based results

The multivariate time series is first generated by randomly drawn values from binomial distributions. The data size is $T \times n$, where T and n represent total time points and total number of spaces, respectively. The space can be considered as total number of brain regions or total number of nodes in a network. We repeatedly generated the time series for 645 independent realizations for each distribution type.

4.1 Effect of non-zero temporal mean on spatial scaling exponent: Independent and identically distributed

We have considered two cases, e.g., without and with detrending operations. We have detrended the multivariate time series. Detrending removes the additional bias from each time series data by setting temporal mean to zero. The detrended data now follow normal distribution. The spatial scaling slope is observed as $b \simeq 2$ in all experimental distributions after detrending.

4.1.1 Independent and identically distributed multivariate data: Poisson distribution

B Fig shows numerical test on a spatio-temporal time series, size 261×116 (time points are 261 and number of locations are 116) has been generated by randomly drawing values from Poisson distribution with λ=50. We first generated a spatio-temporal data, histogram in red is plotted for case 1, the temporal mean at 50. Next, we have shifted the mean of the entire data, a constant value 20 to/from all is added or subtracted (temporal mean=70 for case 2 in black, and temporal mean =30 for case 3 in green) to the temporal of the original data (red) while freezing the variance and auto-correlation. Different random seeds are selected to construct time series for each locations. In all test cases we have considered an average correlation, between any pair of locations, close to zero, i.e., identical and independently distributed through time at each location.

We have plotted single time series out of 116 locations for three different cases 1, 2, 3 in red, black and green, respectively. Time series, histogram plot of the data distribution and cumulative distributions are shown in top row in B Fig, respectively, arranged from left to right order. All the three cases 1, 2 and 3 are again plotted separately in fourth, third and second rows, respectively in B Fig. The spatial SL slope for case 1,2 and 3 are b=2.027, 2.497, 1.567, and the spatial TL slopes are b=1.18, 1.657, 0.711.

After detrending, the temporal mean is set to zero in all three cases. Thereafter, the saptio-temporal time series no longer follow Poisson distribution. The time series, histogram of the data distribution and cumulative distribution are visualized in blue referred as case 4 in top row in B Fig, and also separately plotted in bottom row. After detrending, the data tends to follow normal distribution, as seen in the cumulative distribution plot in blue for case 4. The spatial SL slope is found to be b=2 shown in the bottom row in B Fig.

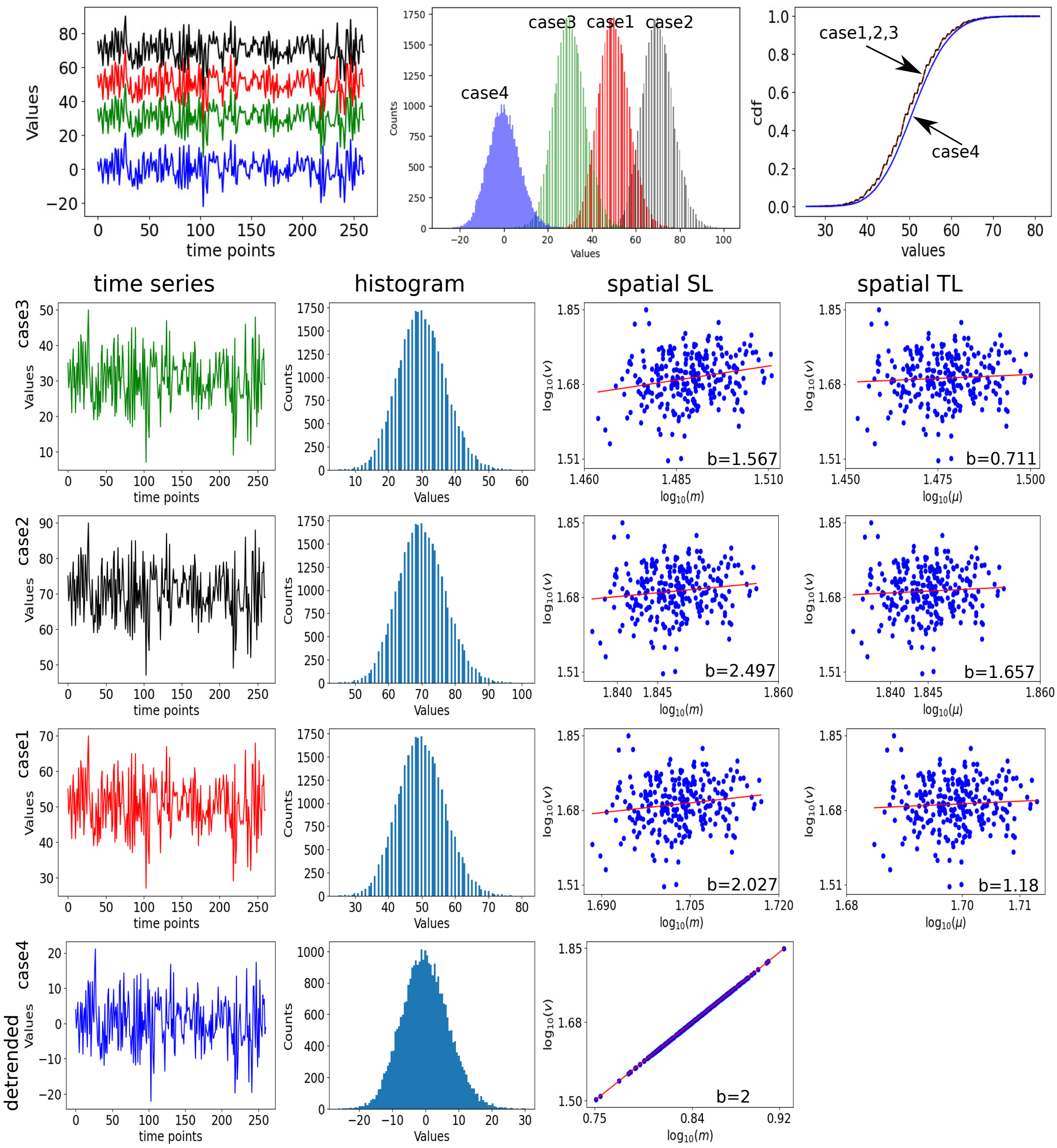

Fig B. Poisson distribution: effect of shifting the temporal mean on the spatial SL slope. We have used Python function "numpy.random.poisson(50, size= (261, 116))" to create the spatio-temporal time series data. The seeds are varied randomly for different locations. Long-term correlation between any pair of locations are almost zero. Next, shifted the temporal mean of the entire data by adding and subtracting 50 from the previous case example to create case 2 (black) and case 3 (green) examples. The detrended scenario, labeled as case 4, shown in blue at the bottom row.

We have repeated the similar steps to generate the plots in C Fig under the same statistical settings for Poisson ($\lambda = 30, 50, 70$) for another realization and derive the spatial SL and TL slopes. Compare to previous example shown in B Fig, for this test model, another realization of the time series are taken into consideration, when other properties are not disturbed. The average spatial correlation is close to zero. The spatial SL slope for case 1, 2 and 3 (red) are b=1.497, 1.729, 1.278 and the spatial TL slopes are b=0.593, 0.831, 0.355. We observed different SL exponent values because of the shifts in only the mean values of the distributions, which eventually alter the SL and TL slopes. These are different compared to the previous observations shown in B Fig. Next, we detrend the multivariate data, which sets temporal mean to zero in all the three cases. The time series of one location, the data distribution and the cumulative distribution are visualized in blue referred as case 4 in top row, in C Fig, the time series and distribution of the detrended data are plotted in bottom row first and second column, respectively, in C Fig. In the detrended cases, the spatial SL slope is found b=2.001 shown in bottom row third column in C Fig.

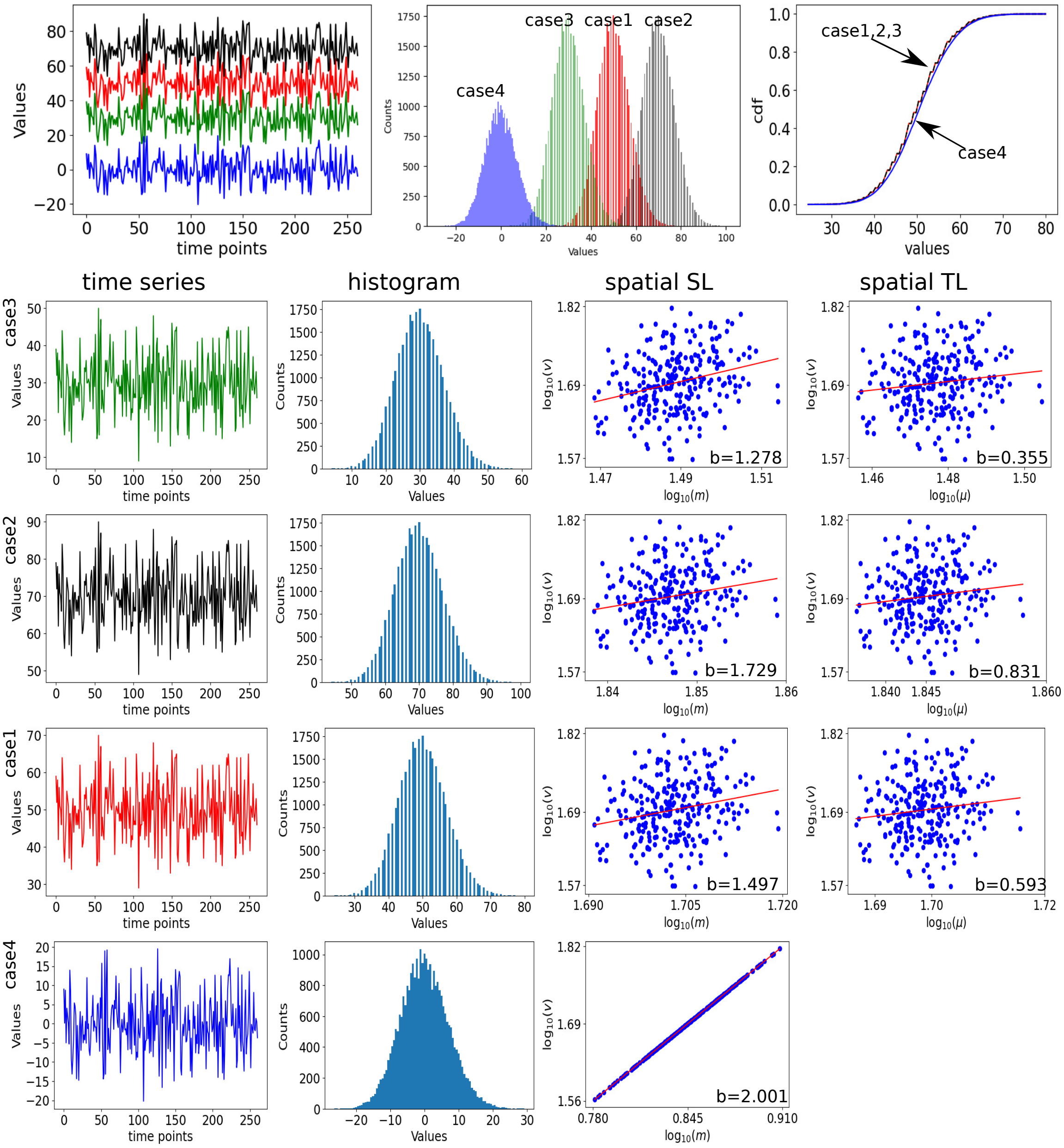

Fig C. Poisson distribution: effect of shifting the temporal mean on the spatial SL slope. We have used Python function "numpy.random.poisson(50, size= (261, 116))" to create the spatio-temporal time series data shown in red. Next, shifted the temporal mean of the entire data by adding and subtracting 50 from the previous case example to create case 2 (black) and case 3 (green) examples. The detrended scenario, labeled as case 4, shown in blue at the bottom row.

4.1.2 Independent and identically distributed data: negative Binomial distributions

D Fig shows numerical test on a spatio-temporal time series, size 261×116 (time points are 261 and number of locations are 116) has been generated by randomly drawing values from Binomial distribution with trials $n = 100$ and probability $p = 0.5$. We first generated a spatio-temporal data, histogram in red is plotted for case 1, the temporal mean at 125. Next, we have shifted the mean of the entire data, a constant value 50 and 100 are added to the temporal of the original time series data (red) while freezing the variance and auto-correlation, shown in black and green (case 2 and case 3). Different random seeds are selected to construct time series for each locations. In all test cases we have considered an average correlation, between any pair of locations, close to zero, i.e., identical and independently distributed through time at each location.

We have plotted single time series out of 116 locations, in green, red and black colors for three different cases, such as, temporal mean=125, 175 and 225 labeled as case 1, 2, 3, and corresponding colors are red, black and green, respectively. Time series, data distribution and cumulative distributions are shown in D Fig, respectively plotted from left to right order in top row. All the three cases 1, 2 and 3 are separately plotted in fourth, third and second rows, respectively in D Fig. The spatial SL slope for case 1,2 and 3 are b= -0.272, -0.82, -1.36, and the spatial TL slopes are b=-1.3, -1.84, -2.38.

After detrending, the temporal mean is set to zero in all three cases. Thereafter, the saptio-temporal time series no longer follow negative Bionomial distribution. The time series, histogram of the data distribution and cumulative distribution are visualized in blue referred as case 4 in top row in D Fig, and also separately plotted in bottom row. After detrending, the data tends to follow normal distribution, as seen in the cumulative distribution plot in blue for case 4. The spatial SL slope is found to be b=2 shown in the bottom row in D Fig.

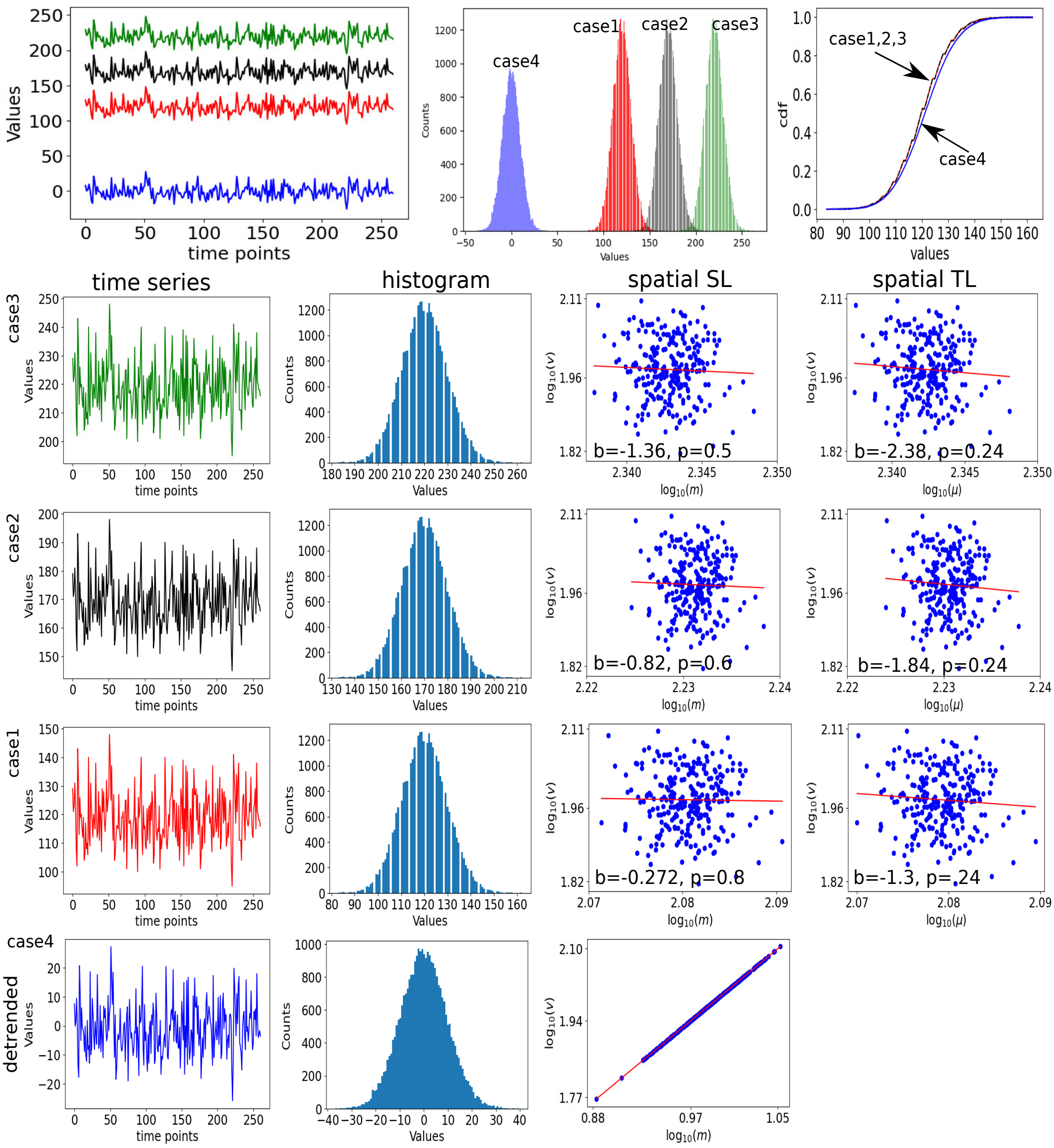

Fig D. Binomial distribution: effect of shifting the temporal mean on the spatial SL slope. The Python function 'numpy.random.binomial(n=100, p=0.5, size= (261, 116))' has been used to construct the time series. The first distribution created with the temporal mean at 125, shown in red labeled as case 1. Next, we have shifted the mean of the entire data, a constant value 50 and 100 are added to the temporal of the original time series data (red) while freezing the variance and auto-correlation, shown in black and green (case 2 and case 3). Detrended data analysis is marked as case 4, shown in blue at the bottom row.

We have repeated the similar steps followed in D Fig and generated the plots in E Fig under similar statistical settings for Binomial distribution (temporal mean=125, 175 and 225) for another realization and derive the spatial SL and TL slopes. Compare to previous example shown in D Fig, for this second test in E Fig, only time series fluctuations are different, but other properties are not disturbed. The average spatial correlation is close to zero. The spatial SL slope for case 1, 2 and 3 are b=4, 5.31, 6.62, 5.31 and the spatial TL slopes are b= 3.13, 4.43, 5.74. We observed different SL exponent values because of the shifts to the mean values of the distributions for three cases 1,2 and 3, which eventually alter the SL and TL slopes, as seen in E Fig. Next, we detrend the multivariate data, which sets temporal mean to zero in all the three cases. The time series of one location, the data distribution and the cumulative distribution are visualized in blue referred as case 4 in top row, in E Fig, the time series and distribution of the detrended data are plotted in bottom row first and second column, respectively, in E Fig. In the detrended cases, the spatial SL slope is found b=2.001 shown in bottom row third column in E Fig.

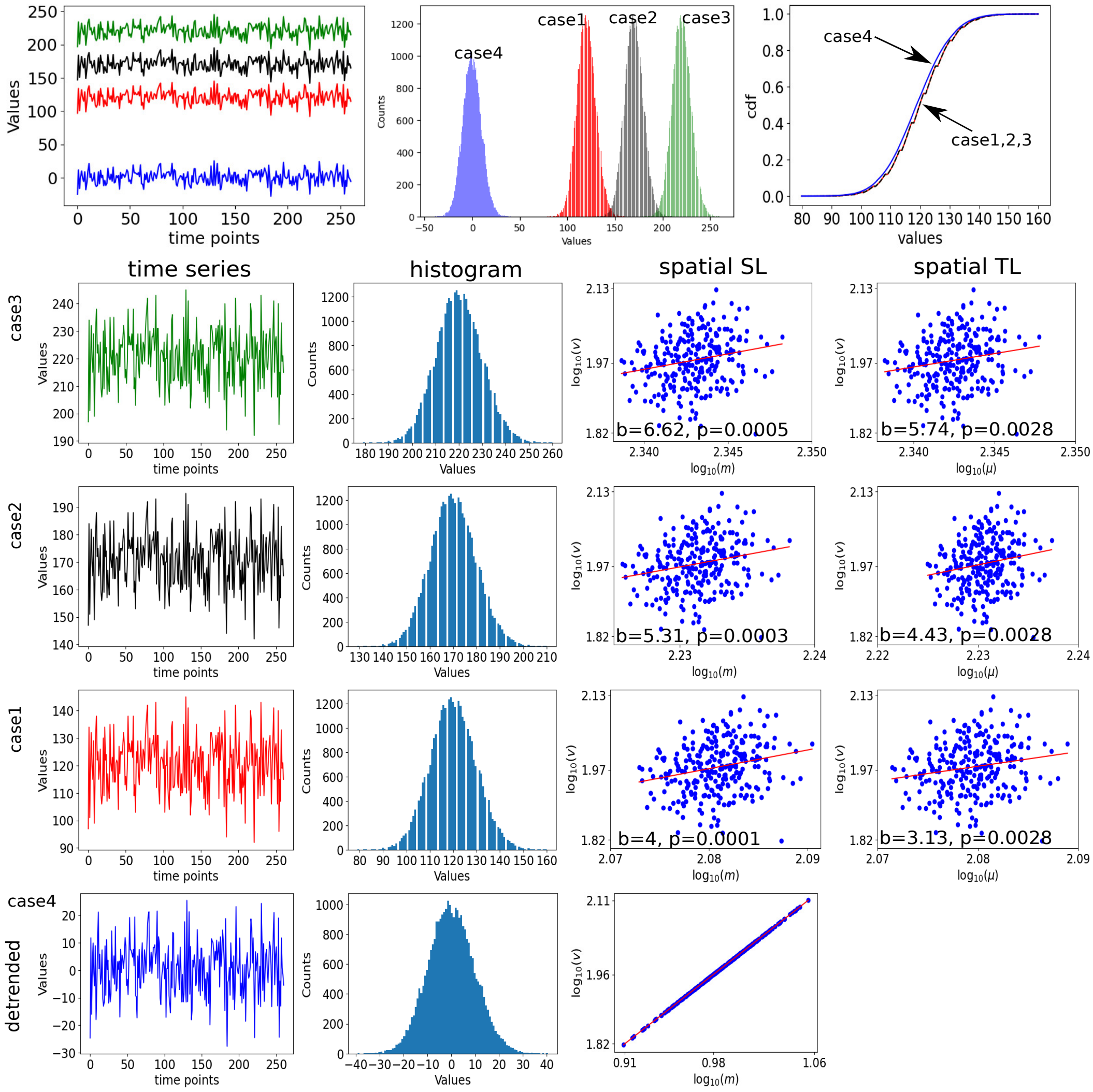

Fig E. Binomial distribution: effect of shifting the temporal mean on the spatial SL slope. The Python function 'numpy.random.binomial(n=100, p=0.5, size=(261, 116))' has been used to construct the time series.

We have repeated the steps followed in previous two cases D and E Figs to generated the plots in F Fig for negative Binomial distribution (temporal mean=0.1, 0.6 and 1.1, labeled as case1, 2 and 3, corresponding colors are green, red and black) and derive the spatial SL and TL slopes in three cases. The spatio-temporal time series constructed for case 2 (red) and case 3 (black) are generated by adding constant values 0.5 and 1 to the temporal mean of case 1 (green). In this test example, the average spatial correlation is close to zero. The spatial SL slope for case 1, 2 and 3 are b=1.28, 4.23, 2.66, and the spatial TL slopes are b=0.314, 3.47, 1.89 as seen in F Fig. We observed different SL exponent values because of the shifts to the mean values of the distributions for three cases 1,2 and 3, which eventually alter the SL and TL slopes, as seen in F Fig. Next, we detrend the spatio-temporal data, which sets temporal mean to zero in all the three cases. The time series of one location, the data distribution and the cumulative distribution are visualized in blue referred as case 4 in top row, in F Fig, the time series and distribution of the detrended data are plotted in bottom row first and second column, respectively, in F Fig. In the detrended cases, the spatial SL slope is found b=2 shown in bottom row third column in F Fig.

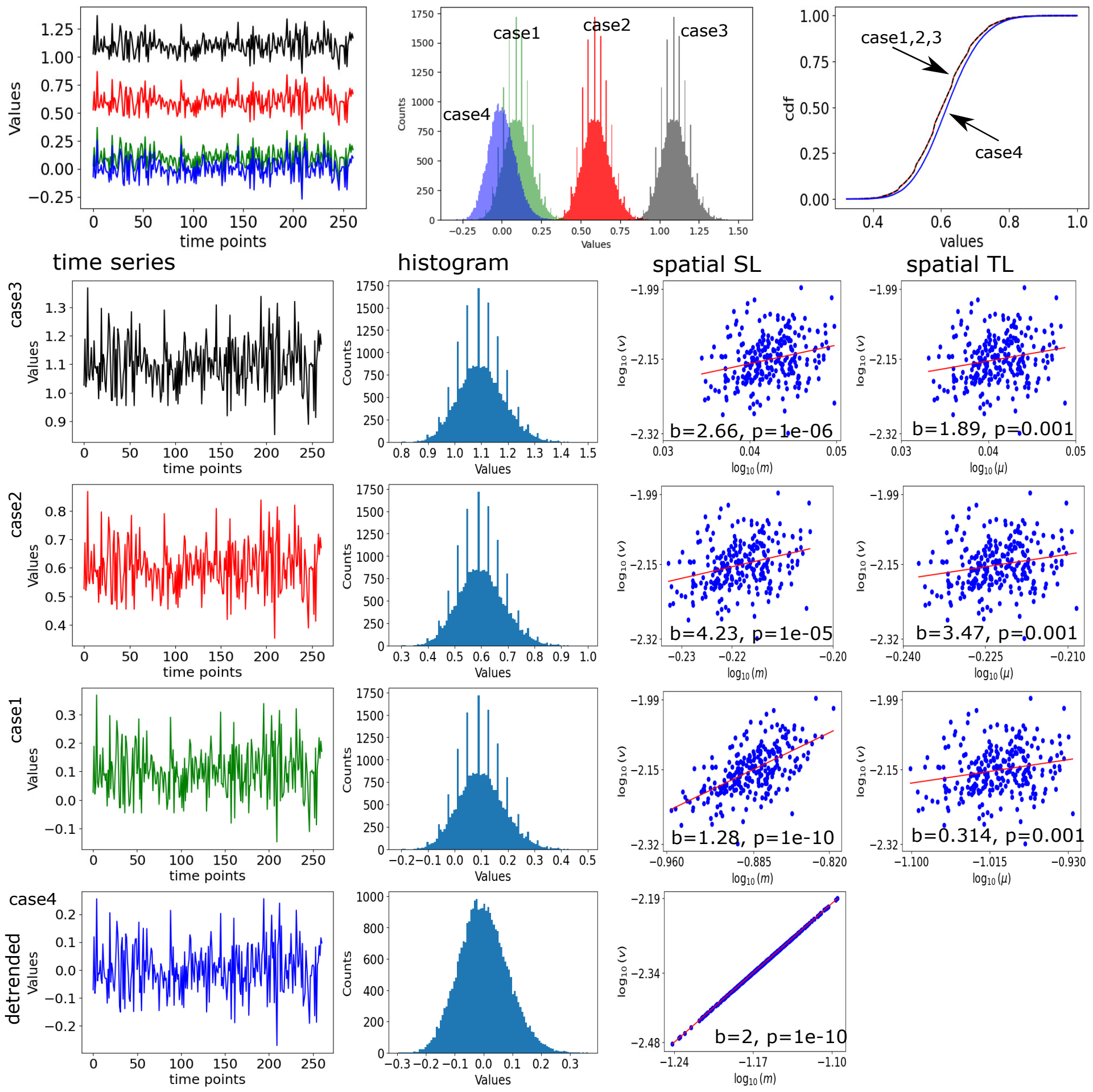

Fig F. Negative Binomial distribution: effect of shifting the temporal mean on the spatial SL slope. The Python function 'numpy.random.negative.binomial(n=100, p=0.5, size=(261, 116))' has been used to construct the time series, and normalized by the maximum amplitude of each location.

4.1.3 Independent and identically distributed data: Uniform distribution

G Fig shows numerical test on a spatio-temporal time series, size 261×116 (time points are 261 and number of locations are 116) has been generated by randomly drawing values from Uniform distribution. We first create a spatio-temporal data having the temporal mean at 0.5. The time series, the histogram and cumulative distributions are plotted in red marked as case 1. Next, we have shifted the mean of the entire data, where constant values 2 and 4 are added (temporal mean=2.5 for case 2 in black, and temporal mean =4.5 for case 3 in green) to the temporal of the original data (red) while freezing the variance and auto-correlation. Different random seeds are selected to construct time series for each locations. In all test cases we have considered an average correlation, between any pair of locations, close to zero, i.e., identical and independently distributed through time at each location.

We have plotted single time series out of 116 locations for three different cases 1, 2, 3 in red, black and green, respectively. Time series, histogram plot of the data distribution and cumulative distributions are shown in top row in G Fig, respectively, arranged from left to right order. All the three cases 1, 2 and 3 are again plotted separately in fourth, third and second rows, respectively in G Fig. The spatial SL slope for case 1,2 and 3 are b=0.478, 0.601, 0.793, and the spatial TL slopes are b=0.056, 0.25, 0.445. The SL and TL exponents are not significantly emerged in these three cases.

After detrending, the temporal mean is set to zero in all three cases. Thereafter, the saptio-temporal time series no longer follow Uniform distribution. The time series, histogram of the data distribution and cumulative distribution are visualized in blue referred as case 4 in top row in G Fig, and also separately plotted in bottom row. After detrending, the data tends to follow normal distribution, as seen in the cumulative distribution plot in blue for case 4. The spatial SL slope is found to be b=2 shown in the bottom row in G Fig.

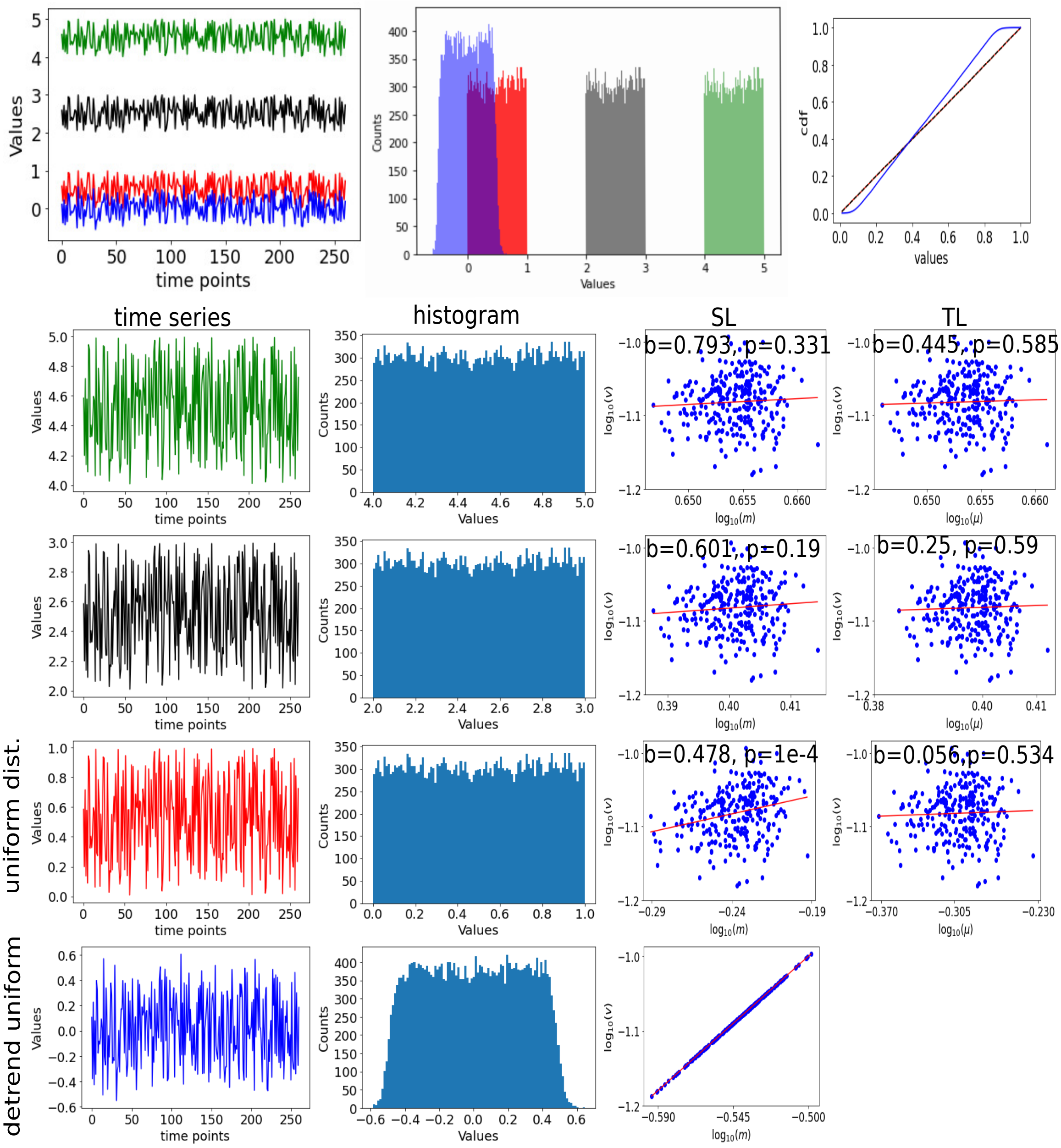

Fig G. Uniform distribution: effect of shifting the temporal mean on the spatial SL slope. We have used Python built-in function 'random.uniform(0,1,[261,116])' to create the spatio-temporal data following uniform distribution, where pair-wise correlation between two locations are near zero. The temporal mean is 0.5 labeled as red. The constant values 2 and 4 are added to shift the temporal of the entire time series and generate two cases 2 and 3, corresponding colors are black and green. Detrended case is shown in blue in bottom row.

4.1.4 Independent and identically distributed multivariate data: Normal distribution

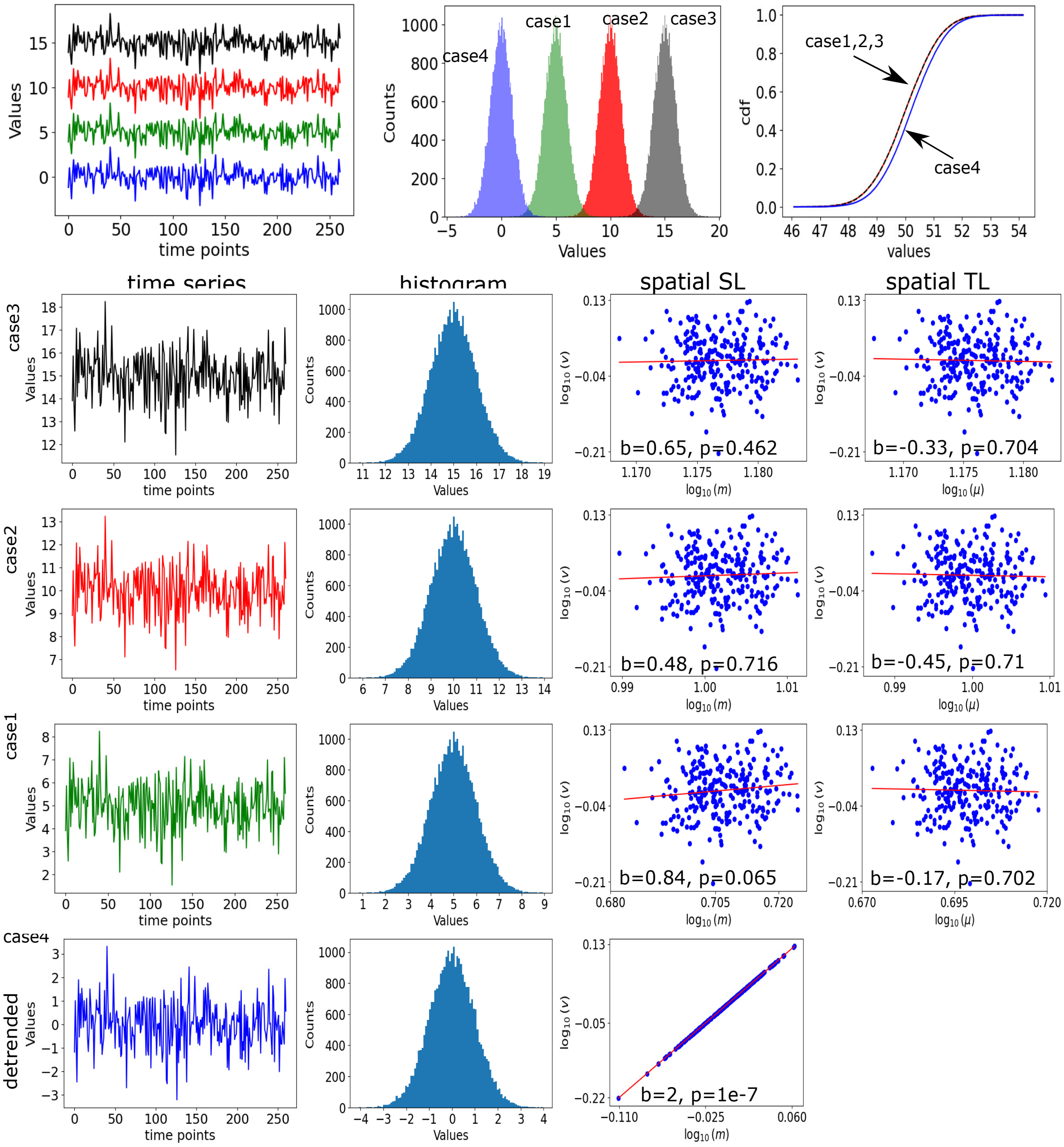

Fig H. Normal distribution: effect of shifting the temporal mean on the spatial SL slope. These figures are generated by Python. The normally distributed spatio-temporal time series data was constructed using the built-in function "numpy.random.normal". The algorithm is written in Algo. 4. The spatio-temporal time series in four cases are generated by changing the mean value in the Python function, as temporal mean=0, 5, 10, 15 (blue, green, red, black) shown in fifth, fourth, third and second rows, respectively.

4.2 Summary: Independent and identically distributed data

After detrending operation, we have observed that the data tends to follow normal distribution around zero mean. We have now conducted numerical tests on the spatio-temporal time series data constructed by randomly drawn values from a normal distribution. In the independent and identically distributed multivariate data (temporal mean $\mu = 0$, and variance=1), we calculated the spatial RMS and spatial variance v and presented in I Fig. We considered Gaussian normal distribution to generate multivariate time series data of size, Time $\times$ locations (261 x 116). Ia Fig shows histogram of the data distribution. Log-normal distributions of the spatial RMS and variance are shown in Ib and Ic Fig, respectively. Id Fig shows exponent of the log-log fit between spatial RMS and variance, found to be b=1.988 (using analytical expression, b=2.002). Ie Fig shows that the exponents are estimated setting different random seeds for 645 independent observations. We generate total 645 spatio-temporal data to check the association between synchrony (Ω) and the exponent (b), when the pair-wise correlation between any two locations are near zero. We observe that synchrony does not influence the scaling exponent in independent and identically distributed normal distributions. The Sl exponent close to $b = 2$, is emerged, only when the temporal mean is set to zero and the average spatial synchrony is very weak. Irrespective of the original distribution in the multivariate data, after detrending the spatial SL exponent is always found near 2, when synchrony is very weak and underlying mechanism of origin is a random process. We consider the normal distribution as a null model, and is responsible for data marginal contribution for random processes in the detrended empirical data. Under this background, we assume that if the neural activity is driven by the physical properties of given external stimuli, or spontaneous activity driven by internal stimuli, the scaling law may be influenced by synchrony differently in different brain states, rather than impacted by data marginals' distribution structures. Thus, we can separate the two effects from our measurements.

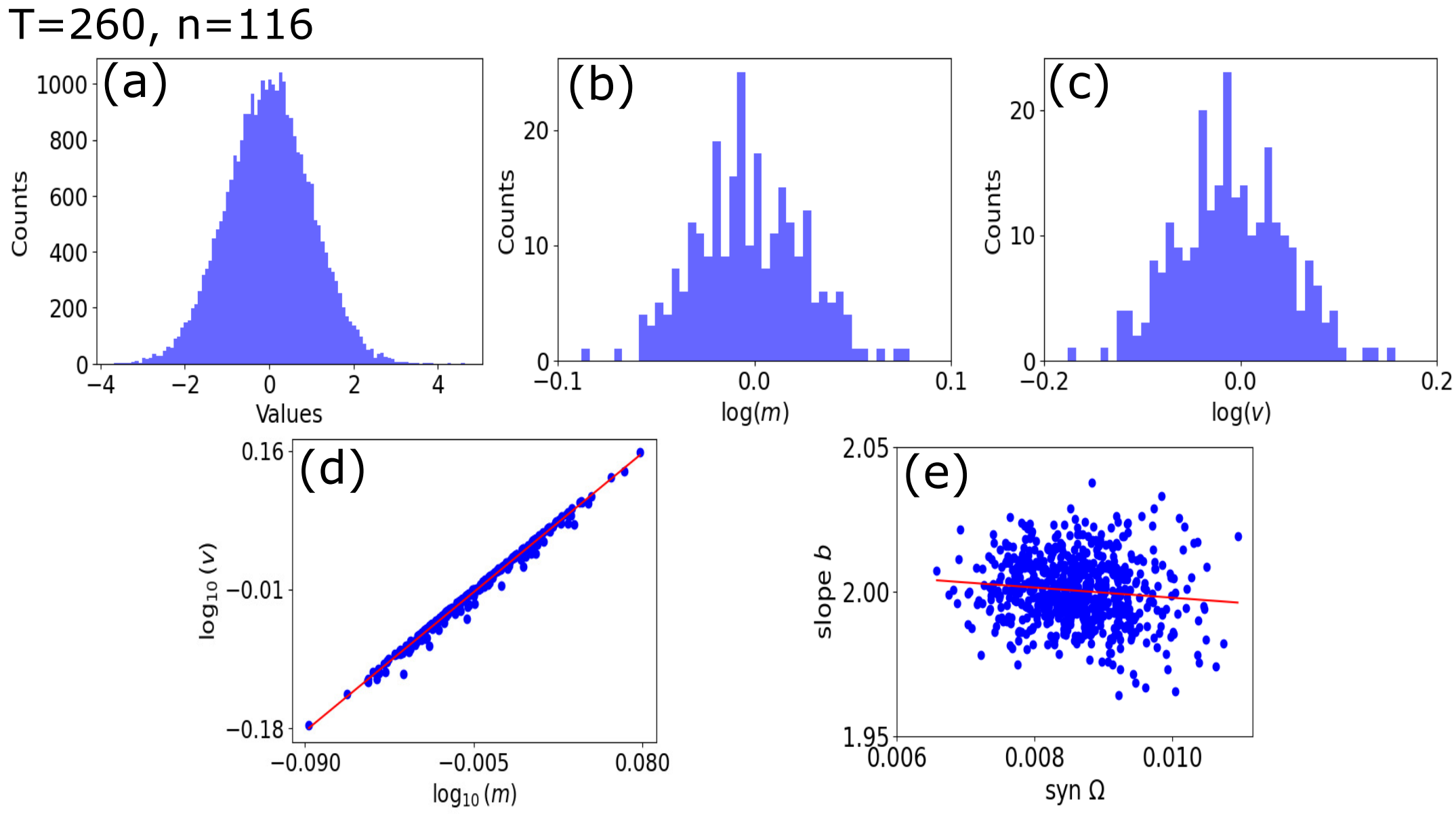

Fig I. Spatial SL–average synchrony (Ω) association for independent and identically distributed normal data. (a-d) We used algorithm shown in Algorithm 4 and Python functions to create a spatio-temporal time series data with 261 time pints and 116 locations. (e) Total 645 realizations are made to construct iid data, where location-wise correlation is nearly zero. The SL slope is found nearly around 2 for the weak synchrony level.

4.3 Identically distributed, but not necessarily independent

We have followed similar steps and mathematical expression proposed by DC Reuman et al. [2], using the well-known "common elements" method [13] of constructing a correlation-based multivariate random variable $(Y_1, \cdots, Y_n)$ such that the Y_i are identically distributed according to some specified distribution, and such that $cor(Y_i, Y_j) = \rho$ for $i \neq j$ for Poisson, Negative Binomial, Gamma and Normal distributions.

4.3.1 Poisson distribution

For identically distributed time series data Y_i, drawn from Poisson distribution, using the common element method are presented here. Assume, a single time series variable X added to the spatio-temporal time series X_i to construct the random variable Y_i. The statistical properties of the data X and X_i are modified by the average correlation ρ, as shown bellow.

$$E(X) = \text{var}(X) = \lambda\rho \tag{S39}$$

$$E(X_i) = \text{var}(X_i) = \lambda(1 - \rho) \tag{S40}$$

Defining $Y_i = X + X_i$, it is well-known that Y_i is Poisson distributed, because the summands are independent, and $E(Y_i) = E(X) + E(X_i) = \lambda\rho + \lambda(1 - \rho) = \lambda$ as desired. Also, for $i \neq j$,

$$corr(Y_i, Y_j) = \frac{cov(Y_i, Y_j)}{\lambda} = \frac{cov(X + X_i, X + X_j)}{\lambda} = \frac{E(X)}{\lambda} = \rho. \tag{S41}$$

For more detailed descriptions and analytical derivations, please refer to Ref. [2]. Simulation results are shown in J Fig. We derived synchrony using Eq. (S15), found to be almost similar to the ρ value, and the statistical properties are numerically calculated, and then put to the Eq. (S31) to derive the spatial SL exponent b. We have stored both the fitted slope and semi-analytical exponent values for different average synchrony values.

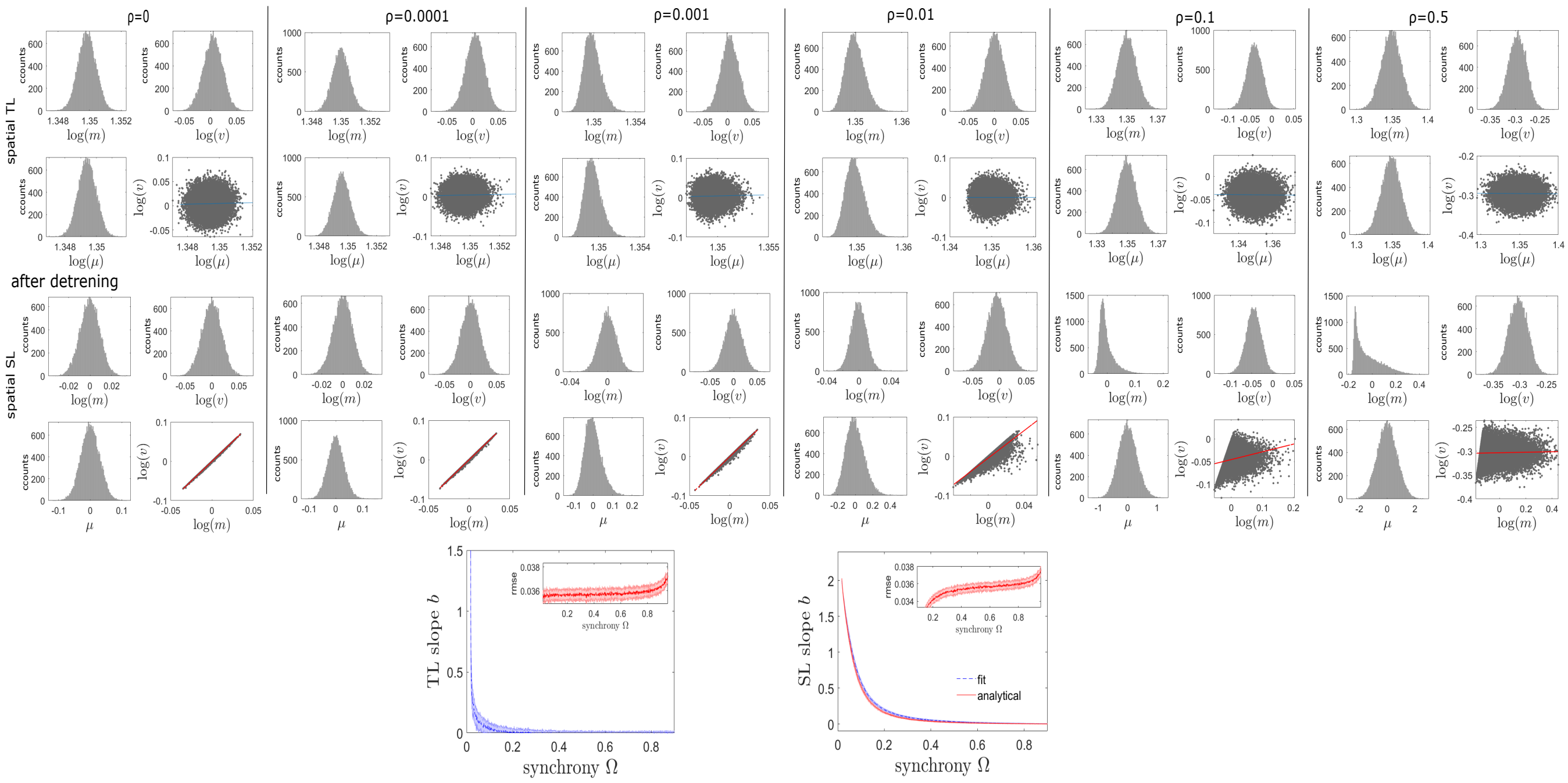

Fig J. Effect of average synchrony on the spatial SL slope for Poisson data (identically distributed, but not necessarily independent). Influence of synchrony on the spatial SL slope in synthetically generated multivariate random variable $(Y_1, \cdots, Y_n)$ has been tested. The random variable Y_i are identically distributed but not necessarily independent, constructed by randomly drawn values for a given Poisson distribution ($\lambda = 50$), using common element method [13], while controlling the correlation between Y_i and Y_j for all $i \neq j$, with the parameter, ρ. The inset shows RMSE, calculated using Eq. (S38). After detrending, the results are produced to visualize the spatial SL for various ρ values and also the spatial SL exponent against the synchrony are shown. As the average correlation increased from zero, the Sl exponent b almost always decreased quite sharply from 2.

4.3.2 Negative Binomial distribution

We employed similar strategy to generate multivariate data drawn from negative binomial distributions, NB(r, p), where p denotes the probability of a success or of a failure, and r represents success or failure. Let X be a time series drawn from negative binomial distribution with parameters $r_X = \rho r$ and p, and let the X_i be independent (with respect to each other and X) negative binomials with parameters $r_i = r - r_X = r(1-\rho)$ and p. Defining $Y_i = X + X_i$, it is known that Y_i is negative binomial with parameters $r = r_X + r_i$ and p, as desired; it is also easy to show, since the variance of a negative binomial with parameters r and p is $r(1-p)/p^2$, that $\mathrm{corr}(Y_i, Y_j) = \rho$, as desired. Simulating draws from $(Y_1, \ldots, Y_n)$ is straightforward and computationally efficient. Standard formulas for negative binomial moments provide, $M = r(1-p)/p$, and $V = r(1-p)/p^2$, for other moments and detailed analytical derivations refer to Ref. [2].

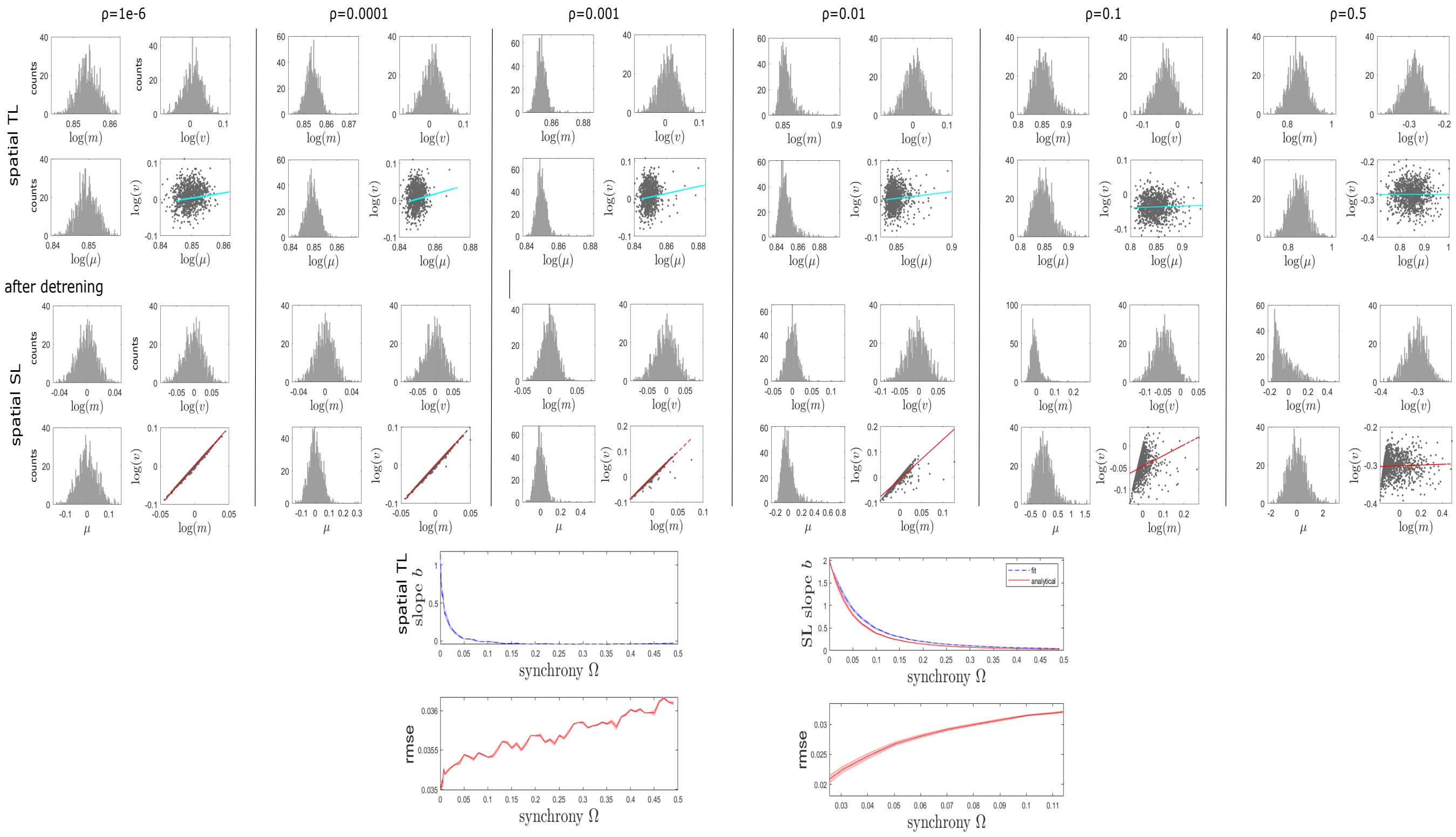

Fig K. Effect of average synchrony on the spatial SL slope for negative binomial data (identically distributed, but not necessarily independent). Influence of synchrony on the spatial SL slope in synthetically generated multivariate random variable $(Y_1, \cdots, Y_n)$ has been tested. The random variable Y_i are identically distributed but not necessarily independent, constructed by randomly drawn values for a given negative binomial distribution and used common element method [13] to create ρ dependent spatio-temporal data, where $\mathrm{corr}(Y_i, Y_j) = \rho$ for all $i \neq j$. The inset shows RMSE, calculated using Eq. (S38). After detrending, the results are produced to visualize the spatial SL for various ρ values and also the spatial SL exponent against the synchrony are shown. As the average correlation increased from zero, the Sl exponent b almost always decreased quite sharply from 2.

4.3.3 Gamma Distribution

The same analytic strategy was applied to the gamma distribution $\Gamma(\alpha, \beta)$, where α is shape parameter and rate parameter is β. Now, let ρ is the induced correlation, then assume X follows gamma distributed with shape and rate parameters $\rho\alpha$, β. The X_i is independent to each other and with X, and is drawn from gamma distribution with parameters $\alpha(1-\rho)$ and β. It is known that $Y_i = X + X_i$ is gamma distributed, with shape parameter equal to the sum of the shape parameters of X and X_i, α and rate parameter equal to the common rate parameter for the summands, β. A straightforward check can give corr(Y_i, Y_j)=ρ for $i \neq j$, as desired. Simulating draws from $(Y_1, \ldots, Y_n)$ is again straightforward and computationally efficient. The first two moments were from standard formulas or were computed by inserting $Y_i = X + X_i$ into the moment definitions and computing: $M = \alpha/\beta$, and $V = \alpha/\beta^2$.

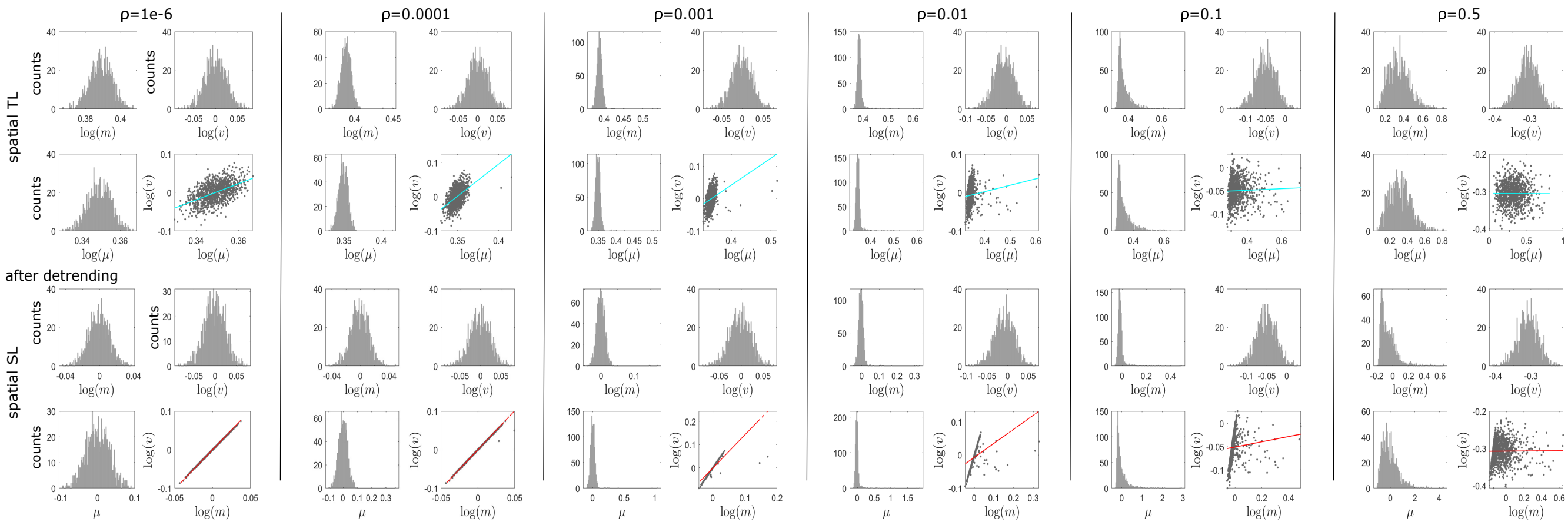

Fig L. Effect of average synchrony on the spatial SL slope for Gamma distribution (identically distributed, but not necessarily independent). Variation in synchrony-induced SL exponents for different ρ in multivariate spatio-temporal time series data.

4.3.4 Normal Distribution

The same analytic strategy was applied to the normal distribution $N(\mu, \sigma)$, where μ is mean and σ is standard deviation. Now, let ρ is the induced correlation, then assume X is single time series dependent on ρ, which follows normal distributed, and let X_i is independent to each other and with X. X_i is drawn from normal distribution with dependency on the parameters $(1-\rho)$. It is known that $Y_i = X + X_i$ is normal distributed. A straightforward numerical check can give corr(Y_i, Y_j)=ρ for $i \neq j$, as desired. Simulating draws from $(Y_1, \ldots, Y_n)$ is again straightforward and computationally efficient.

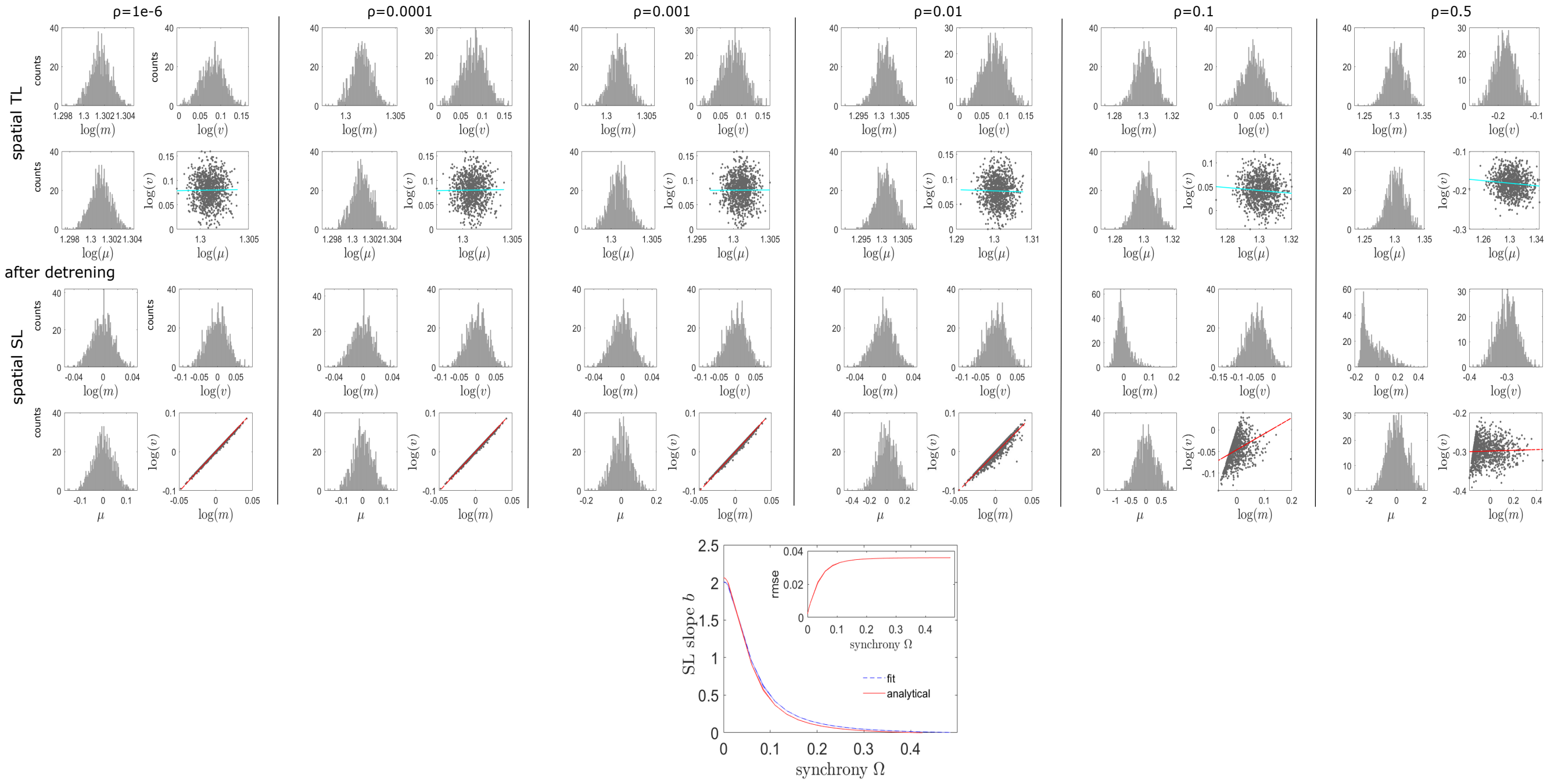

Fig M. Effect of average synchrony on the spatial SL slope for normal distribution (identically distributed, but not necessarily independent). Variation in synchrony-induced SL exponents for different ρ values (where, corr(Y_i, Y_j)=ρ) in spatio-temporal time series data, normally distributed. We have shifted the mean values above zero for the cases of TL derivations and then, either detrended the data or generated it with zero mean using algorithm shown in Algo. 5.

5 Algorithm to construct multivariate time series

5.1 Construct spatio-temporal time series: identically independent distributions

We used MATLAB default random generator, Mersenne 'twister' algorithm with 'mt19937ar' generator keyword and variable seeds for different observations in the three test cases, binomial, uniform and Gaussian normal distributions.

5.1.1 Poisson distribution

Input : $observations, n, T, \lambda$
Output: ts_uni
$observations \leftarrow 645$ % total number of observation
$\lambda \leftarrow 5$ % the expectation of λ events in a given interval
$n \leftarrow 1160$ % number of time series in a single observation
$T \leftarrow 2610$ % total time points
$seed \leftarrow$ linspace(100, 700, $observations$)
$ts\_bion \leftarrow$ cell array of size $(observations, 1)$
for $k \leftarrow 1$ **to** $observations$ **do**
  $r\_array \leftarrow random.poisson(\lambda, size = (T, n))r_array.append(r_array2)$
  $ts\_bion\{k\} \leftarrow r\_array/max(max(r\_array))$
end

Algorithm 1: Test time series have been generated by drawing values from Poisson distribution

5.1.2 Binomial distribution

Input : $observations, n, T$
Output: ts_uni
$observations \leftarrow 645$ % total number of observation
$n \leftarrow 116$ % number of time series in a single observation
$T \leftarrow 261$ % total time points
$seed \leftarrow$ linspace(100, 700, $observations$)
$ts\_bion \leftarrow$ cell array of size $(observations, 1)$
for $k \leftarrow 1$ **to** $observations$ **do**
  $r\_array \leftarrow$ `binornd(ceil(`$seed(k)$`),0.2,`T`,`n`)`
  $ts\_bion\{k\} \leftarrow r\_array/max(max(r\_array))$
end

Algorithm 2: Test time series have been generated by drawing values from Binomial distribution

5.1.3 Uniform distribution

Input : $observations, n, T$
Output: ts_uni
$observations \leftarrow 645$ % total number of observation
$n \leftarrow 116$ % number of time series in a single observation
$T \leftarrow 261$ % total time points
$a \leftarrow 0$, $b \leftarrow 1$
$seed \leftarrow$ linspace(100, 700, $observations$)
$ts\_uni \leftarrow$ cell array of size $(observations, 1)$
for $k \leftarrow 1$ **to** $observations$ **do**
  `rng(ceil(`$seed(k)$`))`
  $ts\_uni\{k\} \leftarrow a + (b - a) *$ `rand`(T, n)
end

Algorithm 3: Time series generated from uniform distribution

5.1.4 Normal distribution

Input : $observations, n, T, \mu$
Output: ts_norm
$observations \leftarrow 645$ % total number of observation
$\mu \leftarrow 0$ % normal distribution with various mean values e.g., 0, 5, 10, 15
$n \leftarrow 1160$ % number of time series in a single observation
$T \leftarrow 2610$ % total time points
$ts\_norm \leftarrow$ cell array of size $(observations, 1)$
for $k \leftarrow 1$ **to** $observations$ **do**
    `random.seed(k)`
    $r\_array \leftarrow$ random.normal$(\mu, \text{size} = (T, n))$
    $ts\_norm\{k\} \leftarrow r\_array$
end

Algorithm 4: Time series generated (iid) from normal distributions in Python

5.1.5 Construct spatio-temporal time series: identically distributed, but not necessarily independent

Input : n, T, μ, ρ
Output: Y_norm
$\mu \leftarrow 0$ % defines mean
$n \leftarrow 116$ % number of locations
$T \leftarrow 261$ % total time points
$X = mvnrnd([\mu\ \mu, \rho * [1\ 0;\ 0\ 1], T)$
for $i \leftarrow 1$ **to** n **do**
    `seed(i)`
    $Xi \leftarrow$ mvnrnd$([\mu\ \mu], (1 - \rho) * [1\ 0;\ 0\ 1], T)$
    $Y\_norm\{i\} \leftarrow X + Xi$
end

Algorithm 5: The spatio-temporal time series is generated from normal distributions, using common element method.

6 Empirical data analysis using CamCAN dataset

6.1 Data sources and participants

We have used the data from http://www.mrc-cbu.cam.ac.uk/datasets/camcan. The data were collected as part of stage 2 of the Cambridge Centre for Aging and Neuroscience (CamCAN) project [14, 15]. The fMRI data in resting state (rest), naturalistic movie watching (mov) and sensorimotor task (smt) from 645 healthy participants were used in our study. The data is made publicly available and can be downloaded from their website.

6.2 Data preprocessing

The fMRI data for each functional run (resting state, movie watching, and SMT) were unwrapped using fieldmap images, realigned to correct for motion and, slicetime was corrected. EPI data were coregistered to the T1-weighted image, transformed to montreal neurological institute (MNI) space using the warps and affine transformation from structural image (estimated using DARTEL). For region of interest (ROI) analysis, mean regional BOLD time series were estimated in 116 parcellated brain areas using Anatomical Automatic Labelling atlas (AAL) [8] (http://www.gin.cnrs.fr/tools/aal). Preprocessed data were provided by CamCAN research consortium. Details of preprocessing pipeline can be found in [14].

For group-level analysis, we divided the data of 645 participants into three cohorts, young adults (YA) age range 18-36 years, middle-aged adults (MA) age 46-60 years, and older adults (OA) age 72-88 years. Each participant's BOLD time series in the resting state, naturalistic movie watching, and SMTs were used. We did region-wise detrending for each subject, resulted in zero 'temporal mean' [2, 16], i.e., the fluctuation of the regional time series is around zero, which also implies that the signals contain both positive and negative values.

6.3 Surrogate time series: phase-randomization

We randomized phases of the empirical BOLD series to produce surrogated data utilized to examine marginal distribution's contribution to measurement. Given a time series $Y(t)$, the complex valued Fourier transform can be written as,

$$Y(f) = A(f)e^{i\phi(f)}.$$

$A(f)$ and $\phi(f)$ are amplitude and phase, respectively. We considered a univariate phase randomization using fast Fourier-transform surrogate data [17]. A 'phase-randomized' Fourier transform $\tilde{Y}(f)$ is made by randomly rotating the phase ϕ at each frequency f by an independent random variable φ which is chosen from uniform distribution in the range $[0,\pi]$. The φ's are not completely independent, but instead are constrained to satisfy the symmetry property $\varphi(f){=}{-}\varphi(-f)$ to insure that the inverse Fourier transform is a time series of real values. That is,

$$\tilde{Y}(f) = A(f)e^{i\varphi(f)}.$$

From this surrogate time series is given by the inverse Fourier transform by,

$$\tilde{Y}(t) = \mathcal{F}^{-1}\{\tilde{Y}(f)\} = \mathcal{F}^{-1}\{A(f)e^{i\varphi(f)}\}.$$

By construction, $\tilde{Y}(t)$ will have the same power spectrum as the original data set $Y(t)$, and by the Wiener-Khintchine theorem the same autocorrelation function, i.e., the circular autocorrelation is preserved [17], when $Y(t)$ has a zero temporal mean.

6.4 Empirical Results

Neural encoding process [18] involves changes in firing rates and timing of neural population within certain brain region, and in synchrony, an emerged co-activation pattern across and within few regions in response to internal or external stimuli. Conventional studies leave answers to many important questions unclear, e.g., how accurately and efficiently can a mental state be inferred? We argued that if the process of neural encoding does indeed translate physical properties of a stimulus into patterns of brain activity

Table A. Functional MRI scans.

Scan type	Sequence	TR (ms)	TE (ms)	Flip angle (°)	FOV (mm)	Voxel Size (mm)	Volumes (N)	Slices (N)	Slice thickness (mm)	Gap (%)	Order	Task
Resting state	EPI	1970	30	78	192 × 192	3 × 3 × 4.44	261	32	3.7	20	Descending	Rest with eyes closed
Movie watching	multi-echo EPI	2470	9.4, 21.2 33, 45, 57	78	192 × 192	3 × 3 × 4.44	193	32	3.7	20	Descending	Watch and listen to movie
Sensorimotor task	EPI	1970	30	78	192 × 192	3 × 3 × 4.44	261	32	3.7	20	Descending	Audio-visual stimuli and manual response

TR = repetition time; TE = echo time; FOV = field of view; EPI = T2*-weighted gradient echo planar image.

that represents stimulus characteristics, then the signature can be extracted from the attributes of spatial fluctuation scaling of a time series.

Decoding a person's current mental state by learning to recognize characteristic spatial patterns of brain activity associated with different mental states takes into account not just activity at single locations but the full spatial pattern of activity [18]. Such decoding method reveals that substantially more information is encoded in fMRI signals [18]. Three major constrains may mask non-linearity in the time series and can cause measurement error. For example, a recent paper argued in favor of linearity in the data, showing how macroscopic resting-state brain dynamics are best described by linear models [19]. They mentioned that although more than a few (around 100-10^4) neurons are required, spatial averaging still dissolves the nonlinear aspects of the dynamics while mostly sparing the linear ones [19]. Secondly, there is an inevitably large time-scale separation between the observed slow varying blood oxygen level dependent activity (0.001Hz-0.1Hz) and underlying neural activity at different spatial brain scale in response to a given stimulus or spontaneous activity. The BOLD activity resulted from much faster neuronal activity at the microscopic level (e.g., single neuron action-potential), or mesoscopic level activity, averaging over an ensemble of neuronal population displaying higher frequency fluctuations ($\sim$4Hz). Finally, microscopic nonlinear dynamics can be counteracted or masked by four factors associated with macroscopic dynamics: averaging over space and over time, which are inherent to aggregated macroscopic brain activity, observation noise and limited data sample, which stem from technological limitations [19].

The conventional approaches have proven successful in elucidating many aspects of the relationship between cognitive and mental states, and brain activity. However, recent advances in data analysis procedures raise the possibility of deciphering additional and complementary information from neuroimaging data [18]. Under this background, we envisaged a scale invariant linear relationship as a quantitative measure of spatial aggregation, while masking the effects from temporal mean and observation noise. In contrast to the strictly single location-based conventional analyses, such as temporal TL [16] or temporal autocorrelation [20], we have utilized spatial scaling law. The spatial SL holding statistical property of a multivariate time series is defined by the linear relationship when the spatial variance is approximately a power law scaling of spatial RMS upon a logarithmic transformation. Thus, the amplitudes of BOLD signals ($Y_i(t)$) in all the brain regions include both positive and negative values evolving around zero temporal-mean [16,21]. Thereby, our analysis is focused on the attributes from spatial properties of BOLD signals while suppressing contributions from temporal features.

The study proposes a revised spatial scaling law (SL) derived from spatial distribution of individual's detrended BOLD signals during different encoding processes for different brain states, including resting state, movie watching and sensorimotor task. Our primary aim is to find a feature less influenced by local temporal mean and data marginals. Next, we examine correlation between the spatial feature and synchrony, quantifying coordinated neural dynamics under different brain states, and finally, aging effect on the synchrony-scaling relationship.

6.5 Empirical data visualization

Empirical data distributions before and after detrending operation for three brain states (rest, movie and sensorimotor task) are shown.

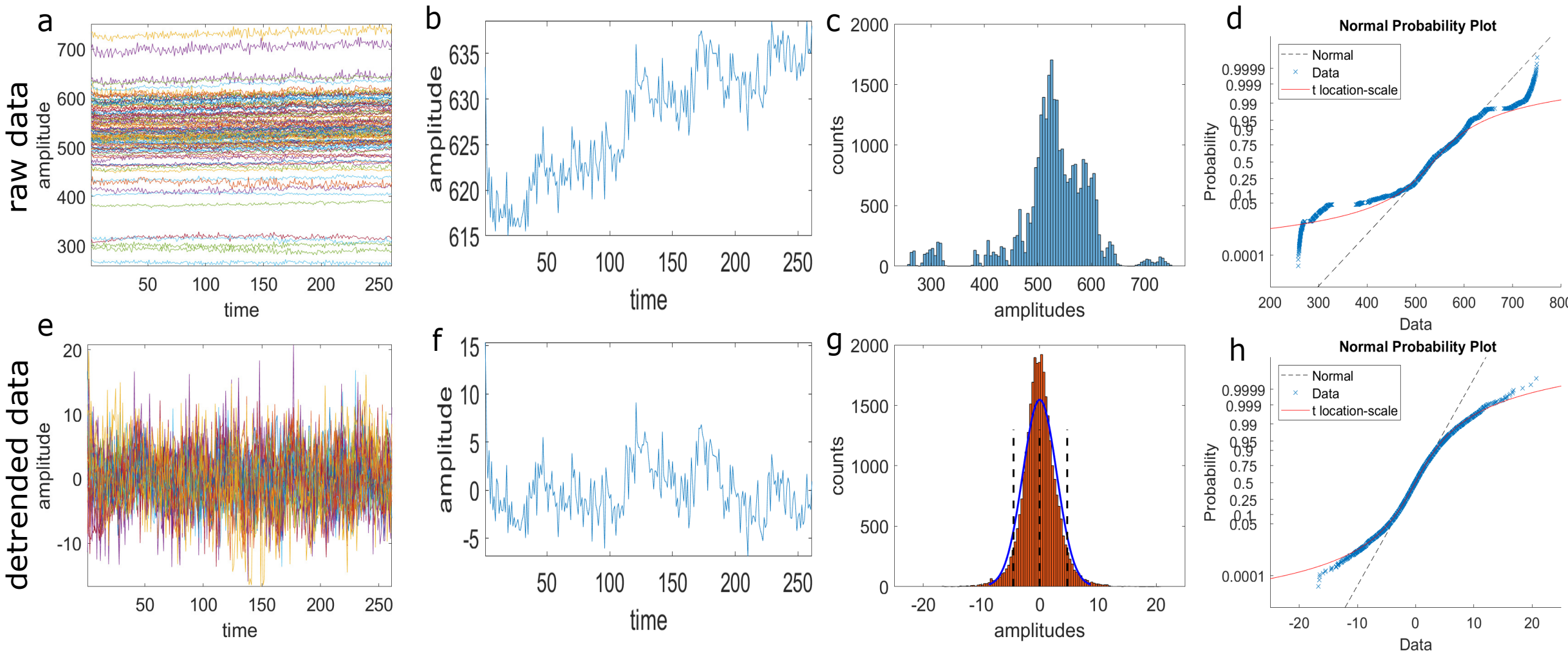

Fig N. Empirical data and detrending operation in resting state. (a-d) Raw data is shown. (a) Empirical BOLD signal of one participant. Data size is 261 × 116, brain regions = 116 (AAL atlas), time points = 261. (b) BOLD series of region-111. (c) Distribution of the raw data. (d) Probability plot of the raw data to test the normal distribution. Detrended data is shown in (e-f). (g) Detrended data distribution. Normal fit is shown in red line. (f) Probability plot of the normally distributed data after detrending operation.

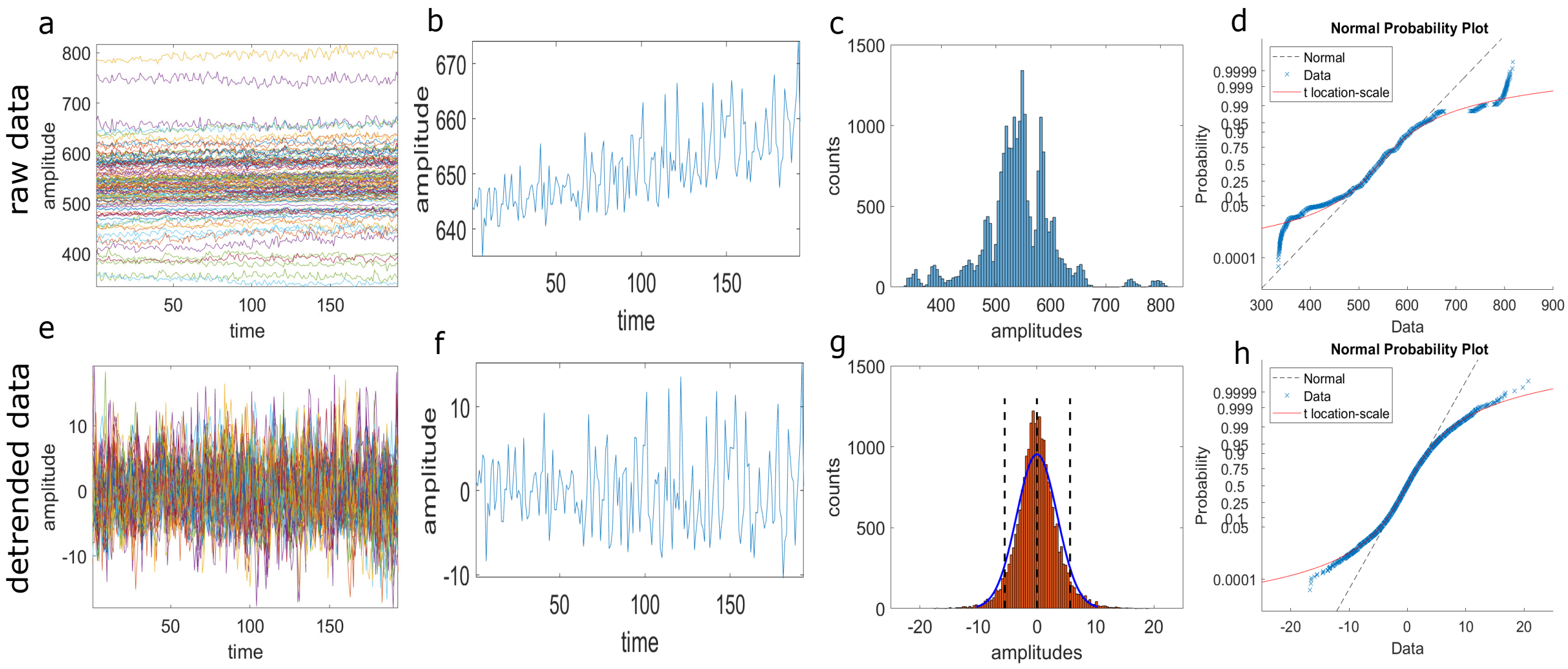

Fig O. Empirical data and detrending operation in movie watching task. (a) Empirical BOLD series of one participant. Data size is 261 × 116; brain regions = 116 (AAL atlas), time points = 261. (b) BOLD signal of left cerebellum-10 (one location). (c) Histogram plot of the raw data. (d) Normal probability plot shows that neither the normal line (black dashed line) nor the location-scale curve (red) fits very well. After detrending, BOLD series of all the brain regions are shown in (e). (f) Suppressed trend of the BOLD signal of left cerebellum-10, compare (b). (g) Data distribution with normal fit in blue curve. The vertical black dashed lines indicate 95% CI. (h) Normal Probability plot of the detrended data shows that it fits well to the normal distribution for the data range within 95% CI.

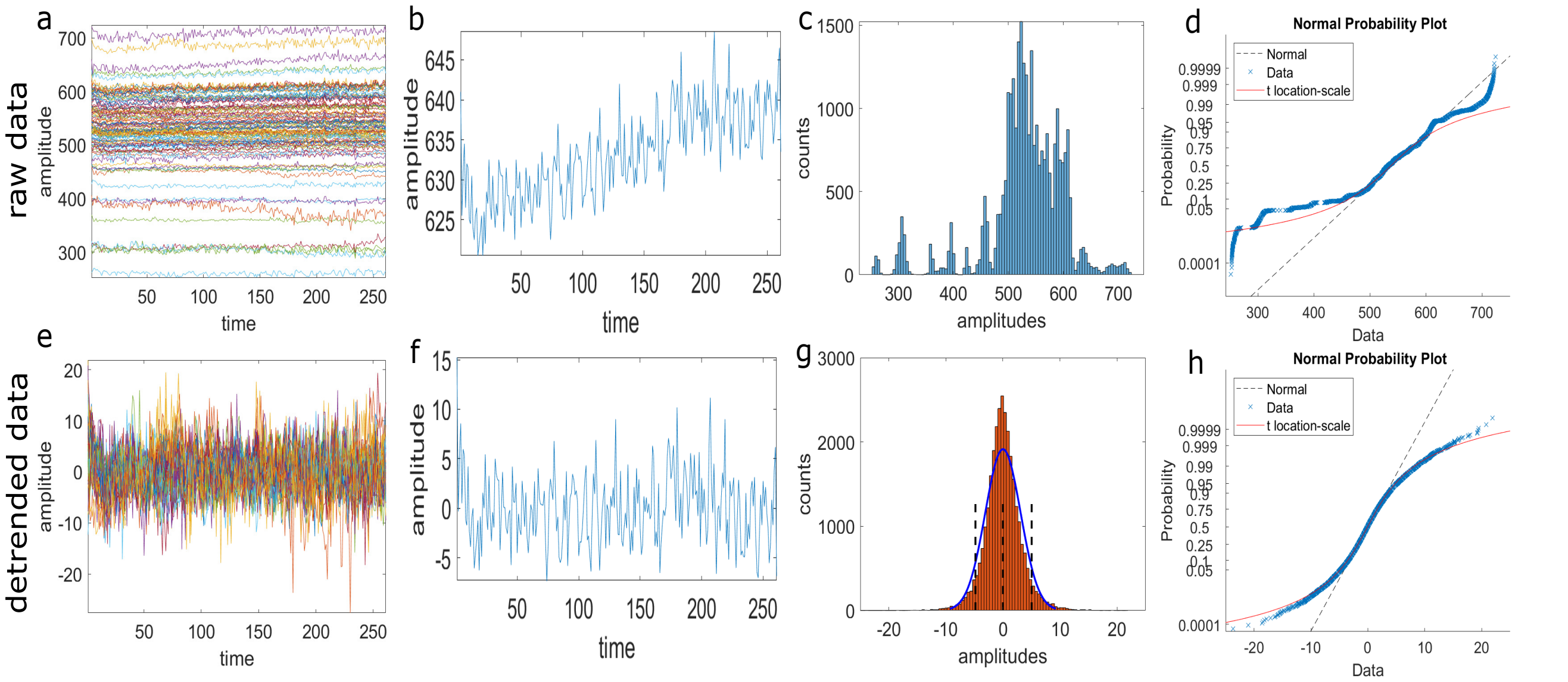

Fig P. Empirical data and detrending operation in sensorymotor task. (a) Empirical BOLD series of one participant. Data size is 261 × 116; brain regions = 116 (AAL atlas), time points = 261. (b) BOLD signal of left cerebellum-10 (one location). (c) Histogram plot of the raw data. (d) Normal probability plot shows that neither the normal line (black dashed line) nor the location-scale curve (red) fits very well. After detrending, BOLD series of all the brain regions are shown in (e). (f) Suppressed trend of the BOLD signal of left cerebellum-10, compare (b). (g) Data distribution with normal fit in blue curve. The vertical black dashed lines indicate 95% CI. (h) Normal Probability plot of the detrended data shows that it fits well to the normal distribution for the data range within 95% CI.

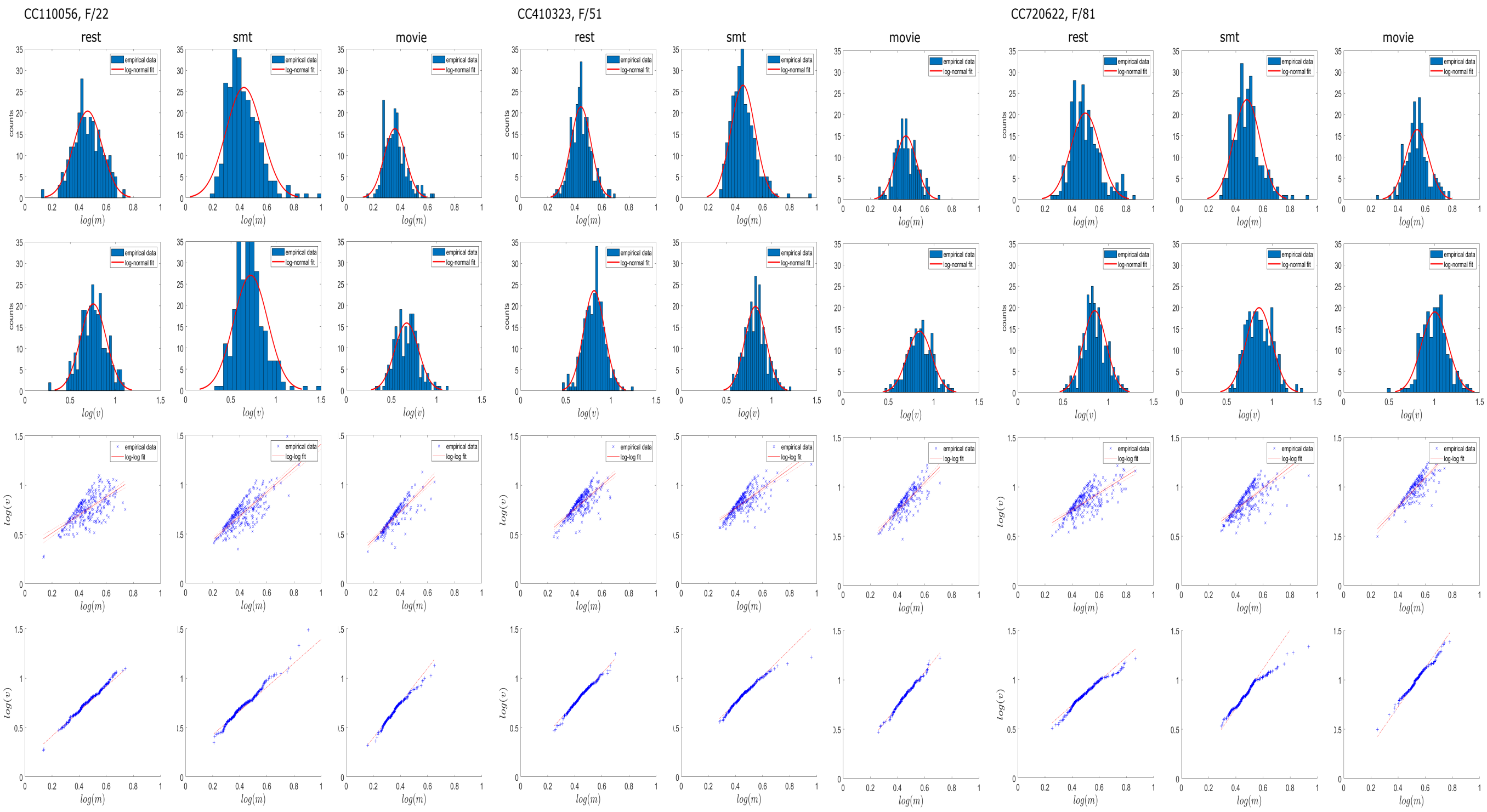

Fig Q. Spatial RMS, variance, and scaling law of three participants in three brain states. Functional MRI scans from three participants are chosen under three different brain states, such as rest, somatosensory task (smt), and movie. Top two rows show distributions of the spatial RMS and spatial variance with log normal fit. Third row from top shows a power law fitting between spatial RMS and variance on log-log scale. Red line indicates spatial SL exponent. Bottom row shows quantile-quantile plot.

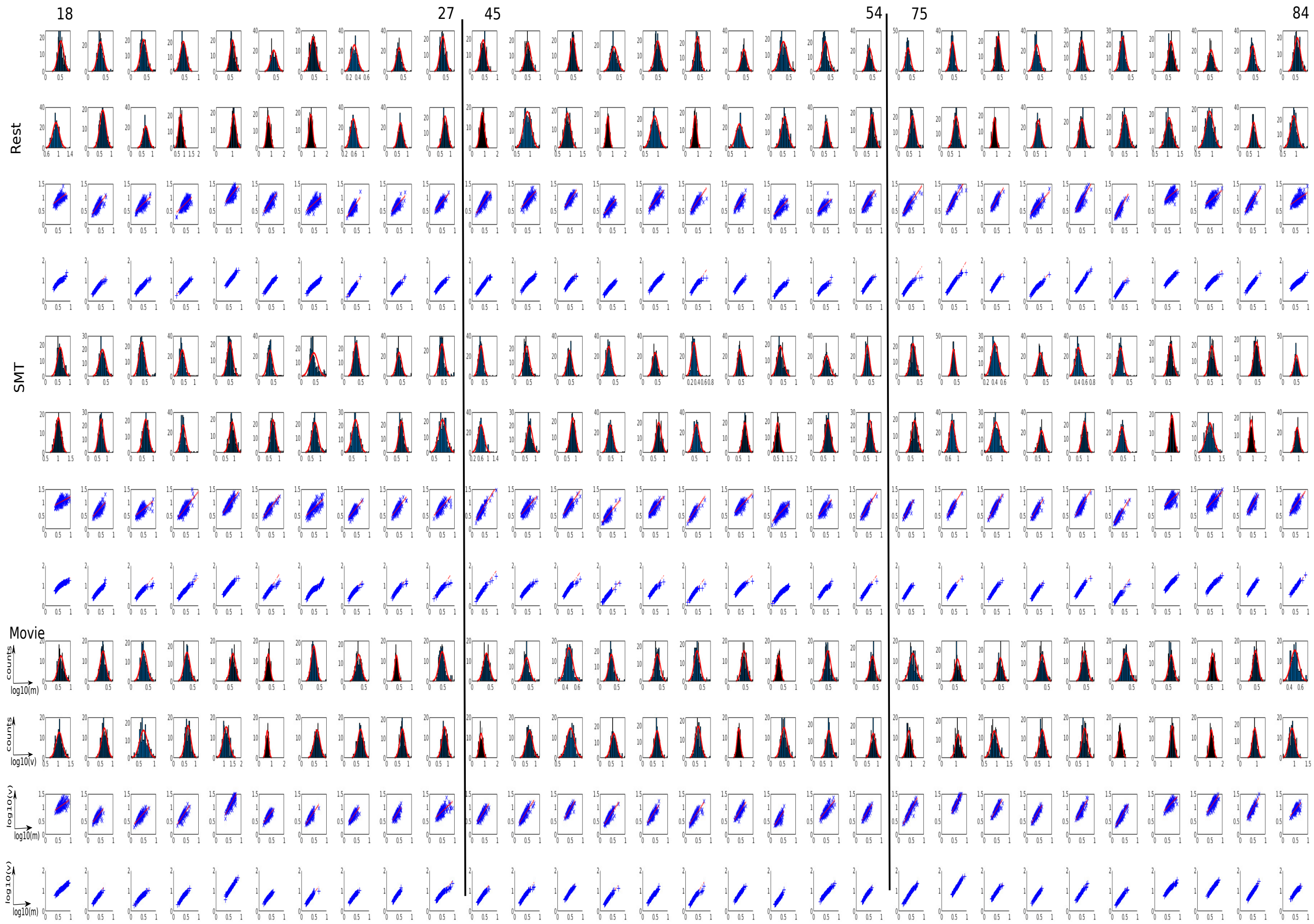

Fig R. Spatial RMS, variance, and SL of participants from young, middle-aged, and older groups in three brain states. Ten participants are chosen from young, middle-aged and elder groups for three brain states. For each brain state, there are four rows. Top two rows show the log-normal distributions of the spatial RMS $\log(m(t))$ and spatial variance $\log(v(t))$. Third row shows log-log scatter plot between spatial RMS and variance. The red line indicates spatial SL slope. Bottom row shows quantile-quantile plot.

From the numerical simulations it is evidenced that any given distribution after detrending starts following normal-like distribution, which is set as a null model underlying random process or as a data marginal when spatial scaling exponent is not driven by the coordinated activity (at a very weaker strength of synchrony the spatial scaling 2). This has been utilized to remove the contributions or the effects of randomness on the empirical observations and measurements, so that we can obtain results solely driven by the internal or external stimuli. Thereby, We are able to decompose the spatial SL exponent b into two parts: the SL driven by random process b_{marg}, and SL driven by the synchrony b_{syn}.

6.6 Task driven coordinated dynamics and spatial scaling law

We examine the influence of synchrony for both the resting state and active tasks using fMRI data from a large cross-section of adult lifespan (18-88 years old) subjects, N=645 participants, BOLD time series of size 261 time points×116 brain locations.

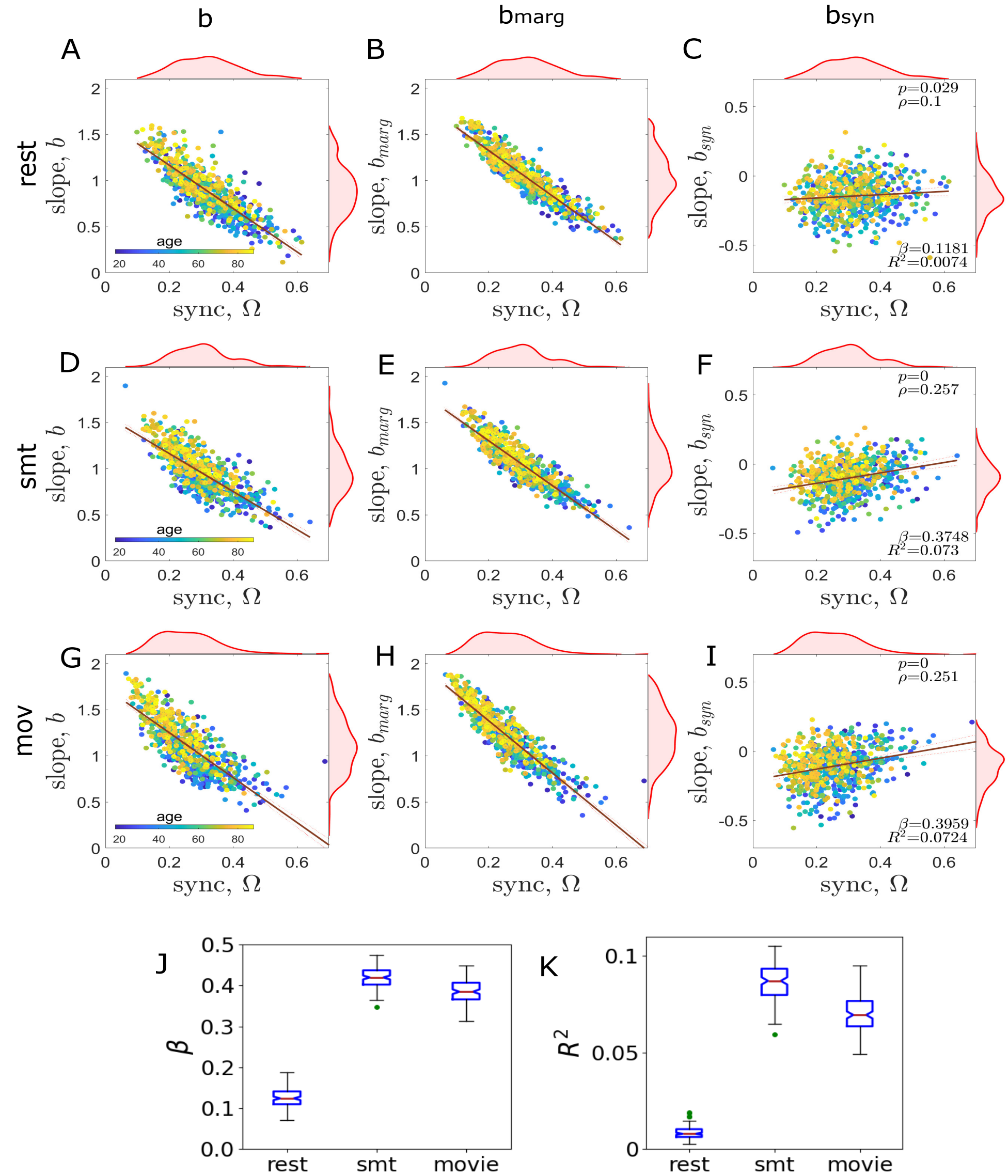

Fig S. Spatial SL exponent and average synchrony for the resting state, sensorimotor and movie watching task in the CamCAN aging cohort. Plots of the SL exponent b and synchrony Ω for resting state (A-C), movie watch (D-F) and sensorimotor task (G-I) in CamCAN aging cohorts. First column (A,D,G) shows the scatter plot between synchrony (Ω) and the SL exponent (b) measured from the empirical data. Second column (B,E,H) shows scatter plot between Ω and the slope b_{marg}, contributed by the data marginals (emerged from random process). The slope b_{marg} has been derived from the data constructed by randomizing the phases of empirical BOLD activity for three brain states. Third column (C,F,I) shows scatter plot fit between Ω and the slope b_{syn}, derived by subtracting slope b_{marg}, due to marginal distribution, from the original slope b, such that the synchrony induced SL slope can be clearly observed, when the contribution from the marginal distribution is separated from the measurement using $b_{syn}=b-b_{marg}$. (J) The degree of correlation is measured by the fitted slope (β) between Ω and b_{syn} for the three brain states, and corresponding R-squared values are shown in (K). We have measured the association slope (β) by phase randomization of the empirical data for 1000 independent realizations.

6.7 Aging effects on synchrony and spatial SL in three brain states

We examine the effect of healthy aging on the spatial scaling. The SL slope is plotted against age as a continuous independent variable in T Fig; the linear regression fitting is shown by the red straight lines; blue curves show the slope by averaging over a window of five years. The synchrony-driven exponent b_{syn} increases (middle column) with increasing age in all three brain states. In a resting state, aging has no significant effect on the slope b_{syn}, whereas the aging trajectory shows a significant increase in the slope b_{syn} against age for *smt* and *mov* cases. In other words, aging effects are prominently visible in the movie-watching and sensory-motor tasks but not in the resting state (rest). The third column in T Fig shows a significant decrease in synchrony across the lifespan trajectory in all three brain states. The error bars (grey shades) are obtained for a number of participants present in each age value.

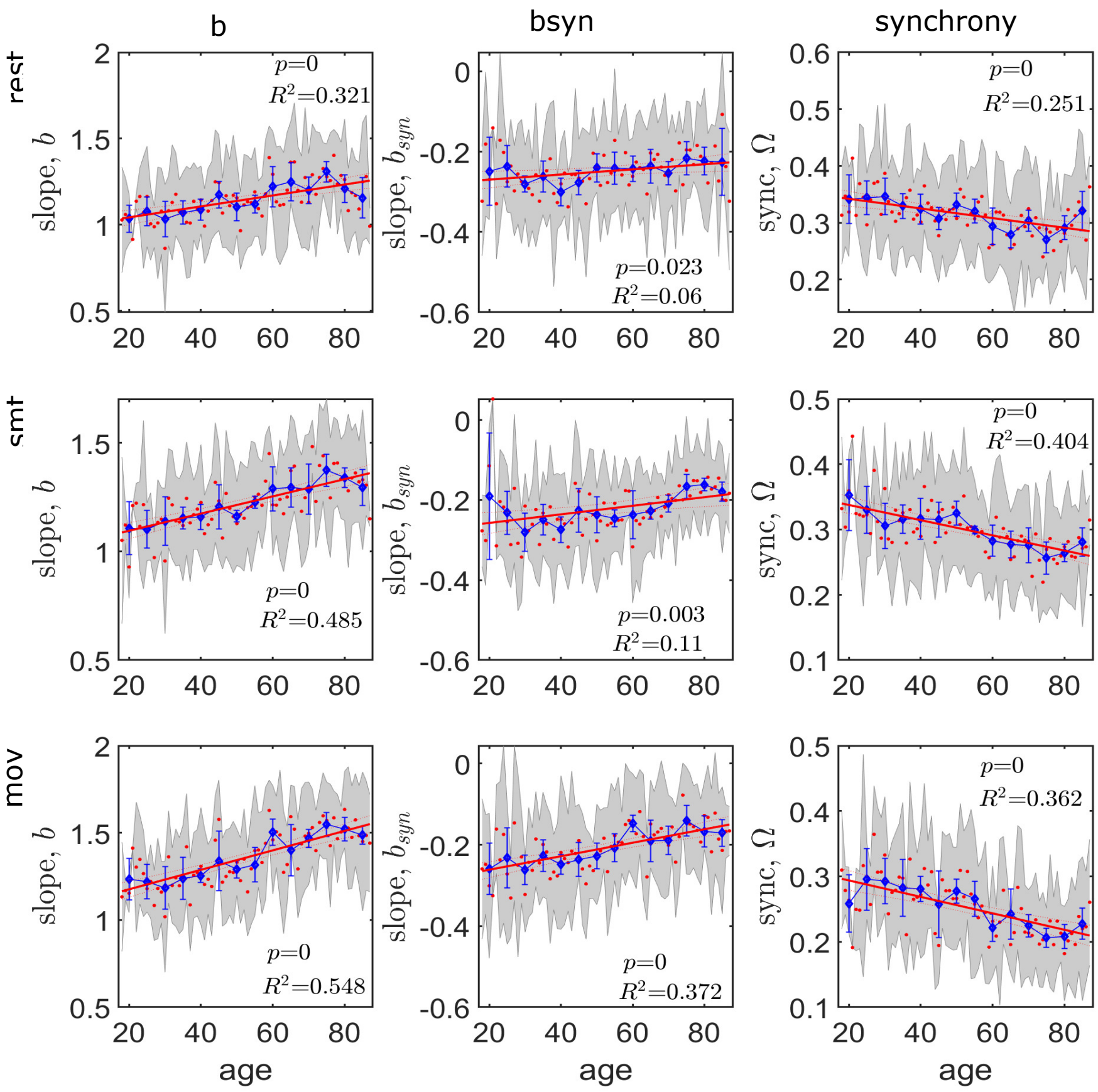

Fig T. Effects of age on the synchrony-driven spatial SL for three brain states. We use least-square linear regression (red line) to fit b_{syn} and age, where age is the continuous independent variable. Blue curve is the slope value derived by averaging over five years bin. Year-wise standard deviation is shown by gray error shade. Details of the stats are given in the legend of each figure. Significance threshold is set at $p<0.005$.

6.8 Group-wise analysis: Age-related alterations in the synchrony-induced SL exponent under three brain states

Associations between the spatial SL (b_{syn}) and synchrony (Ω) are shown for three age groups in *rest* (A-C), *mov* (D-F) and smt (G-I) states.

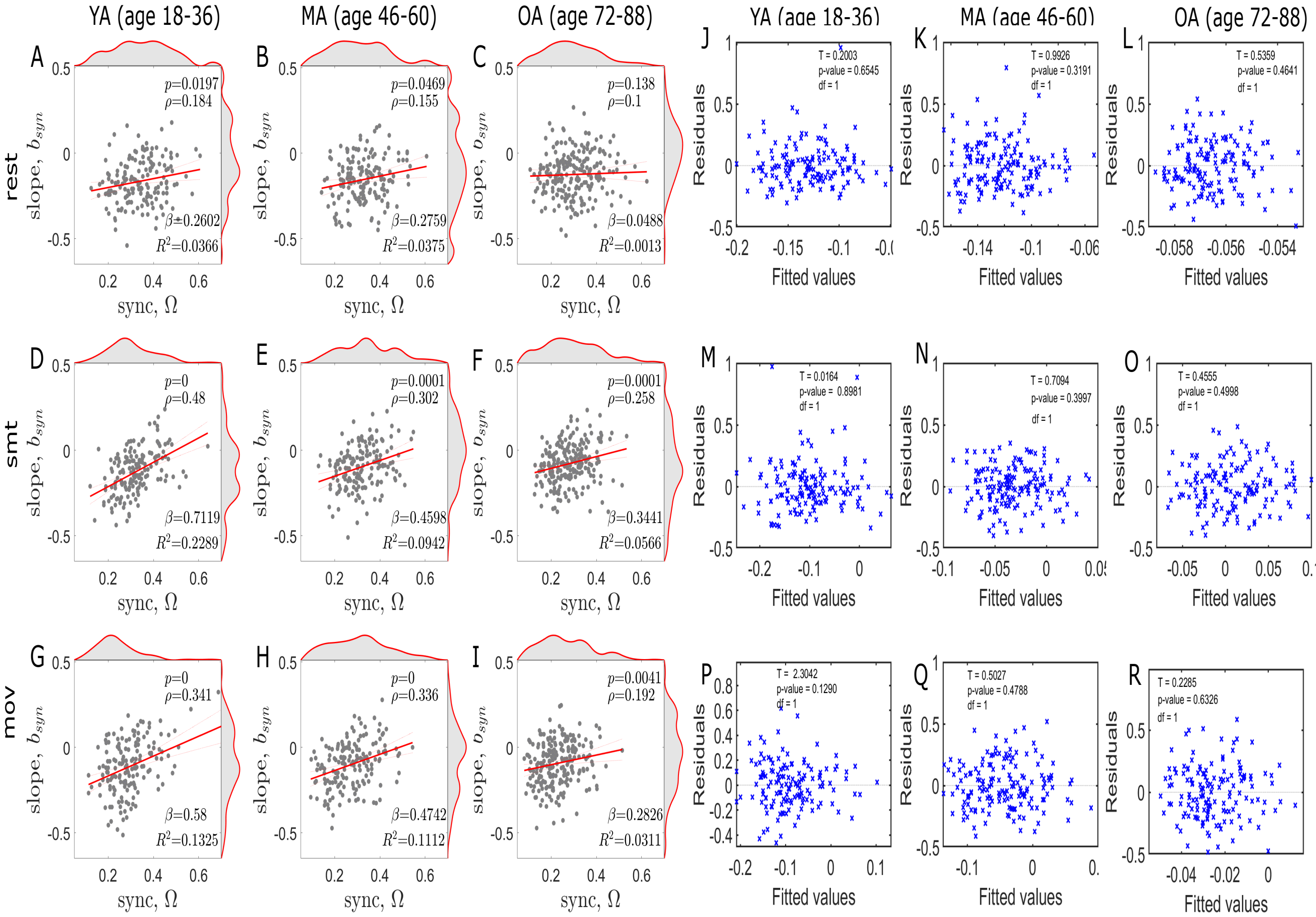

Fig U. Group-wise analysis: age-related alterations in the synchrony-induced SL exponent in three brain states. Age-related changes in the spatial SL, i.e., association between the spatial SL (b_{syn}) and synchrony (Ω) are shown for three age groups in rest (A-C), movie (D-F) and smt (G-I) states. (A,D,G) In young adults (YA), the slope (β) remains positive and significant in all three conditions. (B,E,H) In middle-aged adults (MA), the association slope between the spatial scaling exponent and synchrony becomes flat, whereas, (C,F,I) in older adults (OA), the slop tends to negative. The age-related changes are much prominent in the movie watching task. However, it can be observed that the slope, β has a tendency to rotate clockwise with increasing age in all three brain states. (J-R) The Breusch-Pagan test was conducted to assess whether the residuals from the linear regression exhibit heteroscedasticity — that is, whether the variance of the error terms changes systematically with the fitted values. Across all three mental states and three age groups, the BP test statistics were low: (J-L) T= 0.2003, 0.9926, and 0.5359 with corresponding p-values= 0.6545, 0.3191, and 0.4641 for rest; (M-O) T= 0.0164, 0.7094, and 0.4555 with p-values= 0.8981, 0.3997, and 0.4998 for smt; (P-R) T= 2.3042, 0.5027, and 0.2285 for mov) with p-values= 0.129, 0.4788, and 0.6326 for mov, all well above the conventional significance threshold of 0.05, with one degree of freedom (df=1) in each case. All the tests provide no statistically significant evidence of heteroscedasticity, suggesting that the error variance remains constant across all the linear fittings. This indicates that the residual variance is approximately constant across the range of predictor values, satisfying the key assumption of ordinary least squares regression.

7 Cross validation in NKI dataset: Healthy aging cohort

Data description and preprocessing: Resting-state BOLD activity is processed using AAL parcellation (116 ROIs) in Nathan Kline Institute (NKI)/Rockland dataset, (available in the UCLA Multimodal Connectivity Database (UMCD) [22] from total N=89 healthy participants, young: N=45 (22 Female), and old: N=44 (28 Female). Young group with age range 18-30 years, mean age 22.6222 ± 3.3864. Elderly group with age range 59-83 years, mean age 67.4545 ± 7.1315. Functional MRI (fMRI) for resting state have been preprocessed using the CONN toolbox [23] (https://web.conn-toolbox.org/), a Matlab/SPM-based software. Data were preprocessed using a default preprocessing pipeline of CONN. The preprocessing methods were unwrapping using field-map images, realignment to correct for motion, slice timing correction, segmentation, normalisation to the MNI template, outlier rejection and functional smoothing. A Gaussian kernel with of 2 mm and TR=2.5 sec are used to perform spatial smoothing. Denoising helped remove signal changes related to white matter, cerebrospinal fluid, motion, breathing and cardiac pulsations. Band-pass filter with 0.004-0.1 Hz pass-band frequency is applied. Linear detrending are performed. Mean regional BOLD time series were estimated in 116 brain areas of AAL atlas [8]. BOLD signal has size: Time Points×ROIs = 900×116.

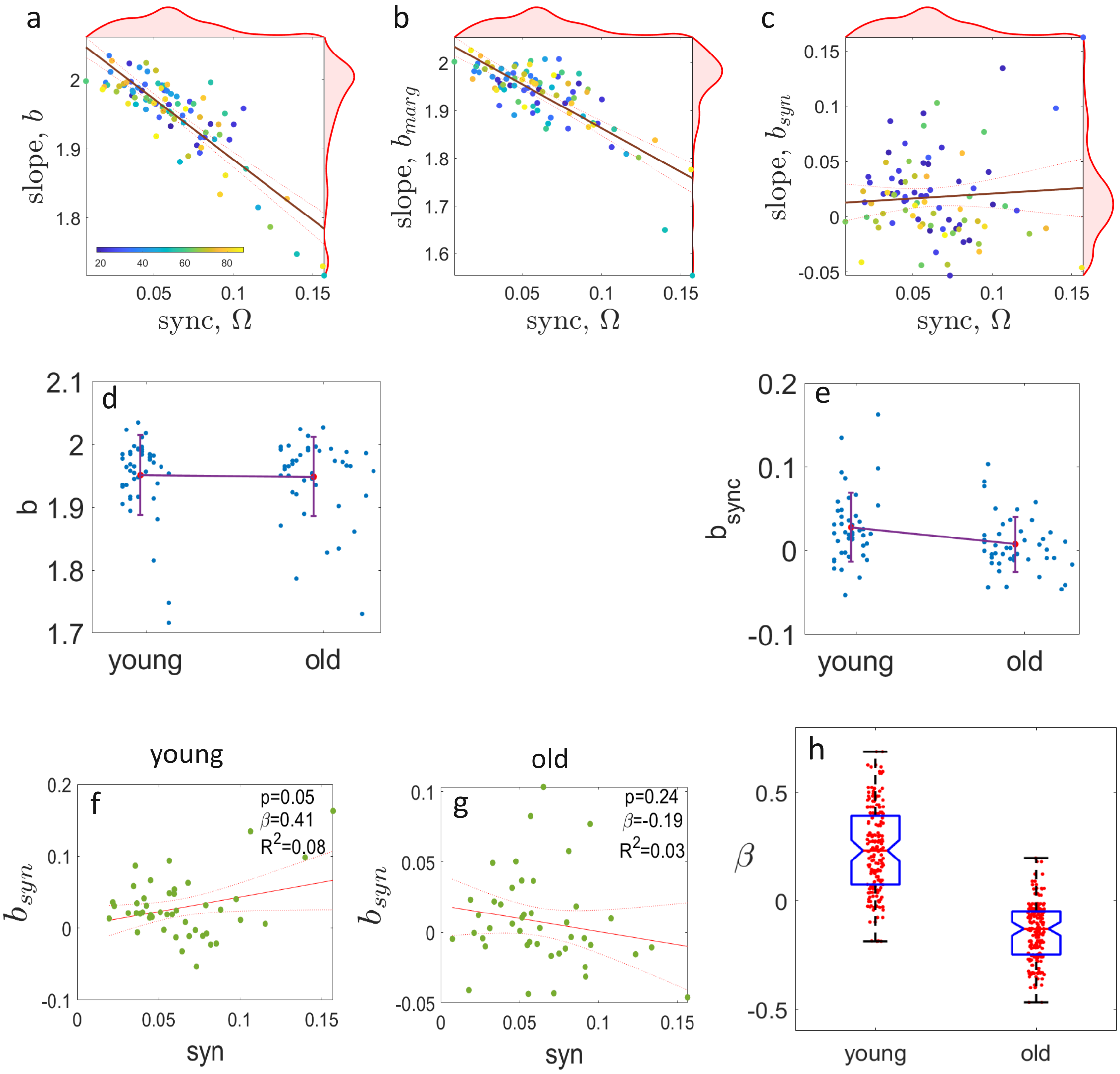

Fig V. Cross validation in NKI dataset of healthy aging cohorts. (a,b,c) Spatial SL slope versus synchrony is derived from resting state BOLD activity. First panel shows raw slope, middle panel shows result for surrogate data by phase randomization, last panel shows synchrony-induced SL slope for entire the participants (N=45 (22 Female) young, and N=44 (28 Female) old). (d, e) The raw and synchrony-induced SL slopes are shown to compare between young and elderly adults. (f, g) Synchrony versus SL slope (b_{syn}) is depicted for the two aging groups. With increasing age, the association between synchrony and Sl slope turns towards clock-wise direction, aligned to the results shown in Ua and Uc Fig for YA and OA, respectively. (h) The effect of the synchrony on scaling behavior is derived for 100 independent realizations, which is aligned to the results for CamCAN dataset shown in red curve comparing YA and OA in Uj Fig. At the advanced age, the β value reduced significantly compared to the young cohort.

8 Synchrony-induced scaling in neural disorder in UCLA dataset

Data description and preprocessing: We have randomly selected N=66 healthy controls (HC) (33 Female) age range 21-50 years, mean age 31.12 ± 8.62, and Attention-Deficit Hyperactivity Disorder (ADHD) (N=40 (19 Female)) age range 21-50 years, mean age 32.05 ± 10.41 from publicly available dataset shared by the Consortium for Neuropsychiatric Phenomics (CNP) (https://f1000research.com/articles/6-1262/v2) [24]. This is a part of the large study funded by the NIH Roadmap Initiative that aims to facilitate discovery of the genetic and environmental bases of variation in psychological and neural system phenotypes, to elucidate the mechanisms that link the human genome to complex psychological syndromes, and to foster breakthroughs in the development of novel treatments for neuropsychiatric disorders [24]. The study includes imaging of a large group of healthy individuals from the community (66 subjects),and individuals diagnosed with ADHD (45). The participants, ages 21-50, were recruited by community advertisements from the Los Angeles area and completed extensive neuropsychogical testing, in addition to fMRI scanning. Participants were screened for neurological disease, history of head injury with loss of consciousness, use of psychoactive medications, substance dependence within past 6 months, history of major mental illness or ADHD, and current mood or anxiety disorder. More details can be found in Ref. [24].

Resting state BOLD signal of size: Time Points × ROIs = $T \times n$= 152×116, and AAL atlas of n=116 ROIs are used. Spatial scaling exponent (b) is derived first from the preprocessed BOLD signals of individual participants, where the violin plots presented in Wa Fig are shown for HC and ADHD groups in blue and red, respectively. Wb Fig depicts SL exponents after removing marginals contributions. Wc and Wd Fig show the effect of synchrony on the SL exponent for HC and ADHD groups. The association between average synchrony and the spatial scaling, captured by the slope β through least square linear regression fit, is found to be insignificant within the people diagnosed with ADHD, but very prominent in healthy controls. Essentially, the brain-wide coordinated activity has no effect on the scaling law in case of ADHD. In other words, the synchrony-scaling relationship breaks down in neural disorder. Further, we have performed 1000 independent realizations to confirm our observations, and derived the association slope β, which depicts a sharp contrast between the healthy and ADHD groups, see We Fig.

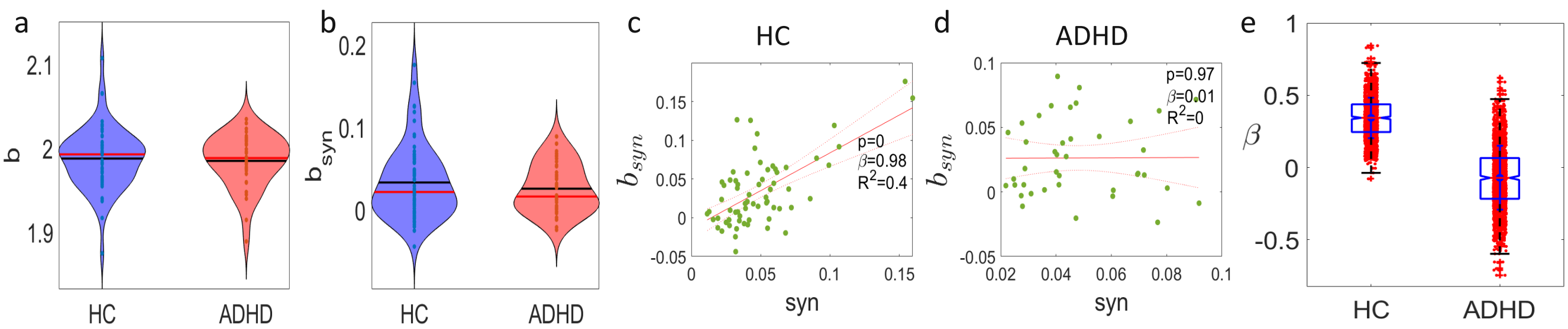

Fig W. Synchrony-induced scaling law in neurodevelopmental disorder. (a, b) Synchrony effects on the SL exponent (raw and synchrony-induced) for healthy participants and ADHD subjects. (c, d) The influence of synchrony on the SL is shown for HC and ADHD groups is obtained for a single realization using linear regression fit, where the association slope is β. It is observed that the effect of synchrony on the SL becomes not significant (p>0.05) in ADHD compared to the HC group. (e) Furthermore, from 1000 independent realizations, the the value of β is derived for the HC and ADHD groups. It seems that the neural disorder has reduced the values of β profoundly, in comparison to the healthy controls.

9 Details of the Datasets

**Dataset**	**Total**	**Group**	**Age range** (years)	**Mean age** ± **Std**
CamCAN	N=645 (326 Female)	Continuous age, N=645	18-88	54.24 ± 18.47
		Young, N=138	18-36	28.86 ± 5.06
		Middle, N=155	46-60	52.72 ± 4.46
		Old, N=146	72-88	78.78 ± 4.02
NKI	N=89 (50 Female)	Young, N=45	18-30	22.62 ± 3.39
		Old, N=44	59-83	67.46 ± 7.13
UCLA	N=106 (52 Female)	HC, N=66	21-50	31.12 ± 8.62
		ADHD, N=40	21-50	32.05 ± 10.41

Table B. Details of the empirical data from three independent cohorts. Total participants, N=840. Task conditions in CamCAN are resting state, movie watching and sensorimotor task. For NKI-Rockland and UCLA datasets, BOLD signals are obtained at eye open resting state.

10 Comparison with other metrics : Metastability, phase synchrony, entropy of functional connectivity dynamics

In this section, we employ three distinct global metrics widely used in neuroscience to investigate age-related changes in brain dynamics. This analysis enables us to identify specific characteristic changes and compare them with our main findings regarding synchrony-induced spatial SL exponents.

10.1 Metastability and Phase Synchrony

Recent brain network computational models suggest that the human brain achieves maximum metastability during resting states [25, 26] . This manifests as optimal information processing capability and switching behavior [25, 27–29]. Furthermore, the concept of metastability to measure brain dynamics enhances our understanding of cognitive aging theories [29–31] and help explore physiological substrate changes during aging [31, 32]. We have selected metastability index (meta) as one of the global metrics to capture alterations in the brain-wide dynamics by comparing among different brain states across the adulthood aging trajectory. Metastability index quantifies the continuous transitional states observed in brain signals without settling into specific attractor states [33–35] and serves as a metric for capturing the brain's dynamic operating point during rest [25, 30, 36]. Metastability index (meta) captures temporal fluctuation by estimating brain regions' tendencies to deviate from coherent states. This provides an effective measurement unit for observing spatial cohesion during short periods (hours) and may serve as a key component for observing gradual changes in lifespan brain neural dynamics. We must mention here that the Kuramoto order parameter is a valid measure in rhythmic oscillations, thereby, the rhythmicity in BOLD signal is achieved by applying a narrow band filter that allows pass-band frequency ranging from 0.001 Hz to 0.1 Hz, prior to compute metastability and synchrony measure. The measurement begins with calculating the Kuramoto order parameter [37], $r(t)$, using,

$$r(t) = \frac{1}{n}\left|\sum_{k=1}^{n} e^{i\phi_k(t)}\right| \tag{S42}$$

where, $\phi_k(t)$ is the instantaneous phase of kth brain region. The value of $r(t)$ lies between $0 \leq r(t) \leq 1$; $r(t) = 1$ representing complete synchrony, $r(t) = 0$ for desynchrony, and $(0 < r(t) < 1)$ representing partial synchrony among all brain regions. Hence, mean of $r(t)$ indexes the global coherence among a system of oscillating brain regions. Metastability index (meta) is calculated by taking the standard deviation of $r(t)$, as:

$$\text{meta} = \sqrt{\left\langle (r(t) - \langle r(t)\rangle)^2 \right\rangle}, \tag{S43}$$

where $\langle . \rangle$ denotes average over time. The global phase synchrony (syn) is measured by taking average over time as,

$$\text{syn} = \langle r(t)\rangle. \tag{S44}$$

The dynamical measure of metastability offers several advantages: it quantifies neural systems' tendencies to operate in both segregative and integrative modes [35], characterizes criticality states where dynamical systems remain at the edge of phase transitions with enhanced information processing capabilities [25, 38], and enables investigation through generative neural dynamics models, providing mechanistic insights into network processes generating brain signals [36, 39, 40]. Based on these insights, we employ metastability as the dynamical measure to capture brain dynamics help compare against our proposed metric, the synchrony-induced scaling exponent, while capturing alteration patterns in different brain states along the adulthood aging.

10.2 Entropy of functional connectivity dynamics

Quantifying the temporal stability of dFC patterns is of immediate concern to studies investigating the relationship between resting state and task-related brain dynamics (Li, Lu, & Yan, 2019). Recent studies show that the correlations among brain regions are not stationary, but evolve over time, and have refocused the study of spontaneous brain activity on characterizing these time-varying functional interactions. the synchrony between the BOLD activities of different brain regions displays global slow fluctuations that reflect the dynamical association and dissociation of functional coherent clusters. Emerging evidence suggests that FC dynamics FC may characterize alterations in macroscopic neural activity patterns underlying critical aspects of cognition and behavior. Temporal similarity is captured by measuring entropy of the functional connectivity dynamics (FCD) [41–43], the time-time correlation matrix. This helps us to examine dynamical pattern of invariance or alterations in the temporal stability across slow time scale, i.e., lifespan aging. Static FC is quantified with metrics such as correlation, covariance, and mutual information between the time series of different regions, wherein the temporal and spatial scales examined are determined by the question of interest (Bressler and Menon, 2010; Bullmore and Sporns, 2009; Friston, 2011). It therefore represents an empirical characterization of the temporal relationship between regions, without indicating how the temporal covariation is mediated (Friston, 2011; Friston and Buchel, 2007).

Instantaneous phases $\theta(i,t)$ of the BOLD signals of $i = 1, ..., n$ ($n = 116$) regions are derived using Hilbert transform. Phase similarity between i^{th} and j^{th} brain regions at t time point is captured by the stream of FCs, computed by

$$\text{dFC}(i,j,t) = \cos(\theta(i,t) - \theta(j,t))$$

where $i, j = 1, ..., n$. Principal component analysis (PCA) reduces the state-space dimension into a much lesser dimension, e.g., the dFC matrix of size $n \times n$ is reduced to size $3 \times n$, considering top three principle components, three largest eigen values. We express the original vector space as, $\mathbf{dFC}_t = \mathbf{V}^T\mathbf{D}\mathbf{V}$ at time point t, where $\mathbf{V}$ is the eigenvector of size $n \times n$. The diagonal of $\mathbf{D}$ contains sorted eigen values, e.g., $\lambda_1 > \lambda_2 > \cdots > \lambda_n$, then, $\mathbf{D} = \begin{pmatrix} \lambda_1 & \cdots & 0 \\ \vdots & \ddots & \vdots \\ 0 & \cdots & \lambda_n \end{pmatrix}$.

If k is the number of principal components chosen for the analysis, and reduced subspace at time point t is $\mathbf{FCD}_t$; the reduced-dimension eigen matrix is $\hat{\mathbf{D}}$, corresponding eigenvector is $\hat{\mathbf{V}}$, then we have

$$\mathbf{FCD}_t = \hat{\mathbf{V}}^T\hat{\mathbf{D}}\hat{\mathbf{V}}.$$

Temporal stability of the dominant subspace $\mathbf{FCD}(k,n,t)$ is estimated by the similarity between two FCDs at any two given time points (say, t_x and t_y) is measured using angular distance. The principal angle between the $\mathbf{FCD}$ subspaces from different time points is calculated by the temporal stability matrix [44, 45], say Φ of size $T \times T$, where T is the total time points, then the angular distance between two time points, t_x and t_y is derived by,

$$\Phi(t_x, t_y) = \angle(\mathbf{FCD}_{t_x}, \mathbf{FCD}_{t_y}).$$

We have quantified complexity of temporal stability matrices by measuring entropy for all three categories, rest, movie viewing and sensorimotor task across the adulthood aging trajectory. The entropy is defined as,

$$E = -\sum p \log(p),$$

where p contains the normalized histogram counts of the temporal stability matrix.

10.3 Results

The dynamical measure of metastability presented in Xa-Xc Figs, using metastability index (meta). No significant changes are observed in resting state [Xa Fig], but significantly decreased in the sensory motor and movie watching tasks; as seen in Xb and Xc Fig. The global phase synchrony (syn) derived by taking time average of the Kuramoto order parameter is used here to effectively track how global coherence changes with age, and also help compare the results against the metric, spatial synchrony Ω. Significant decreasing

patterns are observed in the global phase synchrony with increasing age, as seen from Xd-Xf Figs in all three brain states, which are consistent with the results presented in the main text, Fig 5 (third column). Both the metrics have captured significant decline in the global coherence level with age. Xg-Xi Figs illustrate changes in the entropy of functional connectivity dynamics (FCD) against age. No significant changes are observed in resting state; see Xg Fig, where as entropy significantly increased with age in the sensory motor and movie watching tasks; as seen in Xh and Xi Fig. In summary, it can be concluded that age-related changes are prominent in task evoked activity rather than resting state, whereas age profoundly affects brain-wide coordinated activity measured by synchrony.

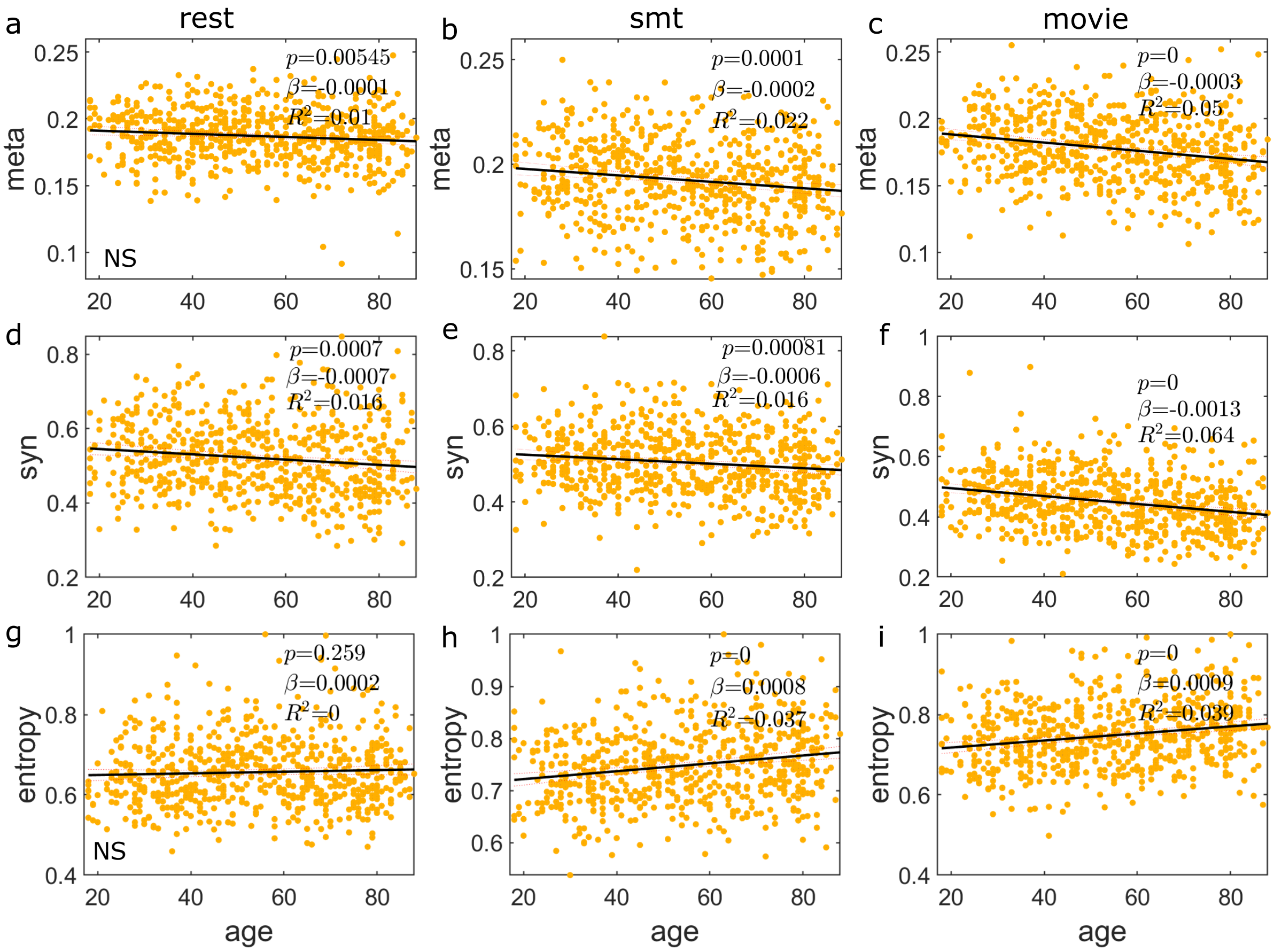

Fig X. Age-related changes captured by other metrics: metastability, global phase synchrony, and entropy of functional connectivity dynamics. (a-c) Metastability index (meta), (d-f) global phase synchrony (syn), and (g-i) entropy of functional connectivity dynamics (entropy) are plotted against age, arranged from top to bottom rows, where columns from left to right represent resting state (a,d,g), sensory motor (b,e,h) and movie watching task (c,f,i) based results. Statistical significance level is defined by $p < 0.005$, otherwise marked as not significant (NS). We fit an ordinary least-squares linear regression line to each scatter plot, with age as the independent variable.

References

[1] David R Brillinger. *Time series: data analysis and theory.* SIAM, 2001.

[2] Daniel C Reuman, Lei Zhao, Lawrence W Sheppard, Philip C Reid, and Joel E Cohen. Synchrony affects Taylor's law in theory and data. *Proceedings of the National Academy of Sciences*, 114(26):6788–6793, 2017.

[3] Fabrizio Gabbiani and Steven James Cox. Chapter 18 - Stochastic processes. In Fabrizio Gabbiani and Steven James Cox, editors, *Mathematics for Neuroscientists (Second Edition)*, pages 335–349. Academic Press, San Diego, second edition edition, 2017.

[4] John Semmlow. Chapter 10 - Stochastic, nonstationary, and nonlinear systems and signals. In John Semmlow, editor, *Circuits, Signals and Systems for Bioengineers (Third Edition)*, Biomedical Engineering, pages 449–489. Academic Press, third edition edition, 2018.

[5] Avijit Lahiri. Chapter 7 - Optical coherence: Statistical optics. In Avijit Lahiri, editor, *Basic Optics*, pages 605–696. Elsevier, Amsterdam, 2016.

[6] Yuzuru Mitsui and Hiroshi Kori. Waveform proportionality and Taylor's law induced by synchronization of periodic and chaotic oscillators. *Physical Review Letters*, 134(16):167202, 2025.

[7] Andrew Liebhold, Walter D Koenig, and Ottar N Bjørnstad. Spatial synchrony in population dynamics. *Annu. Rev. Ecol. Evol. Syst.*, 35:467–490, 2004.

[8] Nathalie Tzourio-Mazoyer, Brigitte Landeau, Dimitri Papathanassiou, Fabrice Crivello, Octave Etard, Nicolas Delcroix, Bernard Mazoyer, and Marc Joliot. Automated anatomical labeling of activations in SPM using a macroscopic anatomical parcellation of the MNI MRI single-subject brain. *Neuroimage*, 15(1):273–289, 2002.

[9] Joel E Cohen and Meng Xu. Random sampling of skewed distributions implies Taylor's power law of fluctuation scaling. *Proceedings of the National Academy of Sciences*, 112(25):7749–7754, 2015.

[10] Gary W Oehlert. A note on the delta method. *The American Statistician*, 46(1):27–29, 1992.

[11] David W Hosmer Jr, Stanley Lemeshow, and Susanne May. *Applied survival analysis: Regression modeling of time-to-event data*, volume 618. John Wiley & Sons, 2008.

[12] Francisco J Baldán and José M Benítez. Complexity measures and features for times series classification. *Expert Systems with Applications*, 213:119227, 2023.

[13] Carl H Fischer. On correlation surfaces of sums with a certain number of random elements in common. *The Annals of Mathematical Statistics*, 4(2):103–126, 1933.

[14] Jason R Taylor, Nitin Williams, Rhodri Cusack, Tibor Auer, Meredith A Shafto, Marie Dixon, Lorraine K Tyler, Richard N Henson, et al. The Cambridge Centre for Ageing and Neuroscience (Cam-CAN) data repository: Structural and functional MRI, MEG, and cognitive data from a cross-sectional adult lifespan sample. *Neuroimage*, 144:262–269, 2017.

[15] Meredith A Shafto, Lorraine K Tyler, Marie Dixon, Jason R Taylor, James B Rowe, Rhodri Cusack, Andrew J Calder, William D Marslen-Wilson, John Duncan, Tim Dalgleish, et al. The Cambridge Centre for Ageing and Neuroscience (Cam-CAN) study protocol: a cross-sectional, lifespan, multidisciplinary examination of healthy cognitive ageing. *BMC Neurology*, 14(1):1–25, 2014.

[16] Dardo G Tomasi, Ehsan Shokri-Kojori, and Nora D Volkow. Brain network dynamics adhere to a power law. *Frontiers in Neuroscience*, 11:72, 2017.

[17] Dean Prichard and James Theiler. Generating surrogate data for time series with several simultaneously measured variables. *Physical Review Letters*, 73(7):951, 1994.

[18] John-Dylan Haynes and Geraint Rees. Decoding mental states from brain activity in humans. *Nature Reviews Neuroscience*, 7(7):523–534, 2006.

[19] Erfan Nozari, Maxwell A Bertolero, Jennifer Stiso, Lorenzo Caciagli, Eli J Cornblath, Xiaosong He, Arun S Mahadevan, George J Pappas, and Dani S Bassett. Macroscopic resting-state brain dynamics are best described by linear models. *Nature Biomedical Engineering*, 8(1):68–84, 2024.

[20] Maxwell Shinn, Amber Hu, Laurel Turner, Stephanie Noble, Katrin H Preller, Jie Lisa Ji, Flora Moujaes, Sophie Achard, Dustin Scheinost, R Todd Constable, et al. Functional brain networks reflect spatial and temporal autocorrelation. *Nature Neuroscience*, 26(5):867–878, 2023.

[21] Zoltán Eisler, Imre Bartos, and János Kertész. Fluctuation scaling in complex systems: Taylor's law and beyond. *Advances in Physics*, 57(1):89–142, 2008.

[22] Jesse A Brown, Jeffrey D Rudie, Anita Bandrowski, John D Van Horn, and Susan Y Bookheimer. The UCLA multimodal connectivity database: A web-based platform for brain connectivity matrix sharing and analysis. *Frontiers in Neuroinformatics*, 6:28, 2012.

[23] Susan Whitfield-Gabrieli and Alfonso Nieto-Castanon. CONN: A functional connectivity toolbox for correlated and anticorrelated brain networks. *Brain Connectivity*, 2(3):125–141, 2012.

[24] Russell A Poldrack, Eliza Congdon, William Triplett, KJ Gorgolewski, KH Karlsgodt, JA Mumford, FW Sabb, NB Freimer, ED London, TD Cannon, et al. A phenome-wide examination of neural and cognitive function. *Scientific Data*, 3(1):1–12, 2016.

[25] G Deco, ML Kringelbach, VK Jirsa, and P Ritter. The dynamics of resting fluctuations in the brain: metastability and its dynamical cortical core. *Sci. Rep.*, 7(1):1–14, 2017.

[26] SG Surampudi, J Misra, G Deco, RS Bapi, A Sharma, and D Roy. Resting state dynamics meets anatomical structure: Temporal multiple kernel learning (tMKL) model. *NeuroImage*, 184:609–620, 2019.

[27] Gerhard Werner. Metastability, criticality and phase transitions in brain and its models. *Biosystems*, 90(2):496–508, 2007.

[28] G Deco, ET Rolls, and R Romo. Stochastic dynamics as a principle of brain function. *Progress in Neurobiology*, 88(1):1–16, 2009.

[29] M Shanahan. Metastable chimera states in community-structured oscillator networks. *Chaos: An Int. Jour. of Nonlinear Sci.*, 20(1):013108, 2010.

[30] Gustavo Deco and Morten L Kringelbach. Metastability and coherence: extending the communication through coherence hypothesis using a whole-brain computational perspective. *Trends in Neurosciences*, 39(3):125–135, 2016.

[31] S Naik, A Banerjee, RS Bapi, G Deco, and D Roy. Metastability in senescence. *Trends in Cognitive Sciences*, 21(7):509–521, 2017.

[32] S Naik, O Subbareddy, A Banerjee, D Roy, and RS Bapi. Metastability of cortical bold signals in maturation and senescence. In *2017 International Joint Conference on Neural Networks (IJCNN)*, pages 4564–4570. IEEE, 2017.

[33] S. L. Bressler and J. A. S. Kelso. Cortical coordination dynamics and cognition. *Trends in Cognitive Sciences*, 5:26–36, January 2001.

[34] Emmanuelle Tognoli and J. A. Scott Kelso. Brain coordination dynamics: True and false faces of phase synchrony and metastability. *Progress in Neurobiology*, 87:31–40, January 2009.

[35] Emmanuelle Tognoli and JA Scott Kelso. The metastable brain. *Neuron*, 81(1):35–48, 2014.

[36] Amit Naskar, Anirudh Vattikonda, Gustavo Deco, Dipanjan Roy, and Arpan Banerjee. Multi-scale dynamic mean field model (MDMF) relates resting-state brain dynamics with local cortical excitatory–inhibitory neurotransmitter homeostasis. *Network Neuroscience*, pages 1–55, 2021.

[37] Yoshiki Kuramoto. Chemical turbulence. In *Chemical Oscillations, Waves, and Turbulence*, pages 111–140. Springer Berlin Heidelberg, 1984.

[38] Luca Cocchi, Leonardo L. Gollo, Andrew Zalesky, and Michael Breakspear. Criticality in the brain: A synthesis of neurobiology, models and cognition. *Progress in Neurobiology*, 158:132–152, November 2017.

[39] James A. Roberts, Leonardo L. Gollo, Romesh G. Abeysuriya, Gloria Roberts, Philip B. Mitchell, Mark W. Woolrich, and Michael Breakspear. Metastable brain waves. *Nature Communications*, 10:1056, March 2019.

[40] Michael Breakspear. Dynamic models of large-scale brain activity. *Nature Neuroscience*, 20:340–352, February 2017.

[41] Nora Leonardi, Jonas Richiardi, Markus Gschwind, Samanta Simioni, Jean-Marie Annoni, Myriam Schluep, Patrik Vuilleumier, and Dimitri Van De Ville. Principal components of functional connectivity: A new approach to study dynamic brain connectivity during rest. *NeuroImage*, 83:937–950, 2013.

[42] R Matthew Hutchison, Thilo Womelsdorf, Elena A Allen, Peter A Bandettini, Vince D Calhoun, Maurizio Corbetta, Stefania Della Penna, Jeff H Duyn, Gary H Glover, Javier Gonzalez-Castillo, et al. Dynamic functional connectivity: Promise, issues, and interpretations. *Neuroimage*, 80:360–378, 2013.

[43] Nisha Chetana Sastry, Dipanjan Roy, and Arpan Banerjee. Stability of sensorimotor network sculpts the dynamic repertoire of resting state over lifespan. *Cerebral Cortex*, 33(4):1246–1262, 2023.

[44] Arpan Banerjee, Ajay S Pillai, Justin R Sperling, Jason F Smith, and Barry Horwitz. Temporal microstructure of cortical networks (TMCN) underlying task-related differences. *NeuroImage*, 62(3):1643–1657, 2012.

[45] Ake Bjorck and Gene H Golub. Numerical methods for computing angles between linear subspaces. *Mathematics of Computation*, 27(123):579–594, 1973.
