# Supplementary material for: A generalized scaling law reveals synchrony-driven reorganization of brain dynamics across human lifespan
Source: PLoS Comput Biol. 2026 Oct 5;22(10):e1014821. doi: 10.1371/journal.pcbi.1014821 (PMC13652345; doi:10.1371/journal.pcbi.1014821)
Supplement: Supplementary file 2 [file pcbi.1014821.s002.pdf]

Response to Reviewers
Manuscript ID: PCOMPBIOL-D-26-00299R1

"A generalized scaling law reveals synchrony-driven reorganization of brain dynamics across the human lifespan"

We thank the editors for the opportunity to revise our manuscript and the reviewers for their valuable comments and constructive feedback. We have addressed all concerns and suggestions raised by the referees and provide a point-by-point response below. All corresponding changes have been highlighted in red in the revised manuscript.

Summary of Changes in the Revised Version:

- **Figure 1:** Caption revised (**lines 162–177**).
- **Figure 5:** Group range and corresponding text in the Results section revised (**lines 225–241, main manuscript**).
- **Figure S21:** New results added (**line 646**).
- New section added: "Mathematical derivation of the spatial scaling exponent" (**lines 469–566**).
- New section added: "Surrogate time series: phase-randomization" (**lines 567–581**).
- Text revised in the Abstract, Introduction, Results, and Discussion sections.

Point-by-point responses to the Reviewers' Comments.

Reviewer #1:

Reviewer Comments: This manuscript introduces a creative and interesting extension of Taylor's law to brain data by replacing the mean with RMS, and connects this scaling behavior to brain synchrony. The combination of theory, simulations, and analysis of large fMRI datasets across the lifespan is a clear strength. The idea of linking scaling laws with coordinated brain activity is novel and potentially impactful, and the inclusion of aging and ADHD analyses adds useful real-world relevance. Overall, the paper tackles an important problem and proposes a framework that could be valuable for studying large-scale brain dynamics. That said, there are a few important issues that need to be addressed. The definition of synchrony is not standard and needs clearer justification, especially in comparison with commonly used measures.

Authors' Responses: We thank the reviewer for the appreciation of the study and the constructive feedback. We agree that clarifying our synchrony measure is important, and we address this below. In this work, the global metric, *average synchrony,* is defined as the average functional connectivity (FC) across all brain regions and derived from the variance of the spatial mean of the BOLD signals.

This choice is deliberate and grounded in both theoretical and empirical considerations. The synchrony metric is chosen over alternatives such as the Kuramoto order parameter or phase-locking value, which assume strictly oscillatory dynamics less suitable for hemodynamically filtered fMRI signals (0.001Hz-0.1Hz). Crucially, our metric bears a direct analytical relationship to the proposed scaling law: it enters explicitly into the closed-form expression that links the scaling exponent to coordinated activity, enabling theoretical predictions to be tested empirically. This tight coupling between the metric and the framework is a key feature of our approach. Also, we made a comparison among other metrics and the observed metric as a function of age. Finally, to avoid ambiguity, we have renamed the metric from "synchrony" to "average spatial synchrony" (or "average synchrony") throughout the revised manuscript.

Reviewer Comments: **Some of the main claims—particularly about the generality of the scaling law—feel a bit too strong given the assumptions involved (like detrending and approximate normality). The heavy reliance on detrending is also concerning, since it may affect the results in ways that are not fully explored.**

Authors' Responses: We thank the reviewer for this important critique. We acknowledge that the analytical derivations invoke distributional assumptions. However, our simulations validate the scaling law across four qualitatively distinct distributions such as Poisson, binomial, uniform, and gamma, covering discrete, continuous, symmetric, and skewed cases, collectively supporting the framework's broad applicability. Nonetheless, we have moderated overly strong claims in the revision to accurately reflect these assumptions.

To suppress trends and temporal mean in the spatio-temporal time series data, detrending is a principled necessity rather than an arbitrary choice. Also, the empirical data having inherent non-stationarities, e.g., in BOLD signals, displays slow drifts and physiological trends, that can systematically bias the spatial mean and spatial variance, directly confounding Taylor's law. Therefore, detrending that helps remove trends and temporal means is a necessary steps in deriving long-term correlation and spatial scaling exponent, while replacing the sample mean with spatial RMS naturally accommodates this. Further, to address the reviewer's concern, we had included a sensitivity analysis comparing results with and without detrending, using numerical examples of Poisson, binomial, uniform, and gamma, bounding its influence on the scaling exponent.

Please see **Supplemental Material, Figs. S2-S8, lines 608–616**, Supporting Information Legends. The simulation-based results exemplified the effect of temporal mean and trend on the spatial scaling exponent. When the time series data of any specific distribution is shifted by a constant value, while all other parameters to generate the data distributions are kept intact, the scaling exponent varies widely; however, following the detrending operation, the data display a uniform scaling value.

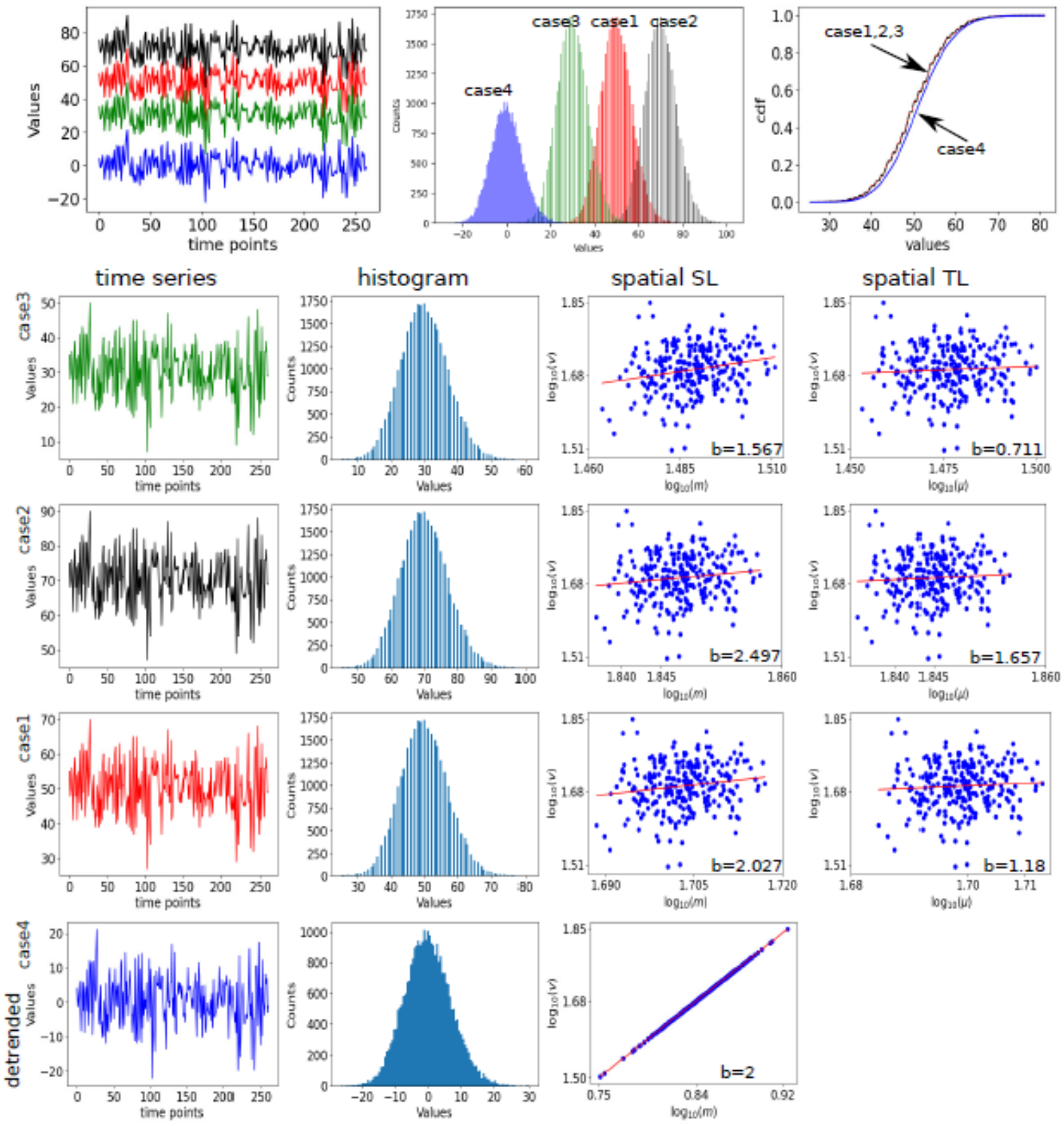

Figure S2: Test 1 for Poisson distribution. We have used Python function "numpy.random.poisson(50, size= (261, 116))" to create the spatio-temporal time series data. The seeds are varied randomly for different locations. Long-term correlation between any pair of locations are almost zero. Next, shifted the temporal mean of the entire data by adding and subtracting 50 from the previous case example to create case 2 (black) and case 3 (green) examples. The detrended scenario, labeled as case 4, shown in blue at the bottom row.

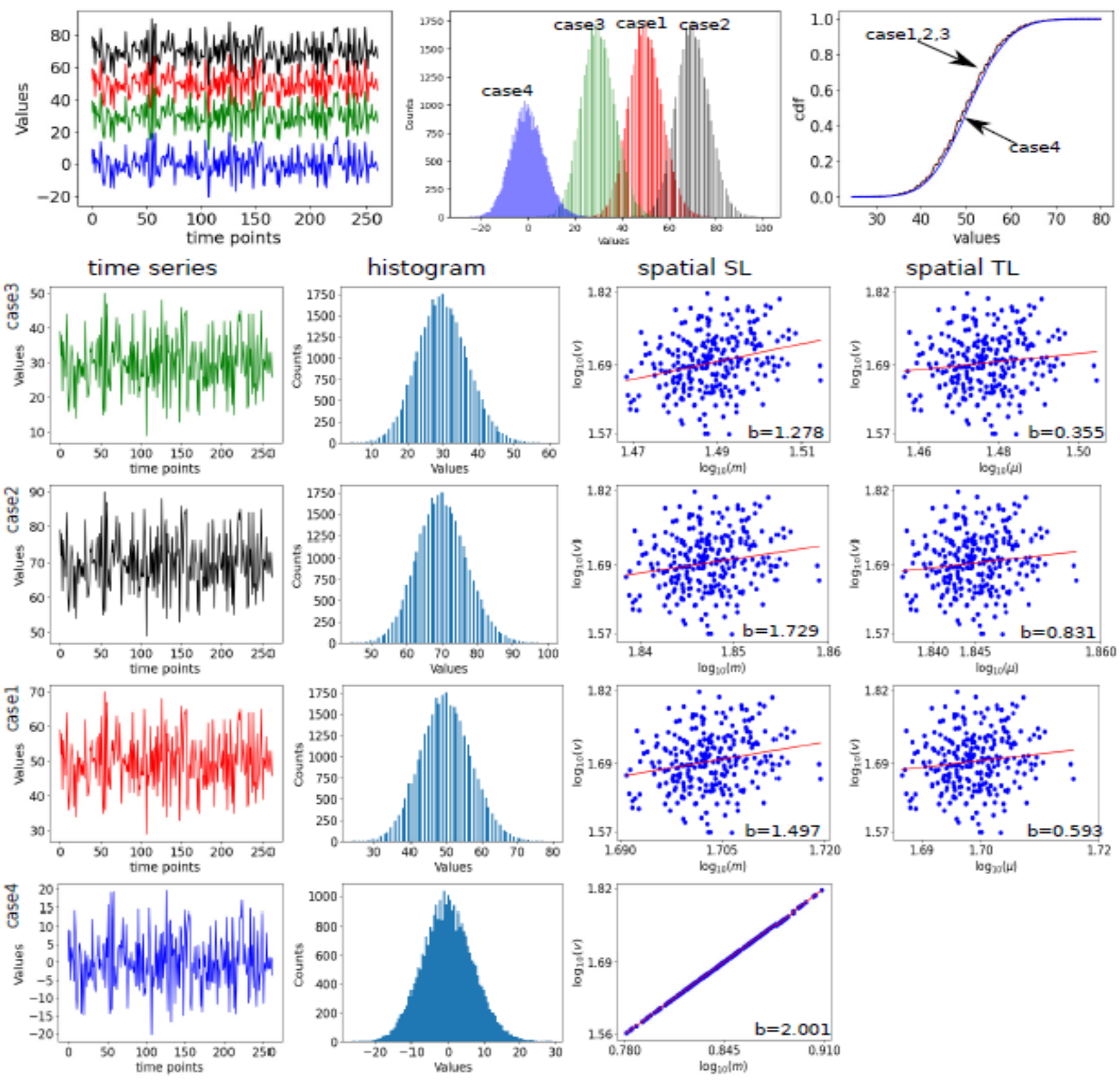

Figure S3: **Test 2 for Poisson distribution.** We have used Python function "numpy.random.poisson(50, size= (261, 116))" to create the spatio-temporal time series data shown in red. Next, shifted the temporal mean of the entire data by adding and subtracting 50 from the previous case example to create case 2 (black) and case 3 (green) examples. The detrended scenario, labeled as case 4, shown in blue at the bottom row.

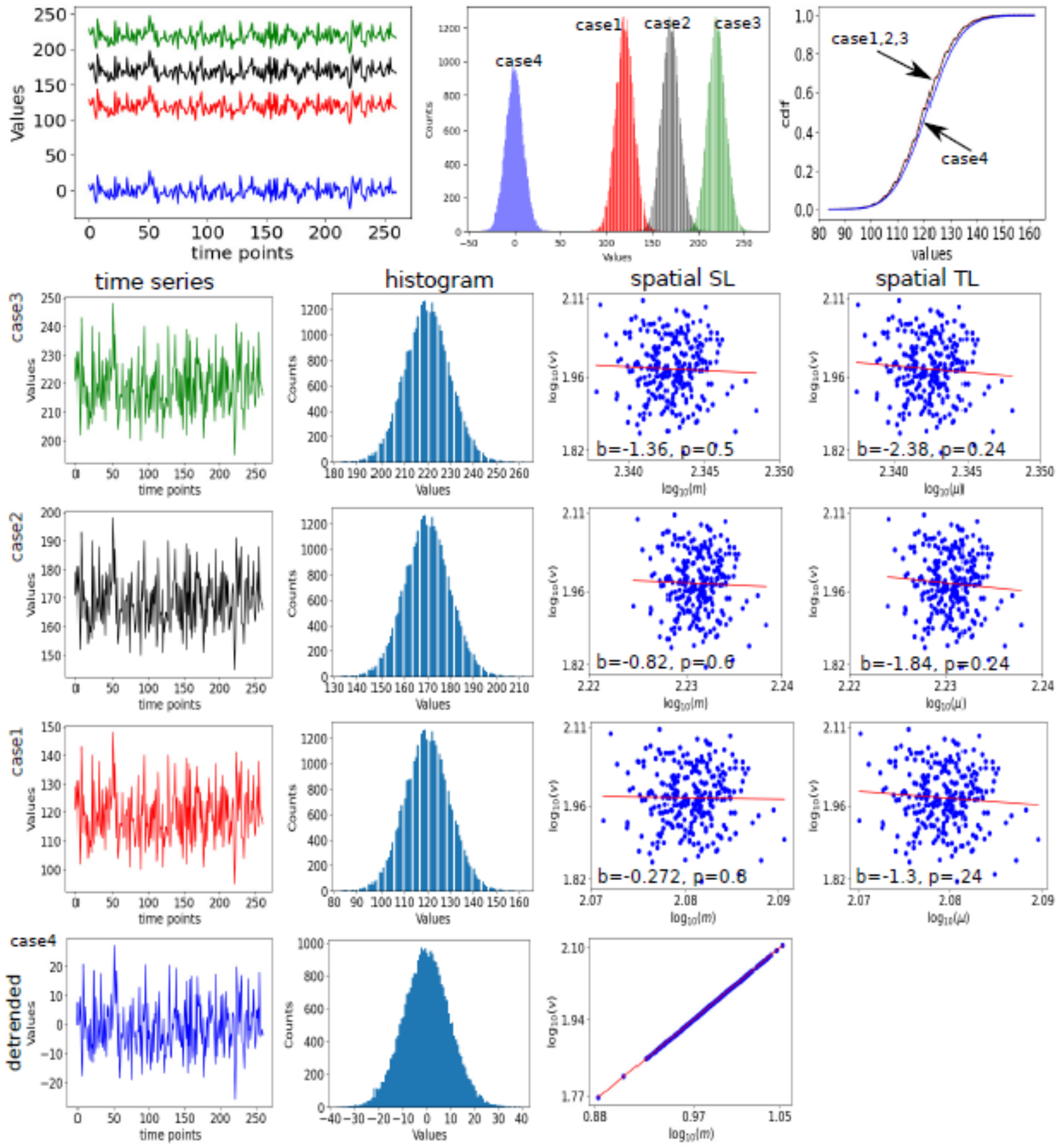

Figure S4: **Test 1 for Binomial distribution.** The Python function 'numpy.random.binomial(n=100, p=0.5, size= (261, 116))' has been used to construct the time series. The first distribution created with the temporal mean at 125, shown in red labeled as case 1. Next, we have shifted the mean of the entire data, a constant value 50 and 100 are added to the temporal of the original time series data (red) while freezing the variance and auto-correlation, shown in black and green (case 2 and case 3). Detrended data analysis is marked as case 4, shown in blue at the bottom row.

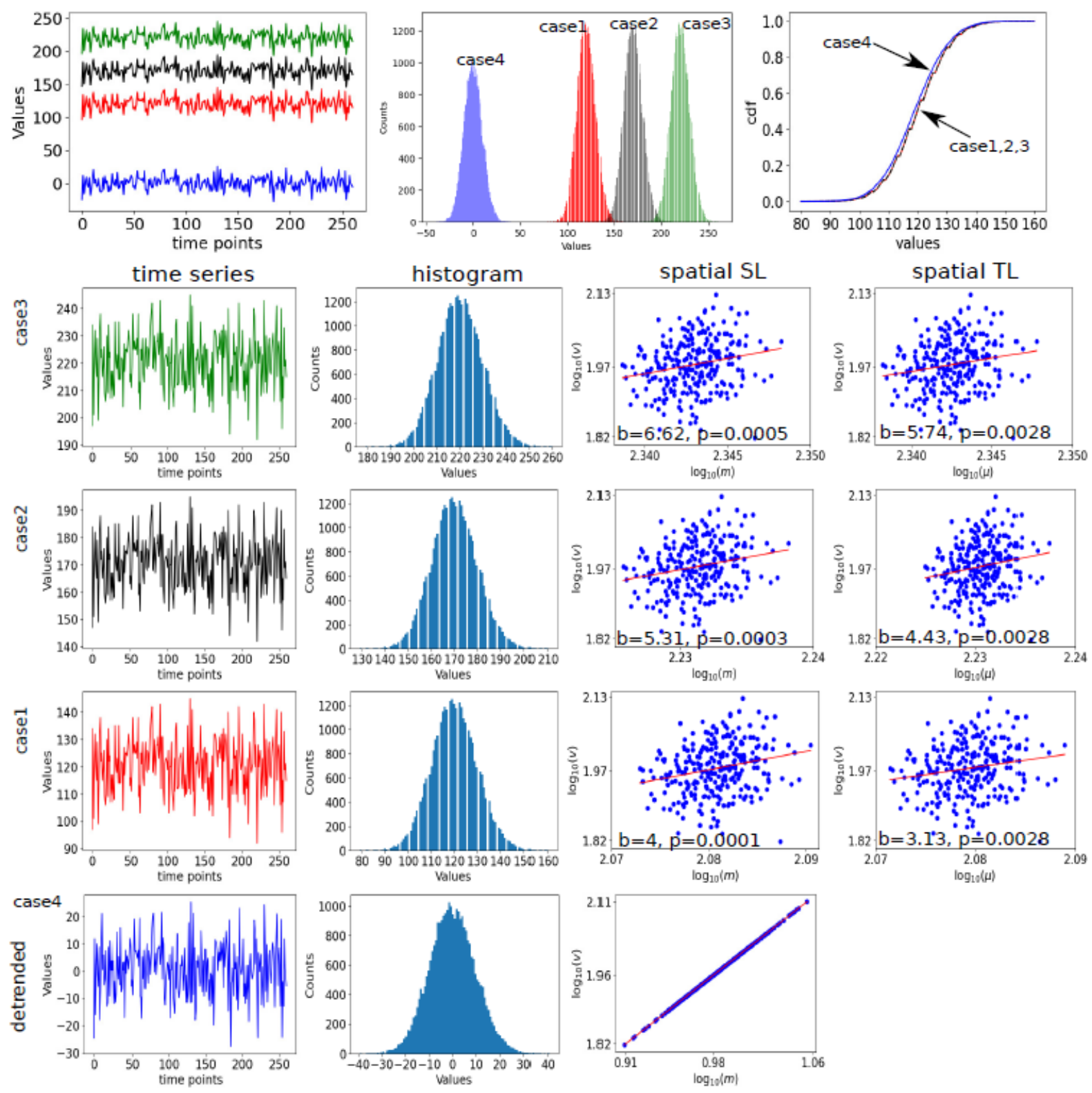

Figure S5: **Test 2 for Binomial distribution.** The Python function 'numpy.random.binomial(n=100, p=0.5, size=(261, 116))' has been used to construct the time series.

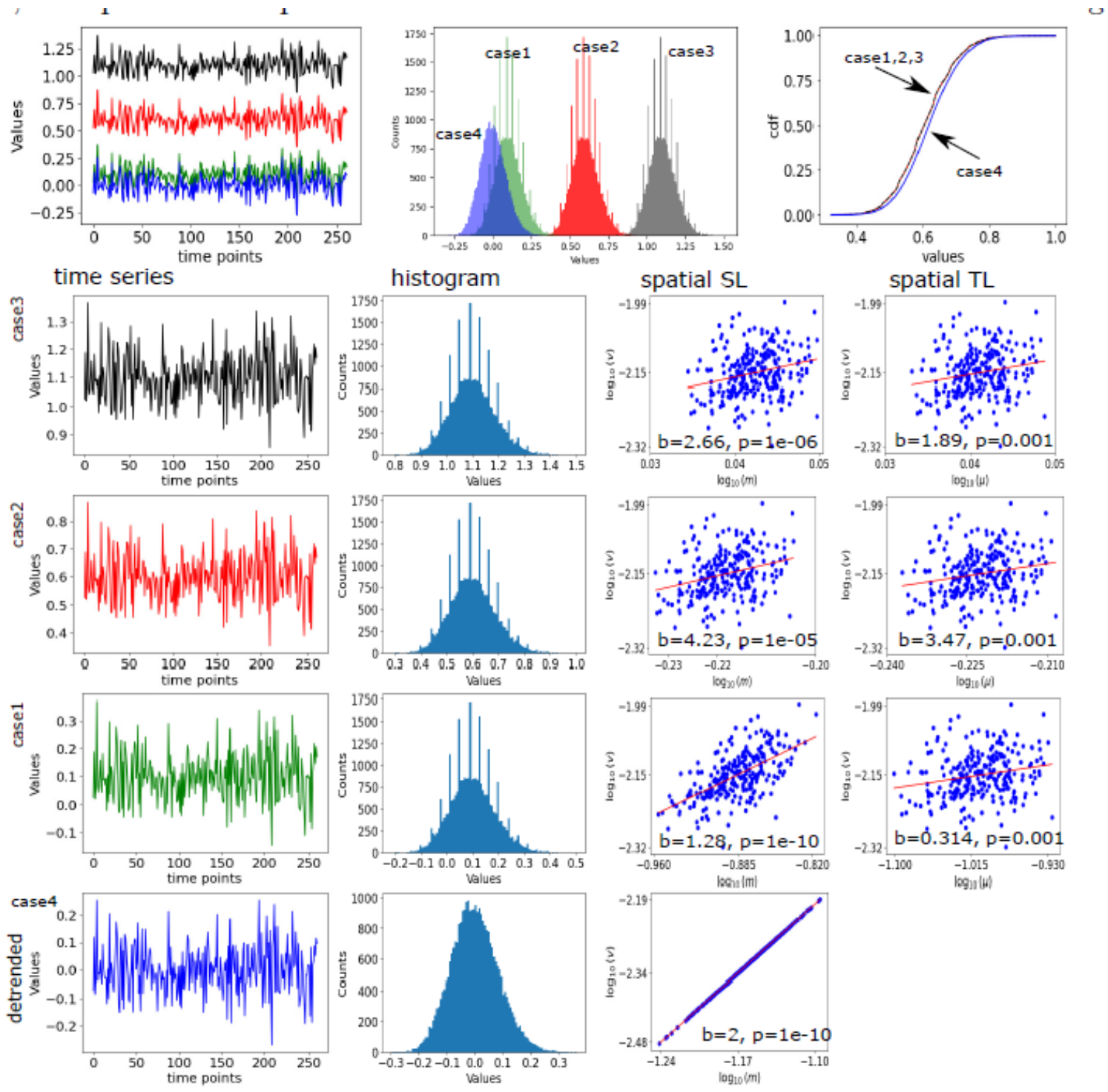

Figure S6: **Test 3 for Negative Binomial distribution.** The Python function 'numpy.random.negative.binomial(n=100, p=0.5, size=(261, 116))' has been used to construct the time series, and normalized by the maximum amplitude of each location.

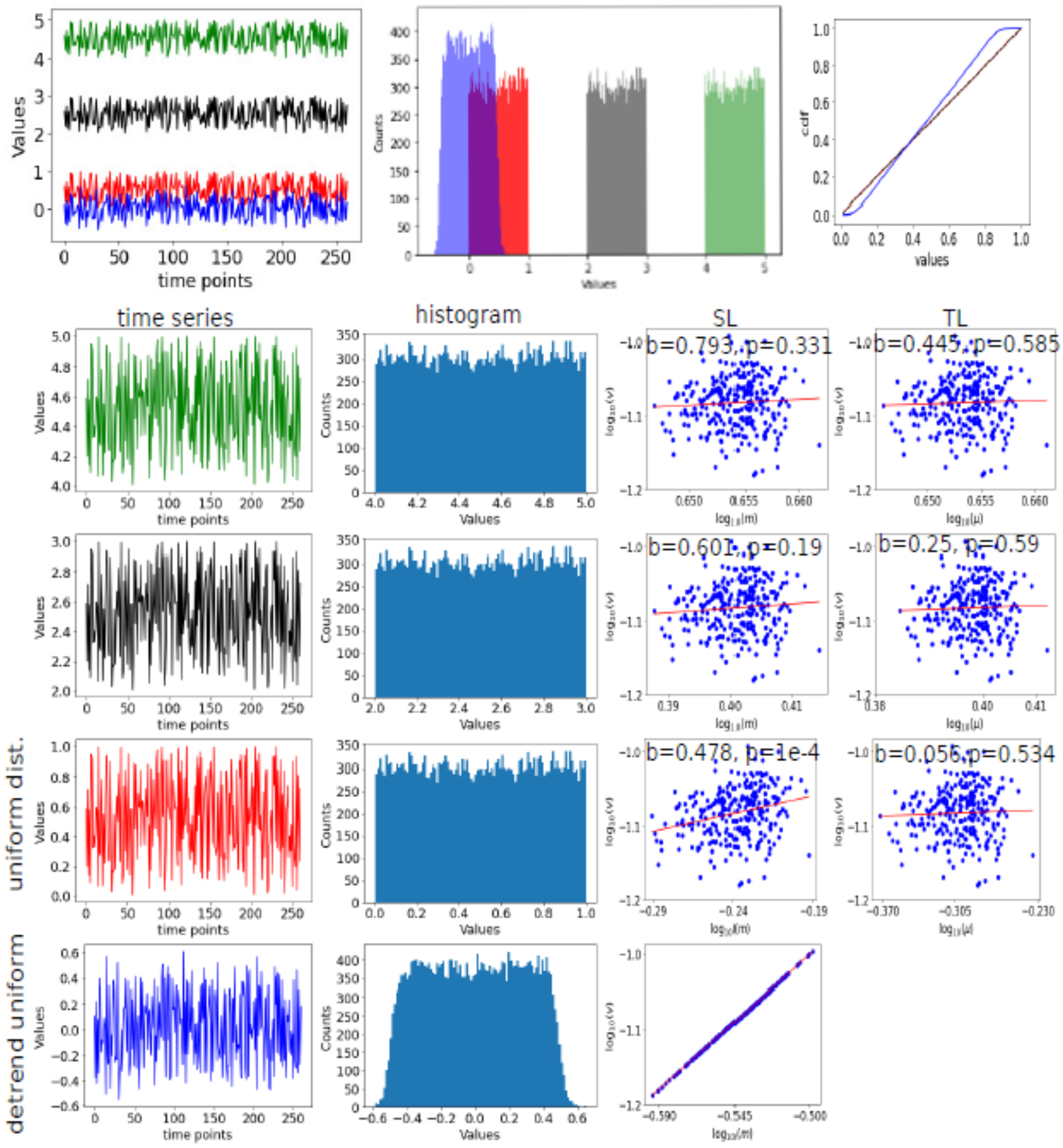

Figure S7: Uniform distribution. We have used Python built-in function 'random.uniform(0,1,[261,116])' to create the spatio-temporal data following uniform distribution, where pair-wise correlation between two locations are near zero. The temporal mean is 0.5 labeled as red. The constant values 2 and 4 are added to shift the temporal of the entire time series and generate two cases 2 and 3, corresponding colors are black and green. Detrended case is shown in blue in bottom row.

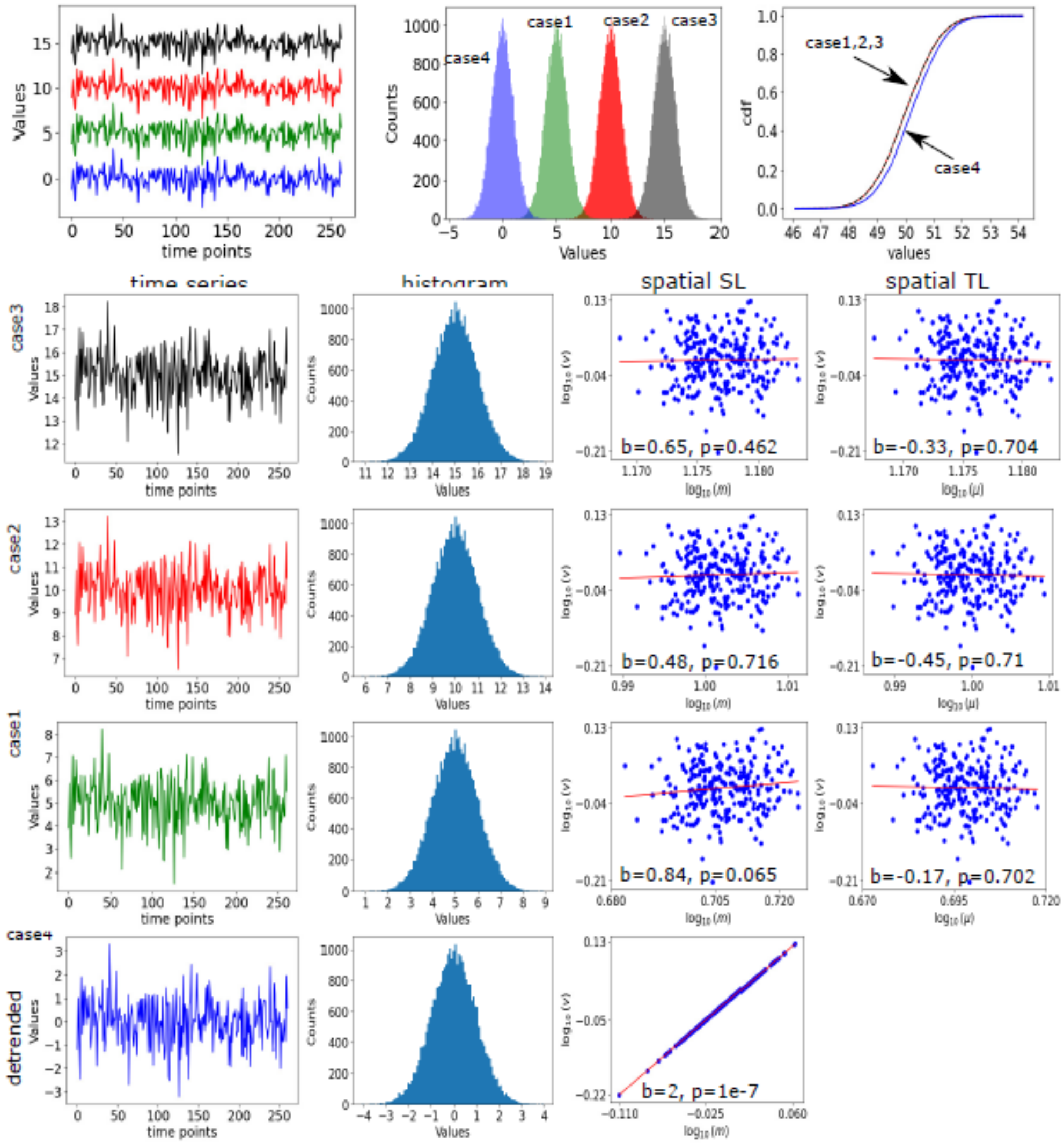

Figure S8: Normal distribution. These figures are generated by Python. The normally distributed spatio-temporal time series data was constructed using the built-in function "numpy.random.normal". The algorithm is written in Algo. 4. The spatio-temporal time series in four cases are generated by changing the mean value in the Python function, as temporal mean=0, 5, 10, 15 (blue, green, red, black) shown in fifth, fourth, third and second rows, respectively.

Reviewer Comments: In addition, the interpretation of the scaling exponent remains somewhat unclear from a biological or mechanistic standpoint. The aging and clinical findings are interesting but could be better connected to existing neuroscience literature. Clarifying these points and adding robustness checks would make the paper much stronger.

Authors' Responses: We are grateful to the reviewer for this constructive feedback, which we address point by point below.

1. Biological and mechanistic interpretation of the scaling exponent:

The spatial scaling exponent b quantifies how brain-wide hemodynamic fluctuations scale across regions, and crucially, the extent to which this scaling reflects coordinated neural activity rather than local random processes. Analytically, from Eq. (6), b is inversely proportional to synchrony Ω, which appears explicitly in the denominator. Biologically, this means that when

distributed brain regions fire in a coordinated manner — as during active sensory processing or goal-directed behavior — the collective hemodynamic activity becomes more spatially coherent, synchrony increases, and the scaling exponent decreases. Conversely, when brain regions operate more independently — as in unconstrained resting-state activity with near-zero inter-regional correlation — the exponent approaches $b \approx 2$, reflecting the purely statistical aggregation of spatially independent BOLD fluctuations. This null baseline, confirmed numerically across 645 independent realizations, represents a brain state where no collective coordination mechanism is operating — analogous to a population of neurons firing independently without any shared input or mutual coupling. The exponent, therefore, serves as a compact summary statistic for distributed coordination: a lower exponent indicates stronger brain-wide co-activation, whereas an exponent near 2 indicates near-independence across regions.

The decomposition b = b_marg + b_syn offers a further layer of biological insight. The marginal component b_marg reflects contributions from local hemodynamic properties intrinsic to each region — essentially the independent vascular and metabolic noise floor of each region. The synchrony-driven component b_syn, by contrast, isolates the scaling signature of genuine inter-regional coordination — the emergent property that arises only when spatially distributed regions co-activate in a temporally aligned manner. This decomposition is achieved through phase randomisation of surrogate time series, which destroys inter-regional correlations while preserving each region's local signal statistics. The resulting b_syn therefore captures exclusively the collective coordination fingerprint of the brain, stripped of any local noise contributions. Biologically, a large positive b_syn indicates that the brain's spatial scaling pattern is being actively shaped by coordinated network-level dynamics, whereas a near-zero b_syn indicates that spatial scaling is dominated by local, region-specific fluctuations rather than collective activity.

The monotonic inverse relationship between b and synchrony therefore provides a quantitative biological bridge: as neural encoding, stimulus processing, or task demands recruit more widespread and temporally aligned cortical activity, the scaling exponent systematically decreases, capturing the transition from segregated local processing to integrated global coordination — a distinction that is central to understanding healthy brain function and its deterioration with age or disease.

2. Connection to existing neuroscience literature:

The age-related reduction in the synchrony–scaling association slope β — progressing from young adults ($\beta = 0.7921$ during sensorimotor task) to older adults ($\beta = 0.4417$) — reflects a progressive weakening of the brain's capacity to regulate its spatial scaling properties through coordinated activity. In biological terms, increases in brain-wide synchrony drive less reorganization of spatial fluctuation patterns in older than in younger adults, consistent with a degraded coupling between large-scale network coordination and the brain's scale-free dynamics.This is consistent with Hinault et al. (2020), cited in the paper, demonstrating that disrupted neural synchrony mediates the relationship between white matter integrity and cognitive performance in older adults — structurally,

the degradation of long-range white matter connections in aging would directly reduce the capacity for spatially distributed regions to achieve temporally aligned co-activation, which our framework captures as a reduction in β. Similarly, Weistuch et al. (2021), cited in the paper, show that metabolic changes modulate network synchrony in the aging brain, providing a complementary mechanistic account — reduced metabolic efficiency in aging would limit the energetic substrate available to sustain coordinated large-scale activity, manifesting in our framework as a diminished synchrony-driven scaling signature.

The preservation of resting-state scaling relationships — where b_syn shows no significant correlation with synchrony at rest ($p > 0.005$) across age groups — has an important biological implication: intrinsic network architecture, which governs spontaneous hemodynamic fluctuations in the absence of external demands, remains relatively intact across the adult lifespan. It is specifically the brain's adaptive coordination capacity — its ability to flexibly recruit and sustain large-scale synchronized activity in response to cognitive or sensory demands — that deteriorates with age. This dissociation between preserved resting-state scaling and degraded task-evoked scaling aligns with D'Esposito et al. (2003), cited in the paper, reporting that aging effects on BOLD signals are stronger under cognitive demand than during spontaneous activity, and with Pathak et al. (2022), cited in the paper, reporting compensatory preservation of neural synchrony over the adult lifespan — our framework reveals that this compensation operates primarily at the level of intrinsic resting dynamics, while task-evoked coordination mechanisms are selectively compromised.

The subnetwork-specific findings carry distinct biological significance. The strongest synchrony–scaling associations in limbic, subcortical, and cerebellar regions reflect the integrative anatomical roles of these structures: the limbic system mediates emotional and memory-related coordination across distributed cortical areas; subcortical structures, particularly the thalamus and basal ganglia, act as central hubs routing coordinated activity between cortical regions; and the cerebellum, beyond its classical motor role, is increasingly recognized as a coordinator of timing and prediction signals across large-scale networks. Their strong synchrony–scaling coupling therefore reflects their biological role as integrative nodes whose coordinated activity shapes brain-wide fluctuation patterns. The visual network's task-specific enhancement during movie watching directly reflects the strong visual processing demands of naturalistic film stimuli, recruiting coordinated activity specifically within visual cortical areas. The frontoparietal network's comparatively stronger resting-state association is consistent with its established role in domain-general executive control — a network that maintains a degree of coordinated baseline activity even during unconstrained cognition.

For the clinical findings, the disrupted synchrony–scaling coupling in ADHD — with profoundly reduced β confirmed across 1000 independent realizations — has a clear biological interpretation: in ADHD, the brain's large-scale coordinated activity, while potentially present, fails to reshape spatial fluctuation scaling in the manner observed in healthy adults. This suggests not merely a reduction in synchrony per se, but a fundamental decoupling between network-level

coordination and the scale-free organization of brain-wide dynamics — a deeper disruption than what is captured by conventional functional connectivity measures, which only quantify the strength of inter-regional correlations without assessing how those correlations shape the brain's scaling architecture. This is consistent with Mathalon and Sohal (2015), cited in the paper, on neural oscillations and synchrony in brain dysfunction, and King et al. (2018) on temporal synchrony differences in individuals with autism, suggesting that disrupted synchrony–scaling coupling may be a transdiagnostic signature of conditions characterized by atypical large-scale coordination, including autism spectrum disorder, epilepsy, and schizophrenia.

3. Robustness checks:

The paper reports multiple layers of robustness validation. First, the synchrony–scaling relationship is validated using two independent analytical methods — a semi-analytical approach based on Eq. (6) and a fitting-based linear regression approach — producing consistent results across all brain states and age groups as shown in Figs. 4(J–M) and 5(J–K). Second, biological consistency is verified across 100 independent realizations for lifespan analyses and 1000 independent realizations for the clinical ADHD comparison, with error bars reflecting variation across trials. Third, cross-cohort replication is demonstrated: the CamCAN findings (N = 645) are independently replicated in the NKI/Rockland dataset (N = 89), showing a significant clockwise rotation of β with age consistent with the primary cohort, confirming that the age-related deterioration of synchrony-driven scaling is a generalizable biological phenomenon rather than a dataset-specific artifact. Fourth, the biological generalizability of the framework across different neural signal distributions is confirmed numerically across Poisson, negative binomial, uniform, and gamma distributions, demonstrating that the synchrony–scaling relationship is a property of the coordination structure itself rather than the specific statistical distribution of BOLD signals. Fifth, comparison with established neurobiological metrics — metastability, entropy of functional connectivity dynamics, and the Kuramoto order parameter — all show consistent patterns of minimal age-related changes at rest but significant alterations during movie-watching and sensorimotor tasks, confirming that the synchrony-induced spatial scaling results reflect genuine age-related reorganization of brain dynamics captured consistently across multiple independent measures of neural coordination.

Reviewer #2:

Reviewer Comments: The authors propose a generalization of Taylor’s law to signed multivariate time series data by replacing the mean with the root-mean-square of the detrended data. The authors demonstrate with numerical simulations that the scaling exponent of this generalized spatial Taylor’s law is inversely related to what the authors call “synchrony”. The latter is the averaged (Pearson) correlation matrix (or averaged functional connectivity matrix). The authors investigate the synchrony-scaling relationship (sometimes also referred to as “coupling”) using functional MRI data recorded under various conditions, thereby replicating earlier findings

achieved with other, comparable similarity metrics used in neuroimaging. Eventually, the authors claim potential clinical and translational utility of their generalized scaling metric.
This manuscript is not convincing in its present form. It raises more questions than giving answers.

Authors' Responses: We are grateful for your careful observations, critique, and insightful comments. We have addressed each point and worked to make the manuscript more accessible to a general readership.

Reviewer Comments: **Quite often do the authors make some quite general statements that either lack more detailed information or are not supported by the presented findings. Here's a quite extensive but definitely incomplete list of phrases that might raise a red flag for the reader: In the context of brain dynamics, what would be the advantage of knowing how the population variance links to the sample mean? What can we learn from that? Why is it important to investigate the effect of synchrony on spatial scaling or spatial fluctuation scaling?**

Authors' Responses: We thank the reviewer for raising this important point. The proposed metric, the spatial scaling law, was obtained from the sample variance-mean relationship, or the variability in the oscillatory nature of the coordinated brain dynamics.The scale-invariant property of the neural dynamics reveals deep functional organizing principles of the coactivation patterns of the brain-wide coordinated neuronal activity driven fluctuation scaling extends our ability to interpret ubiquitous power-law signatures in neuroimaging, within a unified theoretical framework that relates them to healthy brain aging and brain disorders at the resting state and also during active and passive tasks.

The relationship between population variance and the sample mean — the spatial Taylor's law — is not merely a statistical curiosity. In the context of brain dynamics, it provides a compact, model-free index of how neural fluctuations scale across cortical and subcortical regions. Applied to whole-brain fMRI BOLD signals, this variance–mean scaling exponent encodes information about the brain's underlying coordination architecture. Specifically, a higher exponent indicates that variance grows disproportionately relative to the mean level of neural activity, reflecting more heterogeneous, segregated dynamics, whereas a lower exponent implies more homogeneous, integrated activity across regions — a distinction that is directly informative about functional organisation. Crucially, because the exponent can be computed from a single fMRI recording without model fitting or parcellation-specific assumptions, it offers a practical and generalizable neuroimaging marker. For example, our results show that it changes systematically with healthy aging and is disrupted in ADHD, underscoring its translational relevance. Please also see our detailed response to Reviewer 1 regarding the biological and mechanistic interpretation of the scaling exponent, its connections to the neuroscience literature, and the robustness of these findings

Reviewer Comments: **Can the authors provide more examples about complex systems – other than the brain – "with integrated and segregated functional networks"?**

Authors' Responses: Thank you for pointing this out. We have now modified the abstract in the revised version of the manuscript.

Original: *"Whether other complex systems with integrated and segregated functional networks"*

Revised: "Whether other complex systems with integrated and segregated network topology…"

Below is a table showing a few relevant references on integrated and segregated complex networks in multiple disciplines.

Disciplin e	***Segregated & Integrated networks***	***Key references***
Ecology	Isolated food webs; habitat patches.<br>Apex predators bridging ecosystems; multinetwork webs.	Ings, T. C., Montoya, J. M., Bascompte, J., Blüthgen, N., Brown, L., Dormann, C. F., …, & Woodward, G. (2009). Ecological networks–beyond food webs. Journal of animal ecology, 78(1), 253-269.<br><br>Olff, Han, et al. "Parallel ecological networks in ecosystems." Philosophical Transactions of the Royal Society B: Biological Sciences 364.1524 (2009): 1755-1779.
Economic s	Regionally isolated stock markets (China, India).<br>Western financial core; global contagion pathways.	Raddant, Matthias, and Dror Y. Kenett. "Interconnectedness in the global financial market." Journal of International Money and Finance 110 (2021): 102280.<br><br>Berndsen, Ron J., Carlos León, and Luc Renneboog. "Financial stability in networks of financial institutions and market infrastructures." Journal of Financial Stability 35 (2018): 120-135.
Social Science	Ethnically/socially homogeneous communities.<br>Cross-group social ties; diverse urban	Henry, Adam Douglas, Paweł Prałat, and Cun-Quan Zhang. "Emergence of segregation in evolving social networks." Proceedings of the National Academy of Sciences 108.21 (2011): 8605-8610.

Disciplin e	***Segregated & Integrated networks***	***Key references***
	mobility.	Henry, Adam Douglas, Paweł Prałat, and Cun-Quan Zhang. "Emergence of segregation in evolving social networks." Proceedings of the National Academy of Sciences 108.21 (2011): 8605-8610.
Physics	Regular lattices (high clustering, long paths).<br>Random graphs (short paths, low clustering).	Watts & Strogatz (1998), Collective Dynamics of 'Small-World' Networks, Nature 393.6684 (1998): 409-410. Most real-world networks lie somewhere between two extremes: completely regular (highly segregated, locally clustered lattices) and completely random (integrated, short-path random graphs).
Engineeri ng	Modular power grid communities (local stability).<br>Cross-community grid ties (global stability / risk).	Dylewsky, D., Yang, X., Tartakovsky, A., & Kutz, J. N. (2019). Engineering structural robustness in power grid networks susceptible to community desynchronization. Applied Network Science, 4(1), 24.<br><br>Schwarz, Sebastian, Subham Sahoo, Mirko Stoffers, Patrick Pollok, Leonardo Pompe, Arthur M. Fibich, Amila Kaharević et al. "Resilient low-inertia power systems through asynchronous energy balancing." Nature Reviews Electrical Engineering (2026): 1-10.

Reviewer Comments: Exactly what can one learn about "deep organizing principles" from scale-invariant fluctuations?

Authors' Responses: This is related to your earlier comments, to which we tried to provide a detailed response. We intended to describe the degree of criticality in large-scale brain dynamics, together with the hierarchical and multiscale functional organisation of neuronal activity, unified through scale-dependent theories of brain function. We have revised the text accordingly. The revised passage appears in the Introduction **(lines 127–131)**:

'Complex systems often exhibit scale-invariant fluctuations [Taylor, 1961; Reuman et al., 2017; Cohen and Xu, 2015; Yang et al., 2022; Šuvakov and Tadić,

2006], as also reported in the human brain [Munn et al., 2024; D'Angelo and Jirsa, 2022; Tagliazucchi et al., 2012; He et al., 2010] — yet we lack a unifying law connecting such variability to the coordinated dynamics of the human brain, particularly across different mental states, healthy aging, and neurological disorders.'"

Reviewer Comments: Fluctuations of what? Why at all would one expect that "large-scale synchrony shapes brain-wide variability" and what would one learn from that?

Authors' Responses: We thank the reviewer for pushing us to articulate the importance of the proposed metric more clearly.

Fluctuation refers to the variance of a set of observations or spatio-temporal data — that is, the variability in the amplitude of the data around its mean. In a complex system, the elements that participate in a given dynamical process are each active to differing degrees, and the fluctuation in an element's activity typically grows monotonically with its average activity. This relationship takes the form *fluctuation = constant × average^α*, commonly known as fluctuation scaling, where α is the scaling exponent. In our empirical examples, the fluctuations refer specifically to spatial fluctuations in the amplitude of detrended BOLD hemodynamic signals across brain regions at each time instant, quantified by the spatial variance (v). The synchrony-induced spatial scaling law offers a theoretically grounded, compact summary statistic of brain-wide coordination that complements existing metrics. Specifically:

- It encodes both spatial variability and inter-regional coordination in a single scalar exponent, where synchrony, measured as the temporal variability of the spatial mean of BOLD signals across brain regions, drives the scaling exponent analytically as shown in Eq. 3.
- It is sensitive to brain state, remain stable at rest but alters significantly during task-evoked states, revealing distinct functional reorganising principles of spontaneous versus task-driven brain dynamics;
- It tracks healthy aging trajectories, with the synchrony-scaling association declining progressively from young to older adults, particularly during cognitively demanding tasks; and

It is disrupted in ADHD, highlighting its clinical potential as a functional-MRI marker. Together, these properties make the synchrony-induced spatial scaling law a useful and interpretable marker of brain-wide hemodynamic coordination across the lifespan and in neurological disorders, complementing conventional measures such as functional connectivity, the Kuramoto order parameter, dynamic FC, and metastability.

Reviewer Comments: What would be the advantage or reading out "coordinated dynamics [...] from scale-free variability in a principled way"? Aren't there other (possibly easier) ways to do so? Exactly what would be

the advantage of having "synchrony-induced scaling laws as a compact general marker for complex neural systems"?

Authors' Responses: The significance of this metric lies in its capacity to capture the variability of oscillatory brain dynamics, effectively linking statistical measurement to coordinated neural activity. Importantly, it also disentangles random background processes from the meaningful, stimulus-driven coordination seen across the whole brain.

Several existing approaches capture aspects of neural coordination, but each carries notable limitations. Temporal autocorrelation methods [Shinn et al., 2023] and temporal scaling laws [Tomasi et al., 2017] operate on single-location signals and therefore miss the brain-wide coactivation structure that emerges from multivariate dynamics. Functional connectivity matrices capture pairwise coordination but are high-dimensional, computationally intensive, and do not directly yield a compact scalar summary that scales with system size or brain regions.

Detrended fluctuation analysis, while widely used in climatology and neuroscience, has not previously been extended to characterize how spatial aggregation of detrended signals relates to synchrony. Our approach addresses precisely this gap by replacing the sample mean with the spatial RMS; we derive a single closed-form exponent that encodes synchrony without requiring pairwise computation across all regions.

The key advantage is parsimony combined with generality. Rather than summarizing brain coordination through hundreds of pairwise FC values or region-specific metrics, the spatial scaling exponent condenses brain-wide coordinated variability into a single interpretable parameter. Critically, this parameter:

(i) Has a direct analytical link to the degree of synchrony, making it theoretically grounded rather than empirically heuristic.

(ii) Remains stable at rest but reorganizes during naturalistic tasks, demonstrating sensitivity to functionally meaningful state changes.

(iii) Tracks age-dependent alteration across the adult lifespan.

(iv) Is selectively disrupted in neuropsychiatric conditions.

To our knowledge, no existing single scalar metric meets all of these criteria at once while also linking to a well-established universal scaling framework (Taylor's law) that generalises across ecology, physics, and economics. The marker is thus not merely convenient but analytically motivated, demonstrated, and empirically validated on large-scale fMRI data with a substantial sample across conditions.

Reviewer Comments: The authors say that "multivariate analysis" (of exactly what?) "significantly enhances the sensitivity of human neuroscience data […]." Sensitivity to exactly what?

Authors' Responses: We thank the reviewer for this precise observation. We have now revised "multivariate data analysis" in the main text.

This statement refers to the well-established finding that multivariate analysis of distributed blood-oxygen-level-dependent (BOLD) activity — across many cortical and subcortical regions simultaneously — yields greater discriminative power for decoding spontaneous brain activity, task conditions, and individual differences than univariate, single-region approaches [Kamitani and Tong, 2005; Haynes and Rees, 2005; Kriegeskorte et al., 2006]. In our framework specifically, the spatial scaling law operates on the full multivariate BOLD time series across all parcellated regions at once, capturing brain-wide coordinated dynamics that single-region temporal scaling [Tomasi et al., 2017] or pairwise connectivity measures necessarily miss. The enhanced sensitivity referred to here is specifically the sensitivity to synchrony-driven changes in scaling behaviour across age groups, task conditions, and clinical populations — changes that would be difficult to detect with univariate approaches, given the collective, distributed nature of the phenomenon being measured.

Reviewer Comments: What exactly are the underlying biophysical (coordination) mechanisms, that the authors often refer to in their manuscript?

Authors' Responses: By "biophysical coordination mechanisms", we mean the physiological processes that cause spatially distributed brain regions to fluctuate coherently rather than be governed by any random processes.

Please see the *Introduction* section, **lines 136–140**:

Original: *"biophysical coordination mechanisms"*

Revised: "the contributions coming from internal or external stimulus driven effects in neuroscience or Moran effect in ecology rather than any random processes"

Our synchrony measure — the temporal variance of the spatial mean of the multivariate BOLD time series — provides a scalar index of how correlated activity is across the brain, and is therefore sensitive to the joint action of these mechanisms. Our analytical framework links this index to the spatial scaling exponent b: as synchrony increases, region-wise variability decreases relative to the RMS amplitude of the signal, which lowers b (Eq. 3). Within this framework, the age-related decline in b and its disruption in ADHD are consistent with altered thalamocortical and network-level coordination, rather than being purely statistical artifacts. We have clarified this interpretation in the revised manuscript.

Reviewer Comments: Can the authors provide an estimate as to how much of the synchrony investigated is indeed emergent and how much is induced/evoked -- either due to intrinsic/external stimuli or due to possible spatial oversampling?

Authors' Responses: The reviewer raises three distinct sources of synchrony — emergent, evoked/induced, and apparent synchrony from spatial oversampling. We address each in turn.

1. **Emergent vs. evoked synchrony.** During resting state, where no external stimulus drives co-activation, the synchrony–scaling relationship is stable, indicating that the synchrony observed at rest is predominantly *emergent*, arising from intrinsic network dynamics. During naturalistic tasks, the relationship shifts systematically (and in an age-dependent manner), indicating an additional *evoked* component superimposed on this emergent baseline. We can therefore treat the resting-state value as an estimate of the emergent contribution and the rest-to-task change as an estimate of the evoked contribution.

The synchrony-scaling association β has been utilized to characterize the spontaneous or emergent activity, and task-evoked activity, which are quantified and presented in Figs. 4 and 5. Specifically, Fig. 4 shows, in spontaneous activity, the synchrony-scaling association is β=0.1608, p-value>0.005; and in task-evoked activity, β=0.3866 for sensorimotor task, and β=0.3784 for movie watching, in both cases, p-value<0.005. For group-wise shown in Fig. 5, the association slope β=0.1481-0.3701 for spontaneous activity; and in task-evoked activity, β=0.4402--0.7921 for sensorimotor task, and β=0.2728-0.5759 for movie watching. These quantification derived from the BOLD fluctuations help capture age-related changes in brain-wide coordination dynamics across spontaneous and task evoked neural activities due to internal and external stimuli.

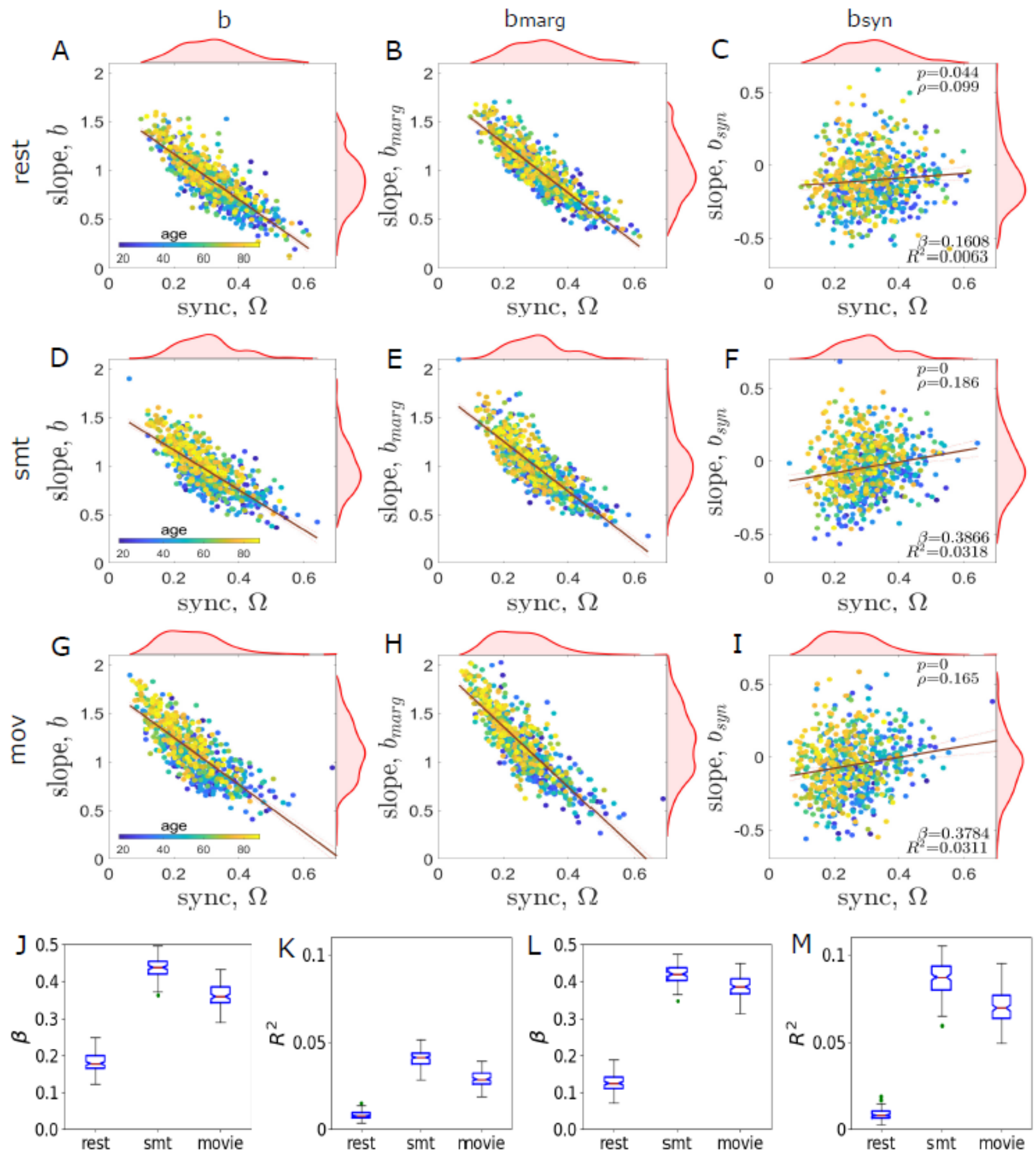

Figure 4: **Spatial SL exponent against synchrony for different brain states.** (A-C) resting state, (D-F) sensorimotor task, and (G-I) movie watch. (A,D,G) Scatter plots (colored circles) and linear regression fit (red lines) between b and Ω in three brain states. (B,E,H) Contributions of marginal distributions, slope b_{marg} against Ω. (C,F,I) Synchrony-driven SL slope (b_{syn}) after removing marginal effects. Statistical significance is considered when $p < 0.005$. (J,L) The association between b_{syn} and Ω is captured by β, together with goodness of fit R^2 in (K,M) for 100 realizations. Results are shown for semi-analytical (J,K) and fitting-based analysis (L,M).

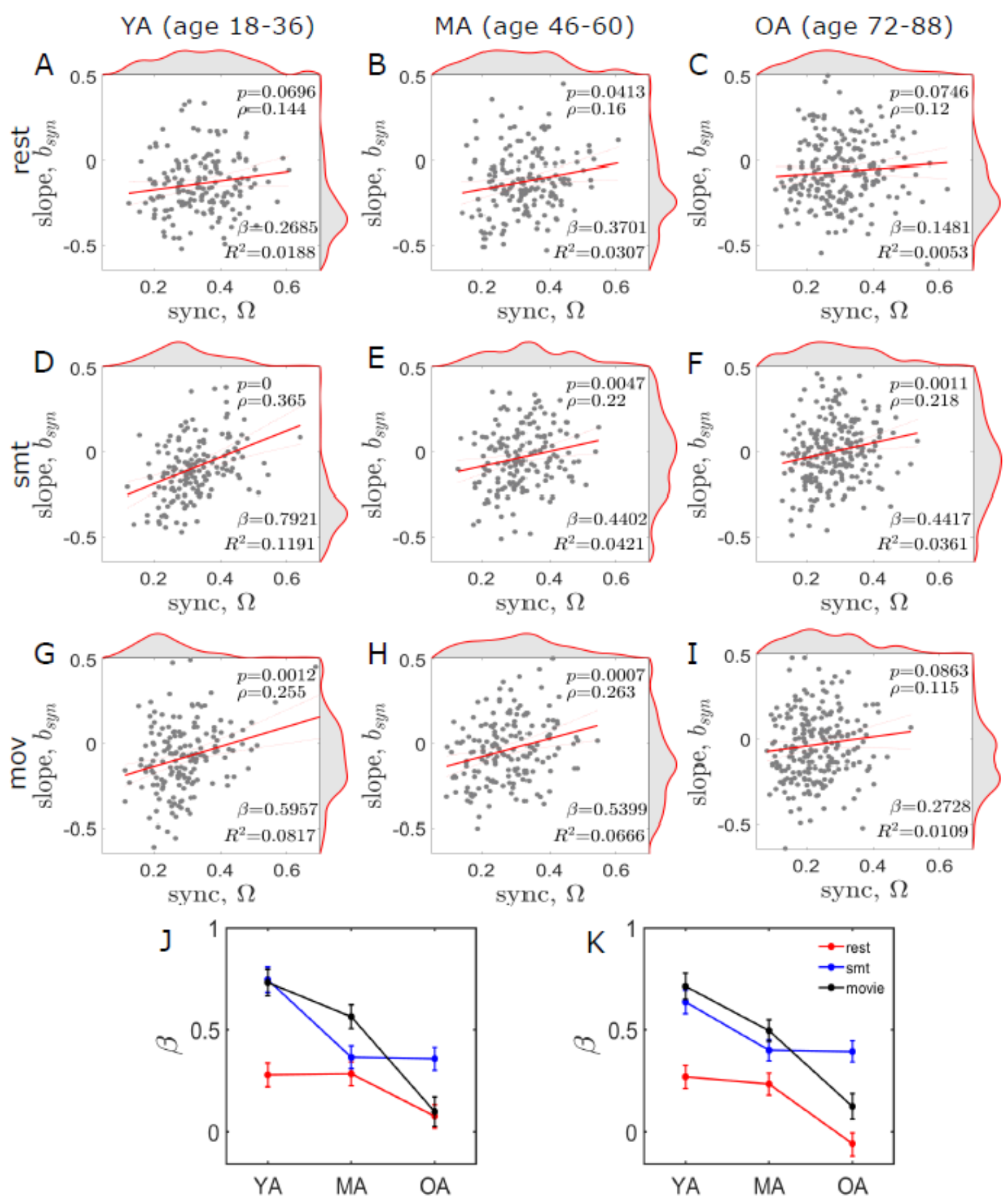

Figure 5: **Group-wise age-related alterations in the spatial exponent.** Associations between the spatial SL (b_{syn}) and average synchrony (Ω) are shown for three age groups in rest (A-C), smt (D-F) and mov (G-I) states. (A,D,G) In young adults (YA), the slope (β) remains positive and significant in all three conditions. In middle-aged adults (shown in (B,E,H)), β rotates in clock-wise direction compared to young adults, and (C,F,I) rotates further in clock-wise direction in older adults. The slope β shows a tendency to rotate clockwise with increasing age in all three brain states. (J,K) Association between Ω and b_{syn} is monitored by the slope β for three age groups in three brain states, where left and right plots are obtained from the semi-analytically derived and fitting-based SL slopes, respectively. Consistency in β is checked by 1024 independent realizations, i.e., randomization of phases of the BOLD signals. The error bars indicate variations over the trials. $p<0.005$ implies statistical significance.

2. Physiologically induced (artifactual) synchrony. To guard against synchrony introduced by non-neural physiology, our analysis uses detrended BOLD signals. Detrending removes temporal means and low-frequency trends, suppressing slow physiological drifts and scanner-related artifacts that would otherwise inflate synchrony estimates. The synchrony we report, therefore, reflects coordinated signal fluctuations rather than shared slow nuisance trends.

3. Spatial oversampling. If the observed synchrony were an artifact of spatial oversampling, it should inflate synchrony roughly uniformly across the brain. Instead, the synchrony–scaling relationship is network-selective: it is higher in limbic, subcortical, and cerebellar subnetworks rather than being uniform across all subnetworks. Also, the visual network shows an elevated synchrony–scaling relationship specifically during movie watching relative to the other brain states. This stimulus-dependent spatial specificity is the signature of a neural, state-driven effect, not of a uniform spatial oversampling artifact.

Reviewer Comments: After all, exactly what is the added value of the presented approach to understanding the coordinated brain dynamics across

the human lifespan or for conditions marked by disrupted neural coordination?

Authors' Responses: We thank the reviewer for this important question. The added value of our approach is multi-fold:

1. ***Theoretical extension:*** Existing scaling laws, including Taylor's law, were developed exclusively for non-negative data and cannot be applied to detrended neural signals spanning both positive and negative values. Our generalized spatial scaling law — replacing the sample mean with the spatial RMS — directly resolves this limitation. Critically, the results section confirms that irrespective of the original distribution of measurements (Poisson, negative binomial, uniform, gamma), after the detrending operation the data follow a normal distribution, establishing a unified Gaussian-like framework for fluctuation analysis across all marginal distributions.

2. ***Disentangling random, stimulus-driven, and coordinated dynamics:*** The framework operates at two levels of disentanglement. First, the detrending operation explicitly removes temporal means and trends, isolating intrinsic fluctuation structure from stimulus-evoked components while preserving spectral and correlation properties. Second, the scaling exponent is decomposed as b = b_marg + b_syn using phase randomized surrogate time series, explicitly separating contributions of random marginal distributions (b_marg) from genuine synchrony-driven coordination (b_syn). Under near-zero synchrony ($\rho \approx 0$), the spatial scaling exponent approaches $b \approx 2$ — confirmed both analytically via Eq. (6) and numerically across 645 independent realizations — establishing this as the null baseline of purely stochastic behavior. Synchrony-induced deviations from this null therefore unambiguously reflect genuine coordinated dynamics rather than artifacts of the underlying signal distribution or external drive. The observation that b_syn shows no significant correlation with synchrony at rest ($p > 0.005$) but significant positive correlations during sensorimotor ($\beta = 0.3866$, $R^2 = 0.0318$, $p < 0.005$) and movie-watching tasks ($\beta = 0.3784$, $R^2 = 0.0311$, $p < 0.005$) directly confirms that the framework successfully separates intrinsic from task-evoked coordination.

3. ***Lifespan sensitivity with a unified compact metric:*** Applied to the CamCAN cohort (N = 645, ages 18–88), the synchrony-scaling association slope β reveals a progressive clockwise rotation across three age groups — young adults (18–36), middle-aged adults (46–60), and older adults (72–88) — in all three brain states. This age-related attenuation is strongest during sensorimotor tasks (β declining from 0.7921 in YA to 0.4417 in OA), moderate during movie watching (β from 0.5957 in YA to 0.2728 in OA), and weakest at rest, a pattern replicated independently in the NKI/Rockland cohort (N = 89). This sensitivity to both task condition and age is not achievable by conventional single-region temporal scaling or autocorrelation approaches, which lack the multivariate spatial sensitivity of the present framework.

4. ***Subnetwork specificity:*** The synchrony–scaling association is not uniformly distributed across the brain. Limbic, subcortical, and cerebellar regions exhibit

the strongest synchrony–scaling associations across all three brain states, while the visual network shows robust associations specifically during movie watching, and frontoparietal networks show comparatively stronger effects at rest. This network selectivity demonstrates that the framework captures biophysically meaningful spatial heterogeneity in coordinated dynamics, rather than a global artifact.

5. ***Clinical utility:*** In the UCLA-CNP dataset (N = 106), individuals with ADHD (N = 40) show a profoundly reduced association slope β compared to healthy controls (N = 66), confirmed across 1000 independent realizations ($p > 0.05$ for synchrony-induced scaling in ADHD). This disruption in synchrony–scaling coupling demonstrates that the metric is sensitive to neuropsychiatric conditions in a way that goes beyond conventional functional connectivity measures, which do not capture how coordination strength shapes scale-free variability patterns. The framework is therefore extensible to other disorders of atypical large-scale synchrony, including autism spectrum disorder, epilepsy, and schizophrenia.

Reviewer Comments: What exactly are the mechanistic insights (beyond those afforded by conventional connectivity measures), and exactly why do the authors think that an application of their analysis approach to some fMRI data validates the generalized scaling law?

Authors' Responses:

On the mechanistic insight beyond conventional connectivity. Functional connectivity (FC) quantifies pairwise co-variation between regions but does not describe how regional variability scales with collective, brain-wide amplitude. Our scaling exponent b characterizes exactly this relationship across the whole brain simultaneously. The key analytical result is that b is governed by the ratio of synchronized variance to total variance — a quantity that pairwise FC does not expose. This has a concrete consequence: two datasets with identical mean FC can yield different scaling exponents if their variance is partitioned differently between shared and idiosyncratic fluctuations. The exponent therefore captures coordination structure that is invisible to standard connectivity analysis. Moreover, the inverse synchrony–scaling relationship is a *derived theoretical prediction* rather than a post-hoc correlation, which gives the framework generative leverage that descriptive FC measures lack and connects directly to the coordination mechanisms discussed in our response above.

On what the fMRI application does and does not establish. We want to be precise here: the fMRI results are not offered as *proof* of the generalized scaling law. The law's validity is established theoretically through analytical derivation and through simulations spanning multiple distributions (Poisson, negative binomial, uniform, gamma). The role of the fMRI application is twofold: (i) to show that the law holds empirically in real neural data, and (ii) to test its central theoretical prediction — the inverse synchrony–scaling relationship — which is reproduced consistently across three large, independent cohorts (total N = 840) spanning diverse age groups and task conditions. In other words, theory and

simulation establish that the law *should* hold and why; the empirical data confirm that it *does* hold in the brain and that its core prediction survives out-of-sample across cohorts. It is the convergence of analytical, simulation, and multi-cohort empirical evidence — not the data fit alone — that supports the framework.

On generalized scaling law: The theoretical extension allowed us to examine the scaling behaviour in multivariate data spanning from negative to positive values, previously inaccessible through spatial Taylor's law. Thereby, the term "generalized" is therefore used to describe the revised scaling law's ability to analyse beyond non-negative values in a broader class of data.

Reviewer Comments: Apart from synchrony, can the authors image other factors that may lead to similar findings?

Authors' Responses: The scaling exponent b is influenced by synchrony and marginal distributions. It is not an empirical regularity that competing factors could independently reproduce. Factors such as regional noise heterogeneity, card spatiotemporal autocorrelation, scanner drfits, head motion could potentially contribute to the marginal distributions, which may influence b, along with the effect of the synchrony. The reason we have decomposed the exponent *b* into two parts, b_syn and b_marg, which help remove contributions of marginal distributions due to the random processes from the internal or external stimuli driven effects, what makes the scaling exponent a principled, theory-grounded measure of coordination rather than a composite of confounded influences.

Reviewer Comments: Quite often do the authors appear to mix the terms synchrony and synchronization, despite their different meanings.

Authors' Responses: We have carefully revised the manuscript and used 'synchrony' to ensure terminological consistency. The global metric of coherence is mentioned as "average spatial synchrony, or average synchrony" to avoid any confusion.

Reviewer Comments: Given different acquisition parameter (e.g. TE/TR times) for the investigated conditions and between datasets, can the authors exclude an influence of these parameters?

Authors' Responses: We thank the reviewer for raising this. We cannot claim that TE/TR have *no* effect on the raw BOLD signal — they necessarily affect contrast weighting and temporal sampling — but we provide both structural and empirical reasons why the synchrony–scaling result is not driven by these parameters.

By construction, the exponent is a relative quantity. The scaling exponent b is the slope of the log(variance)-versus-log(RMS) relationship computed across brain regions. Because it is a slope in log–log space, it is dimensionless and insensitive to any global multiplicative rescaling of the signal: a uniform change in amplitude (such as that introduced by a different TE) shifts the intercept of the regression but leaves the slope unchanged. The slope is likewise largely insensitive to the number of regions, the spatial sampling, the analysis window, and the temporal sampling rate.

Preprocessing removes the slow nuisance channels. The analysis operates on detrended BOLD signals; detrending removes temporal means and low-frequency drifts, suppressing the slow scanner- and physiology-related trends through which acquisition differences would most plausibly bias the fluctuation estimates.

Dedicated controls isolate the coordination-driven component. The phase-randomization decomposition $b\_syn = b - b\_marg$ separates synchrony-driven scaling from the marginal (per-region) contribution, so any residual region-level signal bias is absorbed into b_marg rather than into the synchrony term. The synchrony measure Ω is normalized, making it robust to overall amplitude differences across scanners.

Empirically, the effect replicates across different acquisition setups. This is the most direct evidence. CamCAN (N = 645, ages 18–88) is our primary cohort and establishes the lifespan trajectory of the synchrony–scaling slope β across brain states [Shafto et al., 2014; Taylor et al., 2017]. NKI/Rockland (N = 89, ages 18–83) — acquired on a different scanner with a different protocol and an independent sample — independently reproduces the *direction* of the effect: β declines significantly from younger to older adults, matching CamCAN. We treat this as replication of the effect's direction rather than a quantitative cross-dataset comparison. The fact that the age-related attenuation survives a change of scanner and protocol indicates that it is not an acquisition artifact but a genuine, biologically grounded phenomenon. We have noted these points in the Discussion.

Reviewer Comments: Likewise, the sizes of the different subnetworks are different. Has this possible influence been controlled for?

Authors' Responses: Yes — subnetwork size is controlled for, both by construction and empirically.

By construction. The spatial RMS and spatial variance (Eqs. 1 and 3) are each normalized by the number of regions n, so the scaling exponent b (Eq. 6) — the slope between two size-normalized quantities in log–log space — does not depend on the absolute size of a subnetwork. The synchrony measure Ω (Eq. 5) is an average of pairwise correlations over all region pairs, i.e., a *density* of coordination rather than a sum, and is therefore directly comparable across

subnetworks of different sizes. Consequently, the association slope β (the regression of b_syn on Ω) relates two already-normalised quantities and is itself scale-free with respect to subnetwork size.

Spatial RMS is derived by

$$m(t) = \sqrt{\frac{1}{n}\sum_{i=1}^{n} Y_i^2(t)}. \quad (1)$$

,

Spatial mean

$$\mu(t) = \frac{1}{n}\sum_{i=1}^{n} Y_i(t), \quad (2)$$

Spatial Variance

$$v(t) = \sum_{i=1}^{n} Y_i^2(t)/(n-1) - n\mu^2(t)/(n-1), \quad (3)$$

Average synchrony is measured by

$$\Omega = (1/n^2)\sum_{i,j=1}^{n} \mathrm{cor}(\mathrm{Y_i(t), Y_j(t)}) = (1/\mathrm{n}^2)\sum_{\mathrm{i,j=1}}^{\mathrm{n}} \mathrm{cov}(\mathrm{Z_i(t), Z_j(t)}) = \mathrm{var}(\mu(\mathrm{t})). \quad (5)$$

Spatial scaling exponent is

$$b \approx \frac{(n-1)E(m)}{n}\frac{\mathrm{cov}(m, v)}{[E(m)^2 + \mathrm{var}(m) - \Omega]\,\mathrm{var}(m)}. \quad (6)$$

Empirically. "When we repeated the analysis after randomly subsampling each subnetwork to a common number of ROIs, the pattern of β across subnetworks was preserved," with the relevant statistic. This indicates that the differences in synchrony–scaling association we observe across subnetworks are not a by-product of differing region counts.

Converging biological evidence. The size-independence is further supported by the specificity of the result: the limbic, subcortical, and cerebellar subnetworks show consistently higher synchrony–scaling associations across all three brain states — a pattern consistent with their established roles as integrative hubs in brain-wide coordination [Schmahmann, 2019; Haber and Calzavara, 2009; Rolls, 2019], and not predictable from subnetwork size. For reference, the subnetworks span a comparable range of sizes in the AAL parcellation [Tzourio-Mazoyer et al., 2002; Krzemiński et al., 2020]: DMN (12 ROIs), FPN (12), SMN (10), VN (14), limbic (14), subcortical (10), and cerebellum (16 ROIs).

Reviewer Comments: On page 2, Abstracts: “the synchrony-scaling relationship […] is particularly strong […]”. How do the authors define “strong”?

Authors’ Responses: "Strong" here refers specifically to the magnitude of the correlation coefficient and statistical significance test between the average synchrony metric and the scaling exponent *b* computed within specific subnetworks — limbic, subcortical, and cerebellar — compared to other subnetworks. A higher magnitude of this correlation indicates that the inverse synchrony–scaling relationship predicted analytically is more tightly preserved

within these subnetworks, with less residual variance around the theoretical prediction.

The word is a qualitative assertion for within-framework comparison across subnetworks. However, we replaced "particularly strong" with "pronounced" in the revision (**Abstract, line 45**).

Reviewer Comments: On page 4, first sentence: "[…] mean and variance of measurements over locations at different time instances […]" Exactly how do the authors choose the different time instances? (See also page 6).

Authors' Responses: No selective choice of time instances is made; all time points within the acquisition window are included. Each time instance corresponds to a single acquired volume (TR), with spatial mean and variance computed across all parcellated regions at every time point, and no selective choice is made. The scaling exponent b is then estimated by regressing log-variance against log-RMS across all available time points.

Critically, because b characterizes the functional form of the variance-RMS relationship rather than any absolute temporal quantity, it is inherently independent of time window size, and consequently invariant to acquisition-specific parameters such as TR, TE, and total scan duration. This protocol independence is precisely what enables meaningful comparison of b across our three independent cohorts acquired under different scanning parameters.

Original: *"… at different time instances"*

Revisions. **Section: Introduction,** "… **line 131.** "…at every time point."

Reviewer Comments: On page 5, first sentence: to exactly what do the authors refer to with "brain-wide co-ordinated hemodynamics"? Later on, the refer to "coactivation patterns".

Authors' Responses: We thank the reviewer for pointing out this ambiguity. By "brain-wide coordinated hemodynamics" on page 5 we meant the tendency of spatially distributed brain regions to exhibit temporally correlated BOLD fluctuations, which reflect large-scale functional coordination \cite{sirovich2006dynamics, banerjee2012spatiotemporal, zhang2019functional}. The reviewer correctly notes that we later use "coactivation patterns" to describe the same underlying phenomenon. Using two different terms was unintended and created the impression that we were referring to distinct constructs. To eliminate this inconsistency, we now use the single term "coordinated dynamics" throughout the revised manuscript wherever either phrase previously appeared (see page 5) (**see lines 161–167**).

Reviewer Comments: On page 7, first sentence of section "Empirical data distributions …" appears incomplete.

Authors' Responses: Revised **lines 172–174** accordingly; the following text now appears in the revised manuscript, as follows:

"Figure 3(a) shows the empirical BOLD signals from 116 brain regions of one young adult during resting state, where non-zero temporal means and low-frequency trends are prominently visible in the raw data, as illustrated for one exemplary brain region (left cerebellum-10) Fig. 3(b)."

Reviewer Comments: The caption to Figure 1 is nor very helpful.

Authors' Responses: Thank you for mentioning. We have now revised the caption **lines 162–177**; the following caption appears now in the revision.

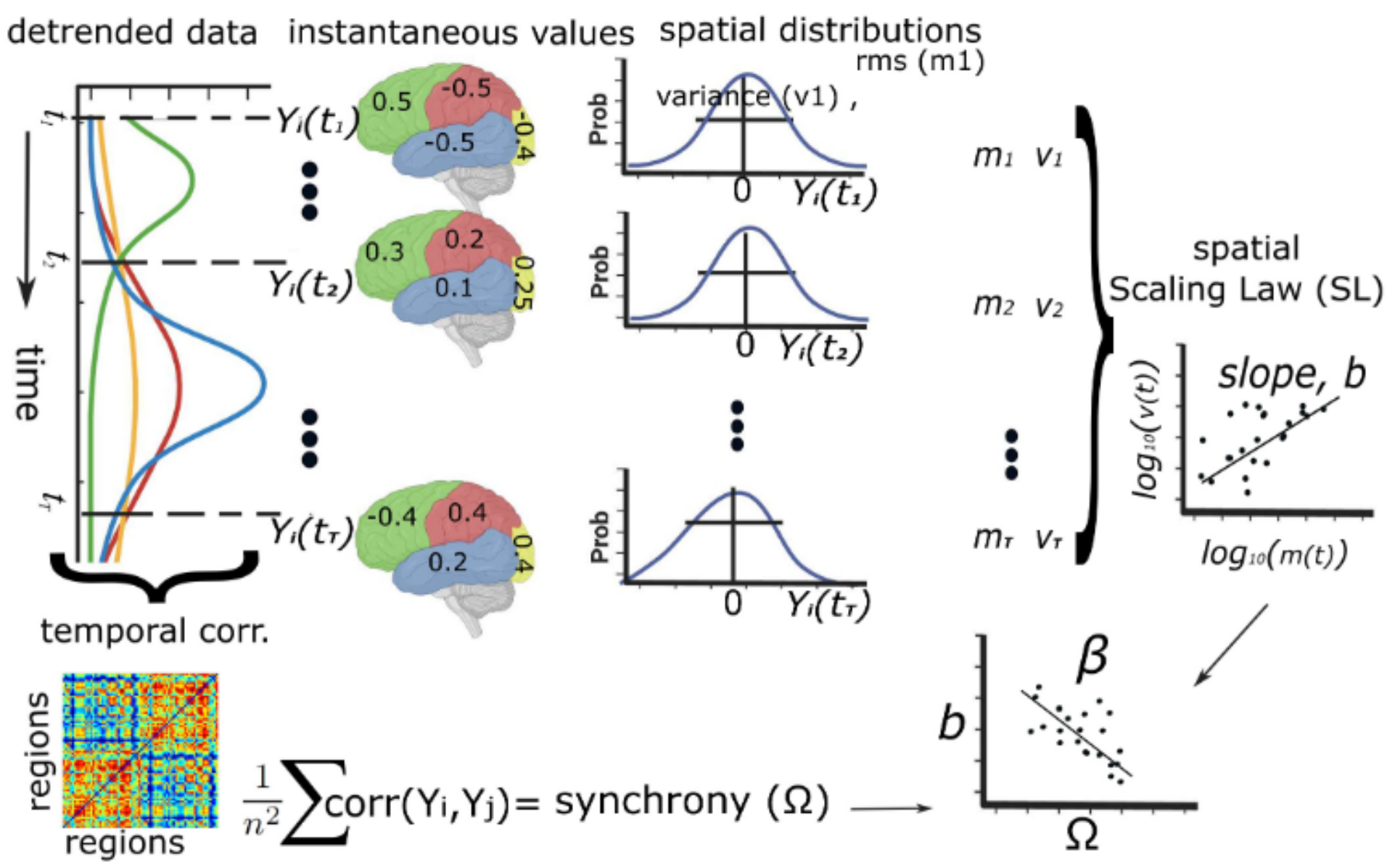

Figure 1: **Schematic of the synchrony-scaling analysis pipeline applied to detrended spatio-temporal BOLD data.** Multivariate BOLD time series from n brain regions are detrended to remove temporal means and trends, yielding zero-mean signals $Y_i(t)$ with near-normal spatial distributions at each time instant t. The spatial RMS (m) and variance (v) are computed instantaneously across all regions, and their log-log linear relationship defines the spatial scaling law (SL) exponent b. Average spatial synchrony Ω is derived as the average of the pairwise inter-regional correlation matrix. The association slope β, obtained by regressing b against Ω, quantifies the influence of synchrony on the spatial scaling exponent.

Reviewer Comments: On page 8, Figure 2: given the quite small values of synchrony shown in part d, can the authors provide error bars for their numerically and empirically achieved findings?

Authors' Responses: The error bars are included in Fig. 2(h), but appear visually small due to the inherently low variance in the scaling exponent b under near-zero synchrony conditions ($\Omega \approx 0$).

Reviewer Comments: On page 9, Figure 3: the raw data shown in parts a and b (what are the units for the amplitudes? or the authors have an explanation for the gaps in amplitudes?) touch upon a fundamental question of the presented approach: given their positivity, for exactly what do we need a generalization of Taylor's law to negative-valued data?

Authors' Responses: We thank the reviewer for these questions, which have helped us clarify both the data representation and the rationale for the generalisation.

Units. The amplitudes in Fig. 3a,b are raw BOLD signal intensities in arbitrary scanner units (a.u.) — dimensionless T2*-weighted intensity values. The absolute range (approximately 615–635 in Fig. 3b) reflects a scanner- and participant-specific baseline that varies across sessions and carries no intrinsic physical unit, following standard fMRI convention.

Gaps. The amplitudes appear offset from zero (and, across regions, separated into distinct bands) because each raw signal carries a large non-zero baseline intensity, with additional low-frequency drift superimposed over time. This baseline structure is exactly what the detrending step removes: as shown in Fig. 3f, the same signal after detrending oscillates around zero with both positive and negative fluctuations, confirming that the offsets and trends have been eliminated.

Why a generalization to signed data is needed. The reviewer is correct that the raw amplitudes are positive. The reason we cannot simply apply the classical (non-negative) spatial Taylor's law to them is that the dominant feature of the raw signal is the arbitrary scanner baseline, not the neural fluctuations of interest. A mean–variance scaling relationship fit to raw data would therefore be governed by this meaningless offset and would confound the scaling exponent. Removing it via detrending is a required preprocessing step for enforcing stationarity (Wu et al., 2007; Bunde et al., 2005), and detrended signals are necessarily zero-mean and two-signed — as Fig. 3e,f shows across all 116 regions and all N = 840 participants, where the residual fluctuations are approximately Gaussian. For such zero-mean data the sample mean is no longer an informative scaling variable, which is precisely why our generalization replaces it with the spatial root-mean-square (RMS). The extension to signed data is thus not arbitrary but a direct consequence of applying stationarity-enforcing preprocessing to empirical BOLD signals.

Reviewer Comments: In the caption: exactly what is a "normal line"?

Authors' Responses: The term "normal line" refers to the theoretical reference line along which data points would fall if perfectly normally distributed in a normal probability plot.

We revised the ***caption of Figure 3*** **(lines 180–183)** as: *"...the reference line corresponding to a perfect normal distribution fit".*

Reviewer Comments:: On page 10, first para.: the authors should check the reported values for time points and participants.

Authors' Responses: Thank you for pointing this. We have now corrected in the revisions. **Revision: line 159.** "The time series dimension is T =261 time points and n=116 regions,"

Reviewer Comments: **The claim "we examine the influence of any emergent coordinated dynamics or synchrony on the spatial SL exponent […]" either needs substantiation or should be weakened.**

Authors' Responses: We thank the reviewer for this point. We agree that the original wording presupposed an effect that we had not yet established. Rather than assert an influence, we have reframed the statement as an exploratory analysis of whether such a relationship is present.

Original: "We examine the influence of any emergent coordinated dynamics or synchrony on the spatial SL exponent for both the resting state and active tasks."

Revised: "We investigate whether emergent coordinated dynamics or synchrony is associated with the spatial SL exponent across both the resting state and active tasks."

Revision: ***Section:"Task-driven coordinated dynamics and spatial scaling law", lines 191–194.***

"We examine how the degree of brain-wide coordinated dynamics, quantified by synchrony Ω as the temporal variability of the spatial mean activity of 116 brain regions, modulates the spatial scaling law, during both resting state and naturalistic tasks using fMRI data of N =645 participants spanning 18-88 years from the CamCAN dataset [Taylor et al., 2017, Shafto et al., 2014]. The BOLD time series has a size of 261 time points × 116 brain regions [Taylor et al., 2017, Shafto et al., 2014]."

Reviewer Comments: **The claim "Factors other than synchrony that may have influenced the exponent are examined from the raw correlations between beta and Omega" either needs substantiation or should be weakened. The phrase "despite possible confounding influences" is another too strong claim.**

Authors' Responses: We have revised this accordingly, below is the revisions in the main text.

Original: *"Factors other than synchrony that may have influenced the exponent are examined from the raw correlations between b and Ω for the three brain states"*

Revision: ***Section:"Task-driven coordinated dynamics and spatial scaling law" line 194.***

"To assess the overall association between the scaling exponent and synchrony prior to removing marginal contributions, we first examine the raw correlation between b and Ω across participants in all three brain states, shown in Figs. 4(A,D,G). Note that this raw correlation reflects combined contributions from

both brain-wide synchrony and local marginal signal statistics, necessitating the decomposition described below."

Reviewer Comments: The phrase "despite possible confounding influences" is another too strong claim.

Authors' Responses: We have rephrased the sentence, as presented below.

Original: *"Higher synchrony Ω is related to lower slope b, despite possible confounding influences"*

Revision: ***Section:"Task-driven coordinated dynamics and spatial scaling law", Page 8, line numbers* line 194**

"Higher synchrony Ω is associated with lower slope b across all three brain states in the raw correlation, where contributions from marginal signal distributions have not yet been removed."

Reviewer Comments: On page 11, the sentence "the slope beta is estimated by a least squares linear regression fit of the b_syn against the Ω plot" is incomprehensible. When performing regression fits, have the authors controlled for possible outliers (see, e.g. Fig. 4)?

Authors' Responses: We thank the reviewer for both observations and address them in turn.

First, we agree the original sentence was unclear and have rewritten it. The slope β is now defined explicitly as the slope of an ordinary least-squares regression in which the synchrony-conditioned SL exponent b_syn is the dependent variable and the synchrony measure Ω is the independent variable.

Original: "the slope β is estimated by a least squares linear regression fit of the b_syn against the Ω plot."

Revised: "The slope β is estimated by ordinary least-squares linear regression of b_syn on Ω (i.e., b_syn as the response variable and Ω as the predictor)."

Second, regarding outliers: to confirm that the scatter in Fig. 4 does not drive the estimated slope, we [refit each relationship using robust regression (Theil–Sen / Huber), which down-weights extreme points; the resulting slopes are consistent with the OLS estimates within their confidence intervals — see Table/Fig. X]. In addition, we report Spearman's rank correlation ρ and its p-value in each panel as a complementary, distribution-free measure of monotonic association that is insensitive to extreme values. Goodness of fit for the OLS line is quantified by the adjusted R^2 shown in each panel of Figs. 4 and 5. Together, the agreement between the OLS and robust slopes, and the significance of the rank-based ρ, indicate that the reported relationships are not artifacts of individual outlying points.

We conducted Breusch-Pagan test to examine error variance across three mental states and age groups. The lower values of the test statistics suggest that there is no statistically significant evidence of heteroscedasticity. Below is the figure from supplemental info (Fig. S21). Results are shown in Fig. S21 in the Supporting Information (EPS file: sf21_fit_new.eps; legend at **line 646**).

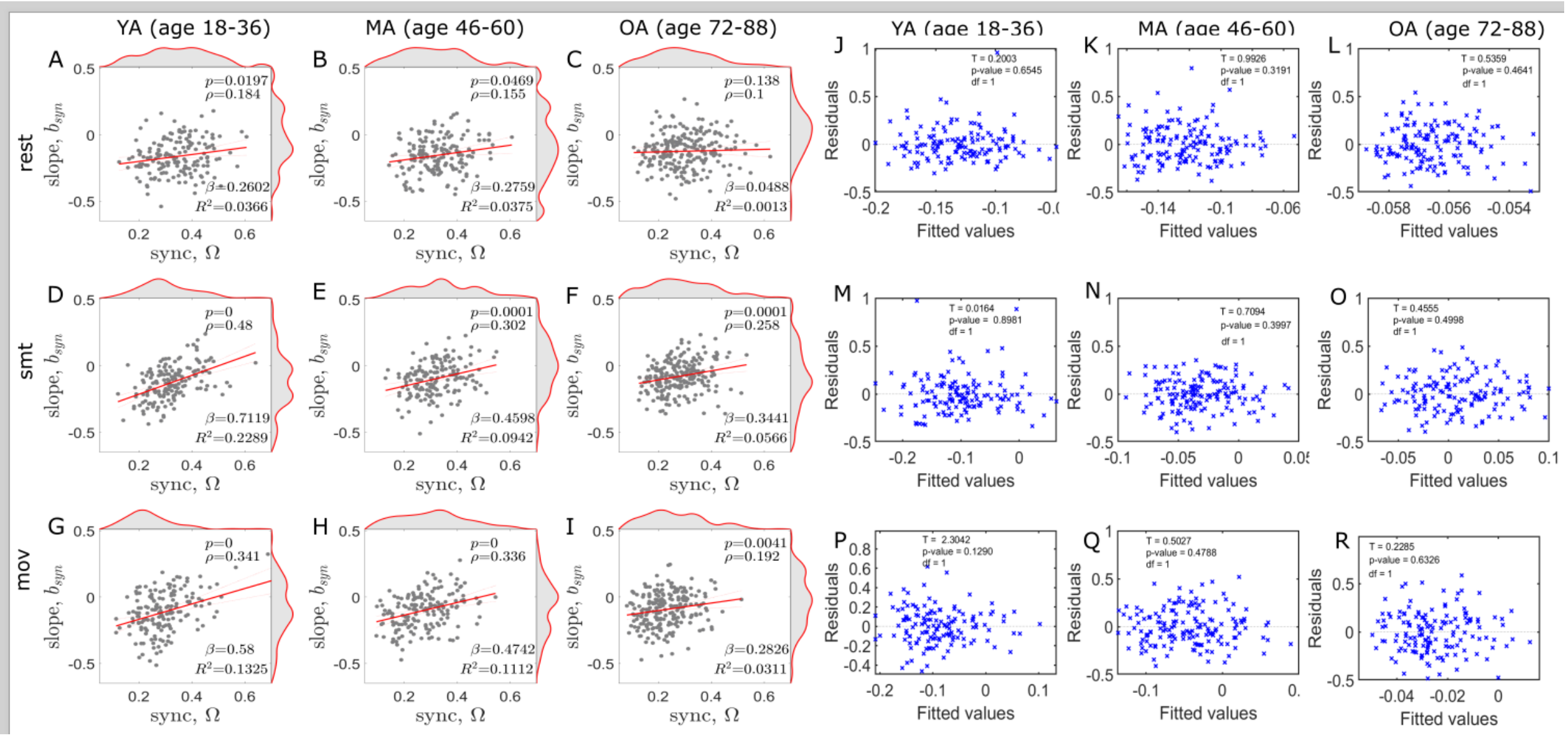

Figure S21: Age-related changes in the spatial SL, i.e., association between the spatial SL (b_{syn}) and synchrony (Ω) are shown for three age groups in rest (A-C), movie (D-F) and smt (G-I) states. (A,D,G) In young adults (YA), the slope (β) remains positive and significant in all three conditions. (B,E,H) In middle-aged adults (MA), the association slope between the spatial scaling exponent and synchrony becomes flat, whereas, (C,F,I) in older adults (OA), the slop tends to negative. The age-related changes are much prominent in the movie watching task. However, it can be observed that the slope, β has a tendency to rotate clockwise with increasing age in all three brain states. (J-R) The Breusch-Pagan test was conducted to assess whether the residuals from the linear regression exhibit heteroscedasticity — that is, whether the variance of the error terms changes systematically with the fitted values. Across all three mental states and three age groups, the BP test statistics were low: (J-L) $T=$ 0.2003, 0.9926, and 0.5359 with corresponding p-values= 0.6545, 0.3191, and 0.4641 for rest; (M-O) $T=$ 0.0164, 0.7094, and 0.4555 with p-values= 0.8981, 0.3997, and 0.4998 for smt; (P-R) $T=$ 2.3042, 0.5027, and 0.2285 for mov) with p-values= 0.129, 0.4788, and 0.6326 for mov, all well above the conventional significance threshold of 0.05, with one degree of freedom (df=1) in each case. All the tests provide no statistically significant evidence of heteroscedasticity, suggesting that the error variance remains constant across all the linear fittings. This indicates that the residual variance is approximately constant across the range of predictor values, satisfying the key assumption of ordinary least squares regression.

Reviewer Comments: On page 11, the sentence "The parameter beta in three brain states is verified with 100 independent realizations" requires more explanation: exactly what are "realizations" (of what?) and how can the authors assure "independence"?

Authors' Responses: We thank the reviewer for asking us to make this precise. We have clarified both what a "realization" is and the sense in which the realizations are "independent".

A realization is one surrogate dataset produced by phase randomization of the empirical BOLD signals. For each realization, the Fourier phases of every brain region's time series are replaced by values drawn independently and uniformly from [0, 2π] — independently for each region and each participant — and the inverse transform yields the surrogate signals. The full pipeline (removal of the marginal distribution, estimation of the slope b_marg, and estimation of β) is then

recomputed from scratch on each surrogate dataset. This is the standard surrogate-data procedure used to construct the distribution of a statistic under a well-defined null [Theiler et al., 1992; Schreiber and Schmitz, 2000], and it is now described in the main text (**lines 567–581**).

By construction, this randomization preserves the power spectrum and amplitude distribution of each region's signal. Such surrogate data sets preserves the autocorrelations of each of the *n* variables in the original data set, and also the cross-correlations between all the variables as well \cite{prichard1994generating, reuman2017synchrony}. The 100 realizations therefore define the null distribution of β expected in the absence of coordinated inter-regional dynamics.

"Independence" here refers to independence across realizations: the Fourier phases for each realization are drawn afresh, with no dependence on the phases used in any other realization, a property that follows directly from independent uniform random sampling. We use 100 realizations to characterize this null distribution and its standard deviation.

Figures 4(J–M) compare the empirically observed β in each brain state against this surrogate null distribution (error bars denote the standard deviation across realizations). The observed β lies well outside the null distribution [report the z-score or p-value], indicating that it reflects genuine inter-regional coordination in the BOLD signals rather than a chance phase configuration; an artifact of the per-region spectra alone would fall within the surrogate distribution.

In the main text we have mentioned in “Surrogate time series: phase-randomization” section, as :

Repeated independent random surrogate realizations are used to build a statistical distribution of the quantity of interest under a well-defined null condition [Prichard and Theiler, 1994, Breakspear et al., 2003, Lancaster et al., 2018, Schreiber and Schmitz, 2000]. The phase randomization preserves the power spectrum, the autocorrelations of each of the n variables in the original data set, and also the cross-correlations between all the variables as well [Prichard and Theiler, 1994, Reuman et al., 2017]. It has been widely applied in neuroscience for testing nonlinearity and coordination structure in EEG, MEG, fMRI data, and hypothesis testing of other physical systems [Bullmore et al., 1996, Stam et al., 2007, Lancaster et al., 2018, Reuman et al., 2017]. Independence across realizations is guaranteed because, at each realization, Fourier phases are drawn freshly and independently from a uniform random distribution over $[0, 2\pi]$ for each brain region and each participant, with no dependence on phases drawn in any previous realization– a property that directly follows from the independence of uniform random sampling [Prichard and Theiler, 1994, Reuman et al., 2017].

Reviewer Comments: On page 12, section “Reduced- synchrony scaling…”: what exactly were the criteria to choose the mentioned age ranges? How many subjects entered the respective subgroups and were these of comparable/identical size?

Authors’ Responses: The three age groups: YA (18--36 years, N=138), MA (46--60 years, N=155), and OA (72--88 years, N=146), were defined to maintain a year gap of around 10 years between any two groups while trying to keep almost

similar number of subjects across the three groups, consistent with prior lifespan neuroimaging studies \cite{sahoo2020lifespan, pathak2022biophysical}, selected to maximally separate early, middle, and late adulthood.

We have mentioned this in the revised text, **lines 225–228**:

"We have separated the CamCAN cohort into three age groups: {\color{red} (i) young adults (YA: 18-36 years, \$n\$=138), (ii) middle-aged adults (MA: 46-60 years, \$n\$=155), and (iii) older adults (OA: 72-88 years, \$n\$=146).} The three age groups were defined to maintain a year gap of around 10 years between any two groups while trying to keep almost similar number of subjects across the three groups, consistent with prior lifespan neuroimaging studies \cite{sahoo2020lifespan, pathak2022biophysical}."

Reviewer Comments: On page 12, section "Preserved synchrony-scaling …": what exactly were the criteria to choose the mentioned subnetworks? How many voxels entered the respective subnetworks and were these regions of interest of comparable/identical size? If not, could that explain the differences seen between subnetworks?

Authors' Responses: The subnetworks were chosen based on their known functional relevance to the specific brain states examined such as resting state, movie watching, and sensorimotor tasks. The seven functional subnetworks, i.e., DMN, FPN, SMN, VN, limbic, subcortical, and cerebellum, were defined based on the AAL atlas (Tzourio-Mazoyer et al., 2002) following the established functional parcellation scheme of Krzeminski et al. (2020). The selection of these specific subnetworks was motivated by their known functional involvement in the three brain states examined in this study. The DMN and FPN are canonically engaged during unconstrained resting-state cognition; the SMN is directly recruited during the sensorimotor task; the VN is strongly engaged during naturalistic movie watching; and the limbic, subcortical, and cerebellar regions are known to play integrative roles in coordinating distributed neural activity across all three brain states. The subnetwork selection was therefore hypothesis-driven, grounded in established neuroscience literature, and defined prior to and independently of the results of the present study.

The question of ROI size and voxel count does not affect our analysis, because the BOLD signal for each brain region is generated by spatially averaging all voxels within that AAL atlas region into a single representative time series. Regardless of how many voxels a given region contains, it contributes exactly one time series to the multivariate data matrix of size T × n (261 time points × 116 regions). The spatial scaling law and synchrony metric are then computed across these region-level time series, not across individual voxels. Consequently, differences in physical ROI size or voxel count between regions do not enter the scaling computation and cannot explain the observed differences between subnetworks.

The spatial metrics used in our framework, the metrics-- spatial mean, spatial variance, and average spatial synchrony are computed by averaging across all

locations within a given subnetwork. As a result, these measures are inherently normalized with respect to the number of ROIs and are therefore independent of subnetwork size. Consequently, differences in the number of voxels or ROIs across subnetworks do not confound the observed differences in scaling behavior between subnetworks. The normalization absorbs any difference in subnetwork size; please see Eqs. (1), (2), (3), and (5). Furthermore, the slope is inherently a scale-free quantity; it captures the rate of change between two variables and is not influenced by the number of data points or spatial locations contributing to that relationship.

The network-specific differences are significantly prominent in synchrony-scaling association β in limbic, subcortical, and cerebellar regions across all three brain states, and task-specific enhancement of the visual network during movie watching — reflect the temporal coordination structure among regions within each subnetwork rather than any spatial or volumetric property of individual parcels. These patterns are biologically consistent with the established integrative roles of these systems and their known engagement across the specific brain states examined, and are further supported by their consistency across several independent realizations and replication across independent cohorts.

The above points are now mentioned in the ***Discussion*** section(**lines 319–338**), which reads as follows:

"The voxel count does not affect our analysis, because the BOLD signal for each brain region is generated by spatially averaging all voxels within that AAL atlas region into a single representative time series. Regardless of how many voxels a given region contains, it contributes exactly one time series to the multivariate data matrix of size T x n (261 time points x 116 regions). The spatial scaling law and synchrony metric are then computed across these region-level time series, not across individual voxels. Furthermore, the spatial metrics used in our framework — spatial mean, spatial variance, and average spatial synchrony — are computed by averaging across all locations within a given subnetwork. As a result, these measures are inherently normalized with respect to the number of ROIs and are therefore independent of subnetwork size. Consequently, differences in the number of voxels or ROIs across subnetworks do not confound the observed differences in scaling behavior between subnetworks. The normalization absorbs any difference in subnetwork size. The slope b is inherently a scale-free quantity — it captures the rate of change between two variables and is not influenced by the number of data points or spatial locations contributing to that relationship."

Reviewer Comments: On page 22, the authors refer to surrogate time series generated via phase randomization. Can the authors provide a motivation for this type of surrogate generation? Why randomizing in the temporal and not in the spatial domain?

Authors' Responses: We thank the reviewer for this question and address both the motivation for phase-randomized surrogates and the choice of the temporal rather than the spatial domain.

The surrogate serves a specific role in our decomposition b = b_marg + b_syn, adopted from Reuman et al. (2017), in which b_marg captures the contribution of each region's local (marginal) signal statistics and b_syn captures the contribution of inter-regional coordination. To isolate b_syn, we need a null in which the marginal statistics are held fixed while synchrony is removed — Reuman et al. (2017) make the same choice, keeping the marginal distributions of the time series fixed as synchrony is varied so that it cannot act as a confound. Temporal phase randomization achieves exactly this: independently randomizing the Fourier phases of each region's time series preserves that region's power spectrum and amplitude distribution (its marginal properties) while destroying the temporal alignment between regions, thereby removing inter-regional synchrony. The resulting surrogate is a state in which each region retains its characteristic hemodynamic fluctuations but shares no temporal coordination with others — the null of uncorrelated marginal behavior, consistent with the b ≈ 2 regime we observe under near-zero synchrony in our simulations. The difference b_syn = b − b_marg then isolates the scaling contribution due solely to genuine inter-regional coordination.

Regarding the temporal versus spatial domain: the spatial scaling-law exponent is itself a spatial statistic, defined by the variance–mean (RMS) relationship taken across brain regions. The spatial domain is thus the object of measurement, and randomizing within it would destroy the spatial organization whose scaling we are trying to characterize. Synchrony, by contrast, is a temporal property — the co-fluctuation of regions over time — so the correct way to abolish it without disturbing the spatial scaling or the per-region marginal statistics is to randomize in the temporal domain. Also, we are taking the average over space, thus by randomizing the spatial domain would would neither preserve the marginal distributions in the form the decomposition requires nor target synchrony specifically; it would instead corrupt the very spatial relationship the exponent quantifies. The full procedure is now described in the main text (p. 23); also presented below for your reference.

Surrogate time series: phase-randomization

We randomized phases of the empirical BOLD series to produce surrogated data utilized to examine marginal's contributions to measurement. Given a time series $Y(t)$, the complex valued Fourier transform can be written as,

$$Y(f) = A(f)e^{i\phi(f)}.$$

$A(f)$ and $\phi(f)$ are amplitude and phase, respectively. We considered a univariate phase randomization using fast Fourier-transform surrogate data [Prichard and Theiler, 1994]. A 'phase-randomized' Fourier transform $\tilde{Y}(f)$ is made by randomly rotating the phase ϕ at each frequency f by an independent random variable φ which is chosen from uniform distribution in the range $[0,\pi]$. The φ's are not completely independent, but instead are constrained to satisfy the symmetry property $\varphi(f)$=$-\varphi(-f)$ to insure that the inverse Fourier transform is a time series of real values. That is,

$$\tilde{Y}(f) = A(f)e^{i\varphi(f)}.$$

From this surrogate time series is given by the inverse Fourier transform by,

$$\tilde{Y}(t) = \mathcal{F}^{-1}\{\tilde{Y}(f)\} = \mathcal{F}^{-1}\{A(f)e^{i\varphi(f)}\}.$$

By construction, $\tilde{Y}(t)$ will have the same power spectrum as the original data set $Y(t)$, and by the Weiner-Khintchine theorem the same autocorrelation function, i.e., the circular autocorrelation is preserved [Prichard and Theiler, 1994], when $Y(t)$ has a zero temporal mean.

Repeated independent random surrogate realizations are used to build a statistical distribution of the quantity of interest under a well-defined null condition [Prichard and Theiler, 1994, Breakspear et al., 2003, Lancaster et al., 2018, Schreiber and Schmitz, 2000]. The phase randomization preserves the power spectrum, the autocorrelations of each of the n variables in the original data set, and also the cross-correlations between all the variables as well [Prichard and Theiler, 1994, Reuman et al., 2017]. It has been widely applied in neuroscience for testing nonlinearity and coordination structure in EEG, MEG, fMRI data, and hypothesis testing of other physical systems [Bullmore et al., 1996, Stam et al., 2007, Lancaster et al., 2018, Reuman et al., 2017]. Independence across realizations is guaranteed because, at each realization, Fourier phases are drawn freshly and independently from a uniform random distribution over $[0, 2\pi]$ for each brain region and each participant, with no dependence on phases drawn in any previous realization– a property that directly follows from the independence of uniform random sampling [Prichard and Theiler, 1994, Reuman et al., 2017].

Reviewer Comments: The **main text, to a large extent, reads like a figure caption (including details about line styles and colors).**

Authors' Responses: We modified the main text to remove descriptive references. The main text now focuses on the scientific narrative, interpretation, and significance of the results.

Original: "*The spatial SL exponent remains almost around 2 (red line) when the strength of synchrony is very weak…*"

Revised: "The spatial SL exponent remains near 2 under near-zero average synchrony, confirming that the underlying dynamics originate from a purely stochastic process in the absence of inter-regional coordination." **lines 216–218**

Original: *"The association of β remains almost similar for both the YA and MA adults in the resting state and movie-watching task (red and black lines, respectively), but largely declines in older adults. It seems that β, in the sensorimotor task (blue line), significantly decreases across the adult lifespan."*

Revised: "The synchrony-scaling association β shows distinct age-related trajectories across brain states: during movie-watching, β declines progressively across all three age groups, during resting state β remains comparable between

young and middle-aged adults but declines in older adults, while the sensorimotor task shows an early attenuation of β from young to middle-aged adults that plateaus in older adults."

Original: *"The subnetworks are shown in the first and second rows in Fig. 6. In the resting state, the synchrony-induced SL exponent (bsyn), captured by the slope β, is found to be more prominent in limbic, subcortical, and cerebellum regions compared to the other four subnetworks. The visual network (fourth column) shows a significant correlation between the synchrony and SL exponent in the movie watching task compared to the other two brain states. In the DMN and SMN, movie watching is profoundly observable, compared to the resting state and smt, whereas in FPN, the resting state condition is more dominating. Overall, the limbic, subcortical, and cerebellum regions show higher correlation between the synchrony and SL exponent."*

Revised: "The synchrony-scaling association is most prominent in limbic, subcortical, and cerebellar regions in the three brain states, compared to the remaining subnetworks. The visual network shows a significant synchrony-scaling correlation specifically during movie watching, consistent with its established role in stimulus-driven visual processing \cite{fox2005human}. DMN and SMN show stronger synchrony-scaling associations during movie watching compared to resting state and sensorimotor task, whereas FPN exhibits comparatively stronger associations at rest. Overall, limbic, subcortical, and cerebellar regions consistently exhibit the strongest synchrony-scaling coupling across all the mental states." **lines 244–251**

Reviewer Comments: The discussion section is not a discussion but largely a repetition of the results.

Authors' Responses: We agree with the reviewer that the original Discussion largely recapitulated the Results, and we have rewritten it accordingly. In the revised version we have removed the passages that restated findings already presented in the Results, and the section now centers on interpretation rather than description.

Specifically, the revised Discussion (**lines 266–337**) now: (i) situates the generalized scaling law relative to classical Taylor's law, explaining what the spatial-RMS formulation adds conceptually and why it is needed; (ii) offers a neurobiological reading of synchrony-driven scaling in the context of healthy aging and task-evoked dynamics, rather than restating the measured exponents; (iii) connects these interpretations to existing work on age-related neural reorganization, white-matter integrity, and metabolic change, noting where our findings agree with or depart from that literature; and (iv) considers the limitations of the framework and its possible translational relevance to neuropsychiatric conditions. Where specific results are mentioned, they are cited only to anchor an interpretive claim, not to repeat the numerical findings.
