# Supplementary material for: A generalized scaling law reveals synchrony-driven reorganization of brain dynamics across human lifespan
Source: PLoS Comput Biol. 2026 Oct 5;22(10):e1014821. doi: 10.1371/journal.pcbi.1014821 (PMC13652345; doi:10.1371/journal.pcbi.1014821)
Supplement: Supplementary file 3 [file pcbi.1014821.s003.pdf]

Response to Reviewers Comments –**Manuscript ID: PCOMPBIOL-D-26-00299R1**

“A generalized scaling law reveals synchrony-driven reorganization of brain dynamics across the human lifespan”

We thank the editor and reviewers for their careful re-evaluation of our manuscript and for the constructive feedback, valuable suggestions that had further improved the clarity and rigor of the paper. Below, we provide a point-by-point response to each comment. Corresponding changes will be highlighted in red in the revised manuscript.

Note on Supplemental Material

The full Supporting Information document has now been reviewed as per Reviewer #2's suggestions and comments. All Supplemental Material items below have been located precisely and are addressed with concrete revisions. We also note that the “??” undefined cross-references flagged by the reviewer were confined to the abbreviated “Supporting Information Legends” section reproduced inside the main manuscript file (a separate, shorter legend summary); the full Supporting Information document itself compiles correctly, with all Algorithm/Equation/Figure references properly resolved. **Figure 2** is edited and necessary corrections are made inside the figure and caption as well. Below are the point-by-point responses.

General Comment

Reviewer Comment: *The authors have – to a large extent – improved their manuscript. It is more accessible and many of the previously too strong claims were either weakened or supported by additional information/findings. Nevertheless, there are still some old and new issues that require the authors' close attention: Contrary to what is claimed in the response letter, the authors continue to mix the terms “synchronized” and “synchronous” and the text still contains descriptive references (see, e.g. captions to Figs. 2 and 3 and text passages related to these figures).*

Authors’ Response: We thank the reviewer for flagging that this issue persists. We have now performed a systematic, manuscript-wide search-and-replace pass (main text, all figure captions, and Supporting Information) to standardize terminology: we retain *“synchronized”* only as a past-participle/adjectival form describing a state (e.g., “synchronized activity”) and *“synchronous”* only when describing simultaneity of events, and we have removed inconsistent interchangeable use of the two terms. In particular, the captions of Figs. 2 and 3 (highlighted in the annotated manuscript) have been revised to (i) use consistent terminology and (ii) move from purely descriptive, panel-by-panel narration toward statements that also convey the interpretive significance of each panel. As a concrete example of the confusion the reviewer flagged, we found the sentence in Supplemental Material Sec. 10.0.1: “*r(t) = 1 representing complete synchronous, r(t) = 0 for desynchrony…*” – corrected to “r(t) = 1 representing complete synchrony, r(t) = 0 for desynchrony…”. We have applied the same fix throughout both the main text and Supplemental Material.

Abstract

Reviewer Comment: *Abstract, lines 30–34 and 40–43, these sentences are largely incomprehensible.*

Authors’ Response: We agree these two sentences were difficult to parse and have rewritten both for clarity. Both revised sentences have been highlighted in red (Abstract).

Original (lines 30–34): “To circumvent this limitation, we first extend the application of Taylor’s spatial scaling law across various discipline by replacing the sample mean with root-mean-square (RMS) of any measurements, and then apply on multivariate time series in neural signals after detrending, in search of a scaling law therein.”

Revised (lines 30-33, page 1): “To circumvent this limitation, we extend Taylor’s spatial scaling law by replacing the sample mean with the root-mean-square (RMS) of the measurements, and apply this generalized law to detrended multivariate neural time series.”

Original (lines 40–43): “Finally, we apply this approach to human fMRI data from three large lifespan cohorts (N = 840, ages 18–88) reveals distinct age-related trajectories of the scaling exponent, with healthy aging characterized by a substantial synchrony-induced reduction in the exponent.”

Revised (lines 39-41, page 1): “Finally, applying this approach to human fMRI data from three large cohorts (N = 840, ages 18–88) reveals distinct age-related trajectories of the scaling exponent, with healthy aging characterized by a substantial synchrony-induced reduction in this exponent.”

Reviewer Comment: *Abstract, lines 34–35: this is quite a strong claim; detrending does not remove any kind of non-stationarity.*

Authors’ Response: Agreed and edited the text accordingly.

Original (lines 34–35): “The detrending operation has been performed as a necessary step to remove non-stationarity that usually appears in measurement data.”

Revised (lines 33-34, page 1): “The detrending operation has been performed as a necessary step to remove linear trends and the temporal mean of the data.”

Figure 3

Reviewer Comment: *Caption to Fig. 3: if the raw data (amplitude, time) shown are dimensionless, the authors should say so in the caption or add “[a.u.]” to axes labeling.*

Authors’ Response: We thank the reviewer for this observation. The BOLD signal amplitude values are indeed in arbitrary units. We have added “[a.u.]” to the relevant axis labels in Fig. 3 and stated explicitly in the caption : “Amplitude is in arbitrary units [a.u.], while time is given in units of TR (repetition time in seconds).” Please refer to Fig. 3, page 7, below is the edited figure.

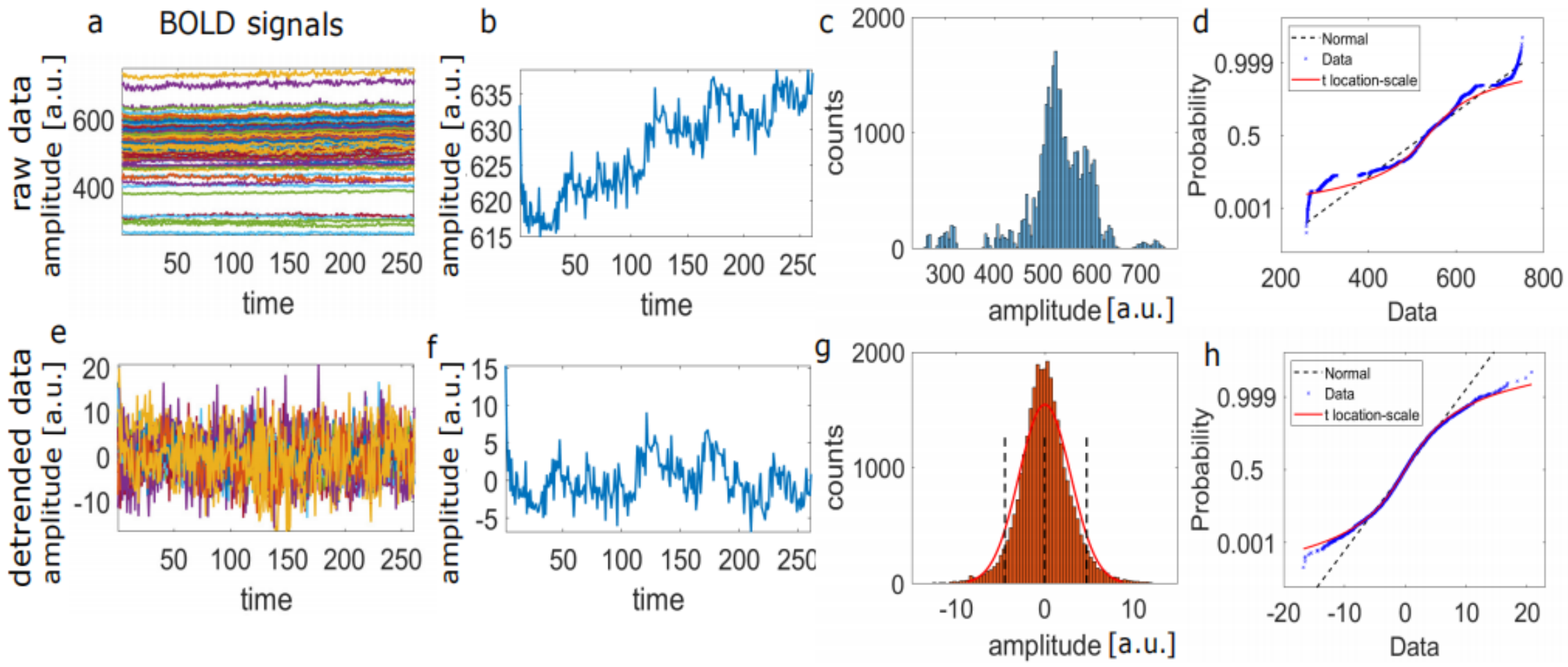

Figure 3: **Empirical data distributions: Before and after detrending operation.** (a) Empirical BOLD series in the resting state of one participant. Data size is 261 × 116; brain regions = 116 (AAL atlas), time points = 261. Amplitude is in arbitrary units [a.u.], while time is given in units of TR (repetition time in seconds). (b) BOLD signal of left cerebellum-10. (c) Histogram plot of the raw data. (d) The normal probability plot shows that neither the normal line (black dashed line, the reference line corresponding to a perfect normal distribution fit) nor the location-scale curve (red) fits very well. (e) After detrending, the BOLD series of all the brain regions is shown to spread across zero. (f) Suppressed trend of the BOLD signal of left cerebellum-10, compare (b). (g) Data distribution with normal fit of the blue curve in (f). The vertical black dashed lines indicate 95% CI. (h) Normal probability plot of the detrended data shows that it fits well to the normal distribution for the data range within 95% CI.

Results – Task-driven coordinated dynamics and spatial scaling law

Reviewer Comment: *Line 281: exactly what is a "scatterplot fit"?*

Authors' Response: We have removed the ambiguous term "scatterplot fit."

Original (Line 281): "…the scatterplot fit shows significantly positive correlations…"

Revised (lines 263-264, page 8): "…the ordinary least-squares regression of b_syn on Ω shows a significant positive slope…"

Reviewer Comment: *Lines 284–285: Contrary to what is claimed in the response letter, the authors have not changed the incomprehensible sentence "the slope beta is estimated by a least squares linear regression fit of the b_syn against the Ω plot".*

Authors' Response: We apologize – on review, this sentence was indeed left unchanged from the original submission in the main 'Results' text, even though it was corrected elsewhere. We have now corrected it in situ.

Revised (lines 266-268, page 8): "The slope β is estimated by ordinary least-squares linear regression of b_syn on Ω (i.e., with b_syn as the dependent variable and Ω as the independent variable)."

Results – Synchrony-scaling association in functional subnetworks

Reviewer Comment: *Lines 322–341: the newly added text contains many abbreviations whose explanations are only given in the following paragraph. Line 335: do you really mean "differentially"?*

Authors' Response: We have moved the full-form definitions of DMN (default mode network), FPN (frontoparietal network), SMN (sensorimotor network), and VN (visual network) to their first point of use in this

paragraph, rather than the paragraph that follows, so each abbreviation is defined before or at its first occurrence. (Please refer to lines 310-314, page 10)

Regarding "differentially" (line ~335): We intend this in the sense that age-related neurodegeneration does not affect all subnetworks equally – subcortical and limbic structures show earlier white-matter and metabolic decline than other subnetworks. We have edited this for clarity:

Revised (line 318-320, page 10): "Critically, age-related neurodegeneration affects these networks unevenly, with subcortical and limbic structures among the earliest to exhibit white matter degradation and metabolic decline [Weistuch et al., 2021, Hinault et al., 2020]."

Reviewer Comment: *Line 346: The abbreviation "ROI" is not defined.*

Authors' Response: Corrected. We have added the full form at first use: "region of interest (ROI)", and use "ROI" consistently thereafter (line 329, page 11).

Typographical Corrections

Reviewer Comment: *Line 442: Nuerobiological -> Neurobiological.*

Authors' Response: Corrected. Please refer to line 425, page 14.

Reviewer Comment: *Line 465: snycrony -> synchrony (appears as "synchorny" in the manuscript).*

Authors' Response: Corrected; we have also searched the full manuscript and Supporting Information for the same transposition error ("synchorny") and corrected all remaining instances.

Reviewer Comment: *Lines 776–778: Replace "Weiner" with "Wiener".*

Authors' Response: Corrected "Weiner-Khintchine theorem" -> "Wiener-Khintchine theorem", (line 760, page 24 in main text, and also in Supporting Information, page 28).

Methods

Reviewer Comment: *Line 625: to what does the suffix 1 (v_1, m_1) refer to here?*

Authors' Response: The subscript 1 denotes the value at the first time instant ($t = t_1$) in the schematic illustration (Fig. 1), i.e., v_1 and m_1 are the spatial variance and spatial RMS computed across all brain regions at that single instant ($t=t_1$), before the full time series of (m,v) pairs is assembled.

We have edited the main text and Fig.1 caption for greater clarity.

Revised (line 607-611, page 18): "The spatial variance $v(t)$ and spatial root-mean-square (RMS) $m(t)$ values are derived from spatial distributions over the entire space at each time point, e.g., m_1 and v_1 (where the subscript denotes the time index) are the spatial RMS and variance, respectively, derived at time point $t = t_1$, as illustrated in Fig. 1."

Fig. 1 caption (page 5): "The spatial RMS (m) and variance (v) are derived from spatial distributions over the entire space at each time point, e.g., m_1 and v_1 (where the subscript denotes the time index) are the spatial RMS and variance, respectively, derived at time point $t = t_1$. The log-log linear relationship between the spatial RMS and variance defines the spatial scaling law (SL) exponent b."

Reviewer Comment: *Line 627: pls use math style for m and v.*

Authors' Response: Corrected – m and v (and related quantities b, Ω, β) are now consistently set in math mode throughout the main text, matching their appearance in the equations; (line 612, page 18).

Reviewer Comment: *Line 652: "when the all regions …" should probably read "when all the regions …".*

Authors' Response: Corrected; (line 637, page 19)

Reviewer Comment: *Line 710: exactly what is a linear regression through a plot?*

Authors' Response: We have rephrased this naming explicitly which variables are regressed.

Original: "…the slope and intercept of an ordinary linear regression through such a plot…"

Revised (line): "…the slope and intercept obtained by regressing the y-axis quantity on the x-axis quantity of this scatter plot…".

Reviewer Comment: *Lines 722–723: can the authors provide a mathematical argument for why they include self-correlation? Doesn't this unnecessarily inflate the value of omega?*

Authors' Response: The average synchrony Ω is not fitted or chosen ad hoc from the summation form in Eq. 5 — it is derived independently as the variance of the spatial mean signal over time, $\Omega = \mathrm{var}(\mu(t))$, where $\mu(t) = (1/n)\Sigma_i Y_i(t)$ (Eqs. 10–16 in the Methodology), a standard measure used in other empirical studies Reuman et al. (2017). This derivation never requires summing a pairwise correlation matrix at all: it is a direct, standard variance computation from the spatial mean, and so the notion of "excluding self-correlation" does not apply to it -- there is no diagonal to include or exclude in a variance of a scalar time series.

The summation form $\Omega = (1/n^2)\Sigma_{ij}\, \mathrm{cor}(Y_i, Y_j)$ (Eq. 5) is an equivalent algebraic expansion of this same quantity (shown via $Z_i = Y_i/\sqrt{\mathrm{var}(Y_i)}$ in Eqs. 10–16), not an independent definition, thus the two must agree exactly, and any "self-correlation" appearing in the expanded form is intrinsic to the identity itself rather than an added or optional term. This is precisely why $\Omega = \mathrm{var}(\mu(t))$ is the standard synchrony measure used in Reuman et al. (2017) and related work on spatial synchrony (e.g., population ecology), which we followed here (manuscript lines 707–711). That said, even taken purely within the summation form, the diagonal terms have no effect on any reported result: each of the n diagonal entries equals $\mathrm{cor}(Y_i, Y_i) = 1$ by definition, contributing a fixed additive offset of $n/n^2 = 1/n = 0.0086$ ($n = 116$ ROIs) to Ω — identical across all participants, tasks, and age groups. Since correlation, covariance, and regression slope are invariant to a uniform additive shift in a variable, $\mathrm{cor}(b_{syn}, \Omega) = \mathrm{cor}(b_{syn}, \Omega - 1/n)$, and the reported β and R^2 between b_{syn}, and Ω are identical to those computed after removing the diagonal terms. The diagonal terms therefore do not inflate or confound any association reported in the paper, they only shift the absolute scale of Ω, not its relationship to b_{syn}.

Reviewer Comment: *Line 733: you might want to use |.| instead of abs(.).*

Authors' Response: Corrected – all instances of "abs(·)" have been replaced with standard absolute-value bars "|·|", line 718, page 21 in main text, and SI page 6.

Reviewer Comment: *Lines 776–778: Although this statement is (theoretically, t -> infinity) correct, the authors should nevertheless acknowledge in the text that large deviations are to be expected given the rather limited observation time (t=261).*

Authors' Response: We agree and have added an explicit caveat immediately following this statement:

Revised (line , page): "We note that this equivalence is an asymptotic ($t \to \infty$) result; with our finite observation window ($t = 261$ time points), the empirical autocorrelation and power spectrum of the surrogate data are expected to deviate somewhat from their theoretical asymptotic values, and we account for this by generating the null distribution empirically from repeated surrogate realizations rather than relying on the asymptotic theorem directly."

Reviewer Comment: *Line 289: the authors probably mean [0,2π), if not pls provide an explanation for your choice.*

Authors' Response: The reviewer is correct. The random phase is drawn from a uniform distribution on the half-open interval [0, 2π), since 0 and 2π represent the same phase angle and including both endpoints would double-sample that angle with vanishing but non-zero probability under a continuous distribution. We have corrected the notation from "[0, 2π]" to "[0, 2π)" throughout the Methods and Supporting Information.

References

Reviewer Comment: *Throughout the reference list, the authors use lowercase letters for proper nouns (such as Taylor or Fermi), some physical units (such as T), some journals as well as for standardized abbreviations (such as EEG, MRI, fMRI, etc).*

Authors' Response: Corrected; we have used the reference style suggested by the PLOS Com. Bio. journal.

Reviewer Comment: *This needs to be corrected. Refs [Brown, 2012], [Sastry, 2022], [Shinn, 2023], [Sivagnanam, 2013], and [Yeo, 2011] are incomplete.*

Authors' Response: Corrected; Brown et al. 2012 (Frontiers in Neuroinformatics), Sastry et al. 2022 (Cerebral Cortex), Shinn et al. 2023 (Nature Neuroscience), Sivagnanam et al. 2013, and Thomas Yeo et al. 2011 (Journal of Neurophysiology), Vincent et al., 2008 (Journal of Neurophysiology), so the reference list can be finalized.

Supporting Information Legends

Reviewer Comment: *The section "Supporting Information Legends" contains many "??". Undefined references to figures and equations?*

Authors' Response: We thank the reviewer for catching this. These are unresolved LaTeX cross-references (\ref/\label mismatches) that were not compiled correctly before submission. We have fixed all broken references in this section (to the relevant algorithm, equation, and figure numbers) and have re-compiled the document to confirm no "??" placeholders remain anywhere in the manuscript or Supporting Information.

Supplemental Material

Reviewer Comment: *The authors should carefully check this document for typos, completeness of sentence, and jargon, and should reconsider their use of the terms synchronization resp. synchronized wherever necessary.*

Authors' Response: We have proofread the full Supplemental Material document and corrected all instances found, including recurring typos:

"sptio-temporal" → "spatio-temporal" (Figs. S2–S7 and surrounding text), (SI pages 13, 17)

"shownn" → "shown" (Figs. S2–S7 text),

"histgram" → "histogram" (Sec. 4.2, (SI page 20)

"balck" → "black" (SI page 16)

We also corrected a copy-paste content error repeated in **Secs. 4.1.2 and 4.1.3** (SI document, pages 13, 17), where the text states "the spatio-temporal time series no longer follow Poisson distribution" after detrending – this is incorrect in these two subsections, since the pre-detrending data there were drawn from the negative Binomial and Uniform distributions, respectively, not Poisson; the sentence has been corrected to name the correct source distribution in each subsection.

Finally, we corrected "complete synchronous" → "complete synchrony" in **Sec. 10.1** (SI, page 41) (previously Sec. 10.0.1). See also our response to the General Comment above. We reviewed all remaining uses of

"synchrony/ synchronization/ synchronized/ synchronous" in this document for consistency with the terminology convention now applied throughout the main text.

Reviewer Comment: *Abstract: The sentence "Section 4 numerically simulated results…" is incomprehensible.*

Authors' Response: Corrected – the sentence was missing a main verb.

Revised: "Section 4 presents numerically simulated results for various case studies of distributions $Y = (Y_1, \cdots, Y_n)$, constructed by transforming multivariate random variables with identically distributed marginals Y_i, for both independent and not-necessarily-independent cases." (SI page 1)

Reviewer Comment: *Text beneath caption to Fig. 1: to what does the suffix 1 (v_1, m_1) refer to here?*

Authors' Response: Corrected.

Original: "…the instantaneous values ($Y_i(t_1)$ at time t_1…) of the detrended data… are shown on the brain plot. The spatial variance (v_1) and spatial root-mean-square (RMS, m_1) value are then derived… for each time instant."

Revised: Fig. S1 caption (SI page 3): "For example, $m_1(t_1)$ and $v_1(t_1)$, where the subscript denotes the time index, are the spatial RMS and variance, respectively, derived at first time point $t = t_1$. Likewise, we derived the values of $m(t)$ and $v(t)$ for every instances."

In SI text (SI page 3): "For example, the instantaneous values of spatial RMS $m_1(t_1)$ and spatial variance $v_1(t_1)$, where the subscript denotes the time index, are derived at first time point $t = t_1$. Likewise, we derived the values of $m(t)$ and $v(t)$ for every times points."

Reviewer Comment: *Table S1 is cropped.*

Authors' Response: Corrected. Table S1 all entries are fully visible in the revised SI document (SI page 29).

Reviewer Comment: *Sect 10.0.1: the mixed usage of metastability as a physical phenomenon, a concept, and as a metric is highly confusing.*

Authors' Response: We have revised Sec. 10.1 (previously Sec 10.0.1) to consistently distinguish: (i) *metastability* as the dynamical phenomenon (transient, recurring switching among quasi-stable coordination states, without settling into a fixed attractor), and (ii) the "*metastability index (meta)"* (denoted *meta*, Eq. S43) as the specific scalar metric, used to quantify that phenomenon. All subsequent uses of the bare word "metastability" where a numeric value is meant have been changed to "metastability index (*meta)*", matching the convention already used for "global phase synchrony (syn)" in the same section. Please refer to SI page 41.

Reviewer Comment: *Caption to Fig S24: exactly how can one fit a scatter plot?*

Authors' Response: Corrected.

Original: *"We have fitted the scatter plots using least square linear regression model, where age is the independent variable."*

Revised: "We fit an ordinary least-squares linear regression line to each scatter plot, with age as the independent variable." (Please refer to SI page 43)

Reviewer Comment: *See my comments above on the reference list.*

Authors' Response: Addressed together with the main reference list. All the necessary corrections have been made carefully in both the main text and supporting information document.
