# Supplementary material for: A generalized scaling law reveals synchrony-driven reorganization of brain dynamics across human lifespan
Source: PLoS Comput Biol. 2026 Oct 5;22(10):e1014821. doi: 10.1371/journal.pcbi.1014821 (PMC13652345; doi:10.1371/journal.pcbi.1014821)
Supplement: Peer review history

# Peer review: Decision Letter 0

**DOI:** 10.1371/journal.pcbi.1014821.r001

**Authors:** Thomas Serre, Adeel Razi

13 Apr 2026

PCOMPBIOL-D-26-00299

A generalized scaling law reveals synchrony-driven reorganization of brain dynamics across the human lifespan

PLOS Computational Biology

Dear Dr. Roy,

Thank you for submitting your manuscript to PLOS Computational Biology. After careful consideration, we feel that it has merit but does not fully meet PLOS Computational Biology's publication criteria as it currently stands. Therefore, we invite you to submit a revised version of the manuscript that addresses the points raised during the review process.

Please submit your revised manuscript by Jun 13 2026 11:59PM. If you will need more time than this to complete your revisions, please reply to this message or contact the journal office at ploscompbiol@plos.org. When you're ready to submit your revision, log on to https://www.editorialmanager.com/pcompbiol/ and select the 'Submissions Needing Revision' folder to locate your manuscript file.

Please include the following items when submitting your revised manuscript:

* A letter that responds to each point raised by the editor and reviewer(s). You should upload this letter as a separate file labeled 'Response to Reviewers'. This file does not need to include responses to formatting updates and technical items listed in the 'Journal Requirements' section below.

* A marked-up copy of your manuscript that highlights changes made to the original version. You should upload this as a separate file labeled 'Revised Manuscript with Track Changes'.

* An unmarked version of your revised paper without tracked changes. You should upload this as a separate file labeled 'Manuscript'.

If you would like to make changes to your financial disclosure, competing interests statement, or data availability statement, please make these updates within the submission form at the time of resubmission. Guidelines for resubmitting your figure files are available below the reviewer comments at the end of this letter

We look forward to receiving your revised manuscript.

Kind regards,

Adeel Razi

Guest Editor

PLOS Computational Biology

Thomas Serre

Section Editor

PLOS Computational Biology

**Journal Requirements:**

1) Please ensure that the CRediT author contributions listed for every co-author are completed accurately and in full.

At this stage, the following Authors/Authors require contributions: Suman Saha, Chittaranjan Hens, Priyanka Chakraborty, Tomasz Kapitaniak, Gustavo Deco, Arpan Banerjee, Syamal Kumar Dana, and Dipanjan Roy. Please ensure that the full contributions of each author are acknowledged in the "Add/Edit/Remove Authors" section of our submission form.

The list of CRediT author contributions may be found here: https://journals.plos.org/ploscompbiol/s/authorship#loc-author-contributions

2) We ask that a manuscript source file is provided at Revision. Please upload your manuscript file as a .doc, .docx, .rtf or .tex. If you are providing a .tex file, please upload it under the item type u2018LaTeX Source Fileu2019 and leave your .pdf version as the item type u2018Manuscriptu2019.

3) Please provide an Author Summary. This should appear in your manuscript between the Abstract (if applicable) and the Introduction, and should be 150-200 words long. The aim should be to make your findings accessible to a wide audience that includes both scientists and non-scientists. Sample summaries can be found on our website under Submission Guidelines:

https://journals.plos.org/ploscompbiol/s/submission-guidelines#loc-parts-of-a-submission

4) Please upload all main figures as separate Figure files in .tif or .eps format. For more information about how to convert and format your figure files please see our guidelines:

https://journals.plos.org/ploscompbiol/s/figures

**Reviewers' comments:**

Reviewer's Responses to Questions

**Comments to the Authors:**

**Please note here if the review is uploaded as an attachment.**

Reviewer #1: This manuscript, , introduces a creative and interesting extension of Taylor’s law to brain data by replacing the mean with RMS, and connects this scaling behavior to brain synchrony. The combination of theory, simulations, and analysis of large fMRI datasets across the lifespan is a clear strength. The idea of linking scaling laws with coordinated brain activity is novel and potentially impactful, and the inclusion of aging and ADHD analyses adds useful real-world relevance. Overall, the paper tackles an important problem and proposes a framework that could be valuable for studying large-scale brain dynamics.

That said, there are a few important issues that need to be addressed. The definition of synchrony is not standard and needs clearer justification, especially in comparison with commonly used measures. Some of the main claims—particularly about the generality of the scaling law—feel a bit too strong given the assumptions involved (like detrending and approximate normality). The heavy reliance on detrending is also concerning, since it may affect the results in ways that are not fully explored. In addition, the interpretation of the scaling exponent remains somewhat unclear from a biological or mechanistic standpoint. The aging and clinical findings are interesting but could be better connected to existing neuroscience literature. Clarifying these points and adding robustness checks would make the paper much stronger.

Reviewer #2: The authors propose a generalization of Taylor’s law to signed multivariate time series data by replacing the mean with the root-mean-square of the detrended data. The authors demonstrate with numerical simulations that the scaling exponent of this generalized spatial Taylor’s law is inversely related to what the authors call “synchrony”. The latter is the averaged (Pearson) correlation matrix (or averaged functional connectivity matrix). The authors investigate the synchrony-scaling relationship (sometimes also referred to as “coupling”) using functional MRI data recorded under various conditions, thereby replicating earlier findings achieved with other, comparable similarity metrics used in neuroimaging. Eventually, the authors claim potential clinical and translational utility of their generalized scaling metric.

This manuscript is not convincing in its present form. It raises more questions than giving answers.

Quite often do the authors make some quite general statements that either lack more detailed information or are not supported by the presented findings. Here’s a quite extensive but definitely incomplete list of phrases that might raise a red flag for the reader: In the context of brain dynamics, what would be the advantage of knowing how the population variance links to the sample mean? What can we learn from that? Why is it important to investigate the effect of synchrony on spatial scaling or spatial fluctuation scaling? Can the authors provide more examples about complex systems – other than the brain – “with integrated and segregated functional networks”? Exactly what can one learn about “deep organizing principles” from scale-invariant fluctuations? Fluctuations of what? Why at all would one expect that “large-scale synchrony shapes brain-wide variability” and what would one learn from that? What would be the advantage or reading out “coordinated dynamics […] from scale-free variability in a principled way”? Aren’t there other (possibly easier) ways to do so? Exactly what would be the advantage of having “synchrony-induced scaling laws as a compact general marker for complex neural systems”? The authors say that “multivariate analysis” (of exactly what?) “significantly enhances the sensitivity of human neuroscience data […].” Sensitivity to exactly what? What exactly are the underlying biophysical (coordination) mechanisms, that the authors often refer to in their manuscript? Can the authors provide an estimate as to how much of the synchrony investigated is indeed emergent and how much is induced/evoked -- either due to intrinsic/external stimuli or due to possible spatial oversampling? After all, exactly what is the added value of the presented approach to understanding the coordinated brain dynamics across the human lifespan or for conditions marked by disrupted neural coordination? What exactly are the mechanistic insights (beyond those afforded by conventional connectivity measures), and exactly why do the authors think that an application of their analysis approach to some fMRI data validates the generalized scaling law? Apart from synchrony, can the authors image other factors that may lead to similar findings?

Quite often do the authors appear to mix the terms synchrony and synchronization, despite their different meanings.

Given different acquisition parameter (e.g. TE/TR times) for the investigated conditions and between datasets, can the authors exclude an influence of these parameters? Likewise, the sizes of the different subnetworks are different. Has this possible influence been controlled for?

On page 2, Abstracts: “the synchrony-scaling relationship […] is particularly strong […]”. How do the authors define “strong”?

On page 4, first sentence: “[…] mean and variance of measurements over locations at different time instances […]” Exactly how do the authors choose the different time instances? (See also page 6).

On page 5, first sentence: to exactly what do the authors refer to with “brain-wide co-ordinated hemodynamics”? Later on, the refer to “coactivation patterns”.

On page 7, first sentence of section “Empirical data distributions …” appears incomplete. The caption to Figure 1 is nor very helpful.

On page 8, Figure 2: given the quite small values of synchrony shown in part d, can the authors provide error bars for their numerically and empirically achieved findings?

On page 9, Figure 3: the raw data shown in parts a and b (what are the units for the amplitudes? Do the authors have an explanation for the gaps in amplitudes?) touch upon a fundamental question of the presented approach: given their positivity, for exactly what do we need a generalization of Taylor’s law to negative-valued data? In the caption: exactly what is a “normal line”?

On page 10, first para.: the authors should check the reported values for time points and participants. The claim “we examine the influence of any emergent coordinated dynamics or synchrony on the spatial SL exponent […]” either needs substantiation or should be weakened. The claim “Factors other than synchrony that may have influenced the exponent are examined from the raw correlations between beta and Omega” either needs substantiation or should be weakened. The phrase “despite possible confounding influences” is another too strong claim.

On page 11, the sentence “the slope beta is estimated by a least squares linear regression fit of the b_syn against the Ω plot” is incomprehensible. When performing regression fits, have the authors controlled for possible outliers (see, e.g. Fig. 4)?

On page 11, the sentence “The parameter beta in three brain states is verified with 100 independent realizations” requires more explanation: exactly what are “realizations” (of what?) and how can the authors assure “independence”?

On page 12, section “Reduced- synchrony scaling…”: what exactly were the criteria to choose the mentioned age ranges? How many subjects entered the respective subgroups and were these of comparable/identical size?

On page 12, section “Preserved synchrony-scaling …”: what exactly were the criteria to choose the mentioned subnetworks? How many voxels entered the respective subnetworks and were these regions of interest of comparable/identical size? If not, could that explain the differences seen between subnetworks?

On page 22, the authors refer to surrogate time series generated via phase randomization. Can the authors provide a motivation for this type of surrogate generation? Why randomizing in the temporal and not in the spatial domain?

The main text, to a large extent, reads like a figure caption (including details about line styles and colors). The discussion section is not a discussion but largely a repetition of the results.

**********

**Have the authors made all data and (if applicable) computational code underlying the findings in their manuscript fully available?**

The PLOS Data policy requires authors to make all data and code underlying the findings described in their manuscript fully available without restriction, with rare exception (please refer to the Data Availability Statement in the manuscript PDF file). The data and code should be provided as part of the manuscript or its supporting information, or deposited to a public repository. For example, in addition to summary statistics, the data points behind means, medians and variance measures should be available. If there are restrictions on publicly sharing data or code —e.g. participant privacy or use of data from a third party—those must be specified.

Reviewer #1: Yes

Reviewer #2: None

**********

PLOS authors have the option to publish the peer review history of their article (what does this mean?). If published, this will include your full peer review and any attached files.

If you choose “no”, your identity will remain anonymous but your review may still be made public.

**Do you want your identity to be public for this peer review?**  For information about this choice, including consent withdrawal, please see our Privacy Policy.

Reviewer #1: No

Reviewer #2: No

[NOTE: If reviewer comments were submitted as an attachment file, they will be attached to this email and accessible via the submission site. Please log into your account, locate the manuscript record, and check for the action link "View Attachments". If this link does not appear, there are no attachment files.]

**Figure resubmission:**

**Reproducibility:**

?>

# Author comment: Author response to Decision Letter 1

**DOI:** 10.1371/journal.pcbi.1014821.r002

15 Jun 2026

Attachment Submitted filename: Response_file_Aging_Revised_26.pdf

# Peer review: Decision Letter 1

**DOI:** 10.1371/journal.pcbi.1014821.r003

**Authors:** Thomas Serre, Adeel Razi

29 Jul 2026

A generalized scaling law reveals synchrony-driven reorganization of brain dynamics across human lifespan

PLOS Computational Biology

Dear Dr. Roy,

Thank you for submitting your manuscript to PLOS Computational Biology. After careful consideration, we feel that it has merit but does not fully meet PLOS Computational Biology's publication criteria as it currently stands. Therefore, we invite you to submit a revised version of the manuscript that addresses the points raised during the review process.

Please submit your revised manuscript by Sep 28 2026 11:59PM. If you will need more time than this to complete your revisions, please reply to this message or contact the journal office at ploscompbiol@plos.org. When you're ready to submit your revision, log on to https://www.editorialmanager.com/pcompbiol/ and select the 'Submissions Needing Revision' folder to locate your manuscript file.

Please include the following items when submitting your revised manuscript:

* A letter that responds to each point raised by the editor and reviewer(s). You should upload this letter as a separate file labeled 'Response to Reviewers'. This file does not need to include responses to formatting updates and technical items listed in the 'Journal Requirements' section below.

* A marked-up copy of your manuscript that highlights changes made to the original version. You should upload this as a separate file labeled 'Revised Manuscript with Track Changes'.

* An unmarked version of your revised paper without tracked changes. You should upload this as a separate file labeled 'Manuscript'.

Kind regards,

Adeel Razi

Guest Editor

PLOS Computational Biology

Thomas Serre

Section Editor

PLOS Computational Biology

**Additional Editor Comments (if provided):**

**Reviewers' comments:**

Reviewer's Responses to Questions

**Comments to the Authors:**

**Please note here if the review is uploaded as an attachment.**

Reviewer #2: The authors have – to a large extent – improved their manuscript. It is more accessible and many of the previously too strong claims were either weakened or supported by additional information/findings. Nevertheless, there are still some old and new issues that require the authors close attention:

Contrary to what is claimed in the response letter, the authors continue to mix the terms “synchronized” and “synchronous” and the text still contains descriptive references (see, e.g. captions to Figs. 2 and 3 and text passages related to these figures).

Abstract, lines 30-34 and 40-43, these sentences are largely incomprehensible.

Abstract, lines 34-35: this is quite a strong claim; detrending does not remove any kind of non-stationarity.

Caption to Fig. 3: if the raw data (amplitude, time) shown are dimensionless, the authors should say so in the caption or add “[a.u.]” to axes labeling.

Line 281: exactly what is a “scatterplot fit”?

Lines 284-285: Contrary to what is claimed in the response letter, the authors have not changed the incomprehensible sentence “the slope beta is estimated by a least squares linear regression fit of the b_syn against the Ω plot”.

Lines 322-341: the newly added text contains many abbreviations whose explanations are only given in the following paragraph. Line 335: do you really mean “differentially”?

Line 346: The abbreviation “ROI” is not defined.

Line 442: Nuerobiological -> Neurobiological

Line 465: snycrony -> synchrony

Line 625: to what does the suffix 1 (v_1, m_1) refers to here?

Line 627: pls use math style for m and v.

Line 652: “when the all regions …” should probably read “when all the regions …”

Line 710: exactly what is a linear regression through a plot?

Lines 722-723: can the authors provide a mathematical argument for why they include self-correlation? Doesn’t this unnecessarily inflate the value of omega?

Line 733: you might want to use |.| instead of abs(.)

Lines 776-778: Although this statement is (theoretically, t-> infinity) correct, the authors should nevertheless acknowledge in the text that large deviations are to be expected given the rather limited observation time (t=261). Replace “Weiner” with “Wiener”.

Line 289: the authors probably mean [0,2pi), if not pls provide an explanation for your choice.

References: Throughout the reference list, the authors use lowercase letters for proper nouns (such as Taylor or Fermi), some physical units (such as T), some journals as well as for standardized abbreviations (such as EEG, MRI, fMRI, etc). This needs to be corrected. Refs [Brown, 2012], [Sastry, 2022], [Shinn, 2023], [Sivagnanam, 2013], and [Yeo, 2011] are incomplete.

The section “Supporting Information Legends” contains many “??”. Undefined references to figures and equations?

Supplemental Material:

The authors should carefully check this document for typos, completeness of sentence, and jargon, and should reconsider their use of the terms synchronization resp. synchronized wherever necessary.

Abstract: The sentence “Section 4 numerically simulated results…” is incomprehensible.

Text beneath caption to Fig. 1: to what does the suffix 1 (v_1, m_1) refers to here?

Table S1 is cropped

Sect 10.0.1: the mixed usage of metastability as a physical phenomenon, a concept, and as a metric is highly confusing.

Caption to Fig S24: exactly how can one fit a scatter plot?

See my comments above on the reference list

**********

**Have the authors made all data and (if applicable) computational code underlying the findings in their manuscript fully available?**

The PLOS Data policy requires authors to make all data and code underlying the findings described in their manuscript fully available without restriction, with rare exception (please refer to the Data Availability Statement in the manuscript PDF file). The data and code should be provided as part of the manuscript or its supporting information, or deposited to a public repository. For example, in addition to summary statistics, the data points behind means, medians and variance measures should be available. If there are restrictions on publicly sharing data or code —e.g. participant privacy or use of data from a third party—those must be specified.

Reviewer #2: None

**********

PLOS authors have the option to publish the peer review history of their article (what does this mean?). If published, this will include your full peer review and any attached files.

If you choose “no”, your identity will remain anonymous but your review may still be made public.

**Do you want your identity to be public for this peer review?**  For information about this choice, including consent withdrawal, please see our Privacy Policy.

Reviewer #2: No

[NOTE: If reviewer comments were submitted as an attachment file, they will be attached to this email and accessible via the submission site. Please log into your account, locate the manuscript record, and check for the action link "View Attachments". If this link does not appear, there are no attachment files.]

**Figure resubmission:**

**Reproducibility:**

?>

# Author comment: Author response to Decision Letter 2

**DOI:** 10.1371/journal.pcbi.1014821.r004

4 Aug 2026

Attachment Submitted filename: Response_to_Reviewers_Round2_2.pdf

# Peer review: Decision Letter 2

**DOI:** 10.1371/journal.pcbi.1014821.r005

**Authors:** Thomas Serre, Adeel Razi

15 Sep 2026

Dear Dr. Roy,

We are pleased to inform you that your manuscript 'A generalized scaling law reveals synchrony-driven reorganization of brain dynamics across human lifespan' has been provisionally accepted for publication in PLOS Computational Biology.

Before your manuscript can be formally accepted you will need to complete some formatting changes, which you will receive in a follow up email. A member of our team will be in touch with a set of requests.

Please note that your manuscript will not be scheduled for publication until you have made the required changes, so a swift response is appreciated.

IMPORTANT: The editorial review process is now complete. PLOS will only permit corrections to spelling, formatting or significant scientific errors from this point onwards. Requests for major changes, or any which affect the scientific understanding of your work, will cause delays to the publication date of your manuscript.

Should you, your institution's press office or the journal office choose to press release your paper, you will automatically be opted out of early publication. We ask that you notify us now if you or your institution is planning to press release the article. All press must be co-ordinated with PLOS.

Thank you again for supporting Open Access publishing; we are looking forward to publishing your work in PLOS Computational Biology.

Best regards,

Adeel Razi

Guest Editor

PLOS Computational Biology

Thomas Serre

Section Editor

PLOS Computational Biology

***********************************************************

Reviewer's Responses to Questions

**Comments to the Authors:**

**Please note here if the review is uploaded as an attachment.**

Reviewer #2: The authors for the most part sufficiently addressed my previous concerns

**********

**Have the authors made all data and (if applicable) computational code underlying the findings in their manuscript fully available?**

The PLOS Data policy requires authors to make all data and code underlying the findings described in their manuscript fully available without restriction, with rare exception (please refer to the Data Availability Statement in the manuscript PDF file). The data and code should be provided as part of the manuscript or its supporting information, or deposited to a public repository. For example, in addition to summary statistics, the data points behind means, medians and variance measures should be available. If there are restrictions on publicly sharing data or code —e.g. participant privacy or use of data from a third party—those must be specified.

Reviewer #2: None

**********

PLOS authors have the option to publish the peer review history of their article (what does this mean?). If published, this will include your full peer review and any attached files.

If you choose “no”, your identity will remain anonymous but your review may still be made public.

**Do you want your identity to be public for this peer review?**  For information about this choice, including consent withdrawal, please see our Privacy Policy.

Reviewer #2: No

# Editor report: Acceptance letter

**DOI:** 10.1371/journal.pcbi.1014821.r006

**Authors:** Thomas Serre, Adeel Razi

PCOMPBIOL-D-26-00299R2

A generalized scaling law reveals synchrony-driven reorganization of brain dynamics across human lifespan

Dear Dr Roy,

I am pleased to inform you that your manuscript has been formally accepted for publication in PLOS Computational Biology. Your manuscript is now with our production department and you will be notified of the publication date in due course.

The corresponding author will soon be receiving a typeset proof for review, to ensure errors have not been introduced during production. Please review the PDF proof of your manuscript carefully, as this is the last chance to correct any errors. Please note that major changes, or those which affect the scientific understanding of the work, will likely cause delays to the publication date of your manuscript.

Soon after your final files are uploaded, unless you have opted out, the early version of your manuscript will be published online. The date of the early version will be your article's publication date. The final article will be published to the same URL, and all versions of the paper will be accessible to readers.

For Research, Software, and Methods articles, you will receive an invoice from PLOS for your publication fee after your manuscript has reached the completed accept phase. If you receive an email requesting payment before acceptance or for any other service, this may be a phishing scheme. Learn how to identify phishing emails and protect your accounts at https://explore.plos.org/phishing.

Thank you again for supporting PLOS Computational Biology and open-access publishing. We are looking forward to publishing your work!

With kind regards,

Sharmila Kamatchi

PLOS Computational Biology | Carlyle House, Carlyle Road, Cambridge CB4 3DN | United Kingdom ploscompbiol@plos.org | Phone +44 (0) 1223-442824 | ploscompbiol.org | @PLOSCompBiol
